# Minor limonoid constituents from *Swietenia macrophylla* by simultaneous isolation using supercritical fluid chromatography and their biological activities†

**DOI:** 10.1039/d4ra03663h

**Published:** 2024-08-22

**Authors:** Kathiravan Asokan, A. Zahir Hussain, Rajesh Kumar Gattu, Andivelu Ilangovan

**Affiliations:** a Aragen Life Sciences Pvt Ltd Bengaluru-562106 India; b Department of Chemistry, Jamal Mohamed College Tiruchirappalli Tamilnadu-620020 India; c School of Chemistry, Bharathidasan University Tiruchirappalli Tamilnadu-620024 India ilangovanbdu@yahoo.com

## Abstract

This study reports simultaneous isolation of three new limonoids (1–3), six known regio isomers (6, 7, 9–12), and three more known limonoids (4, 5, 8) from *Swietenia macrophylla* (*S. macrophylla*) seeds. Structures of these compounds were determined *via* extensive study of their 1D/2D-NMR and mass spectral data. Known limonoids (4–12) were identified by comparing their physical and spectroscopic data with literature values. A novel environmentally friendly supercritical fluid chromatography (SFC) technique facilitated simultaneous and rapid separation of these compounds. The pharmacological activities of the new limonoids were investigated.

## Abstract 2

Three new and nine known limonoids were isolated simultaneously from *Swietenia macrophylla* using supercritical fluid chromatography, and their biological activities were examined.

## Graphical abstract

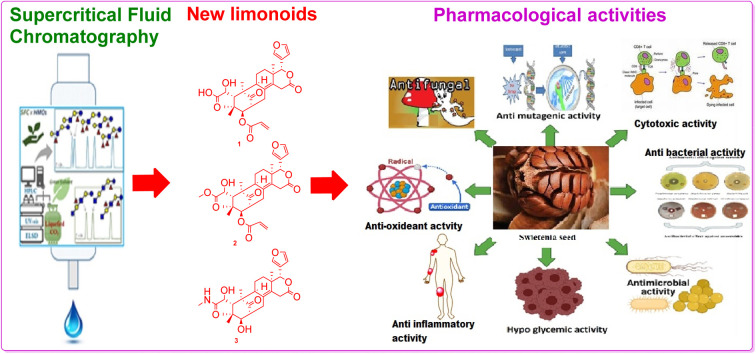

## 1 Introduction

**Fig. 1:**
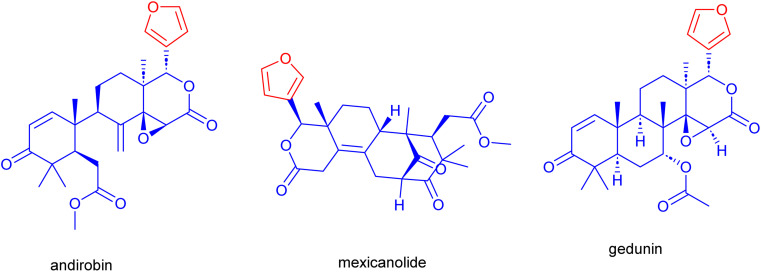
Selected biologically active limonoids (d-ring-opened phragmalin-type).

Mahogany, or *Swietenia macrophylla* (*S. macrophylla*), is a significant medicinal plant cultivated in tropical and subtropical regions. Traditional medicine employs all parts of the *S. macrophylla* to cure a range of human ailments.^1^ The study of the phytochemical components found in *S. macrophylla*'s various plant sections demonstrated the plant's abundance in triterpenes, referred to as limonoids, and their derivatives.^2^ These limonoids are prevalent in citrus fruits of *S. macrophylla* with a bitter taste and sweet or sour scent. With a furan ring attached at the C-17 position, they usually exhibit high degrees of oxidation and skeletal rearrangements. These carbon framework containing limonoids include andirobin, gedunin, mexicanolide, phragmalin, and d-ring-opened phragmalin (Fig. 1).^2*b*^ Purified limonoids from *S. macrophylla* have been employed in several medical treatments such as hypertension,^3^ anti-diabetic,^4,5^ anti-bacterial,^6^ anti-inflammatory, antimicrobial, anti-malarial, anti-oxidant, anti-tumor, treatment of dengue virus^7–10^ and hypolipidemic activity.^11,12^

The primary botanical families known to contain limonoids are Meliaceae, Rutaceae, and Simaroubaceae. Within these plant families, various species have been identified as rich sources of limonoids. These compounds are often found in the seeds, fruits, leaves, and bark of such plant species. More than 300 different limonoids have been identified from numerous plant sources,^13,14^ and there are undoubtedly many more limonoids yet to be found.

Medicinal plants possess complex matrices that allow them to produce compounds showing wide variety of biological activities. Purification, measurement, separation, and characterization of bioactive compounds from extracts of plant material have never been easy. Fortunately, several innovative techniques have emerged that provide compelling evidence for improving the method's sensitivity, selectivity, and run times in the evaluation of therapeutic herbs. Therefore, selecting a simple and appropriate separation technique for natural product separation has become increasingly crucial in recent years.

The development of new, supercritical technology instruments for a wide variety of column chemistries and fluid chromatography, along with innate technological qualities, have made supercritical fluid chromatography (SFC) a stand-in and renowned analytical platform for research on therapeutic plants. The SFC is a green technique, offers several advantages over Prep-HPLC such as easy to use, faster separations, and availability of wide variety of SFC stationary phases with diverging properties. Furthermore, SFC can assess substances that are insoluble in Prep-HPLC solvents, leading to more precise and well-resolved peaks. SFC is a desirable technique for the separation of non-polar natural components such as terpenes, fatty acids, vitamins, sterols, and also moderate-to-strong polar components using modifiers.^15,16^ This technique is particularly well-liked for the enantio separation of chiral compounds.^17^ Supercritical fluids have higher diffusivities and lower viscosities, which make them more efficient, easier to scale up, and need less time to analyse. It makes drying at lower temperatures easier and maintains the stability of the phytochemical elements because of the non-organic solvent systems.^18^ This perspective saw SFC as a promising substitute that safeguards the thermally labile compounds and gives a complimentary chromatography environment. Research to date indicates that the SFC technique has been used to analyze a wide range of phytochemical constituents, including vitamins,^19–21^ triterpenoids,^22–26^ alkaloids,^27–31^ and flavonoids.^32–34^ However, only a few instances SFC purification processes were used to isolate plant extracts such as the ergostane triterpenoids from *Antrodia camphorata*,^35^ polyphenols from *Mangifera indica* Linn,^36^ carsonic acid from rosemary extracts,^37^ and *Piper kadsura*.^38^

Phytochemicals from *S. macrophylla* were traditionally extracted and purified using energy-intensive methods like Prep-HPLC and repeated column chromatography,^15,39^ involving large volumes of petroleum hydrocarbon solvents, harmful to health and the environment.^40–43^ Isolating pure components was laborious, and many compounds degraded during drying. Due to the complexity and abundance, traditional isolation of limonoids from *S. macrophylla* posed challenges, due to their low UV absorbance (steroidal skeleton) and similarity in adsorption behavior.^22^ Availability of no report on the use of SFC for the study of phytochemicals from *S. macrophylla* set out the goal of the current work to use the SFC approach to separate the limonoids from the acetone and ethanol extracts of *S. macrophylla* seeds.

## 2 Results and discussion

**Fig. 2:**
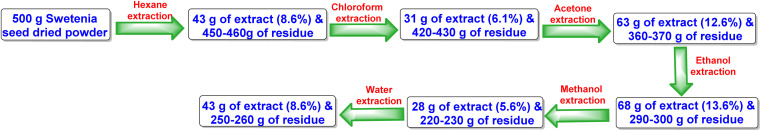
Room temperature extraction of *S. macrophylla* seeds using different solvents.

Harvested fruits of *S. macrophylla* were collected from Salem, Tamilnadu, India, in the northern hemisphere of Asia, which is situated at latitude 11.65376 and longitude 78.15538. After shade drying and grinding the seeds to coarse powder, it was repeatedly defated using hexane at room temperature (Fig. 2) and successively extracted with chloroform, acetone, ethanol, methanol, and water.

**Fig. 3:**
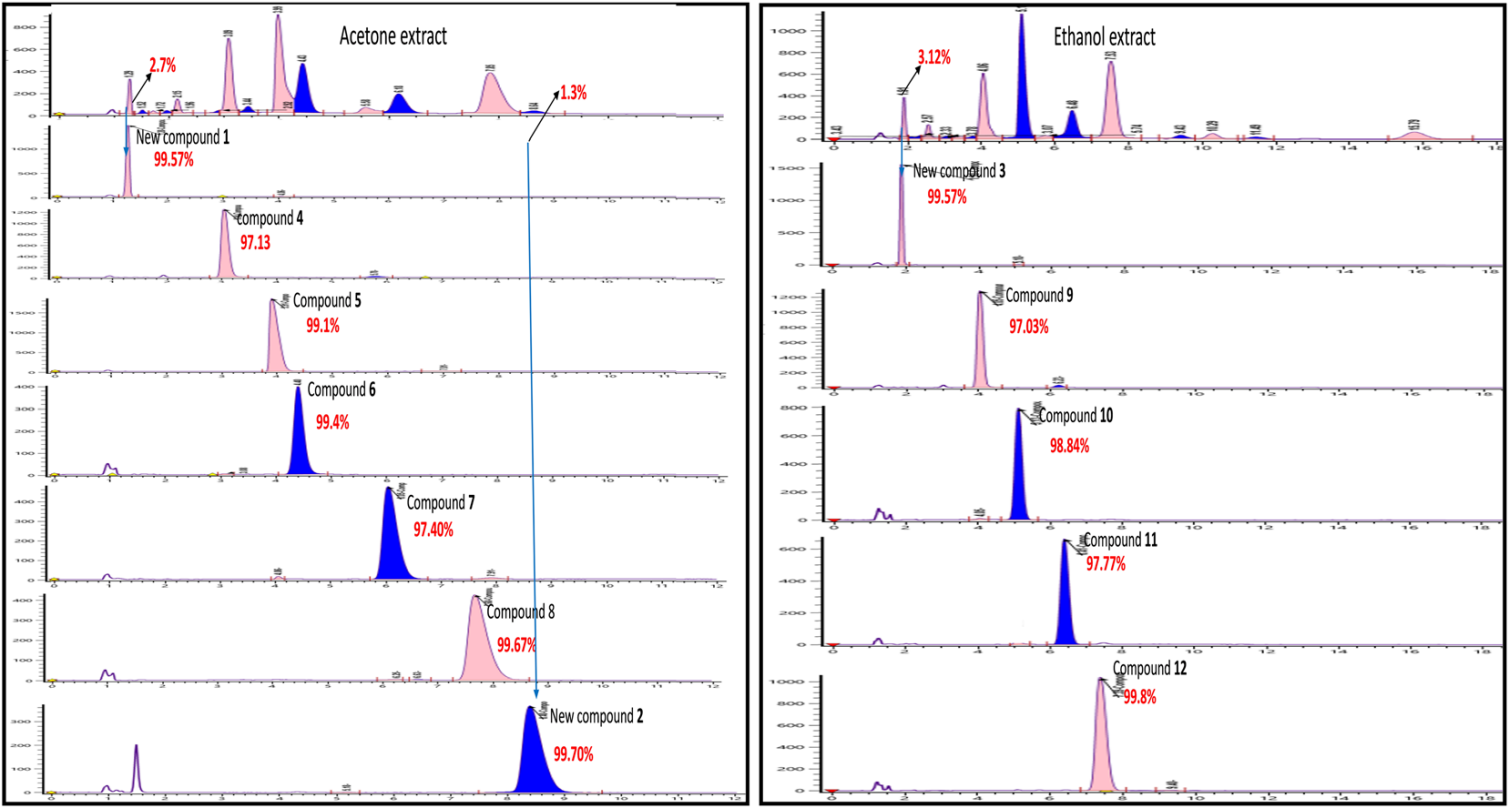
SFC chromatogram purity of compounds 1–12 isolated using Chiralpak IE (4.6 × 250 mm) 5μ column using methanol as co-solvent.

**Fig. 4:**
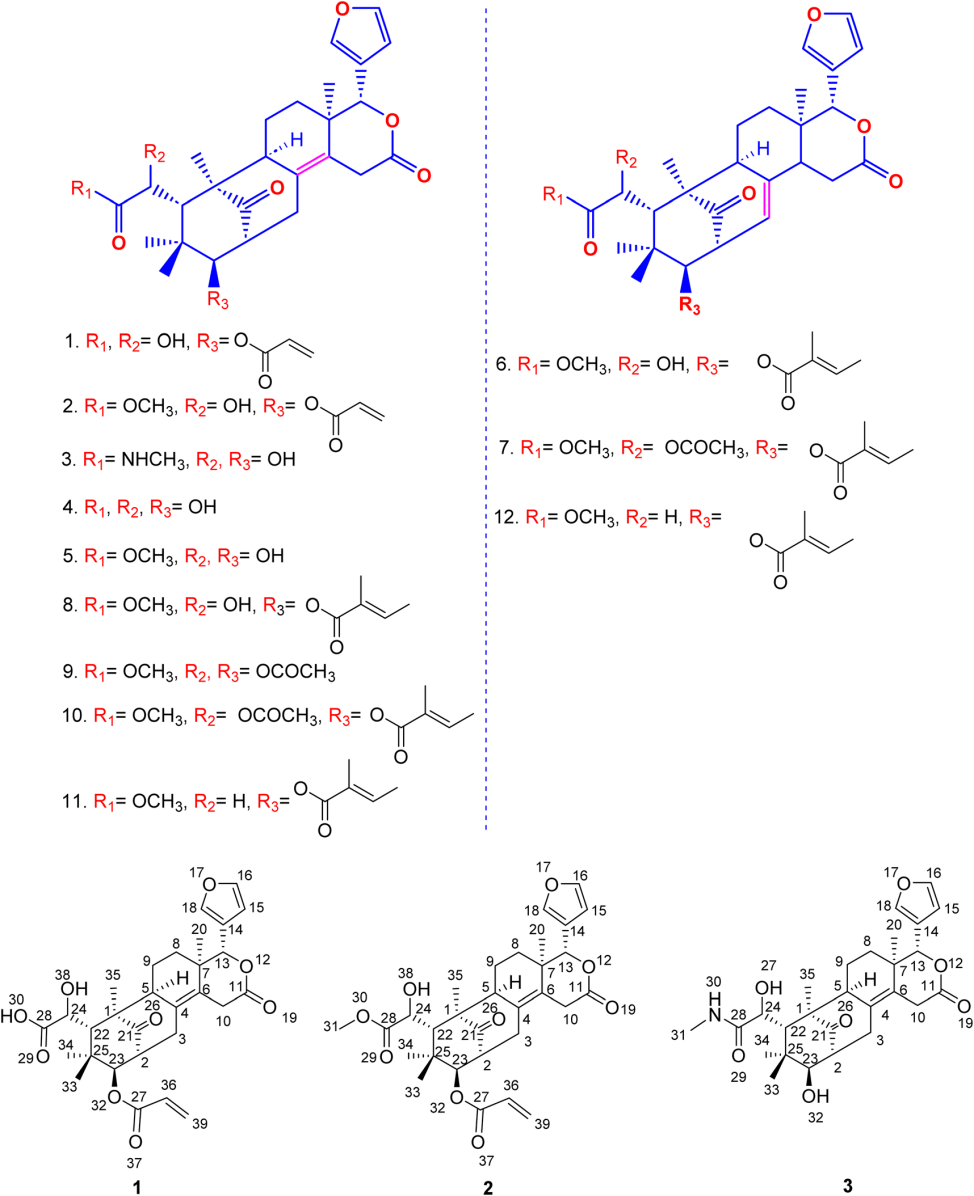
Molecular structure of new and known limonoids isolated from the seeds of *Swietenia macrophylla*.

Even though each extract had a variety of phytoconstituents, the acetone and ethanol extracts which provided higher quantity of crude phytoconstituents were selected for further study. Steps were taken to develop an effective, simultaneous, one-step SFC purification of several phytochemicals. To optimise SFC conditions, several factors encompassing the stationary phase, mobile phase, and other instrumental parameters were considered. Three achiral columns namely, Princeton 2-ethylpyridine, YMC Diol, and Daicel-P4VP, as well as nine chiral columns namely, LUX-i-Amylose-3, Chiralpak AD-H, LUX-Amylose-2, Chiralpak IG, Chiralpak AS-H, Chiral ART Amylose SA, Chiralpak IH, (*R*,*R*) Whelk-O1 and Chiralpak-IE, were examined. Due to the varying stereo-configurations of epimers, chiral columns typically exhibited significantly higher resolutions compared to achiral columns. Stationary phases primarily composed of polysaccharides offer enhanced resolution in separating regio-, *E*/*Z* and non-enantiomeric isomeric mixtures of compounds.^44,45^ Among these polysaccharide phases, the Amylose-based Chiralpak-IE column, specifically amylose tris(3,5-dichlorophenylcarbamate), was identified as providing superior separation of individual limonoid compounds while maintaining reasonable retention times (Fig. 3). After testing a variety of co-solvents, including ethanol, acetonitrile, isopropyl alcohol, the most effective co-solvent was identified as methanol and used along with supercritical carbon dioxide liquid as mobile phase for separating twelve different compounds from acetone and ethanol extracts. Structures of all compounds were determined by the interpretation of their 1D/2D-NMR, and additional spectroscopic data (Fig. 4).

Compound 1 was obtained as an amorphous white solid having Mp 178–180 °C, [*α*]^25^_D_ −180.8 and shows IR (KBr) absorptions at 3501 cm^−1^ (OH) and 1718 cm^−1^ (C=O). The molecular formula of C_29_H_34_O_9_ was determined from positive HR-ESI-MS value *m*/*z* 527.1511. Its structure was supported by ^1^H, ^13^C, HSQC, and HMBC NMR data, revealing features such as proton signals appearing at *δ*_H_ 5.87 (dd, *J* = 10.2, 1.8 Hz, 1H), 6.27 (dd, *J* = 17.2, 1.8 Hz, 1H), 6.07 (dd, *J* = 17.2, 10.2 Hz, 1H) due to the presence of substituted end alkene. Further, protons corresponding to the presence of a furan ring were observed at *δ*_H_ 6.49 (d, 1H), 7.65 (s, 1H), 7.67 (d, 1H), tertiary methyl groups at *δ*_H_ 0.89 (s, 3H), 1.22 (s, 3H), and oxygen-attached methine groups at *δ*_H_ 3.35 (d, 1H, *J* = 9.4 Hz), 4.40 (s, 1H), 5.47 (s, 1H) (Table 1). Notably, 29 peaks corresponding to different carbons, including methyls, methylenes, methines, aliphatic and aromatic quaternary carbons, and carbonyl groups, were observed in their expected *δ*_C_ values. Substituted end alkene group protons appearing at *δ*_H_ 5.87 (dd, *J* = 10.2, 1.8 Hz, 1H), 6.27 (dd, *J* = 17.2, 1.8 Hz, 1H), 6.07 (dd, *J* = 17.2, 10.2 Hz, 1H) were distinguished by HSQC and located at C-27 by the corresponding HMBC correlations of the alkene protons to C-27.

**Table 1:** ^1^H and ^13^C NMR spectral data for compounds 1, 2 and 3. Assignments made based on HSQC and HMBC correlations^a^

Positions	Compound 1		Compound 2		Compound 3	
	*δ*_H_ (*J* in Hz)	*δ*_C_	*δ*_H_ (*J* in Hz)	*δ*_C_	*δ*_H_ (*J* in Hz)	*δ*_C_
1	—	53.5	—	53.5	—	53.3
2	2.80 (m)	50.5	2.79 (m)	50.5	2.80 (m)	50.3
3	1.80, 3.06 (m)	33.7	1.80, 3.06 (m)	33.7	1.81, 3.05 (m)	33.6
4	—	128.8	—	128.8	—	128.6
5	1.89 (m)	52.8	1.88 (m)	52.8	1.89 (m)	52.7
6	—	129.9	—	129.9	—	129.8
7	—	37.6	—	37.6	—	37.1
8	0.95, 1.68 (m)	28.9	0.93, 1.68 (m)	28.9	0.95, 1.68 (m)	28.8
9	1.66, 1.80 (m)	18.4	1.66, 1.80 (m)	18.4	1.66, 1.80 (m)	18.2
10	3.41, 3.90 (m)	33.1	3.29, 3.90 (m)	33.1	3.43, 3.93 (m)	32.9
11	—	170.3	—	170.3	—	170.0
12	—	—	—	—	—	—
13	5.47 (s)	79.6	5.47 (s)	79.6	5.48 (s)	79.3
14	—	121.0	—	121.0	—	120.8
15	6.49 (d)	110.2	6.49 (s)	110.2	6.50 (s)	110.0
16	7.67 (d)	143.5	7.67 (s)	143.5	7.66 (s)	143.3
17	—	—	—	—	—	—
18	7.65 (s)	141.3	7.64 (s)	141.3	7.68 (s)	141.2
19	—	—	—	—	—	—
20	0.90 (s)	18.0	0.89 (s)	18.0	0.90 (s)	17.9
21	—	220.3	—	220.3	—	219.9
22	3.11 (s)	43.6	3.10 (s)	43.6	3.11 (s)	43.4
23	3.36 (d, 9.4 Hz)	77.1	3.35 (d, 9.4 Hz)	77.1	3.36 (dd, 9.4, 4.8 Hz)	76.9
24	4.42 (s)	72.6	4.40 (s)	72.6	4.41 (d, 4.5 Hz)	72.4
25	—	39.6	—	39.6	—	39.4
26	—	—	—	—	—	—
27	—	167.1	—	167.1	5.28 (d, 4.5 Hz)	—
28	—	177.2	—	176.6	—	172.8
29	—	—	—	—	—	—
30	br (moisture)	—	—	—	7.81 (q, 4.0 Hz)	—
31	—	—	3.64 (s)	52.1	2.56 (d, 4.8 Hz)	28.2
32	—	—	—	—	5.17 (d, 4.8 Hz)	—
33	0.84 (s)	23.4	0.84 (s)	23.4	0.84 (s)	23.2
34	0.77 (s)	24.0	0.77 (s)	24.0	0.78 (s)	23.8
35	1.22 (s)	17.9	1.22 (s)	17.9	1.22 (s)	17.7
36	6.07 (dd, 17.20, 10.20 Hz)	129.6	6.07 (dd, 17.20, 10.20 Hz)	129.6		
37	—	—	—	—		
38	br (moisture)	—	br (moisture)	—		
39	5.87 (dd, 10.20, 1.80 Hz)	130.9	5.87 (dd, 10.20, 1.80 Hz)	130.9		
	6.24 (dd, 17.20, 1.80 Hz)		6.24 (dd, 17.20, 1.80 Hz)			

a m: multiplet, t: triplet d: doublet, s: singlet, dd: doublet of doublet, br: broad.

The furan ring protons resonated at *δ*_H_ 6.49 (s, 1H), 7.64 (s, 1H), 7.67 (s, 1H), were distinguished by HSQC and were located at C-13 by the corresponding HMBC correlations of the furan ring group protons to C-13 (oxymethine group). The oxymethine groups at C-13 were assigned based on the HMBC correlations from H-15, H-18, H-20 to C-13, C-23 was assigned based on the HMBC correlations from H-3, H-22, H-33, H-34, and C-24 was assigned based on the HMBC correlations from H-22 to C-24 (Table 2). The HMBC cross-peaks of H-10, H-13, and H-20 were assigned two olefinic quaternary carbon atoms C-4 and C-6. The lactone carbonyl carbon at C-11 was assigned based on the HMBC correlations from H-13, and H-20, and one acid carbonyl carbon C-28 was assigned based on the HMBC correlations from H-22, H-24, while the HMBC cross-peaks of H-3, H-23, H-35 confirmed the most de-shielded keto carbon C-21. The positions of functional groups were discerned through HMBC correlations.

**Table 2:** Key HMBC correlations of new compounds 1, 2 and 3

Position	Compound 1	Compound 2	Compound 3
	HMBC	HMBC	HMBC
2	C-21, C23	C-21, C23	C-21, C23
10	C-4, C-6, C-7, C-11, C13	C-4, C-6, C-7, C-11, C13	C-4, C-6, C-7, C-11, C13
13	C-6, C-11, C-15, C-18, C-20	C-6, C-11, C-15, C-18, C-20	C-6, C-11, C-15, C-18, C-20
15	C-13, C-14, C-16, C-18	C-13, C-14, C-16, C-18	C-13, C-14, C-16, C-18
20	C-6, C-7, C-8, C-13	C-6, C-7, C-8, C-13	C-6, C-7, C-8, C-13
22	C-21, C-23, C-28, C-33 & 34	C-21, C-23, C-28, C-33 & 34	C-21, C-23, C-28, C33 & 34
23	C-21, C-27, C-33 & 34	C-21, C-27, C-33 & 34	C-21, C-27, C-33 & 34
31		C-28	C-28
39	C-27, C-36	C-27, C-36	

Further, compound 2, also was obtained as an amorphous white solid with Mp 188–190 °C, [*α*]^25^_D_ −119.40 and shows characteristic IR (KBr) spectral peaks at 3483 cm^−1^ (OH), 1733 cm^−1^ (C=O), and 1251 cm^−1^ (CO–OCH_3_). The molecular formula of C_30_H_36_O_9_ was determined through HR-ESI-MS observed at *m*/*z* 541.2446 and was supported by the ^1^H, ^13^C, HSQC and HMBC NMR data. The ^1^H-NMR spectrum indicated the presence of a substituted end alkene through the proton peaks appearing at *δ*_H_ 5.87 (dd, *J* = 10.2, 1.8 Hz, 1H), 6.27 (dd, *J* = 17.2, 1.8 Hz, 1H), 6.07 (dd, *J* = 17.2, 10.2 Hz, 1H), a furan ring at *δ*_H_ 6.49 (s, 1H), 7.64 (s, 1H), 7.67 (s, 1H) and two tertiary methyl groups at *δ*_H_ 0.89 (s, 3H), 1.22 (s, 3H), one gem dimethyl group at *δ*_H_ 0.77 (s, 3H), 0.84 (s, 3H), three methine groups attached to oxygen appeared at *δ*_H_ 3.35 (d, 1H, *J* = 9.4 Hz), 4.40 (s, 1H), 5.47 (s, 1H). ^13^C NMR data displayed 30 different carbon resonances, which were resolved into five methyls, five methylenes, ten methines, three aliphatic quaternary carbons, three aromatic quaternary carbons, three ester carbonyl group and a keto carbonyl carbon. HSQC and HMBC correlations located functional groups: alkene (C-27), furan ring (C-13), methoxy (C-31), oxymethine (C-13), aromatic quaternary carbons (C-4, C-6), ester carbonyl (C-11) and keto carbon (C-21).

Similarly, the amide compound 3 was obtained as an amorphous white solid with Mp 195–197 °C, [*α*]^25^_D_ −125.8, and IR (KBr) spectrum of compound 3 displayed peaks at 3426 cm^−1^ (OH), 1649 cm^−1^ (C=O–NH). The molecular formula, C_27_H_35_NO_7_ was as established based on the HR-ESI-MS appearing at *m*/*z* 486.2492 and was supported by the ^1^H, ^13^C, and HSQC, HMBC NMR data. In the ^1^H-NMR spectrum, the secondary amide group protons appeared at *δ*_H_ 7.81 (q, 1H, NH), 2.56 (d, 3H), the presence of furan ring was detected based on the proton peaks appearing at *δ*_H_ 6.50 (s, 1H), 7.66 (s, 1H), 7.68 (s, 1H). Further, two tertiary methyl groups appeared at *δ*_H_ 0.90 (s, 3H), 1.22 (s, 3H), one gem dimethyl group resonated at *δ*_H_ 0.78 (s, 3H), 0.85 (s, 3H), three methine groups attached to oxygen appeared at *δ*_H_ 3.36 (d, 1H, *J* = 9.4 Hz), 4.41 (s, 1H), 5.48 (s, 1H) and two secondary alcohol group protons were found at *δ*_H_ 5.17 (d, 1H, *J* = 4.8 Hz), 5.28 (d, 1H, *J* = 4.5 Hz). Its ^13^C NMR data exhibited 27 carbons signals, which were resolved into five methyls, four methylenes, nine methines, three aliphatic quaternary carbons, three aromatic quaternary carbons, an acid carbonyl carbon, an ester carbonyl carbon and a keto carbonyl carbon by HSQC and HMBC data.

**Fig. 5:**
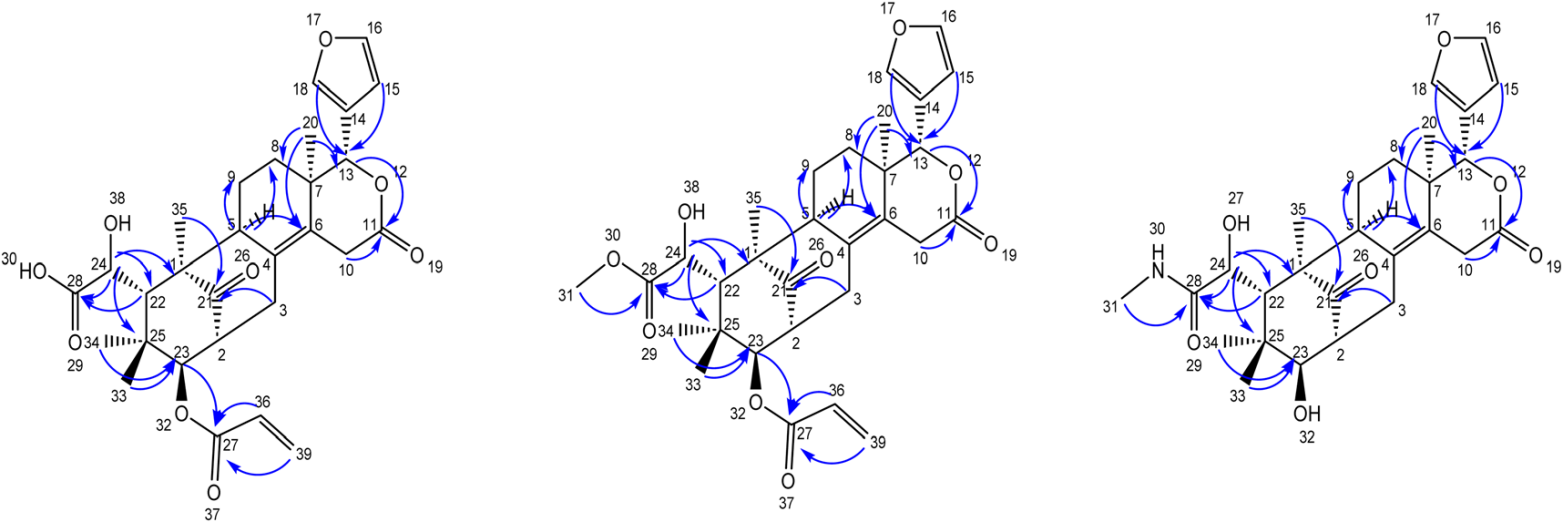
HMBC correlations for new limonoids 1, 2, and 3.

The secondary amide functional group appearing at *δ*_H_ 7.81 (q, 1H, NH), 2.56 (d, 3H) was distinguished by HSQC, nitrogen HSQC and were located at C-28 by the corresponding HMBC correlations of the secondary amide group protons to C-28. HSQC and HMBC correlations was used to locate the functional groups such as furan ring (C-13), oxymethine (C-13), olefinic quaternary carbons (C-4, C-6), ester carbonyl (C-11) and keto carbon (C-21). Many closely related compounds resembling the new compounds 1, 2, and 3, albeit with distinct substitutions at C-22 and C-23, have been previously documented in the literature from *Swietenia macrophylla* seeds. Therefore, the relative configuration of compounds 1, 2, and 3 at C-2, C-13, C-22, C-23, and C-24, as well as the ring junctions, were inferred based on biogenetic analogy with known swietenioides (Fig. 5).^46–55^

### 2.1 Structural identification of the known isolates

The nine compounds demethylisoswietenolide (4),^56,57^ swietenolide (5),^46,47^ swietenine (6),^48,49^ swietenine acetate (7),^49–51^ 3-*O*-tigloylswietenolide (8),^48,49,52^ diacetyl swietenolide (9),^49,53^ 3-*O*-tigloyl-6-*O*-acetyl swietnolide (10),^49,52,54^ khayasin T (11),^49,51,55^ and febrifugine (12).^49,51,52^ isolated along with the unknown compounds were found to be known already in the literature and their structure was established and easily recognized by comparing their physical and spectroscopic data (LCMS, ^1^H-NMR, ^13^C and HMBC & HSQC and [*α*]_D_) with those of the matching authentic samples or literature values.

Thus, overall SFC purification of acetone and ethanol extract resulted in simultaneous isolation and identification of three unknown, new limonoids 1–3, together with nine known compounds.

### 2.2 Biological activities

*S. macrophylla* exhibits a wide range of pharmacological benefits, including antibacterial, anti-inflammatory, antioxidant, antimutagenic, anticancer, antitumor, antidiabetic, anti-nociceptive, hypolipidemic, antidiarrheal, anti-infective, antiviral, antimalarial, acaricidal, and heavy metal phytoremediation activities.^58^ We examined the biological activities of newly discovered compounds 1, 2, and 3, in line with previously isolated compounds from *S. macrophylla*. Since several limonoids from *S. macrophylla* were the subject of thorough theoretical computations and computational analyses,^59,60^ we did not conduct experimental tests or empirical validation to support these theoretical conclusions.

#### 2.2.1. Anti-fungal activity

Triterpenoids, a significant class of constitutive defines phytochemicals found in sufficient concentrations in *S. macrophylla*, are the true cause of the plant's increased ability to combat pathogenic fungus. Antifungal activity of newly isolated compounds 1, 2, and 3 were evaluated against *Aspergillus Niger* using the disc diffusion method, on Sabouraud dextrose agar plates and inoculated them with the fungal culture (Table 3).

**Table 3:** Anti-fungal activity of selected test samples by disc diffusion method^a^

Sl. no	Organism	Control fluconazole	Radius of the zone of inhibition (mm)		
			Compound 1	Compound 2	Compound 3
1	*Aspergillus niger*	25 mm	NI	NI	NI

a NI – no inhibition.

Nevertheless, against *Aspergillus Niger*, none of the test compounds 1, 2 and 3 demonstrated any inhibition at 25 μl (0.05 mg), 50 μl (0.1 mg), 75 μl (0.15 mg), or 100 μl (0.2 mg).^61^ This information suggests that while these compounds may exhibit antibacterial activity while they do not possess the any efficacy against *Aspergillus Niger*, highlighting the importance of considering the target organism when assessing the effectiveness of antimicrobial agents.

#### 2.2.2. Anti-inflammatory activity

The anti-inflammatory assay examines a substance's potential to prevent protein (1% bovine albumin) denaturation, a process linked to inflammatory diseases like arthritis. Denatured proteins can result from tissue damage, exacerbating inflammation. Percentage inhibition of denaturation is calculated using absorption at 660 nm. For the membrane lysis assay, erythrocyte suspension is prepared from human serum albumin (HSA), centrifuged, and washed with saline before reconstitution with an isotonic buffer solution.^62–64^

The anti-inflammatory effects of the newly isolated compounds 1, 2 and 3 (Table 4) revealed that while compounds 2 and 3 showed significantly higher efficacy in causing protein denaturation, compound 1 performed poorly. It was also evident that compound 3's ability to denaturize proteins rises in direct proportion to concentration.

**Table 4:** Anti-inflammatory activity for 25 μl (0.05 mg) concentration^a^

Sl no	Sample ID	Protein denaturation		Hemolytic activity		Heat-induced	
						Haemolytic activity	
		25 μl(0.05 mg)	50 μl(0.1 mg)	25 μl (0.05 mg)	50 μl (0.1 mg)	25 μl (0.05 mg)	50 μl (0.1 mg)
1	Compound 1	—	7%	—	NI	24.30%	27.50%
2	Compound 2	—	14%	34.46%	65.30%	45.62%	52.30%
3	Compound 3	34%	60%	NI	23.43%	48.12%	62.30%

a NI – no inhibition.

Compound 2 exhibited increased hemolytic activity with increase compound concentration, while compound 3 exhibited hemolytic activity, compound 1 exhibited no activity at all. However, the same compounds studied against heat-induced hemolytic activity all the compounds exhibited increased hemolytic activity with increasing concentration.^65,66^

#### 2.2.3. α-Amylase inhibition activity

In order to check hypoglycemic activity, the α-amylase inhibition activity was studied.^67^ Starch was used as a substrate, generation blue color due to the presence of undigested starch was used as an indicator of enzyme inhibition (Table 5). Acarbose served as the positive control. Pre-incubation involved mixing 40 μl of substrate solution with acarbose or compounds 1, 2 and 3 at various concentrations (10–640 μg ml^−1^) before the addition of α-amylase (3 μg ml^−1^). After incubation at 37 °C for 15 minutes, the reaction was terminated with hydrochloric acid, followed by the addition of iodine reagent. Absorbance was then measured at 630 nm.^68,69^

**Table 5:** Alpha amylase % inhibition activity of compounds 1, 2 and 3^a^

S. no	Sample ID	Alpha amylase activity at 25 μl (0.05 mg) concentration	Alpha amylase activity at 50 μl (0.1 mg) concentration
1	Compound 1	20.53%	26.78%
2	Compound 2	16.96%	24.10%
3	Compound 3	28.57%	41.96%

a % inhibition= (absorbance-control − absorbance-test)absorbance-control ×100, where A-control = absorbance of the blank control and A-test = absorbance of the test sample.

Study of alpha-amylase inhibitory potency revealed that compounds 1, 2 and 3 possessed potential hypoglycemic activity. As the concentration of compounds 1, 2 and 3 rises, there is a corresponding increase in the percentage inhibition of alpha-amylase. This suggests that higher concentrations of the compounds lead to greater inhibition of the enzyme's activity.^70,71^ Inhibiting its activity can be desirable for various reasons, such as controlling blood sugar levels in diabetes management or preventing spoilage in food processing.

#### 2.2.4. Antimicrobial activity of the samples using the well diffusion method

The antibacterial activities of compounds 1, 2 and 3 were evaluated against *Escherichia coli* (MTCC 2412), *Bacillus cereus* (MTCC 2128), *Staphylococcus aureus*, and *Klebsiella pneumoniae* (MTCC 2451) using the agar-well diffusion method (Table 6). Ciprofloxacin at 1 mg ml^−1^ was used as the positive reference. Mueller Hinton Agar (MH) plates were sterilized, inoculated with bacterial strains, and then incubated at 37 °C for 24 hours. The diameter of inhibition zones (DIZ) around the wells was measured in millimetres to assess antibacterial activity. Three replicates of each experiment were performed.

**Table 6:** Antibacterial activity of compounds 1, 2 and 3 selected test samples by disc diffusion method (0.05 mg, 0.1 mg, 0.15 mg and 0.2 mg)^a^

Organism	Compound 1				Compound 2				Compound 3				Control
	0.05 mg	0.1 mg	0.15 mg	0.2 mg	0.05 mg	0.1 mg	0.15 mg	0.2 mg	0.05 mg	0.1 mg	0.15 mg	0.2 mg	0.2 mg
*E. coli*	NI	6	7.5	8	NI	NI	6	7	NI	NI	4	NI	12.5
*B. cereus*	NI	NI	5	5	NI	NI	5	6	NI	4.5	6	6	15
*S. aureus*	NI	NI	5	5	NI	NI	5	5	NI	NI	NI	NI	13
*K. pneumoniae*	4	NI	NI	NI	NI	NI	NI	4	NI	5	6	6.5	15

a Disc diffusion method (radius of the zone of inhibition mm).

The antibacterial activity of the compounds 1, 2 and 3 increases with the increase in concentration. This means there are more molecules available to target and inhibit the growth or kill the bacteria. As a result, the antibacterial effect becomes stronger because there's a higher likelihood of these compounds effectively neutralizing or eliminating the bacteria they encounter.

#### 2.2.5. Anti-mutagenic activity by comet assay

The comet assay, or single-cell gel electrophoresis (SCGE), is a method used to quantify DNA damage in individual cells. This is a conventional method used to assess DNA damage and repair, monitor biological samples, and conduct tests to determine the potential for genetic damage.^71^

Compound 1 exhibited notable genotoxic effects on human lymphocytes (Table 7). This suggests that when exposed to compound 1, there was an observable damage to the genetic material of the lymphocytes. Such damage can have serious implications, including increased risk of cancer or other genetic disorders. On the other hand, compounds 2 and 3 show no discernible impacts on human lymphocytes in terms of genotoxicity. This implies that exposure to compounds 2 and 3 does not result in observable genetic damage or mutations in the lymphocytes.

**Table 7:** Genotoxic activity of the compounds on human lymphocytes

Sl. no	Sample ID	Sample concentration	Cell scored	Tail length ± SE
1	Untreated	—	100	0.35 ± 0.02
2	Compound 1	10 μl (0.02 mg)	100	0.41 ± 0.05
		20 μl (0.04 mg)	100	0.45 ± 0.04
3	Compound 2	10 μl (0.02 mg)	100	0.59 ± 0.1
		20 μl (0.04 mg)	100	0.9 ± 0.15
4	Compound 3	10 μl (0.02 mg)	100	0.65 ± 0.015
		20 μl (0.04 mg)	100	01.1 ± 0.37

## 3 Experimental section

### 3.1 General experimental procedures

Using a digital polarimeter (JASCO P-2000), optical rotations were measured. Using a Shimadzu 2000 FT-IR spectrophotometer, IR spectra were acquired. NMR spectra were acquired using a 400 MHz Bruker Avance. A Waters ACQUITY UPLC H-Class coupled with SQ Detector-2 mass spectrometer was used to gather ESIMS data. ESIMS data with high resolution were acquired using an exploris 240 Thermo orbit trap. SFC purifications using Waters SFC-150 mgm equipment and SFC analysis performed on Waters SFC-Investigator instruments. Chiralpak-IE (4.6 × 250 mm; 5μ id) column from Diacel chiral technologies is used for purification, and Chiralpak-IE (4.6 × 250 mm; 5μ) for analytical development.

### 3.2 Plant material

*S. macrophylla* fruits were collected from Salem located at latitude 11.65376 and longitude 78.15538. It is part of Asia and the northern hemisphere, Tamilnadu, India, and the seeds were removed by peeling them.

### 3.3 Extraction and isolation

After being processed using an electronic grinder for a week to a coarse powder, the seeds were weighed, shade-dried, and stored in a dry place. 500 g of dry powder were continuously cold extracted using hexane residue, followed by chloroform, acetone, ethanol, methanol, and water three times each. A rotary evaporator was utilized to eliminate the solvents from every extract. The extracts were then kept at −70 °C for 48 h, and a freeze-dryer (Labconco Corporation, Denmark) was used to freeze-dry them under a vacuum for 24 h at −40 °C. Strictly sealed glass bottles containing each dried extract were kept at 4 °C. The extraction yield from *S. macrophylla* seeds was found to be highest in ethanol solvent (68 g with 13.6%) and lowest in aqueous solvent (28.0 g with 5.6%), according to a quantitative evaluation of the extracts observed from the seeds using different solvents.

### 3.4 Optimizing SFC conditions for efficient phytochemical separation

We focused on developing an effective one-step SFC method for isolating phytochemicals, prioritizing compounds separable by acetone and ethanol. SFC offers superior separation efficiency and additional benefits such as online coupled processes, faster separations, reduced solvent consumption, wide applicability, and easy analyte recovery.

SFC conditions were optimized, including stationary phase, mobile phase, and instrumental parameters, for separating limonoids from *S. macrophylla* seed extracts. Chiral columns showed higher resolution than achiral ones due to the presence of various positional isomers.

The Chiralpak-IE column was identified as optimal for limonoid separation using methanol as the most effective solvent. Key parameters included a column temperature of 35 °C, a 30% co-solvent composition, a sample volume of 10 μl, a flow rate of 4.0 ml min^−1^, and 100 bars of back pressure. These optimized conditions were applied in the analytical SFC for isolating compounds from ethanol and acetone extracts. Seven compounds 1, 2, 4, 5, 6, 7, and 8 were isolated from acetone extract, and five compounds 3, 9, 10, 11, and 12 from ethanol extract, even with extremely low abundances (2–3%), demonstrating the efficiency of the SFC approach.

The SFC purification was not performed for extracts of hexane, chloroform, methanol, and water due to their low solubility, poor peak shape, and retention behaviour. The purity of isolated compounds was confirmed using SFC investigation, and certain compounds were disregarded for further research as they were well-known and extensively reported in the literature. Initial LCMS analysis confirmed the presence of the same compounds in multiple extracts, leading to the selection of ethanol and acetone extracts for SFC purification.

## 4 Conclusion

In conclusion, we demonstrated for the first time that SFC could be used as a powerful technique for the simultaneous and superior separation of twelve compounds, in particular limonoids from *S. macrophylla* in a single-step. An optimum condition was arrived at based the study of different parameters such as column, pressure, temperature, and co-solvent composition. Compounds 1, 2, and 3 were previously unknown and challenging to isolate by conventional natural products separation methods. This study further shows that using the SFC isolation technique, even trace-level (2–3%) phyto constituents such as limonoids 1, 2, 3, 9, 10, 11 and 12 could be conveniently separated from complex mixtures. The use of recyclable carbon dioxide as the primary solvent, reduced use of organic solvent, efficient and simultaneous separation of minor natural product constituents by means SFC makes our separation technique sustainable, green and eco-friendly. These results are unattainable through traditional chromatographic methods, efficient alternative, and are unprecedented in the literature on *S. macrophylla*.

Further, the new limonoids 1, 2 and 3 assessed for antimicrobial, anti-inflammatory, hemolytic, and genotoxic properties disclose novel biological activities. Compounds 1, 2 and 3 shows good hypoglycemic activity useful in controlling blood sugar levels and diabetes management, significant antibacterial activity and no anti-fungal activity. Compounds 2 and 3 showed significant anti-inflammatory activity and anti-mutagenic activity. Whereas compound 1 showed poor anti-inflammatory activity and genotoxic effects on human lymphocytes. Studying mechanism and therapeutic effects of the limonoids isolated from *S. macrophylla*, could facilitate the discovery of potential new drugs.

## Data availability

All spectral data and other information supporting the contents of the main manuscript can be found in the ESI.†

## Conflicts of interest

The authors declared that they have no conflict of interest.

## Supplementary Material

RA-014-D4RA03663H-s001
