# Supplementary material for: Minor limonoid constituents from Swietenia macrophylla by simultaneous isolation using supercritical fluid chromatography and their biological activities
Source: RSC Adv. 2024 Aug 22;14(36):26637–47. doi: 10.1039/d4ra03663h (PMC11339773; doi:10.1039/d4ra03663h)
Supplement: RA-014-D4RA03663H-s001 [file RA-014-D4RA03663H-s001.pdf]

Supplementary Information (SI) for RSC Advances.
This journal is © The Royal Society of Chemistry 2024

Supporting Information

Minor limonoid constituents from *Swietenia macrophylla* by Simultaneous isolation using supercritical fluid chromatography and their biological activities

Kathiravan Asokan,[1,2,3] A. Zahir Hussain,[2] Rajesh Kumar Gattu,[3] Andivelu Ilangovan[4]*

[1,3]Aragen Life Sciences Pvt. Ltd. Bengaluru-562106, India.

[2]Department of Chemistry, Jamal Mohamed College, Tiruchirappalli, Tamilnadu-620020, India.

[4]School of Chemistry, Bharathidasan University, Tiruchirappalli, Tamilnadu-620024, India.

Email: ilangovanbdu@yahoo.com

Table of Contents

1. Molecular structures compounds **1-12**....4

2. Experimental Procedure and Spectral data for compounds **1-12** 5

Figure S1. SFC screening chromatogram of crude material of *Swietenia macrophylla* seed. 12

Figure S2. Optimized SFC method for Acetone and ethanol extract of *Swietenia macrophylla* seed.41

Figure S3. ^{1}H NMR Spectrum of compound **1** at (400 MHz) in DMSO- *d6* 42

Figure S4. ^{13}C NMR NMR Spectrum of compound **1** at (400 MHz) in DMSO- *d6*....43

Figure S5. HSQC Spectrum of compound **1** at (400 MHz) in DMSO- *d6* 44

Figure S6. HMBC Spectrum of compound **1** at (400 MHz) in DMSO- *d6* 45

Figure S7. HRESI mass spectrum of compound **1** 46

Figure S8. IR spectrum of compound **1** 47

Figure S9. Optical rotation of compound **1** 48

Figure S10. UV Spectra of compound **1**....49

Figure S11. ^{1}H NMR Spectrum of compound **2** at (400 MHz) in DMSO- *d6* 50

Figure S12. ^{13}C NMR Spectrum of compound **2** at (400 MHz) in DMSO- *d6* 51

Figure S13. HSQC Spectrum of compound **2** in DMSO- *d6* 52

Figure S14. HMBC Spectrum of compound **2** in DMSO- *d6*....53

Figure S15. HRESI Mass spectrum of compound **2**....54

Figure S16. IR spectrum of compound **2** 55

Figure S17. Optical rotation of compound **2** 56

Figure S18. UV spectra of compound **2**....57

Figure S19. ^{1}H NMR Spectrum of compound **3** at (400 MHz) in DMSO- *d6* 58

Figure S20. ^{13}C NMR Spectrum of compound **3** at (400 MHz) in DMSO- *d6*....59

Figure S21. HSQC Spectrum of compound **3** in DMSO- *d6* 60

Figure S22. HMBC Spectrum of compound **3** in DMSO- d6 61
Figure S23. N15 HSQC Spectrum of compound **3** in DMSO- *d6* 62
Figure S24. HRESI Mass spectrum of compound **3** 63
Figure S25. IR spectrum of compound **3** 64
Figure S26. Optical rotation of compound **3** 65
Figure S27. UV spectrum of compound **3** 66
Figure S28. ^{1}H NMR Spectrum of compound **4** at (400 MHz) in DMSO- *d6* 67
Figure S29. ^{13}C NMR Spectrum of compound **4** at (400 MHz) in DMSO- d6 68
Figure S30. HSQC Spectrum of compound **4** in DMSO- *d6* 69
Figure S31. HMBC Spectrum of compound **4** in DMSO- d6 70
Figure S32. HRESI Spectrum of compound **4** 71
Figure S33. ^{1}H NMR Spectrum of compound **5** at (400 MHz) in DMSO- *d6* 72
Figure S34. ^{13}CNMR Spectrum of compound **5** at (400 MHz) in DMSO- *d6* 73
Figure S35. HSQC Spectrum of compound **5** in DMSO- *d6* 74
Figure S36. HMBC Spectrum of compound **5** in DMSO- *d6* 75
Figure S37. HRESI Mass Spectrum of compound **5** 76
Figure S38. ^{1}H NMR Spectrum of compound **6** at (400 MHz) in DMSO- *d6* 77
Figure S39. ^{13}C NMR Spectrum of compound **6** at (400 MHz) in DMSO- *d6* 78
Figure S40. HSQC Spectrum of compound **6** in DMSO- *d6* 79
Figure S41. HMBC Spectrum of compound **6** in DMSO- *d6* 80
Figure S42. LCESI Mass Spectrum of compound **6** 81
Figure S43. ^{1}H NMR Spectrum of compound **7** at (400 MHz) in DMSO- *d6* 82
Figure S44. ^{13}C NMR Spectrum of compound **7** at (400 MHz) in DMSO- *d6* 83
Figure S45. HSQC Spectrum of compound **7** in DMSO- *d6* 84
Figure S46. HMBC Spectrum of compound **7** in DMSO- *d6* 85
Figure S47. HRMS-ESI Mass Spectrum of compound **7** 86
Figure S48. ^{1}H NMR Spectrum of compound **8** at (400 MHz) in DMSO- *d6* 87
Figure S49. ESI LCMS spectrum of compound **8** 88
Figure S50. ^{1}H NMR Spectrum of compound **9** at (400 MHz) in DMSO- *d6* 89
Figure S51. ^{13}C NMR Spectrum of compound **9** at (400 MHz) in DMSO- *d6* 90
Figure S52. HSQC Spectrum of compound **9** in DMSO- *d6* 91
Figure S53. HMBC Spectrum of compound **9** in DMSO- d6 92
Figure S54. LC-ESI Mass Spectrum of compound **9** 93
Figure S55. ^{1}H NMR Spectrum of compound **10** at (400 MHz) in DMSO- *d6* 94
Figure S56. ^{13}C NMR Spectrum of compound **10** at (400 MHz) in DMSO- *d6* 95
Figure S57. HSQC Spectrum of compound **10** in DMSO- d6 96

Figure S58. HMBC Spectrum of compound **10** in DMSO- d6 97
Figure S59. ESI LCMS Spectrum of compound **10**....................98
Figure S60. [1] H NMR Spectrum of compound **11** at (400 MHz) in DMSO- d6 99
Figure S61. ^{13}C NMR Spectrum of compound **11** at (400 MHz) in DMSO- d6 100
Figure S62. HSQC Spectrum of compound **11** in DMSO- d6 101
Figure S63. HMBC Spectrum of compound **11** in DMSO- d6 102
Figure S64. HRESI Spectrum of compound **11** 103
Figure S65. [1] H NMR Spectrum of compound **12** at (400 MHz) in DMSO- d6 104
Figure S66. HSQC Spectrum of compound **12** in DMSO- d6 105
Figure S67. HSRMS Spectrum of compound **12** 106
Pharmacological activities 107
Figure S62. Antibacterial activity 109
Figure 68. Antifungal activity....................115
Figure 69. Anti-mutagenic activity 115
References....................117

1. Molecular structures compounds **1-12**

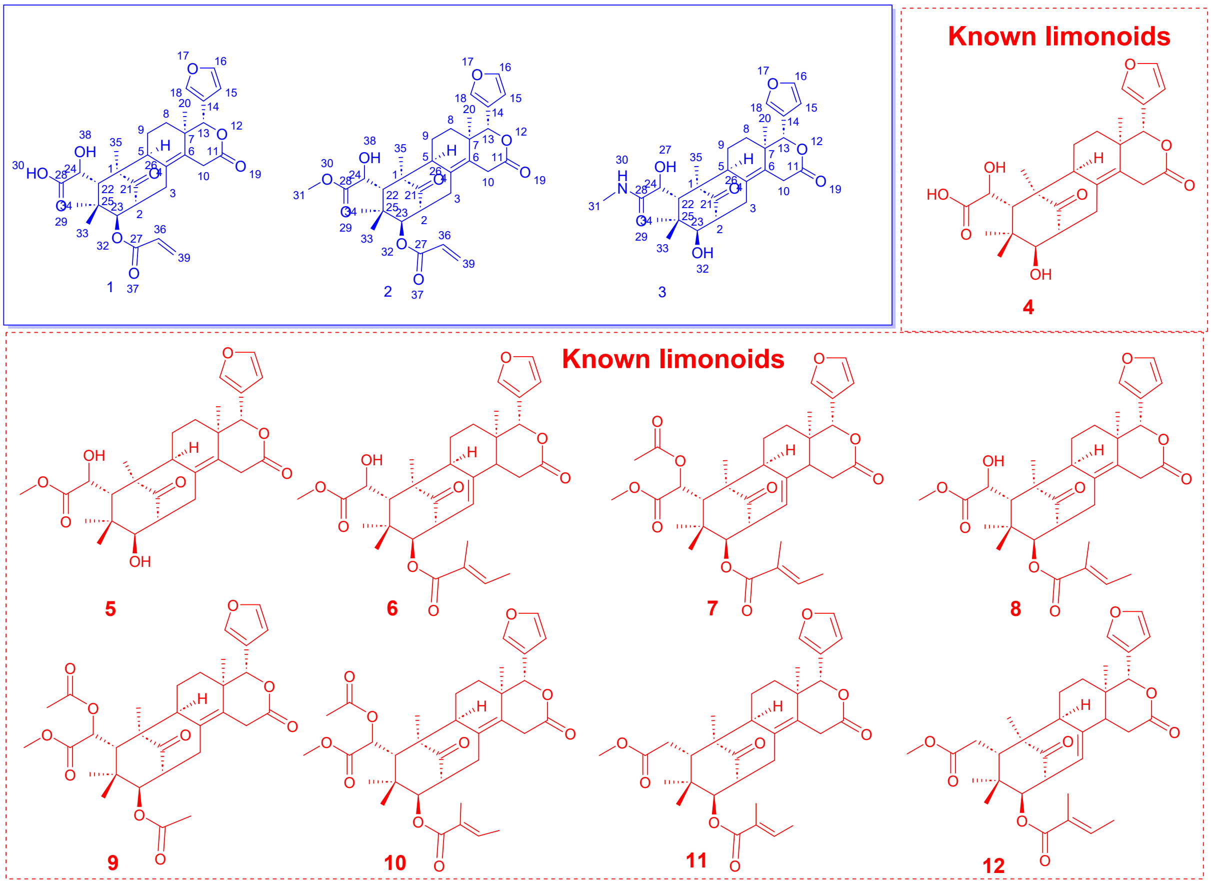

Figure 1. Molecular structure of three new limonoids and nine known limonoids isolated from acetone and ethanol extracts of the seeds of *Swietenia macrophylla.*

2. Experimental Procedure and Spectral data for compounds **1-12**

All the reagents were purchased commercially and used without further purification. ^{1}H NMR and ^{13}C NMR were recorded with Bruker 400 MHz. ^{1}H NMR (400MHz) and ^{13}C NMR (100MHz) spectra were recorded in $CDCl_3$ with tetramethylsilane as the internal standard. All the NMR spectra were acquired at ambient temperature. Analytical thin layer chromatography (TLC) was performed using Silica Gel 60 Å F_{254} pre-coated plates (0.25 mm thickness). High resolution mass spectra (HRMS) were recorded on Bruker Compass Data Analysis 4.1, HRMS-ESI Mass Spectrometer with Orbitrap Exploris-240 Analyzer, Source Type ESI, in positive mode.

Materials and Methods:

Chemicals and reagents:

The HPLC grade chemicals utilized in the study such as methanol (MeOH), acetonitrile (ACN), Trifluoroacetic acid (TFA), diethylamine (DEA) and 7N Methanolic ammonia, isopropyl alcohol (IPA) were purchased from Sigma-Aldrich Co. (Merck-INDIA).

Instrumentation:

Analytical Acquity UPC2 PDA (Waters) with a six-position modifier and column switching valves, Thar's automated SFC method development stations with a six-position modifier and a ten-position column switching valves, and Waters SFC150Mgm prep were all purchased from Waters (WATERS GES MBH, W-Austria). Waters ACQUITY H-Class UPLC equipment coupled with Waters SQ Detector-2 was used for the purity analysis and mass identification of compounds. Achiral and chiral Columns were purchased from Daicel, Waters, YMC, Phenomenex and Regis Technologies.

Collection of plant material and preparation of crude extract from seed powder:

Swietenia macrophylla fruits were collected from the Salem is located at latitude 11.65376 and longitude 78.15538. It is part of Asia and the northern hemisphere, Tamilnadu, India, and their seeds were removed by peeling them. After being processed using an electronic grinder for a week to a coarse powder, the seeds were weighed, shade-dried, and stored in a dry place. 500 g of dry powder were continuously Soxhlet-extracted using hexane residue, followed by chloroform, acetone, ethanol, methanol, and water three times each at 40–50 °C. A rotary evaporator was utilized to eliminate the solvents from every extract. The extracts were then kept at −70°C for 48 hours, and a freeze-dryer (Labconco Corporation,

Denmark) was used to freeze-dry them under a vacuum for 24 hours at −40°C. Strictly sealed glass bottles containing each dried extract were kept at 4°C.

Method of extraction	Solvent Extracts (gram)	Solvent used	Extracts	Yield (%)
Cold extraction	500 g	Hexane	43 g	8.6
		Chloroform	31g	6.2
		Acetone	63 g	12.6
		Ethanol	68 g	13.6
		Methanol	43 g	8.6
		Water	28 g	5.6

Spectral data for Compounds 1-12

2-((4R,4aR,7R,10R)-10-(acryloyloxy)-4-(furan-3-yl)-4a,7,9,9-tetramethyl-2,13-dioxo-1,4,4a, 5, 6, 6a, 7, 8,9,10,11,12b-dodecahydro-2H-7,11-methanocycloocta[f]isochromen-8-yl)-2-hydroxyacetic acid (1)

Compound **1** is an amorphous white solid with a melting point of 178-180°C. Its molecular formula is $C_{29}H_{34}O_9$ as determined by positive HR-ESI-MS (m/z 527.1511). The IR spectrum (KBr) shows absorption bands at 3501 cm^{-1} (OH) and 1718 cm^{-1} (C=O). The optical rotation $[\alpha]^{25}_{D}$ -180.8.

The structure of Compound **1** was supported by ^{1}H, ^{13}C, HSQC, and HMBC NMR data. Key structural features include:

- A substituted end alkene (δ_H 5.87 (dd, J = 10.2, 1.8 Hz), 6.27 (dd, J = 17.2, 1.8 Hz), 6.07 (dd, J = 17.2, 10.2 Hz)) located at C-27.
- A furan ring (δ_H 6.49 (s), 7.64 (s), 7.67 (s)) located at C-13, with an oxymethine group.
- Tertiary methyl groups (δ_H 0.89 (s), 1.22 (s)).
- Oxygen-attached methine groups (δ_H 3.35 (d, J = 9.4 Hz), 4.40 (s), 5.47 (s)).

Carbon resonances include methyls, methylenes, methines, aliphatic and aromatic quaternary carbons, as well as carbonyl groups. Specific assignments were made using HMBC correlations, such as:

- C-13 (furan ring) correlated with H-15, H-18, H-20.
- C-23 correlated with H-3, H-22, H-33, H-34.
- C-24 correlated with H-22.
- C-4 and C-6 (aromatic quaternary carbons) correlated with H-10, H-13, H-20.
- C-11 (ester carbonyl carbon) correlated with H-13, H-20.
- C-28 (acid carbonyl carbon) correlated with H-22, H-24.
- C-21 (most deshielded keto carbon) correlated with H-3, H-23, H-35.

(4R,4aR,7R,10R)-4-(furan-3-yl)-8-(1-hydroxy-2-methoxy-2-oxoethyl)-4a,7,9,9-tetramethyl-2,13-dioxo-1,4,4a,5,6,6a,7,8,9,10,11,12b-dodecahydro-2H-7,11-methanocycloocta[f]isochromen-10-yl acrylate (2)

Compound **2** is an amorphous white solid with a melting point of 188-190°C. Its molecular formula, $C_{30}H_{36}O_9$, was determined by HR-ESI-MS (m/z 541.2446). The IR spectrum (KBr) shows absorption bands at 3483 cm^{-1} (OH), 1733 cm^{-1} (C=O), and 1251 cm^{-1} (CO-OCH3). The optical rotation $[\alpha]^{25}_{D}$ -119.40.

The ^{1}H NMR spectrum of Compound **2** indicated the presence of:

- A substituted end alkene at δ_H 5.87 (dd, J = 10.2, 1.8 Hz, 1H), 6.27 (dd, J = 17.2, 1.8 Hz, 1H), 6.07 (dd, J=17.2, 10.2 Hz, 1H).
- A furan ring at δ_H 6.49 (s, 1H), 7.64 (s, 1H), 7.67 (s, 1H).
- Two tertiary methyl groups at δ_H 0.89 (s, 3H), 1.22 (s, 3H).
- One gem-dimethyl group at δ_H 0.77 (s, 3H), 0.84 (s, 3H).
- Three oxygen-attached methine groups at δ_H 3.35 (d, J = 9.4 Hz, 1H), 4.40 (s, 1H), 5.47 (s, 1H).

The ^{13}C NMR data showed 30 carbon signals, resolved into five methyls, five methylenes, ten methines, three aliphatic quaternary carbons, three aromatic quaternary carbons, three ester carbonyl groups, and one keto carbonyl carbon, as supported by HSQC and HMBC data. Key structural assignments include:

- The substituted end alkene at δ_H 5.87, 6.27, 6.07, located at C-27 through HMBC correlations.
- The furan ring protons at δ_H 6.49, 7.64, 7.67, located at C-13 by HMBC correlations.
- A methoxy group at C-31 assigned based on cross-peaks from H-31 to C-28.

- Oxymethine groups at C-13 assigned by HMBC correlations from H-15, H-18, H-20 to C-13.
- C-23 assigned by HMBC correlations from H-3, H-22, H-33, H-34.
- C-24 assigned by HMBC correlations from H-22.
- Aromatic quaternary carbons C-4 and C-6 assigned by HMBC cross-peaks of H-10, H-13, H-20.
- An ester carbonyl carbon at C-11 assigned by HMBC correlations from H-13, H-10.
- The most deshielded keto carbon at C-21 confirmed by HMBC cross-peaks of H-3, H-23, H-35

2-((4R,4aR,10R)-4-(furan-3-yl)-10-hydroxy-4a,7,9,9-tetramethyl-2,13-dioxo-1,4,4a,5,6,6a,7,8,9,10,11,12-dodecahydro-2H-7,11-methanocycloocta[f]isochromen-8-yl)-2-hydroxy-N-methylacetamide (3)

Compound **3** is an amorphous white solid with a melting point of 195-197°C. Its molecular formula, $C_{27}H_{35}NO_7$, was determined by HR-ESI-MS (m/z 486.2492). The IR spectrum (KBr) shows absorption bands at 3426 cm^{-1} (OH) and 1649 cm^{-1} (C=O-NH). The optical rotation $[\alpha]^{25}_{D}$ -125.8.

The ^{1}H NMR spectrum indicated:

- A secondary amide functional group at δ_H 7.81 (q, 1H, NH), 2.56 (d, 3H).
- A furan ring at δ_H 6.50 (s, 1H), 7.66 (s, 1H), 7.68 (s, 1H).
- Two tertiary methyl groups at δ_H 0.90 (s, 3H), 1.22 (s, 3H).
- One gem-dimethyl group at δ_H 0.78 (s, 3H), 0.85 (s, 3H).
- Three oxygen-attached methine groups at δH 3.36 (d, *J* = 9.4 Hz, 1H), 4.41 (s, 1H), 5.48 (s, 1H).
- Two secondary alcohol groups at δH 5.17 (d, *J* = 4.8 Hz, 1H), 5.28 (d, *J* = 4.5 Hz, 1H).

The ^{13}C NMR data revealed 27 carbon signals, resolved into five methyls, four methylenes, nine methines, three aliphatic quaternary carbons, three aromatic quaternary carbons, an acid carbonyl group, an ester carbonyl group, and a keto carbonyl carbon, supported by HSQC and HMBC data. Key structural assignments include:

- The secondary amide functional group located at C-28 through HMBC correlations.
- The furan ring protons at δ_H 6.50, 7.66, 7.68, located at C-13 by HMBC correlations.
- Oxymethine group at C-13 assigned by HMBC correlations from H-15, H-18, H-20.
- C-23 assigned by HMBC correlations from H-3, H-22, H-33, H-34.
- C-24 assigned by HMBC correlations from H-22.
- Aromatic quaternary carbons C-4 and C-6 assigned by HMBC cross-peaks of H-10, H-13, H-20.
- Ester carbonyl carbon at C-11 assigned by HMBC correlations from H-10, H-13.
- The most deshielded keto carbon at C-21 confirmed by HMBC cross-peaks of H-3, H-23, H-35

2-((4R,4aR,10R)-4-(furan-3-yl)-10-hydroxy-4a,7,9,9-tetramethyl-2,13-dioxo-1,4,4a,5,6,6a,7,8,9,10,11,12-dodecahydro-2H-7,11-methanocycloocta[f]isochromen-8-yl)-2-hydroxyacetic acid (4)

^{1}H-NMR (400MHz, DMSO) δ 7.67 (q, *J* = 3.5Hz, 2H),6.50 (d, *J* = 1.1Hz, 1H), 5.48 (s, 1H), 4.40 (s, 1H),3.90 (d, *J* = 20.9Hz, 1H),3.39 (q, *J* = 11.3Hz, 2H),3.08 (m, *J*=6.4Hz, 2H), 2.80 (m,*J* = 2.8Hz, 1H),1.84 (q, *J* = 14.5Hz, 3H),1.68 (t, *J* = 8.5Hz, 2H),1.23 (s, 3H), 0.90 (m, 4H),0.85 (s, 3H),0.78 (s, 3H). [1,12]

Methyl 2-((4R,4aR,7R,10R)-4-(furan-3-yl)-10-hydroxy-4a,7,9,9-tetramethyl-2,13-dioxo-1,4,4a,5,6,6a,7,8,9,10,11,12-dodecahydro-2H-7,11-methanocycloocta[f]isochromen-8-yl)-2-hydroxyacetate (5)

^{1}H-NMR (DMSO) 0.78 (3H, s), 0.85 (3H, s), 0.90 (3H, s), 0.94 (1H, t, *J* = 4.40 Hz), 1.23 (3H, s), 1.68 (2H, t, *J* = 9.05 Hz), 1.78 (2H, m, *J* = 4.98 Hz), 1.89 (1H, s), 2.79 (1H, q, *J* = 3.14 Hz), 3.07 (1H, q, *J* = 5.46 Hz), 3.11 (1H, s), 3.37 (2H, t, *J* = 4.80 Hz), 3.66 (3H, s), 3.91 (1H, d, *J* = 20.93 Hz), 4.41 (1H, d, *J* = 4.72 Hz), 5.17 (1H, d, *J* = 4.84 Hz), 5.28 (1H, d, *J* = 4.20 Hz), 5.48 (1H, s), 6.51 (1H, d, *J* = 1.16 Hz), 7.68 (2H, q, *J* = 3.56 Hz).[2-3]

Methyl 2-((4R,4aR,7R,10R)-4-(furan-3-yl)-10-hydroxy-4a,7,9,9-tetramethyl-2,13-dioxo-1,4,4a,5,6,6a,7,8,9,10,11,12-dodecahydro-2H-7,11-methanocycloocta[f]isochromen-8-yl)-2-hydroxyacetate (6)

^{1}H-NMR (DMSO) 0.84 (3H, s), 0.91 (3H, s), 1.01 (3H, s), 1.30 (3H, s), 1.49 (2H, m, *J* = 7.42 Hz), 1.64 (3H, d, *J* = 7.03 Hz), 1.70 (4H, s), 2.01 (1H, q, *J* = 5.96 Hz), 2.23 (1H, q, *J* = 5.57 Hz), 2.32 (1H, d, *J* = 5.33 Hz), 2.65 (1H, d, *J* = 18.75 Hz), 2.85 (1H, q, *J* = 8.39 Hz),

3.28 (1H, d, J = 8.36 Hz), 3.41 (1H, s), 3.61 (3H, s), 4.49 (1H, d, J = 4.90 Hz), 4.53 (1H, d, J = 9.42 Hz), 5.17 (1H, d, J = 7.23 Hz), 5.45 (1H, s), 5.57 (1H, d, J = 4.67 Hz), 6.52 (1H, d, J = 1.16 Hz), 6.79 (1H, m, J = 3.97 Hz), 7.71 (1H, t, J = 1.60 Hz), 7.77 (1H, s). [4-5]

(4R,4aR,7R,10R)-8-(1-acetoxy-2-methoxy-2-oxoethyl)-4-(furan-3-yl)-4a,7,9,9-tetramethyl-2,13-dioxo-1,4,4a,5,6,6a,7,8,9,10,11,12b-dodecahydro-2H-7,11-methanocycloocta[f]isochromen-10-yl (E)-2-methylbut-2-enoate (7)

^{1}H-NMR (DMSO) 0.87 (3H, s), 0.91 (3H, s), 1.04 (3H, s), 1.08 (3H, s), 1.67 (9H, t, J = 14.02 Hz), 2.02 (1H, s), 2.18 (3H, s), 2.32 (2H, m, J = 7.16 Hz), 2.66 (1H, d, J = 18.66 Hz), 2.86 (1H, q, J = 8.37 Hz), 3.37 (1H, d, J = 8.14 Hz), 3.61 (4H, d, J = 22.39 Hz), 4.64 (1H, d, J = 9.42 Hz), 5.20 (1H, d, J = 7.22 Hz), 5.43 (1H, s), 5.50 (1H, s), 6.55 (1H, s), 6.78 (1H, d, J = 7.06 Hz), 7.71 (1H, s), 7.77 (1H, s). [5-7]

(4R,4aR,7R,10R)-4-(furan-3-yl)-8-(1-hydroxy-2-methoxy-2-oxoethyl)-4a,7,9,9-tetramethyl-2,13-dioxo-1,4,4a,5,6,6a,7,8,9,10,11,12-dodecahydro-2H-7,11-methanocycloocta[f]isochromen-10-yl (E)-2-methylbut-2-enoate (8)

^{1}H-NMR (DMSO) 0.79 (3H, s), 0.90 (3H, s), 0.98 (3H, s), 1.05 (1H, t, J = 8.89 Hz), 1.28 (3H, s), 1.70 (5H, q, J = 11.14 Hz), 1.78 (3H, s), 1.83 (1H, d, J = 10.34 Hz), 2.02 (2H, t, J = 7.28 Hz), 2.60 (1H, d, J = 14.71 Hz), 2.99 (1H, q, J = 4.52 Hz), 3.25 (1H, s), 3.35 (2H, s), 3.69 (1H, s), 4.47 (3H, d, J = 4.53 Hz), 4.60 (1H, d, J = 9.66 Hz), 5.40 (1H, s), 5.51 (1H, d, J = 4.48 Hz), 6.52 (1H, s), 6.84 (1H, d, J = 7.07 Hz), 7.71 (2H, s). [4, 5, 8]

Methyl 2-acetoxy-2-((4R,4aR,7R,10R)-10-acetoxy-4-(furan-3-yl)-4a,7,9,9-tetramethyl-2,13-dioxo-1,4,4a,5,6,6a,7,8,9,10,11,12-dodecahydro-2H-7,11-methanocycloocta[f]isochromen-8-yl) acetate (9)

^{1}H-NMR (DMSO) 0.77 (3H, s), 0.95 (3H, s), 0.99 (3H, s), 1.05 (3H, s), 1.12 (1H, d, J = 9.03 Hz), 1.73 (3H, q, J = 10.37 Hz), 2.10 (5H, s), 2.16 (3H, s), 2.97 (2H, m, J = 8.36 Hz), 3.30 (1H, s), 3.51 (1H, d, J = 20.28 Hz), 3.73 (4H, s), 4.81 (1H, d, J = 9.89 Hz), 5.38 (1H, s), 5.51 (1H, s), 6.53 (1H, d, J = 1.08 Hz), 7.71 (1H, t, J = 1.64 Hz), 7.75 (1H, s). [5, 9]

(4R,4aR,7R,10R)-8-(1-acetoxy-2-methoxy-2-oxoethyl)-4-(furan-3-yl)-4a,7,9,9-tetramethyl-2,13-dioxo-1,4,4a,5,6,6a,7,8,9,10,11,12-dodecahydro-2H-7,11-methanocycloocta[f]isochromen-10-yl (E)-2-methylbut-2-enoate (10)

^{1}H-NMR (DMSO) 0.83 (3H, s), 0.92 (3H, s), 1.01 (3H, s), 1.07 (3H, s), 1.12 (1H, q, *J* = 5.09 Hz), 1.75 (8H, q, *J* = 9.10 Hz), 2.05 (2H, d, *J* = 16.28 Hz), 2.17 (3H, s), 2.66 (1H, q, *J* = 2.35 Hz), 3.06 (1H, t, *J* = 4.00 Hz), 3.36 (2H, s), 3.45 (3H, s), 3.73 (1H, s), 4.71 (1H, d, *J* = 9.74 Hz), 5.36 (1H, s), 5.41 (1H, s), 6.54 (1H, s), 6.83 (1H, q, *J* = 6.75 Hz), 7.72 (1H, d, *J* = 1.52 Hz), 7.77 (1H, s). [5, 8, 10]

(4R,4aR,7R,10R)-4-(furan-3-yl)-8-(2-methoxy-2-oxoethyl)-4a,7,9,9-tetramethyl-2,13-dioxo-1,4,4a,5,6,6a,7,8,9,10,11,12-dodecahydro-2H-7,11-methanocycloocta[f]isochromen-10-yl (E)-2-methylbut-2-enoate (11)

^{1}H-NMR (400 MHz, DMSO) δ 0.68 (s, 3H), 0.74 (s, 3H), 0.85 (q, *J* = 3.5 Hz, 2H), 0.94 (s, 3H), 0.99 (m, *J* = 4.0 Hz, 1H), 1.07 (s, 3H), 1.47 (m, 1H), 1.64 (d, *J* = 10.0 Hz, 1H), 1.74 (d, *J* = 7.0 Hz, 3H), 1.81 (s, 1H), 1.99 (m, *J* = 9.7 Hz, 1H), 2.18 (m, *J* = 3.6 Hz, 2H), 2.39 (t, *J* = 8.7 Hz, 1H), 2.61 (t, *J* = 8.2 Hz, 2H), 3.03 (q, *J* = 4.6 Hz, 1H), 3.24 (d, *J* = 10.4 Hz, 1H), 3.39 (d, *J* = 15.4 Hz, 2H), 3.66 (s, 3H), 4.76 (d, *J* = 9.6 Hz, 1H), 5.45 (s, 1H), 6.52 (d, *J* = 1.2 Hz, 1H), 6.86 (q, *J* = 2.9 Hz, 1H), 7.67 (s, 1H), 7.71 (t, *J* = 1.6 Hz, 1H). [5, 7, 11]

(4R,4aR,7R,10R)-4-(furan-3-yl)-8-(2-methoxy-2-oxoethyl)-4a,7,9,9-tetramethyl-2,13-dioxo-1,4,4a,5,6,6a,7,8,9,10,11,12b-dodecahydro-2H-7,11-methanocycloocta[f]isochromen-10-yl (E)-2-methylbut-2-enoate (12)

^{1}H-NMR (400 MHz, DMSO) δ 7.84 (s, 1H), 7.71 (t, J = 1.6 Hz, 1H), 6.82 (m, *J* = 4.0 Hz, 1H), 6.55 (d, *J* = 1.2 Hz, 1H), 5.51 (s, 1H), 5.19 (d, *J* = 7.1 Hz, 1H), 4.74 (d, *J* = 9.4 Hz, 1H), 3.65 (s, 3H), 3.35 (s, 1H), 3.32 (s, 1H), 2.86 (t, *J* = 9.9 Hz, 1H), 2.66 (d, *J* = 18.8 Hz, 1H), 2.55 (d, *J* = 7.9 Hz, 1H), 2.40 (q, *J* = 9.3 Hz, 1H), 2.33 (q, *J* = 1.9 Hz, 1H), 2.17 (d, *J* = 4.7 Hz, 1H), 1.89 (m, *J* = 5.4 Hz, 1H), 1.72 (s, 4H), 1.65 (q, *J* = 2.7 Hz, 3H), 1.44 (m, *J* = 5.7 Hz, 2H), 1.06 (s, 3H), 0.99 (s, 3H), 0.77 (s, 3H), 0.70 (s, 3H). [5,7-8.]

Figure S1. SFC screening chromatogram of crude material of *Swietenia macrophylla* seed.

Screening-1 various columns with Isopropyl alcohol as a Co-solvent

Column: Daicel-P4VP

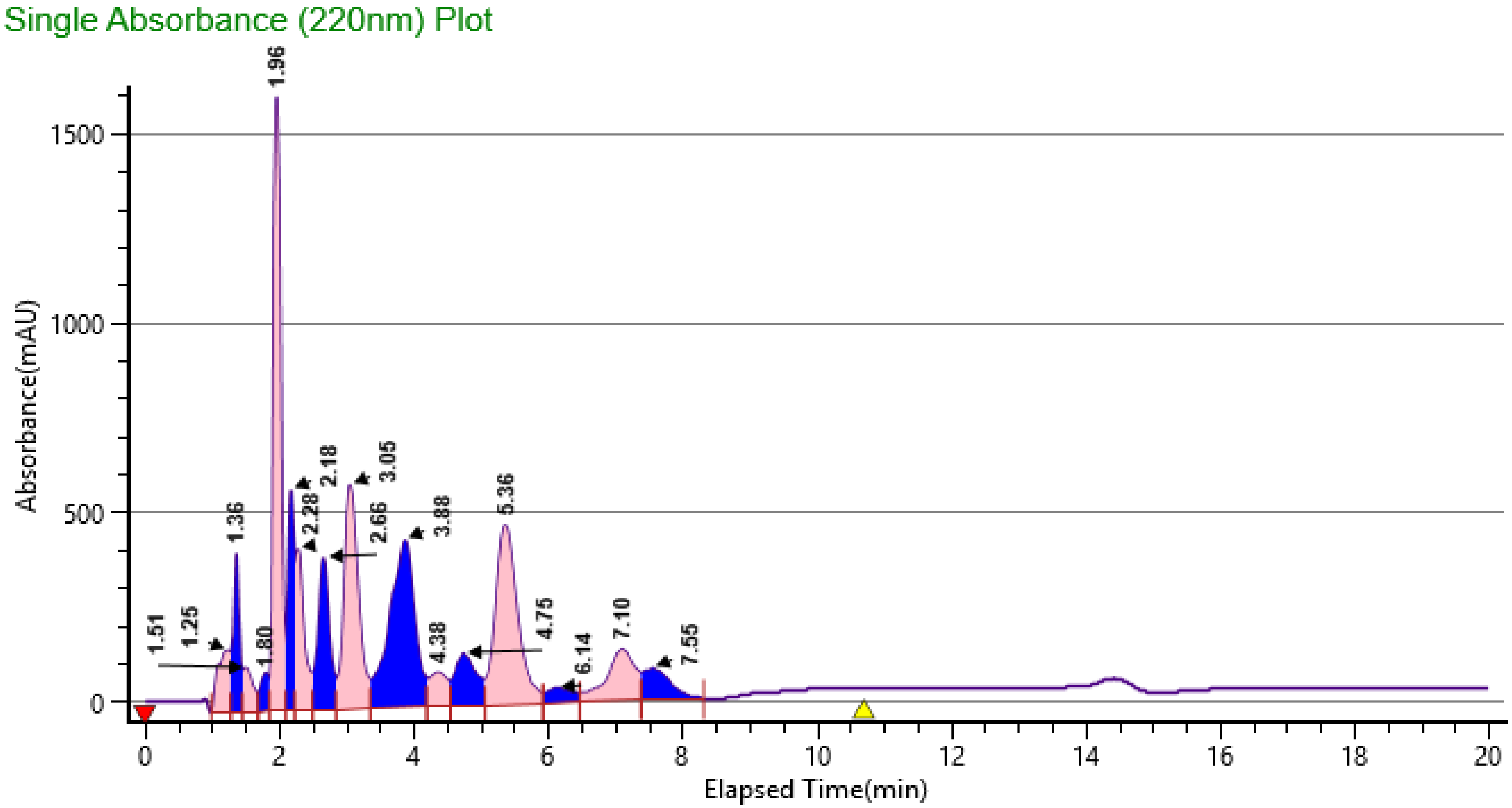

Column: LUX-i-amylose-3

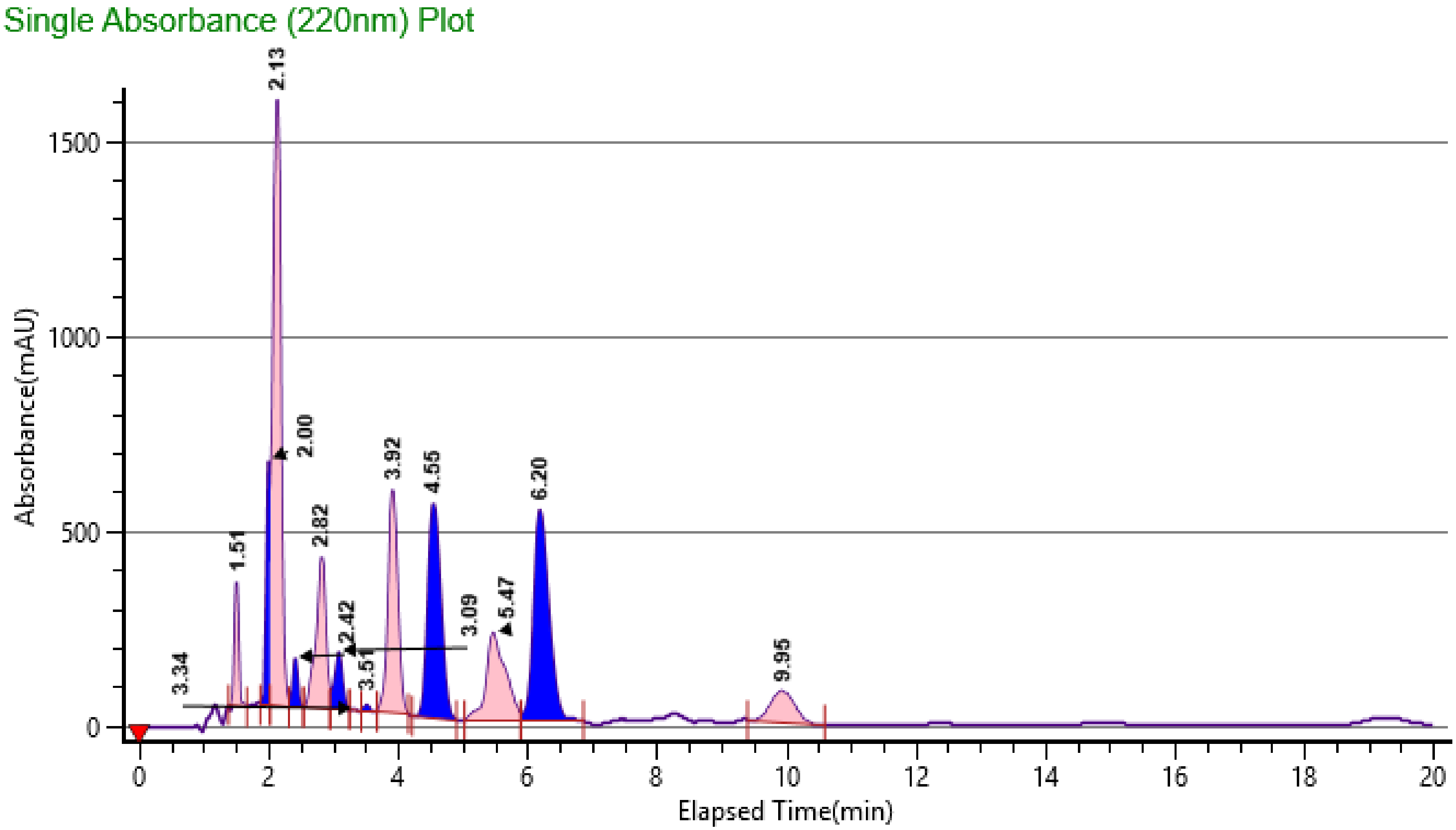

Column: Chiralpak-AD-H

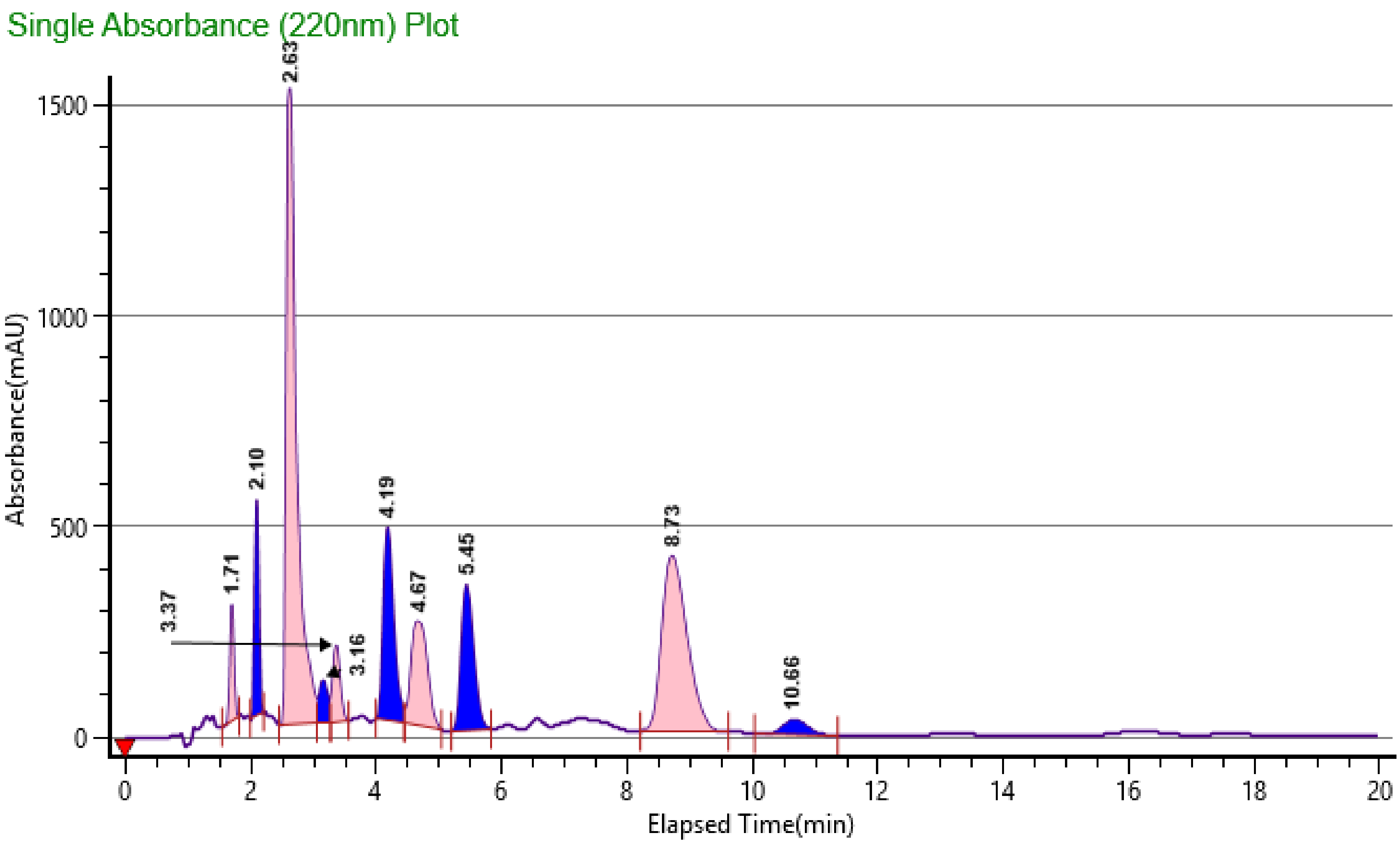
Single Absorbance (220nm) Plot
Absorbance(mAU)
Elapsed Time(min)
1500
1000
500
0
0
2
4
6
8
10
12
14
16
18
20
2.63
2.10
4.19
8.73
5.45
4.67
1.71
3.37
3.16
10.66

Column : LUX-Amylose-2

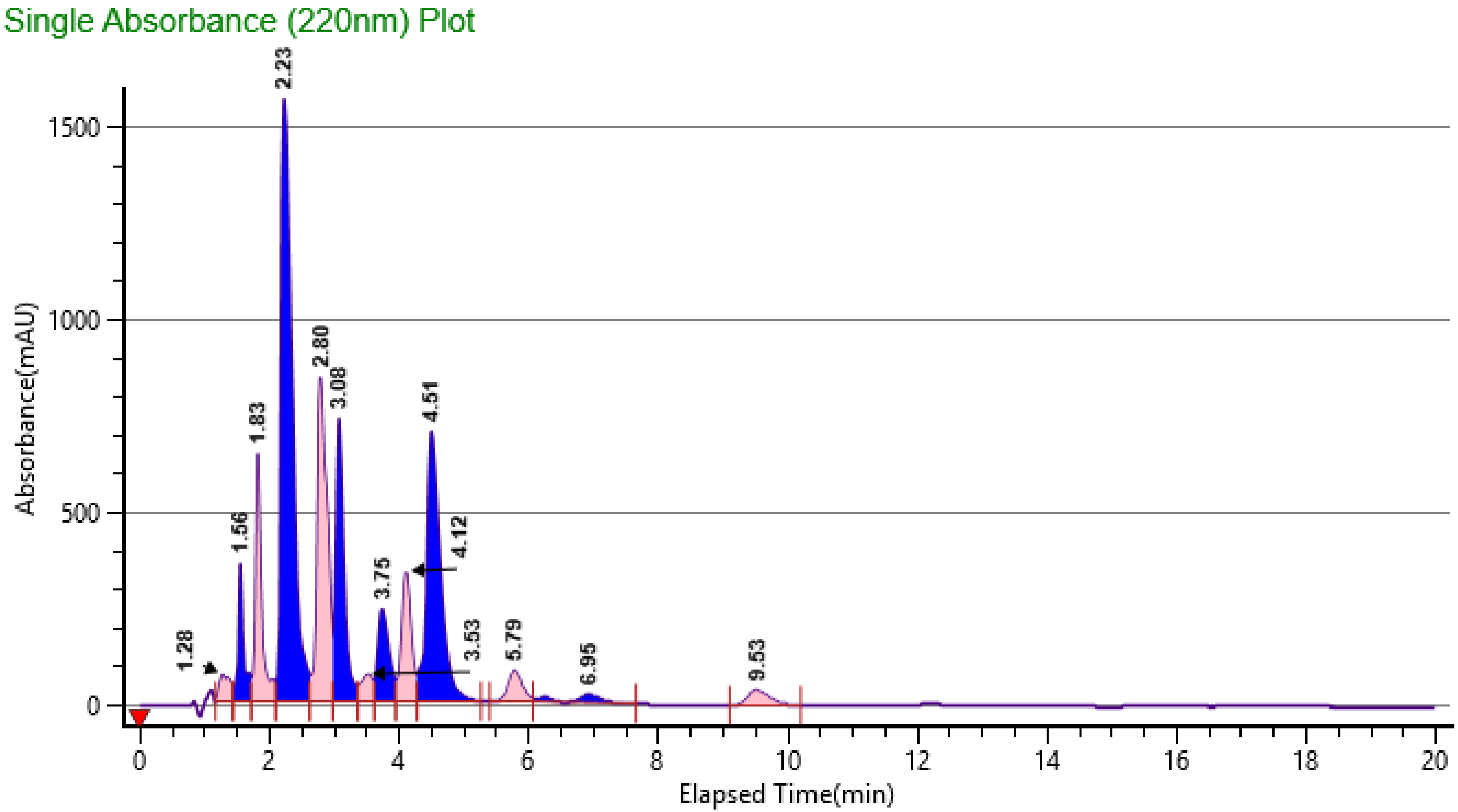

Column : Chiralpak-IG

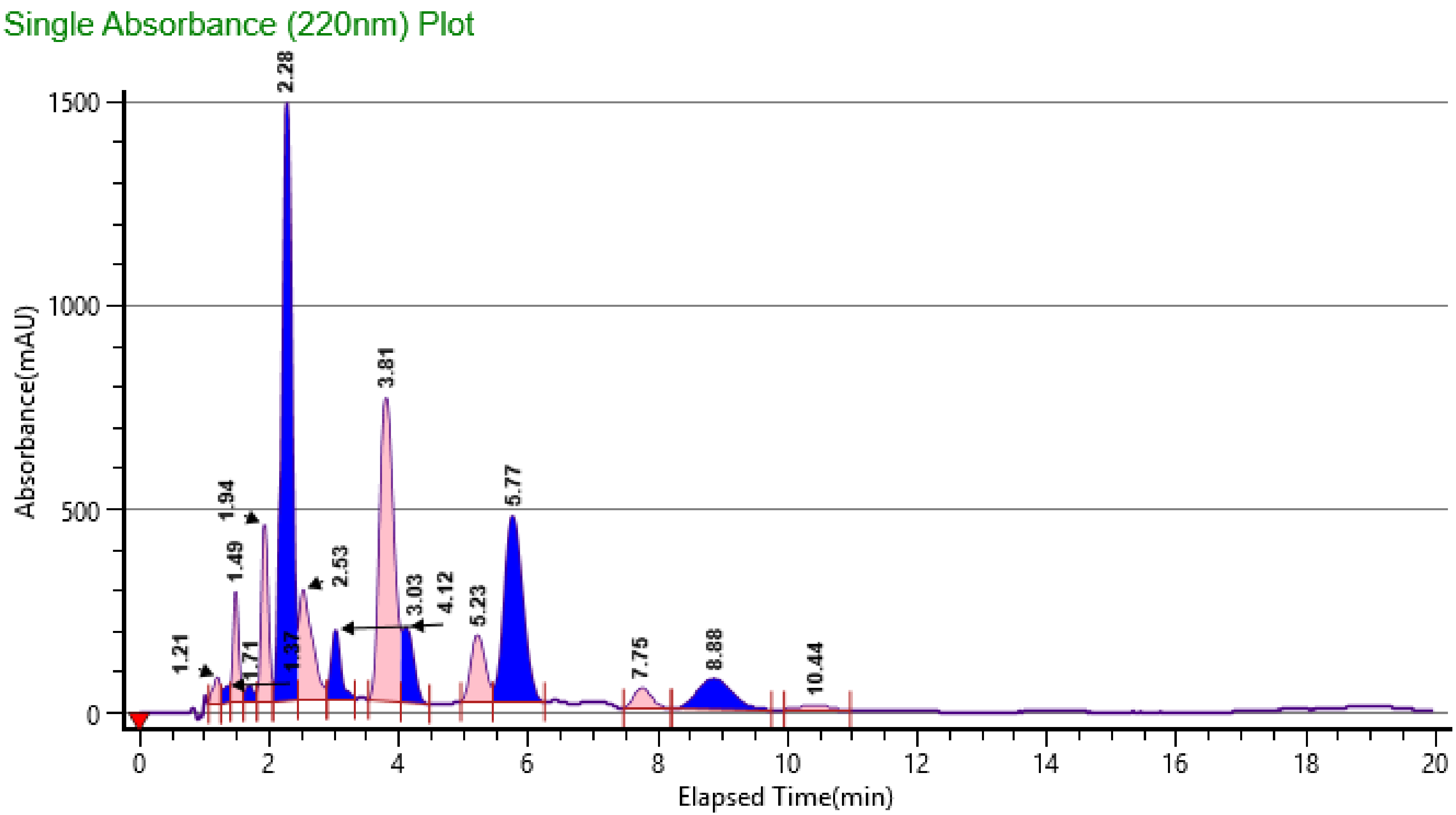
Single Absorbance (220nm) Plot
Absorbance(mAU)
1500
1000
500
0
2.28
3.81
5.77
1.94
1.49
2.53
3.03
4.12
5.23
1.21
1.71
1.37
7.75
8.88
10.44
0
2
4
6
8
10
12
14
16
18
20
Elapsed Time(min)

Column: Chiralpak-AS-H

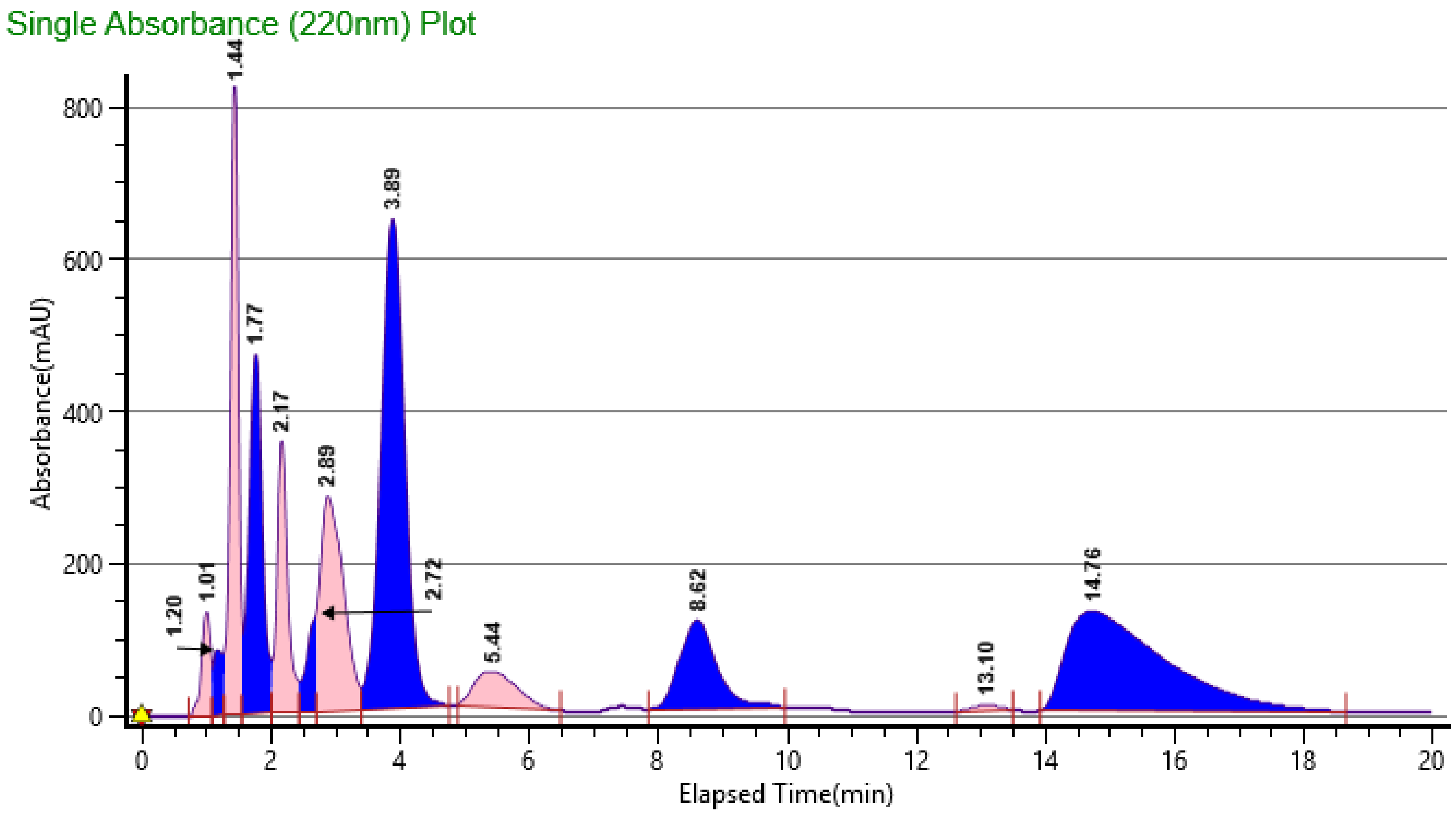

Column: Chiral ART Amylose SA

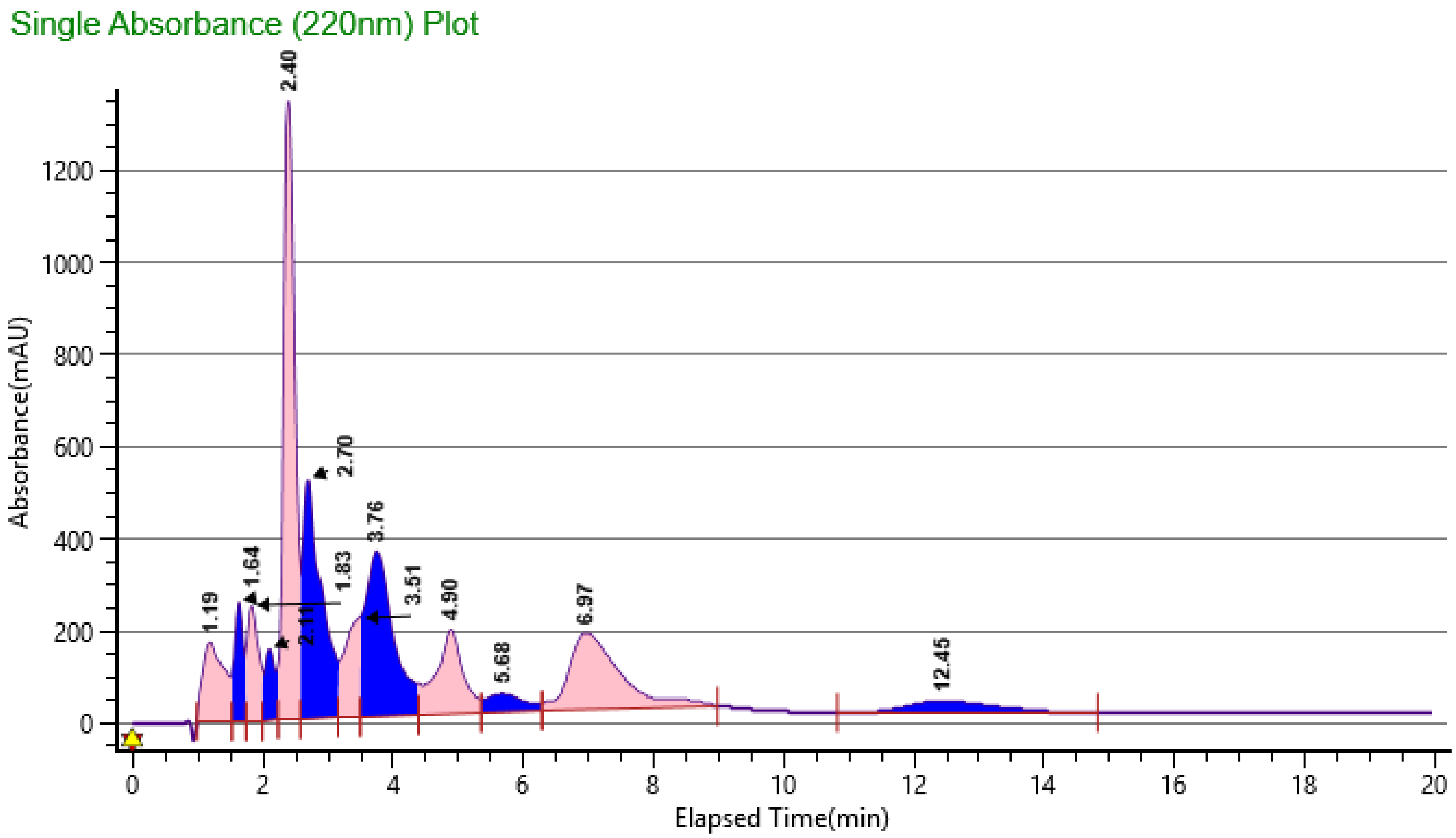

Column : (R,R)Whelk-O1

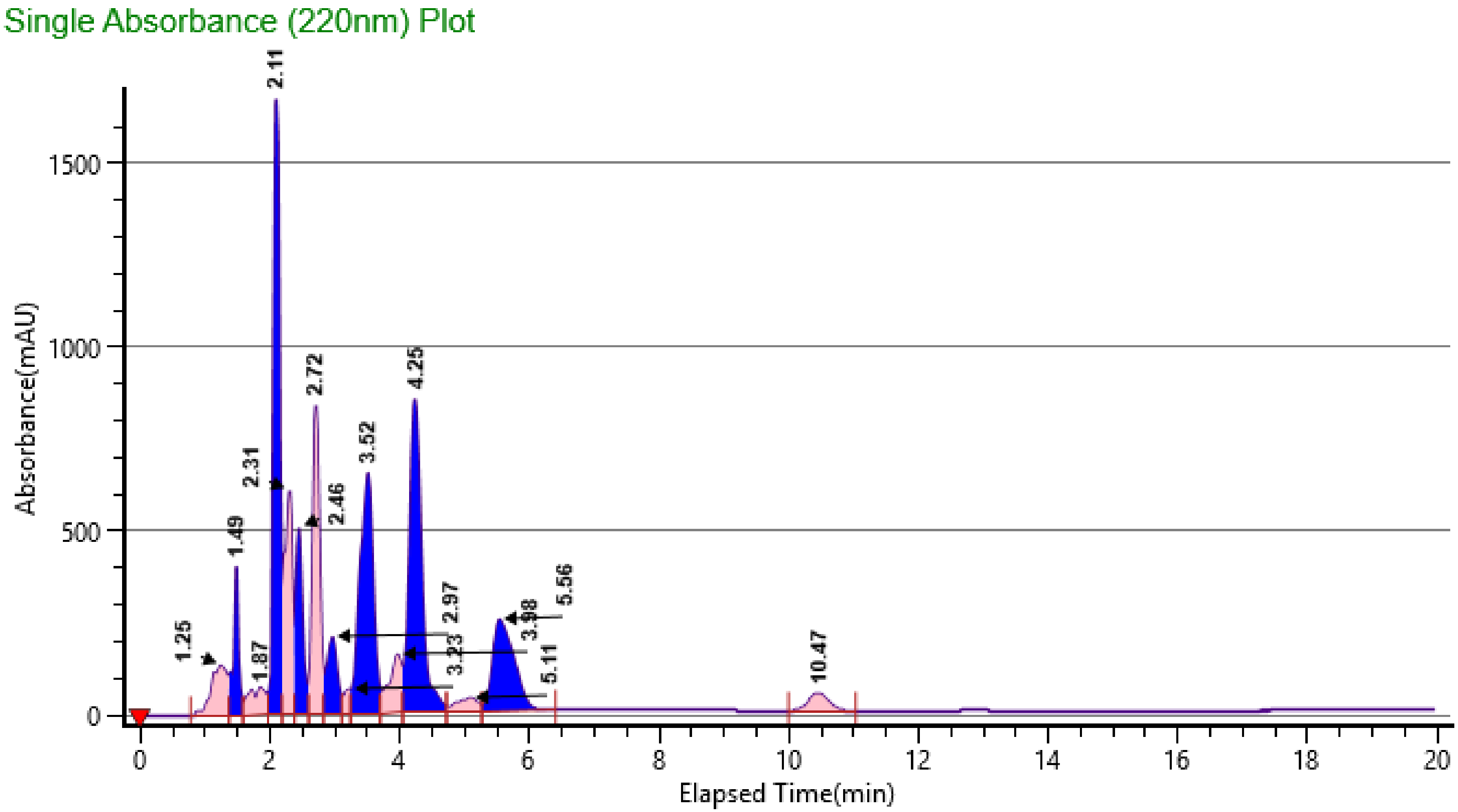

Screening-2 various columns with Ethanol as a Co-solvent

Column: Daicel-P4VP

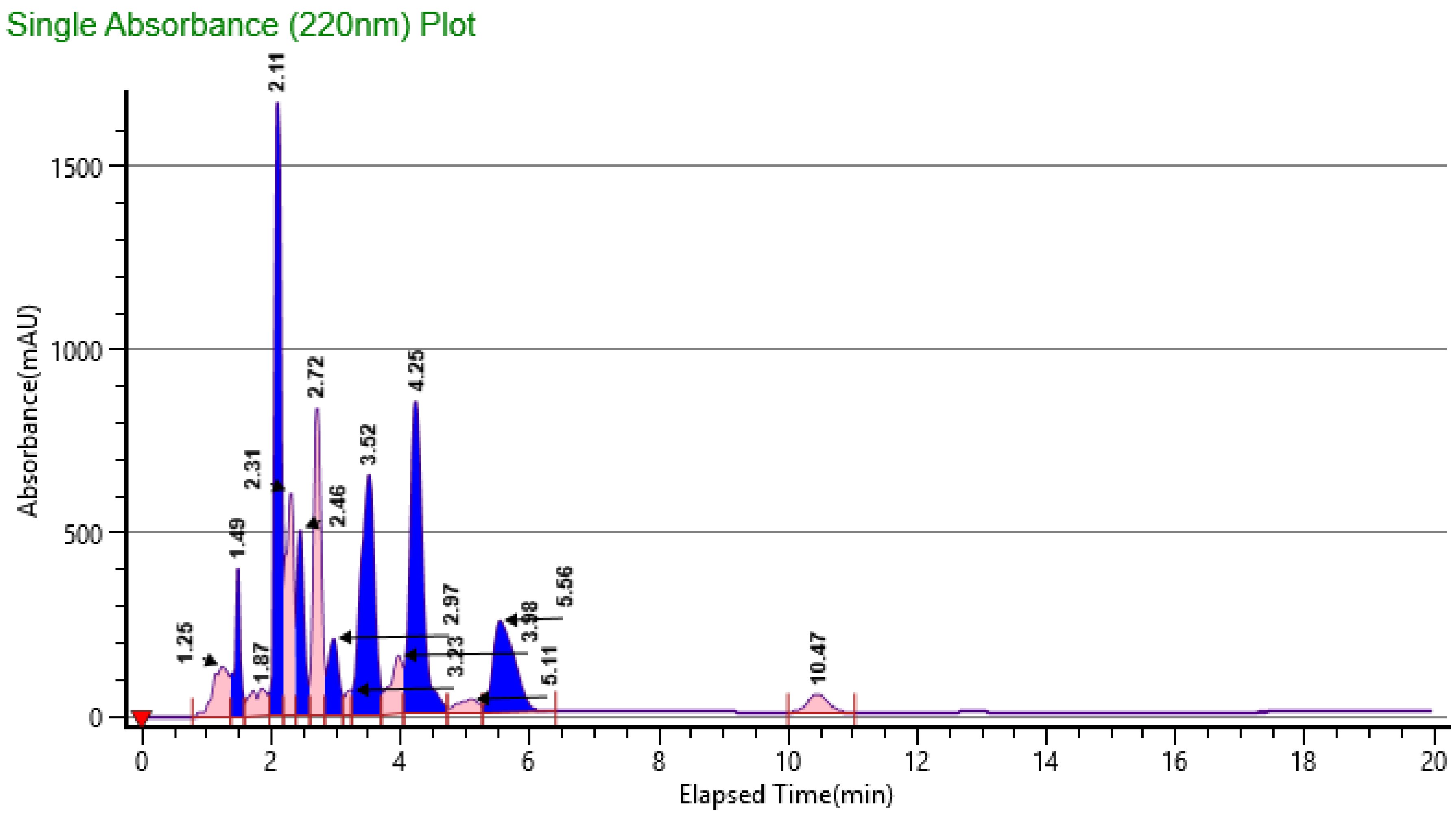

Column: LUX-i-amylose-3

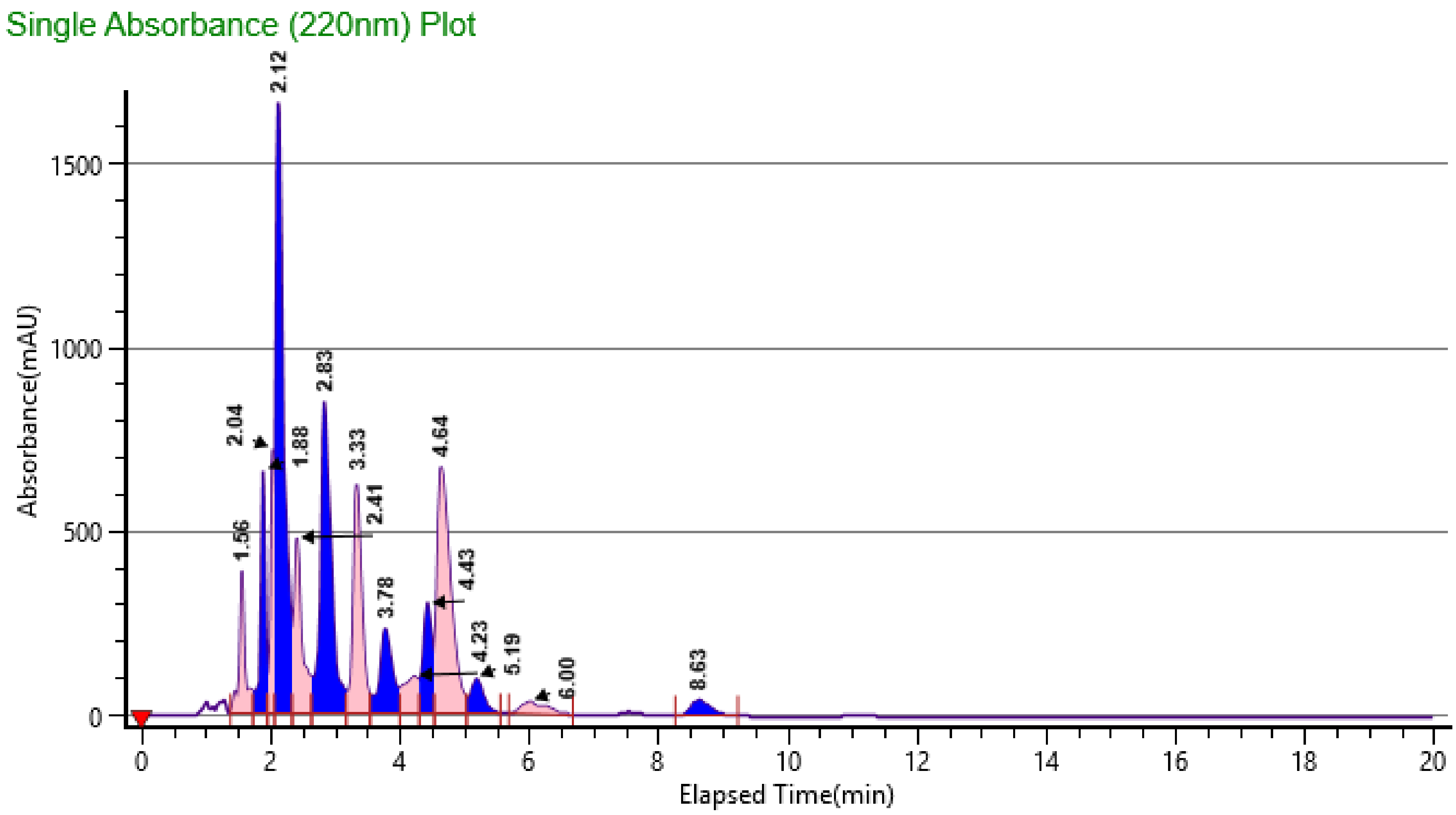

Column: Chiralpak-AD-H

Single Absorbance (220nm) Plot

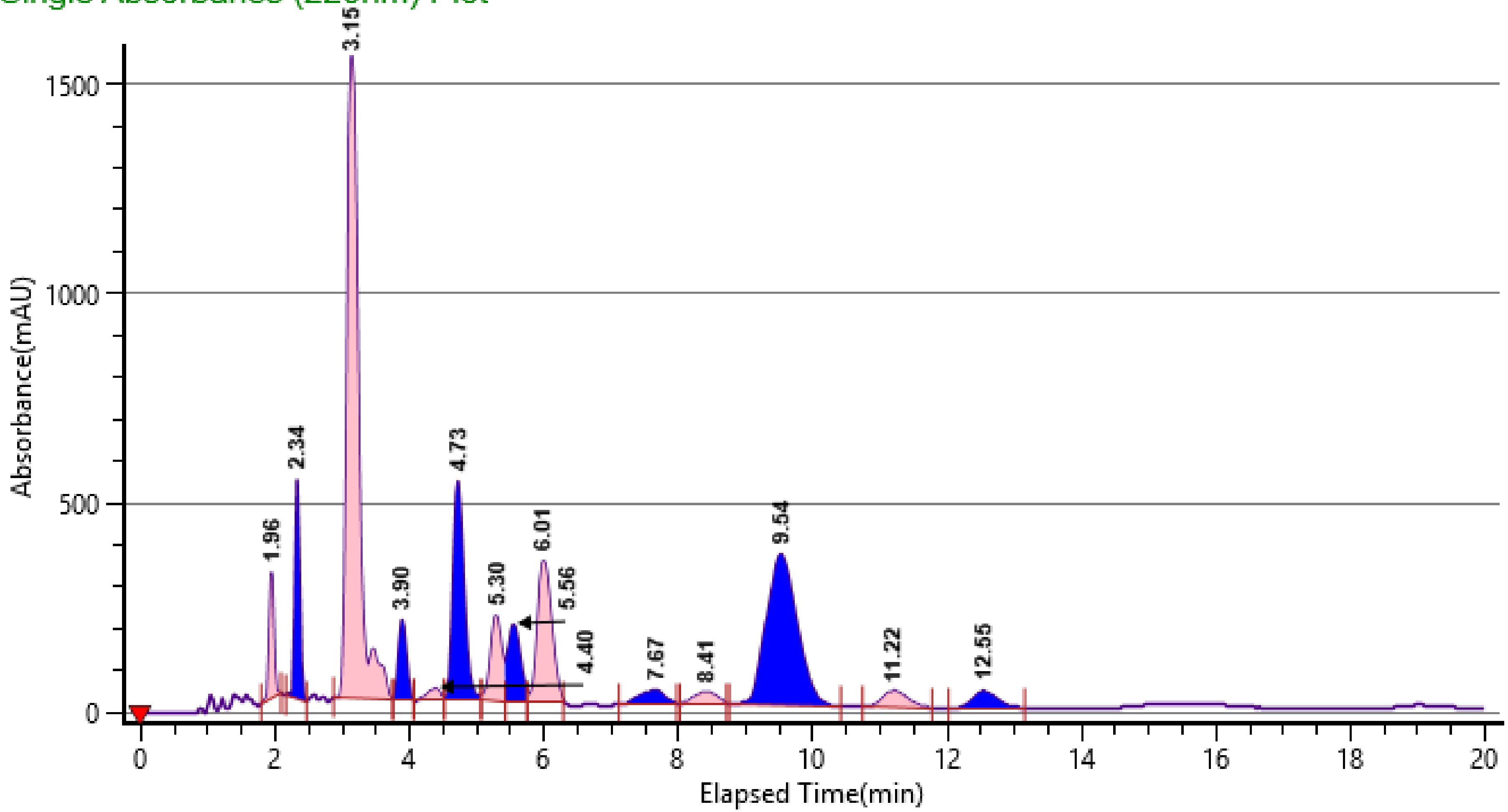

Column: LUX-amylose-2

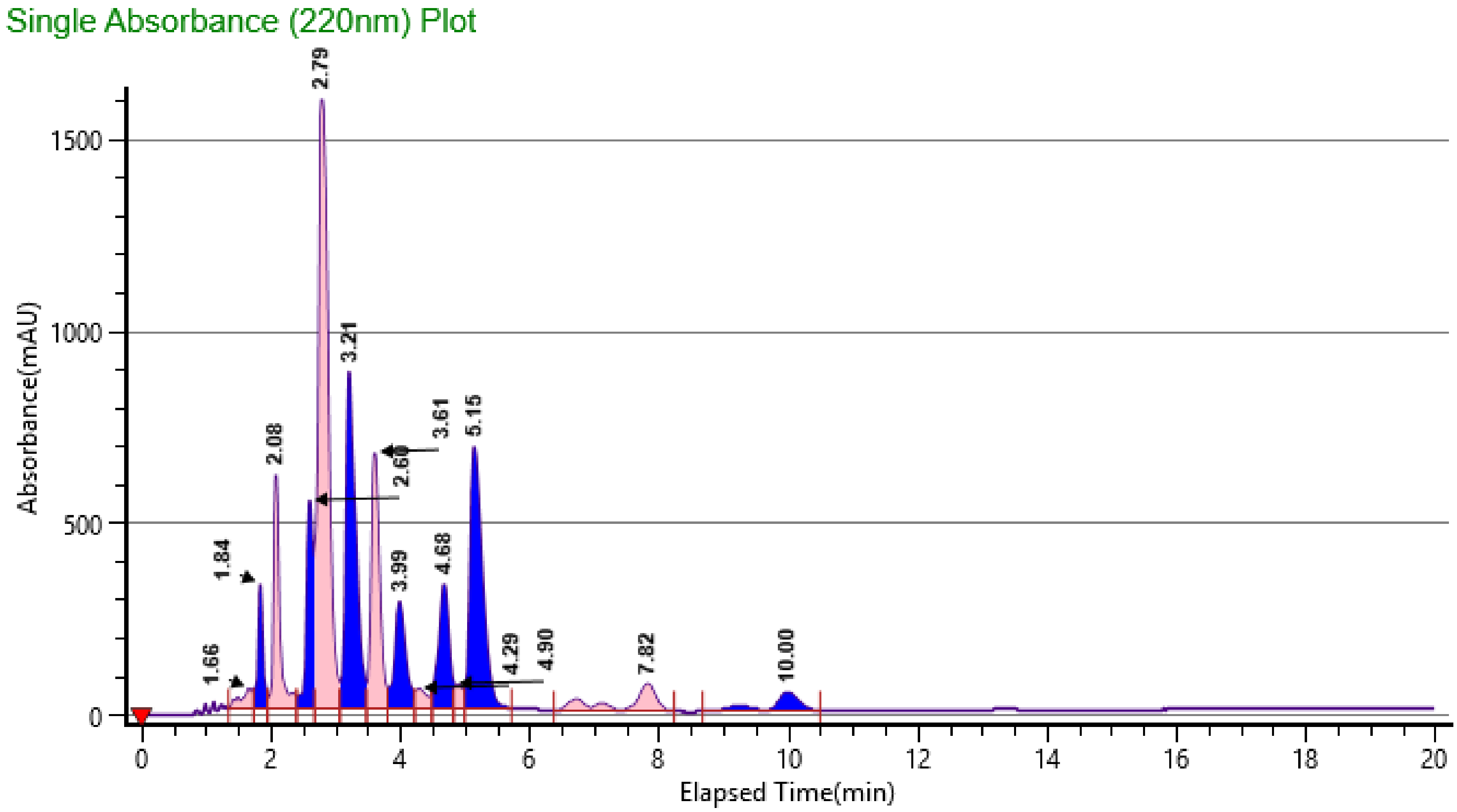

Column: Chiralpak-IG

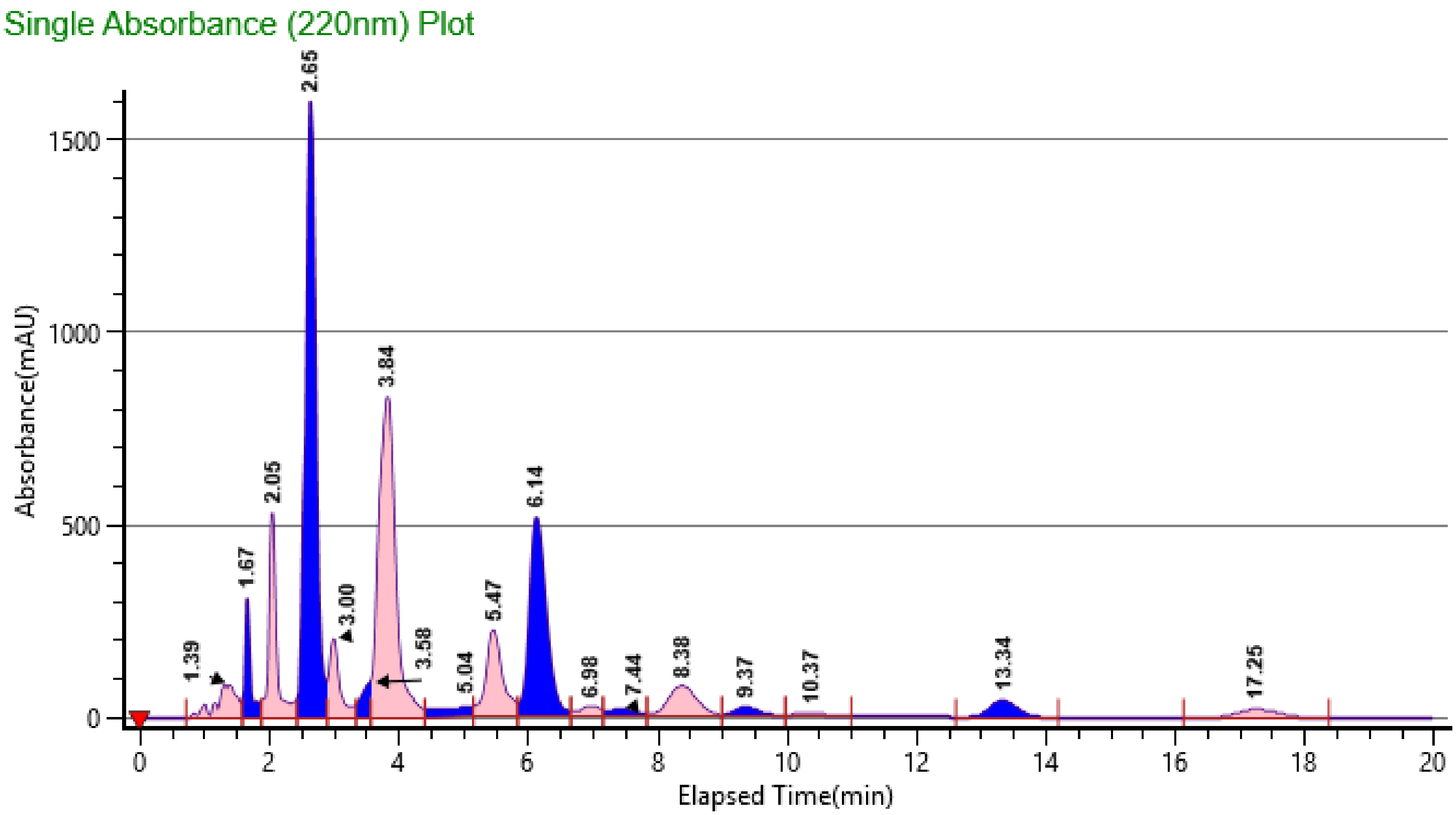

Column: Chiralpak-AS-H

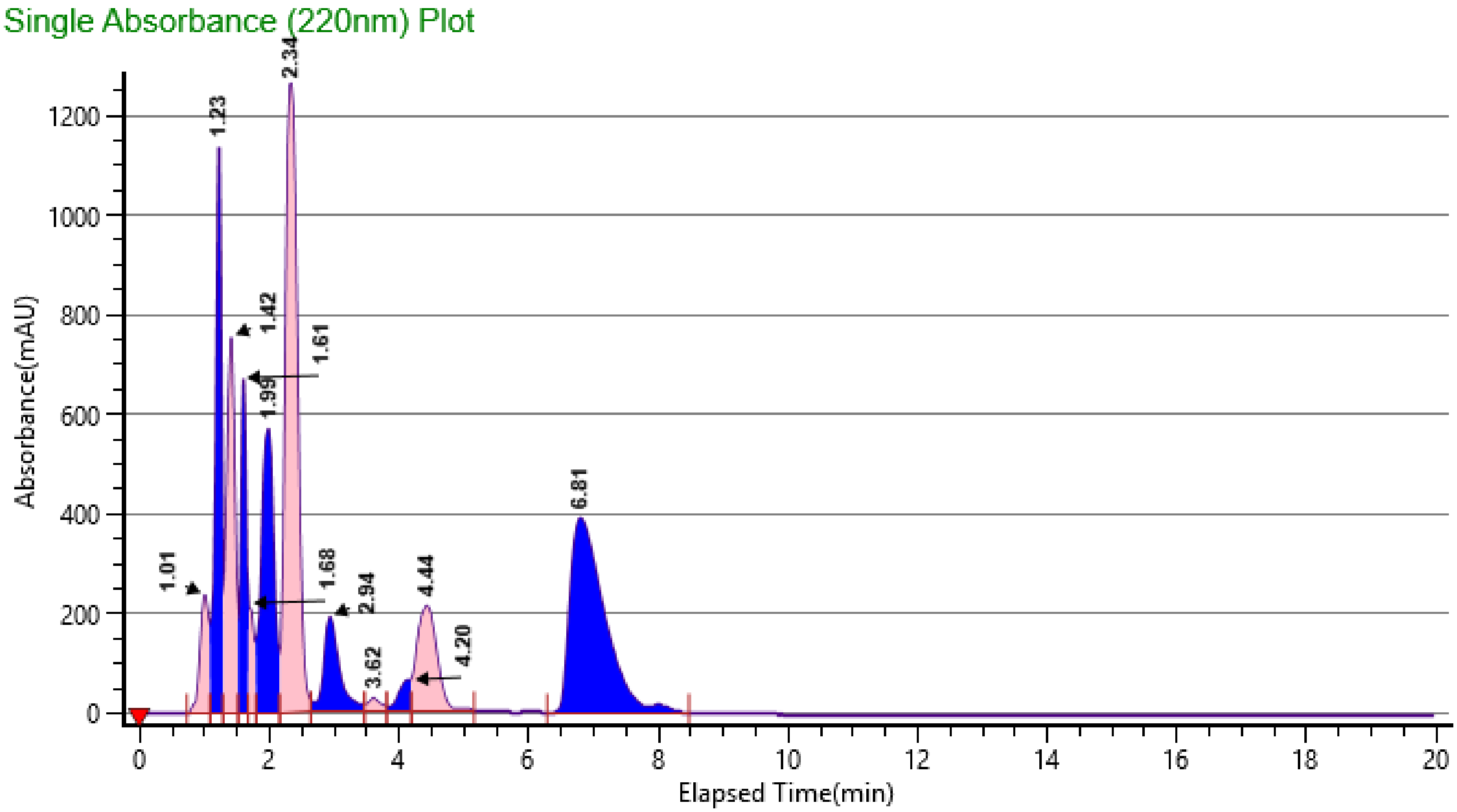

Column: Chiral ART Amylose SA

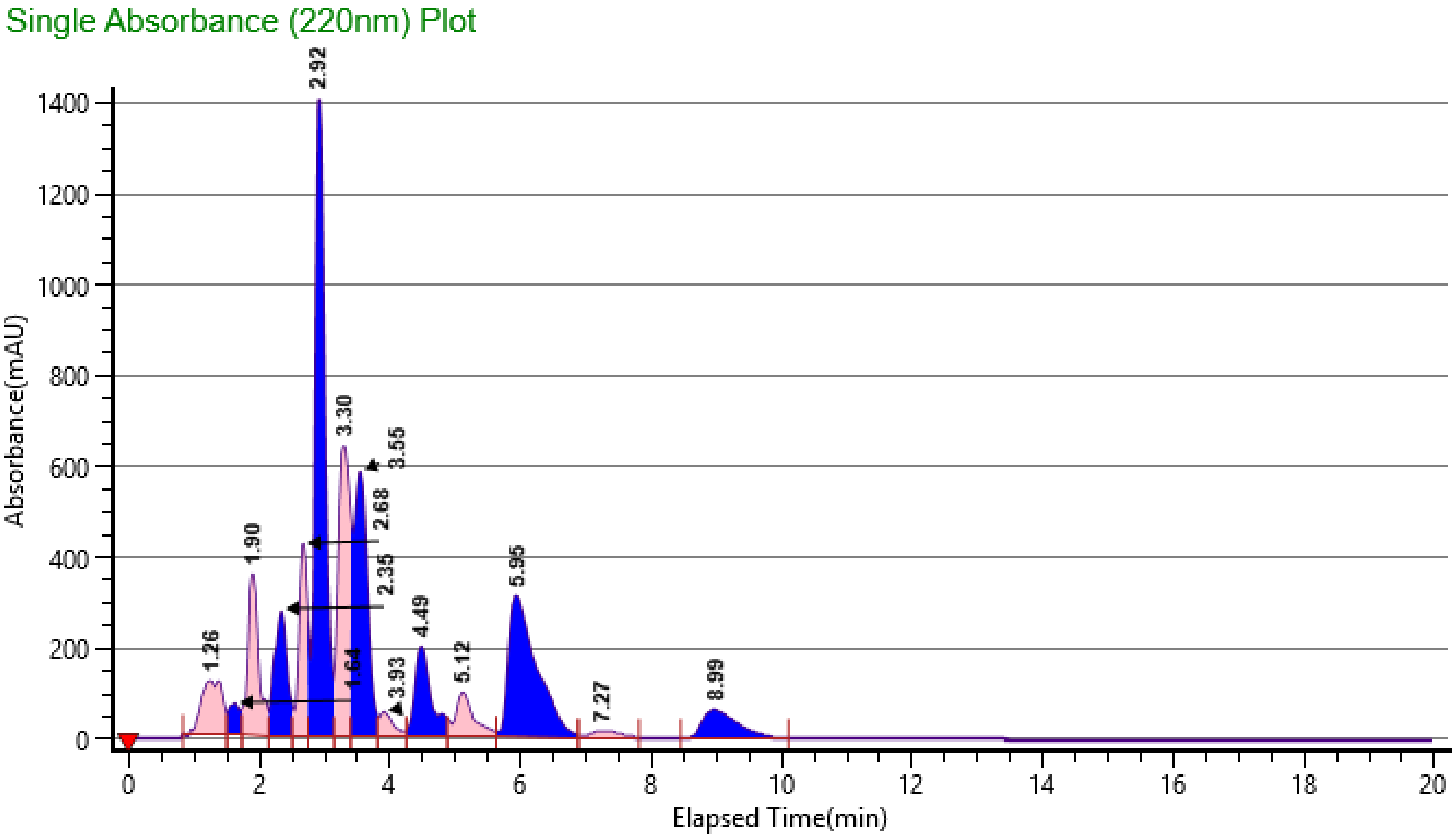

Column: (R,R) Whelk-O1

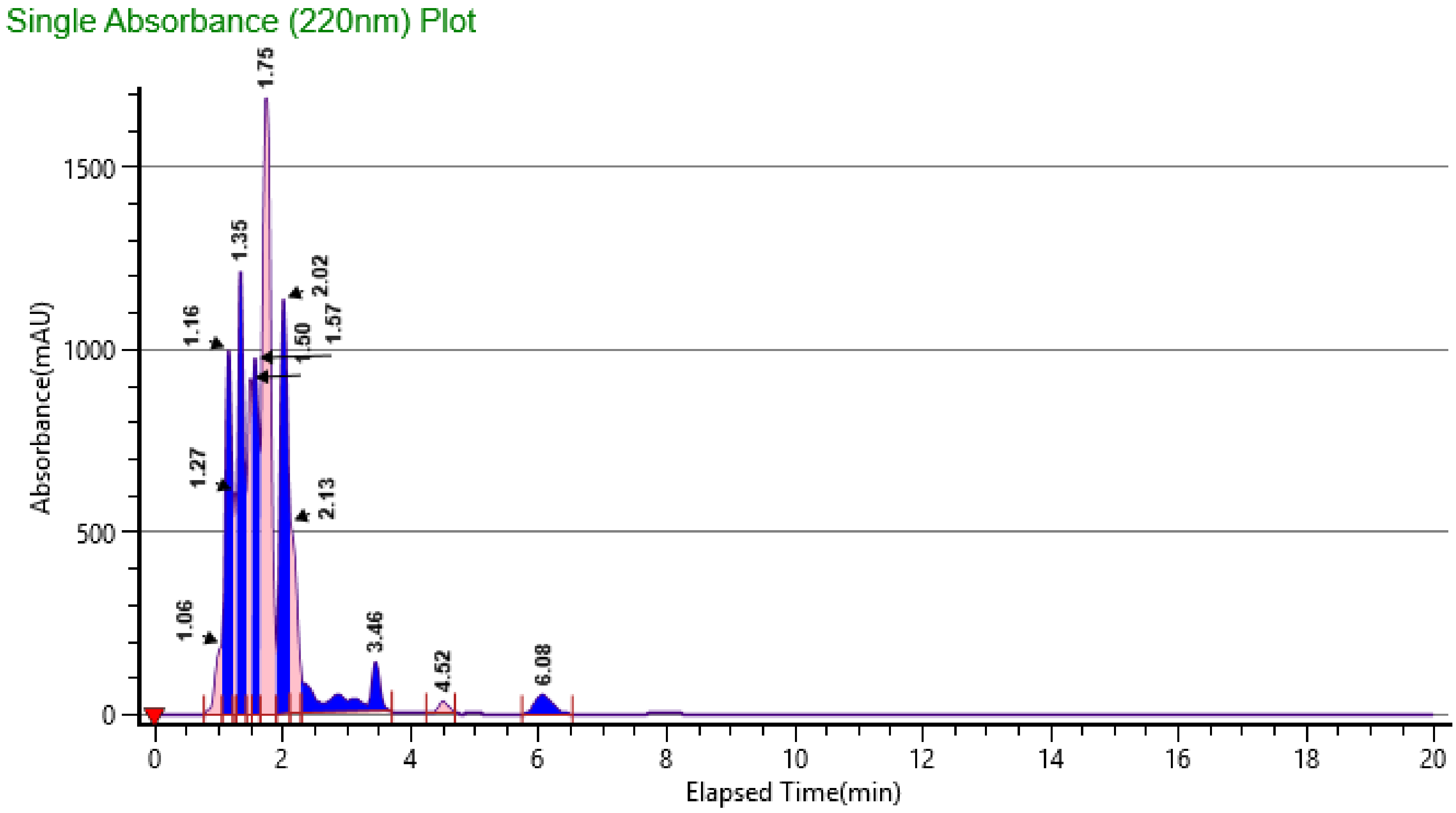

Screening-3 various columns with Methanol as a Co-solvent

Column: Daicel-P4VP

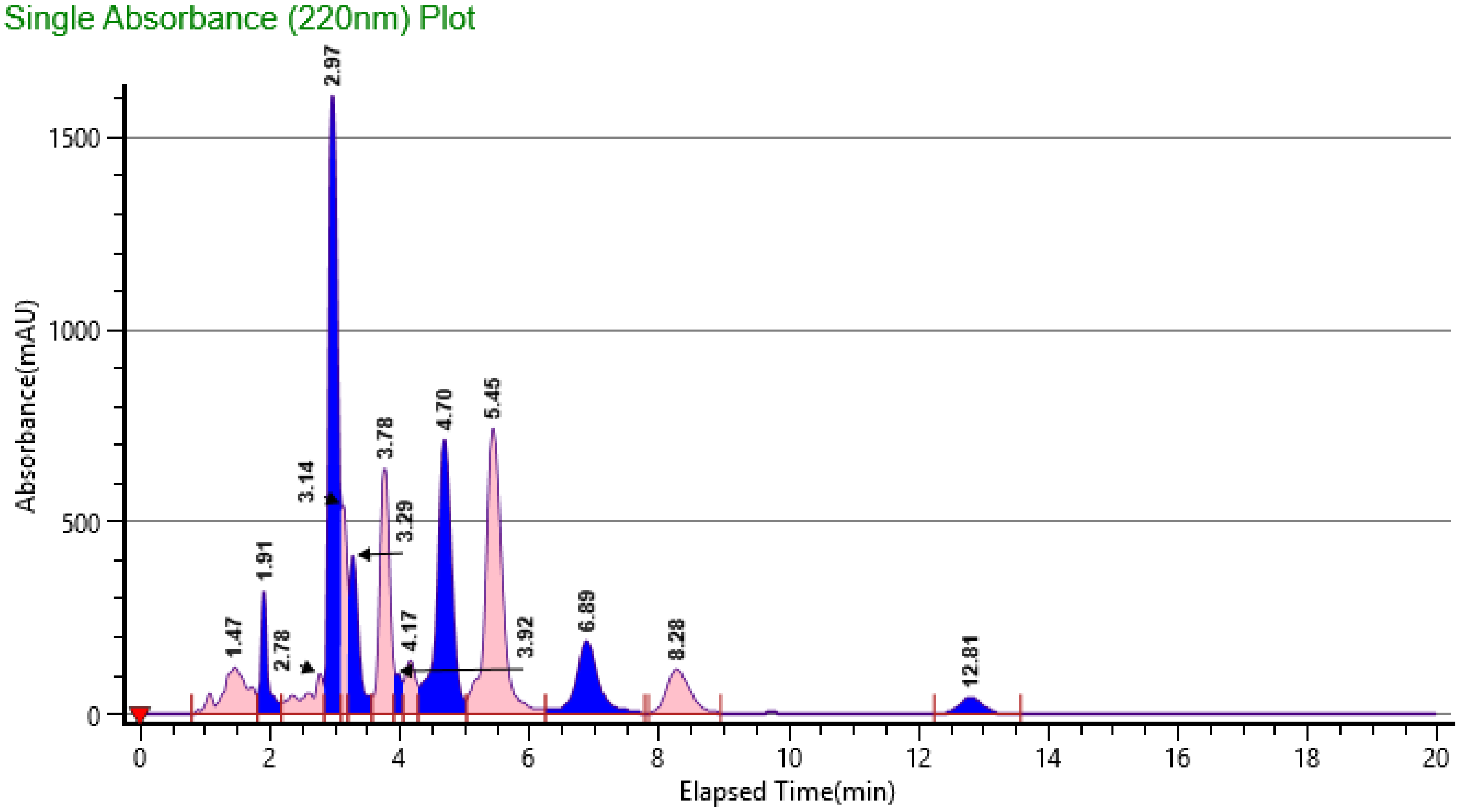

Column: LUX-i-amylose-3

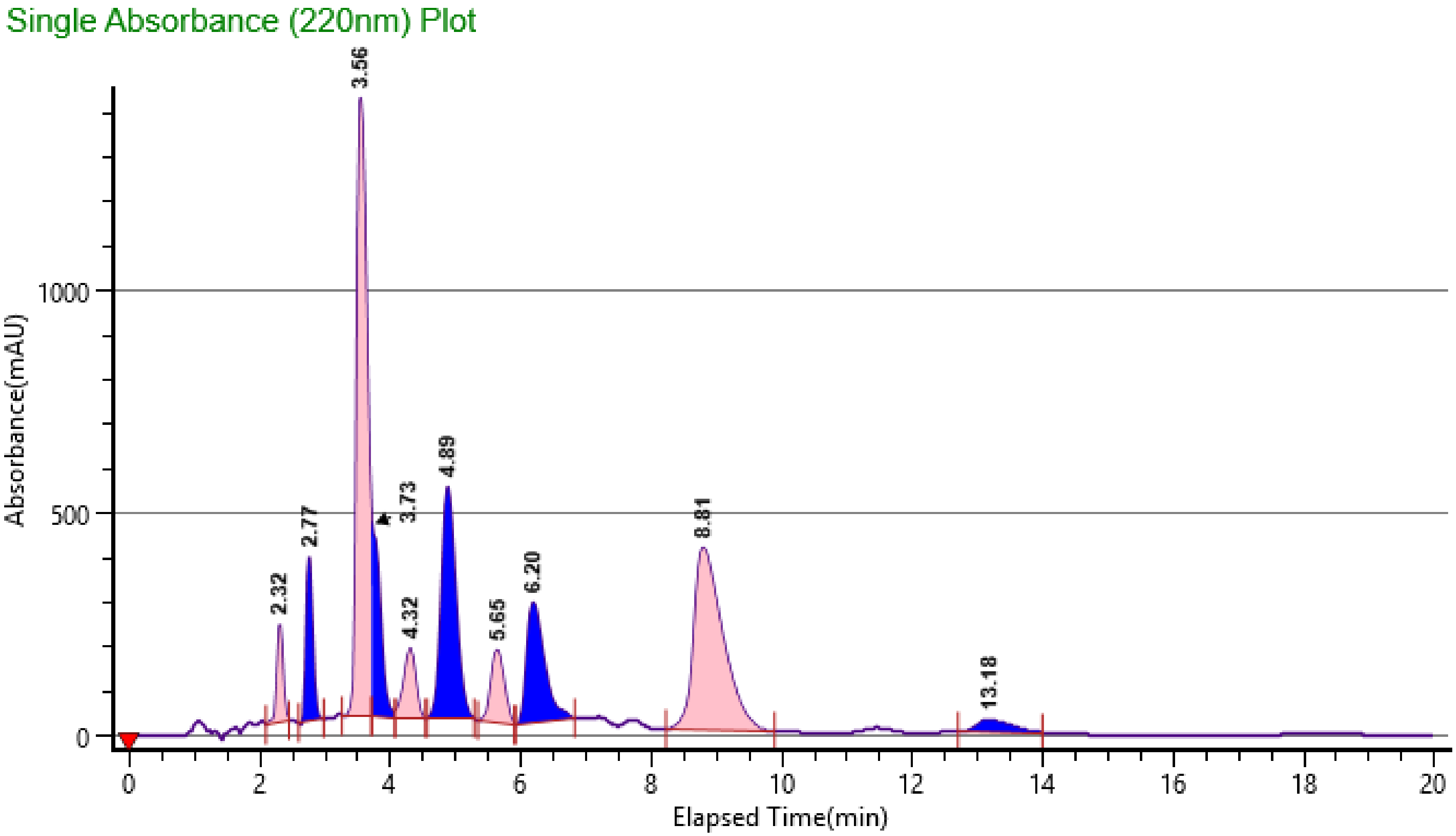

Column: Chiralpak-AD-H

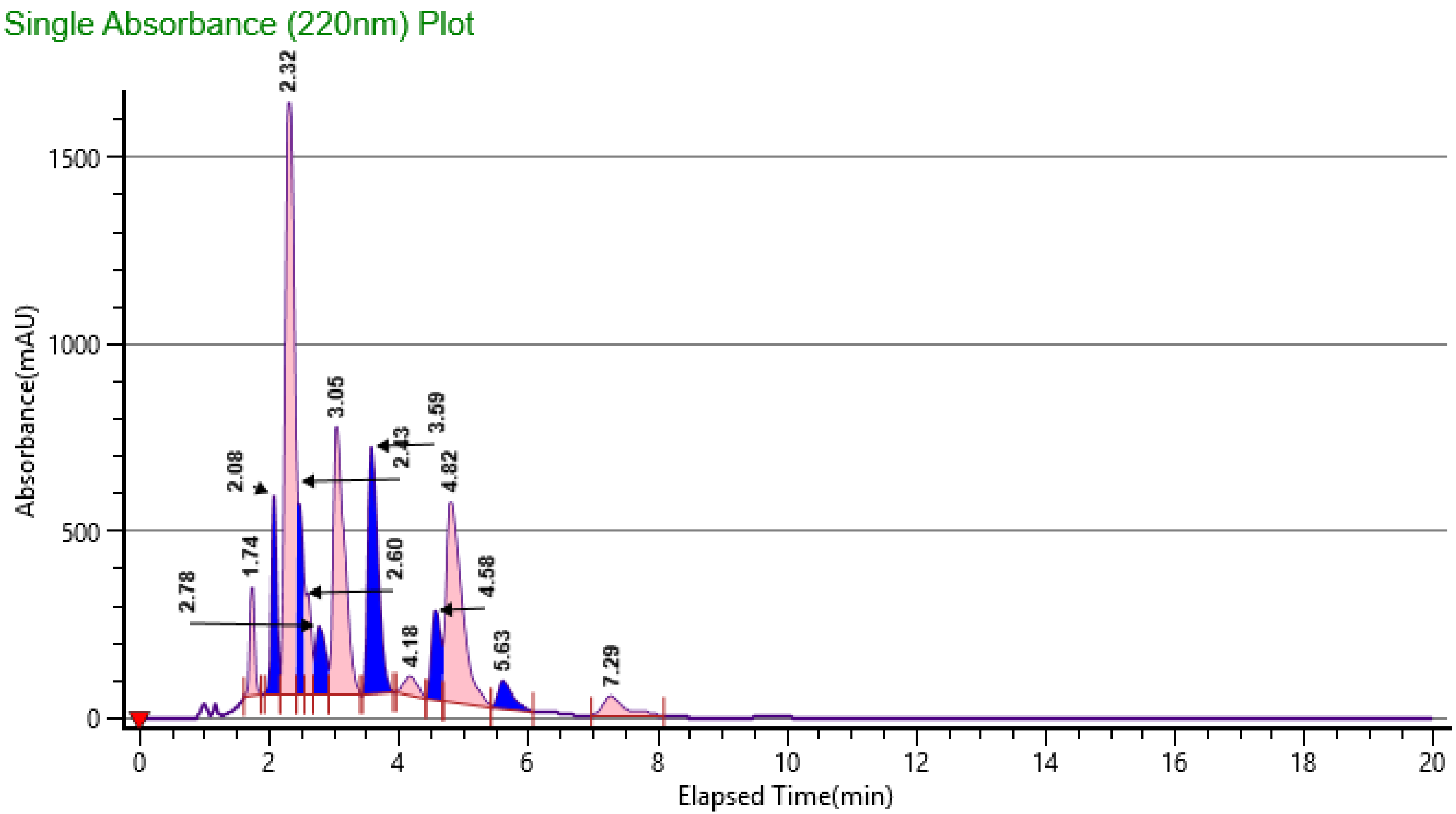

Column: LUX-amylose-2

Single Absorbance (220nm) Plot

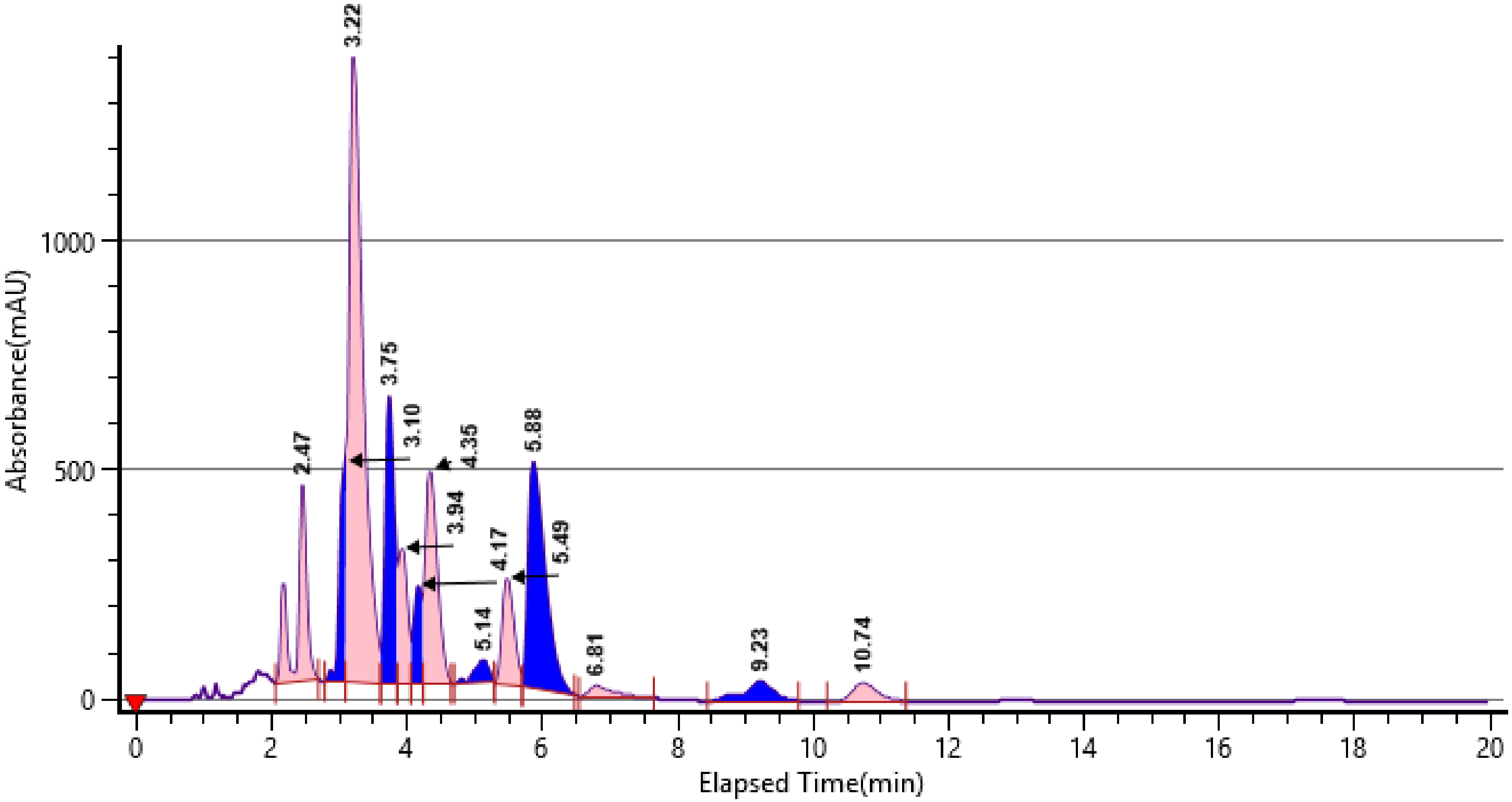

Column: Chiralpak-IG

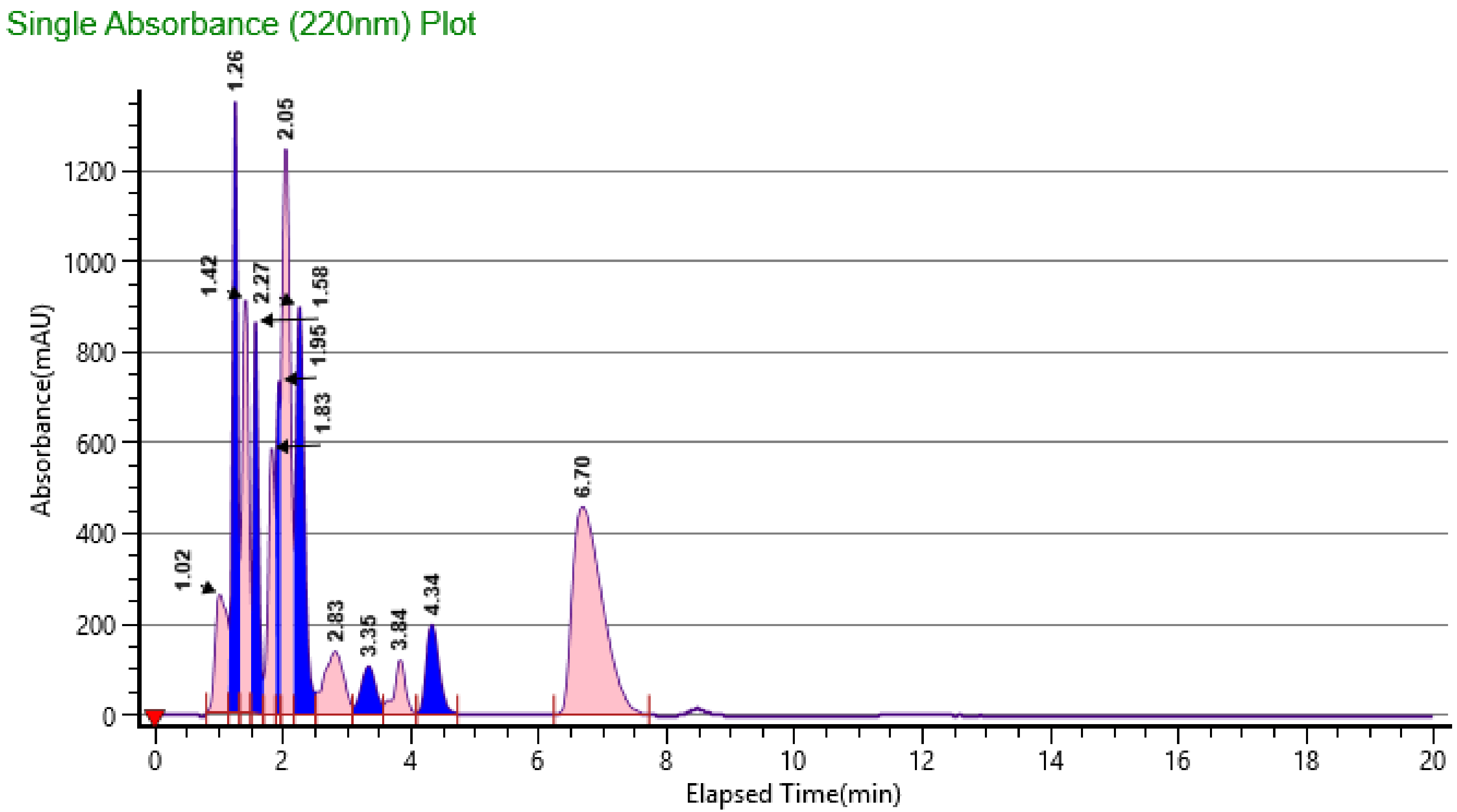

Column: Chiralpak-AS-H

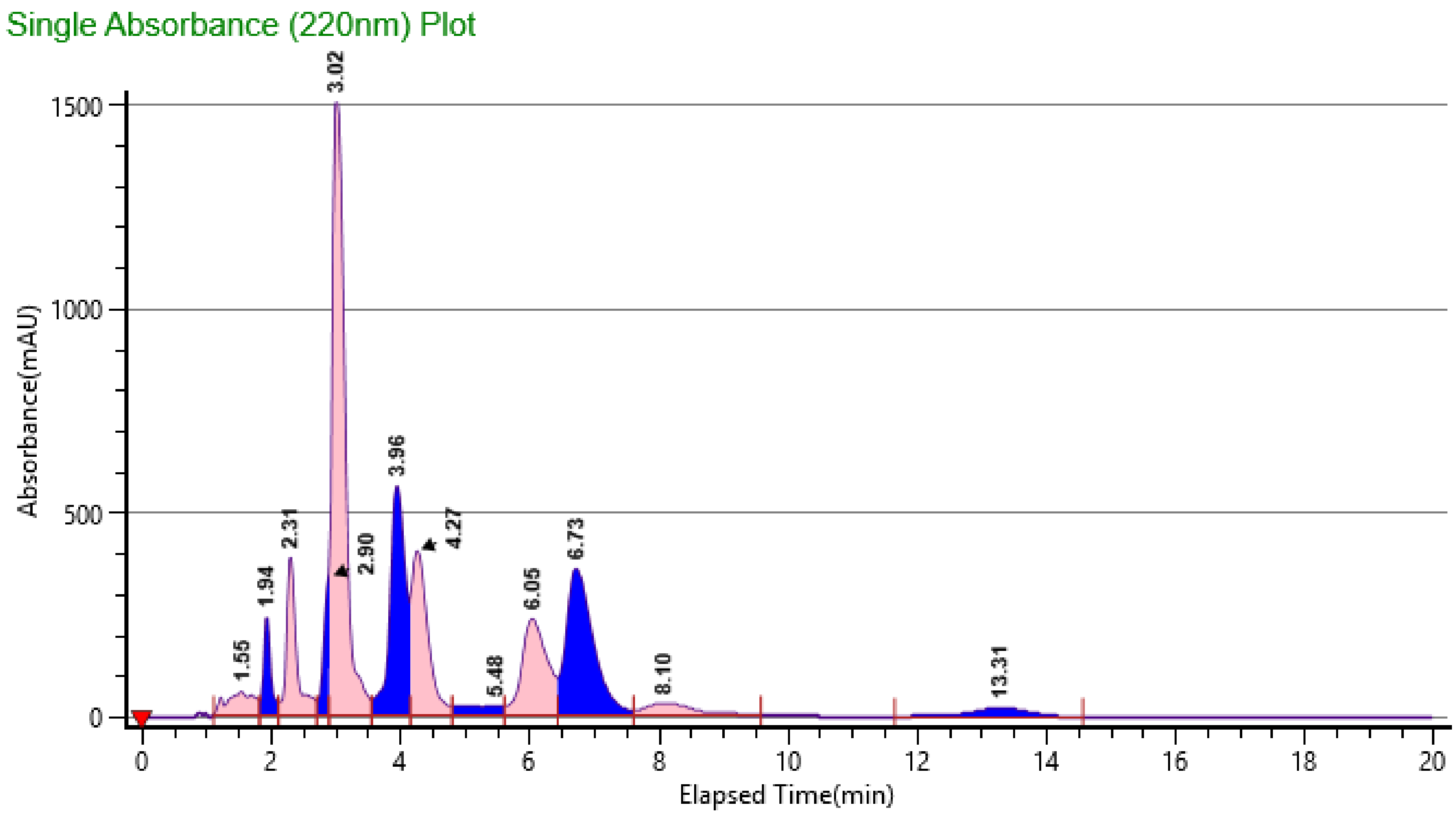

Column: Chiral-ART Amylose SA

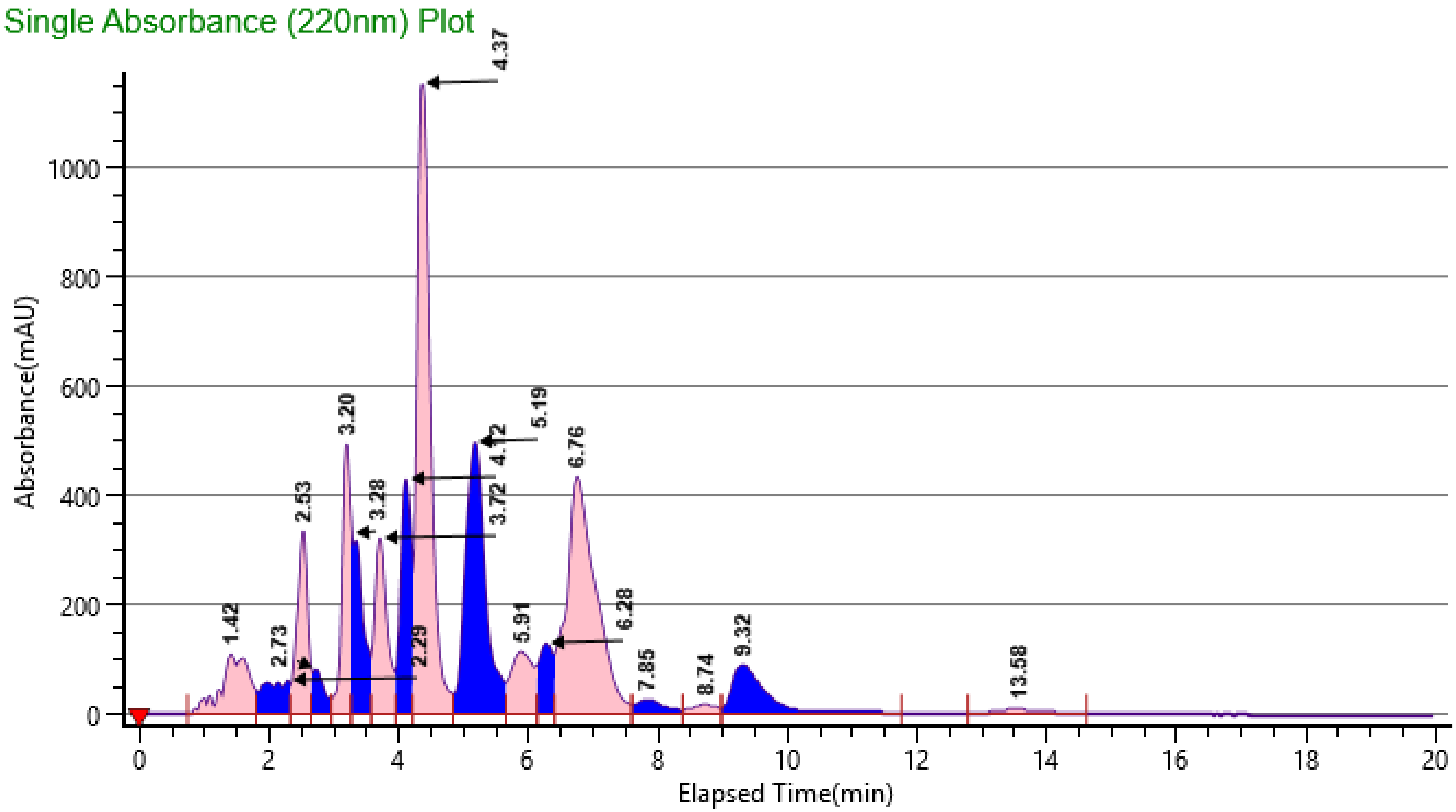

Column: (R,R) Whelk-O1

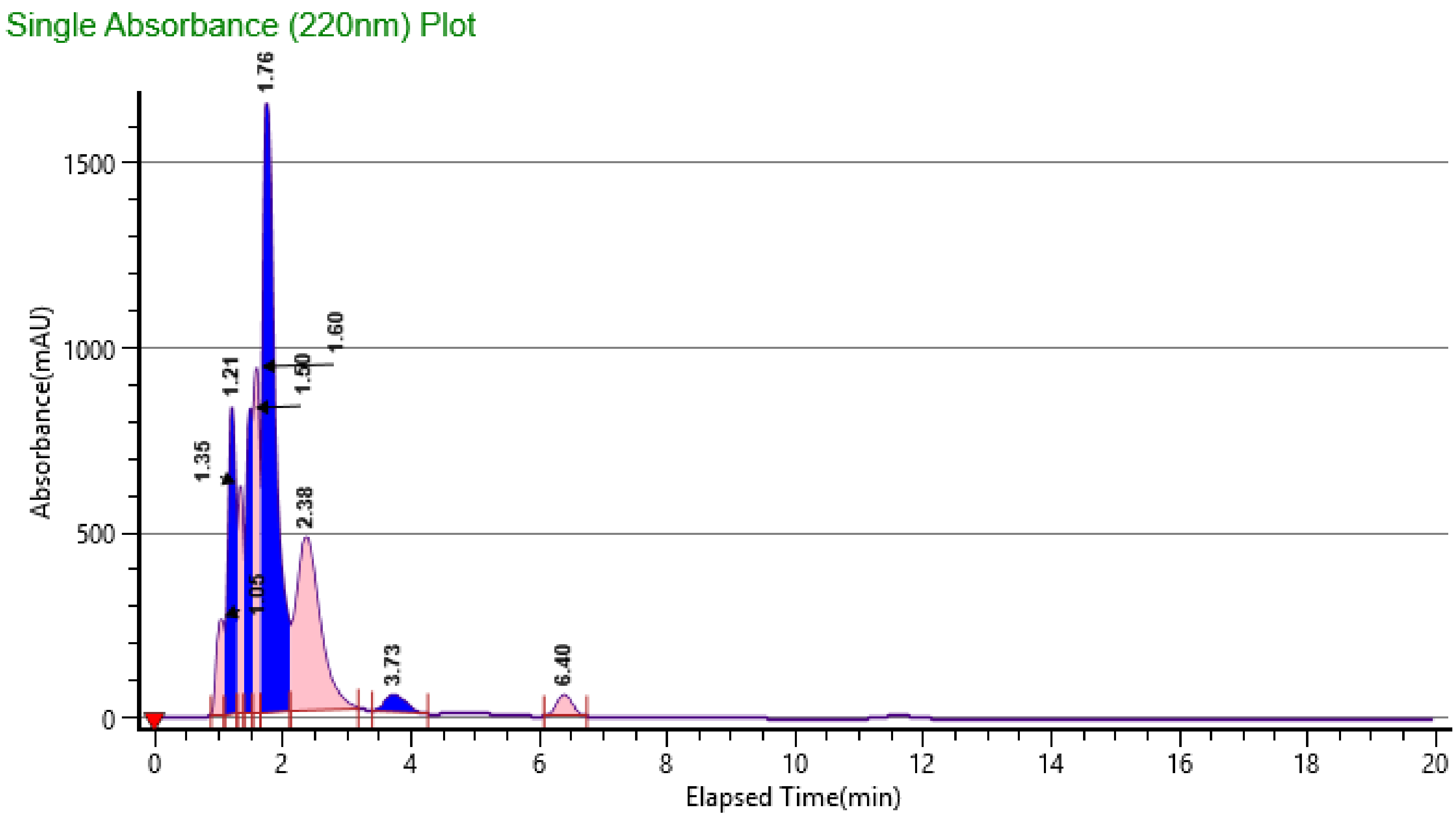

Screening-4 various columns with Acetonitrile as a Co-solvent

Column: Daicel-P4VP

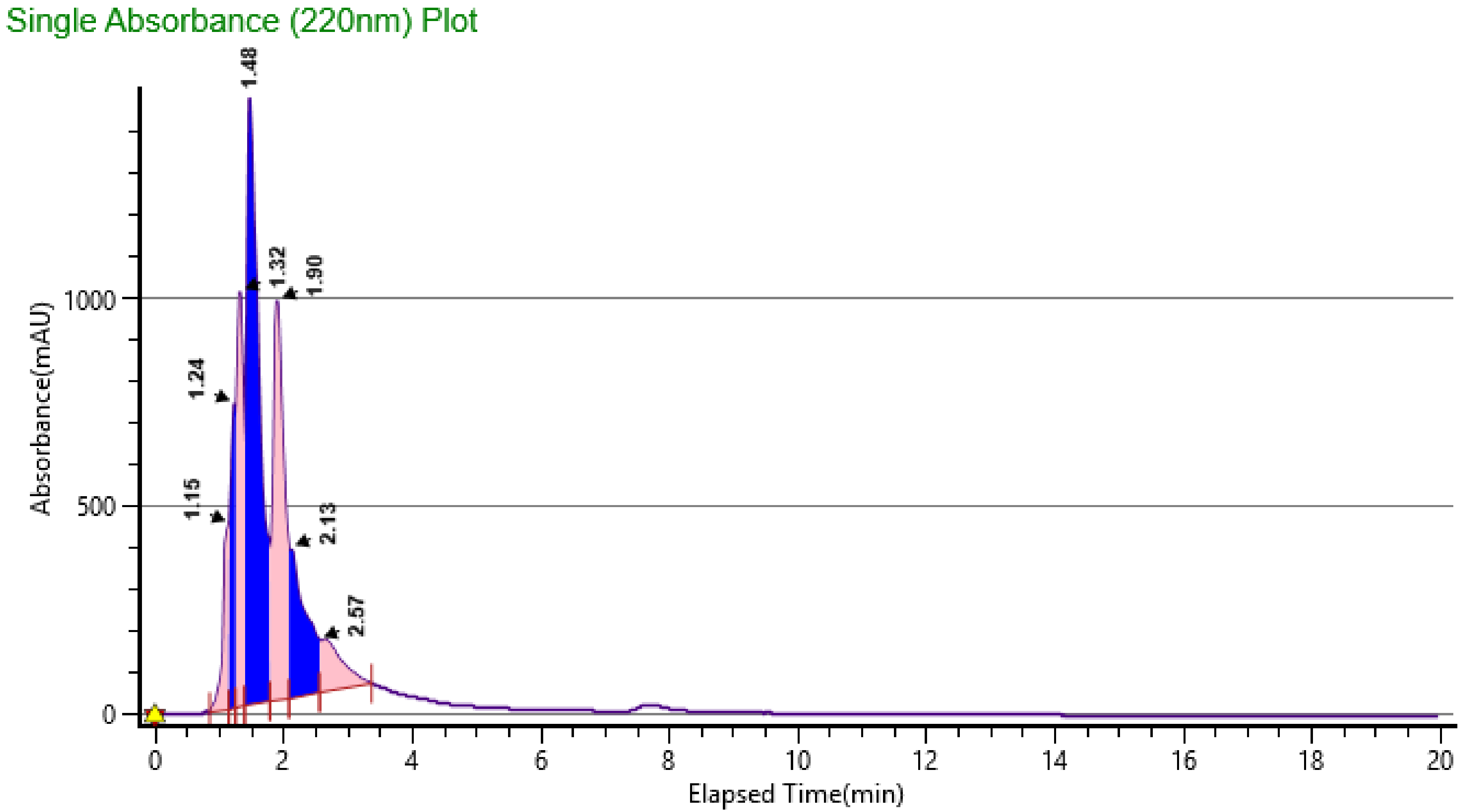

Column: LUX-i-amylose-3

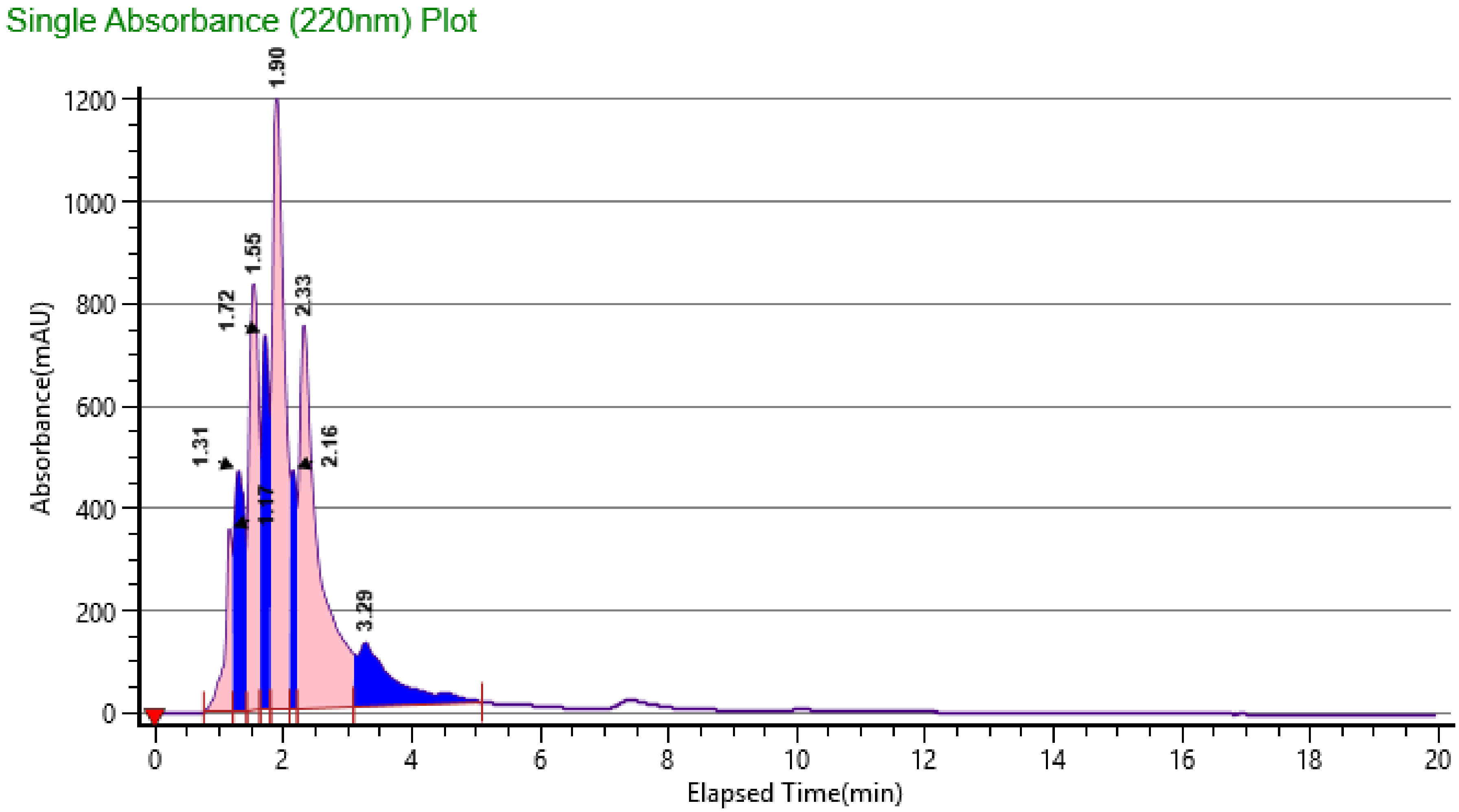

Column: Chiralpak-AD-H

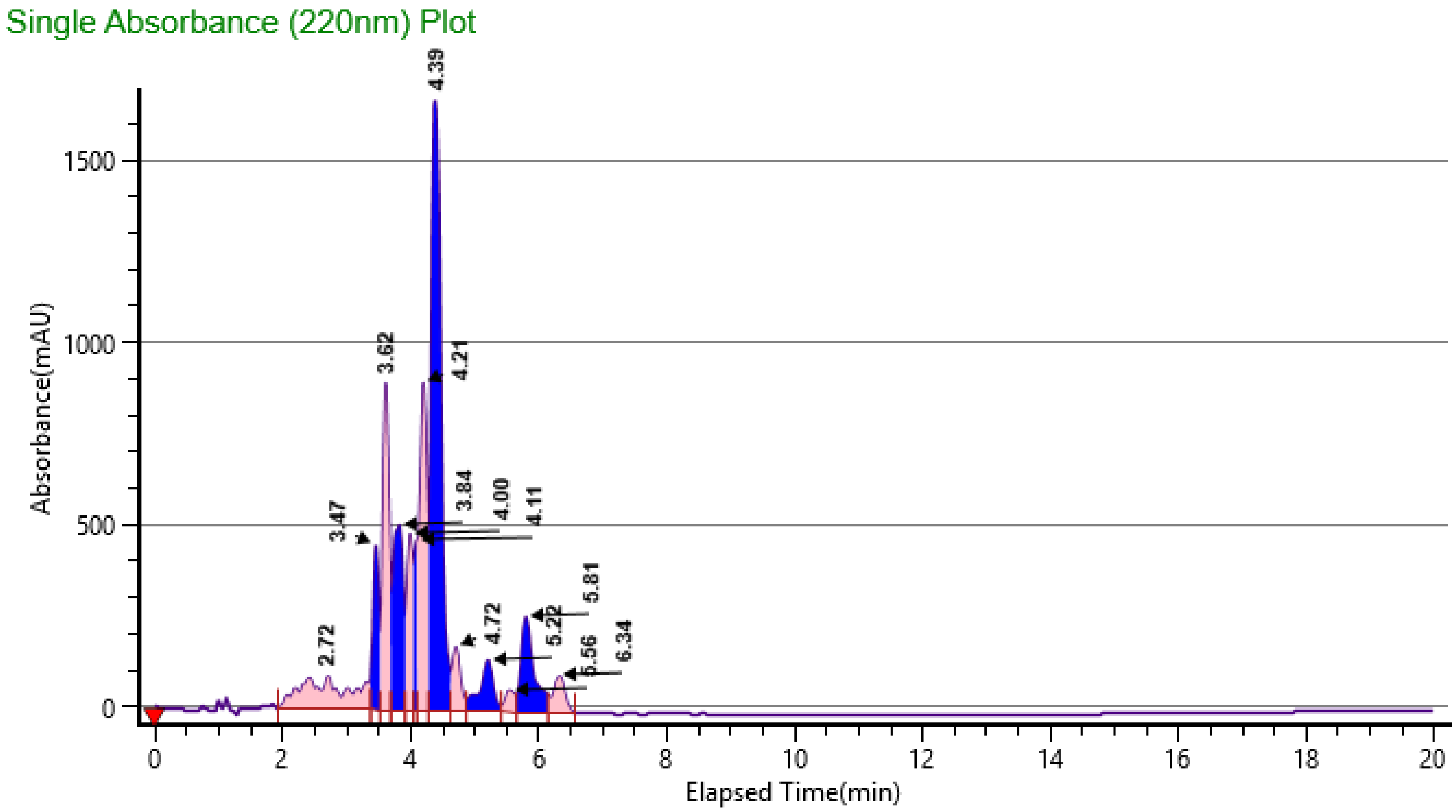

Column: LUX-Amylose-2

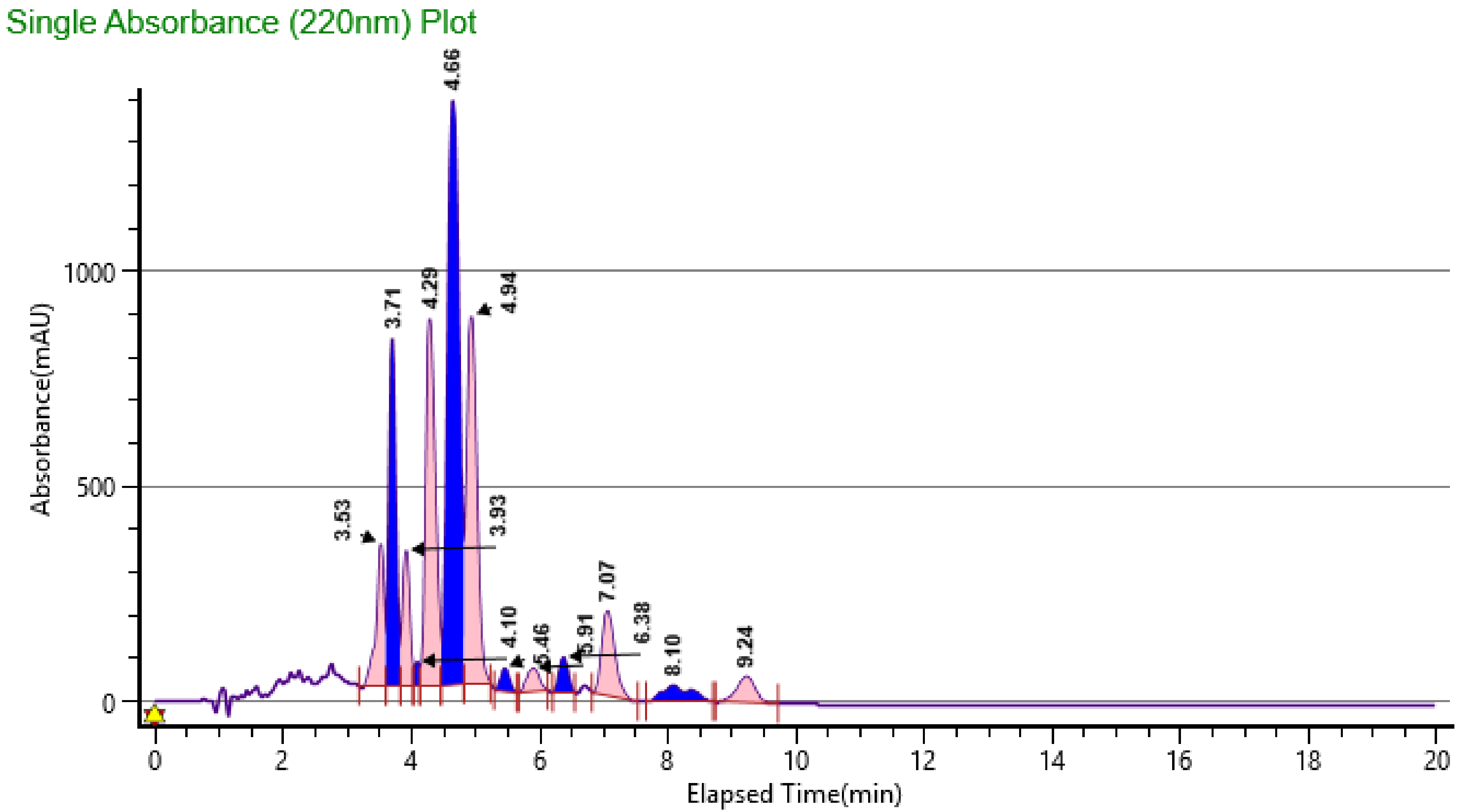

Column: Chiralpak-IG

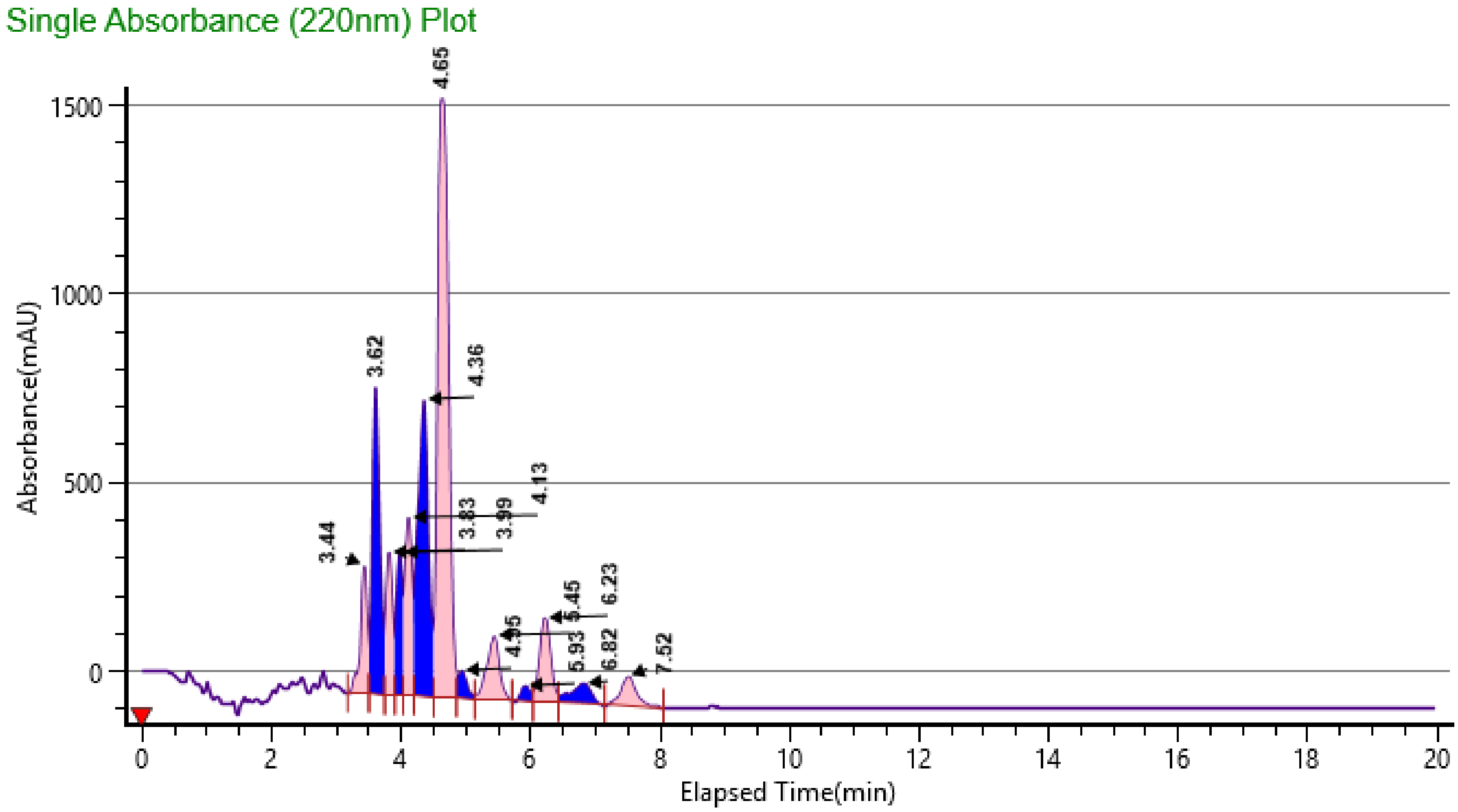

No elution with Chiral ART Amylose- SA and (R,R) Whelk-O1 columns

Figure S2. Optimized SFC method for Acetone and ethanol extract of *Swietenia macrophylla* seed

Acetone extract

Sample	SM-CRUDE
Instrument Method	4ml-35-100 BAR
Column	Chiralpak IE (4.6*250mm)5μ
Co-Solvent	Methanol
Col. Temp. (C)	35

Log Date	29-Jun-23 5:23:37 PM
Inj. Vol.(ul)	8
Sample Well	2,1,F
Flow(ml/min)	4.00
Co-Solvent% Values	35.0
Back Pressure	100 Bar

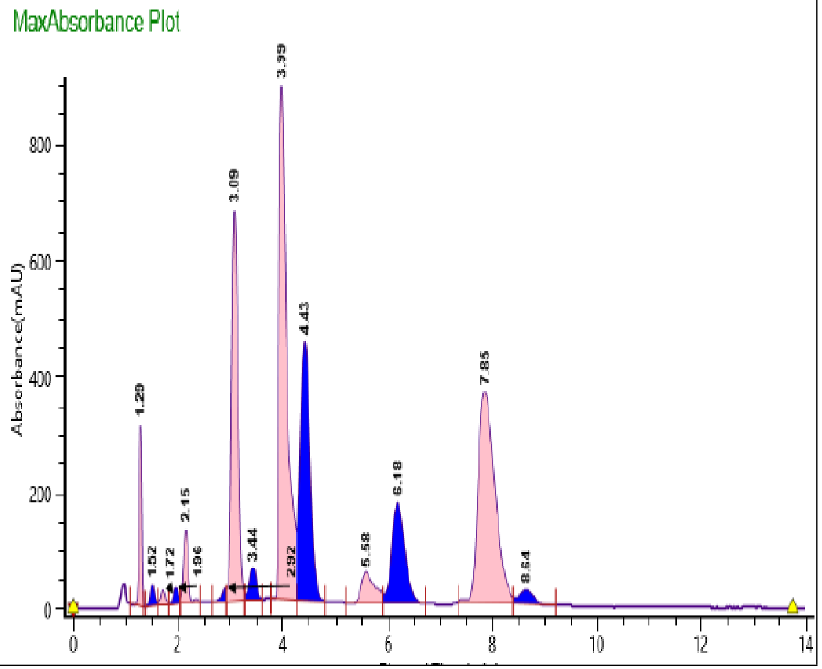

Ethanol extract

Sample	SM-CRUDE
Instrument Method	3ml-30-100 BAR
Column	Chiralpak IE (4.6*250mm)5μ
Co-Solvent	Methanol
Col. Temp. (C)	35

Log Date	27-Jun-23 7:11:19 PM
Inj. Vol.(ul)	8
Sample Well	2,1,F
Flow(ml/min)	3.00
Co-Solvent% Values	30.0
Back Pressure	99 Bar

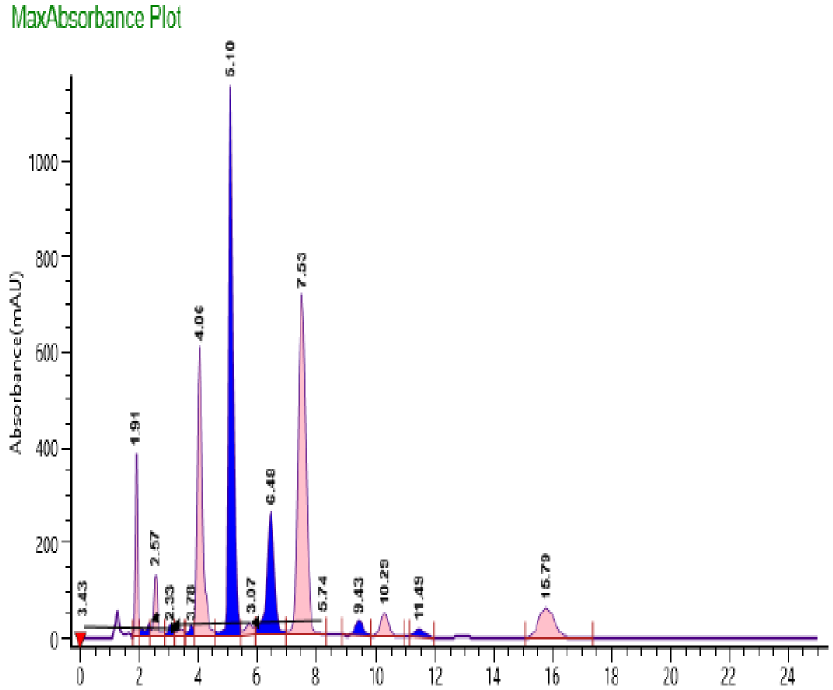

Figure S3. ^{1}H NMR Spectrum of compound **1** at (400 MHz) in DMSO- *d6*

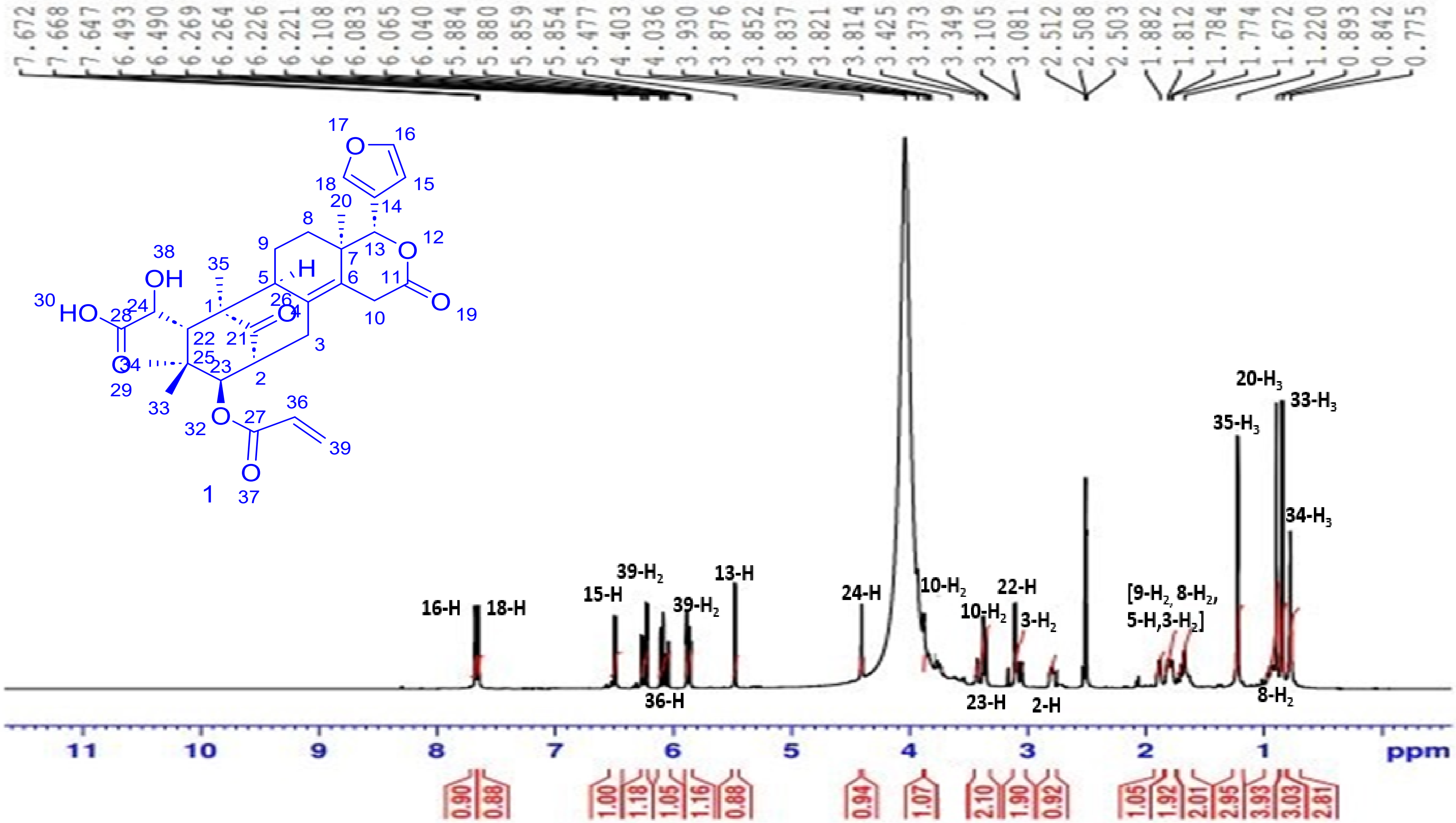

Figure S4. ^{13}C NMR NMR Spectrum of compound **1** at (400 MHz) in DMSO- *d6*

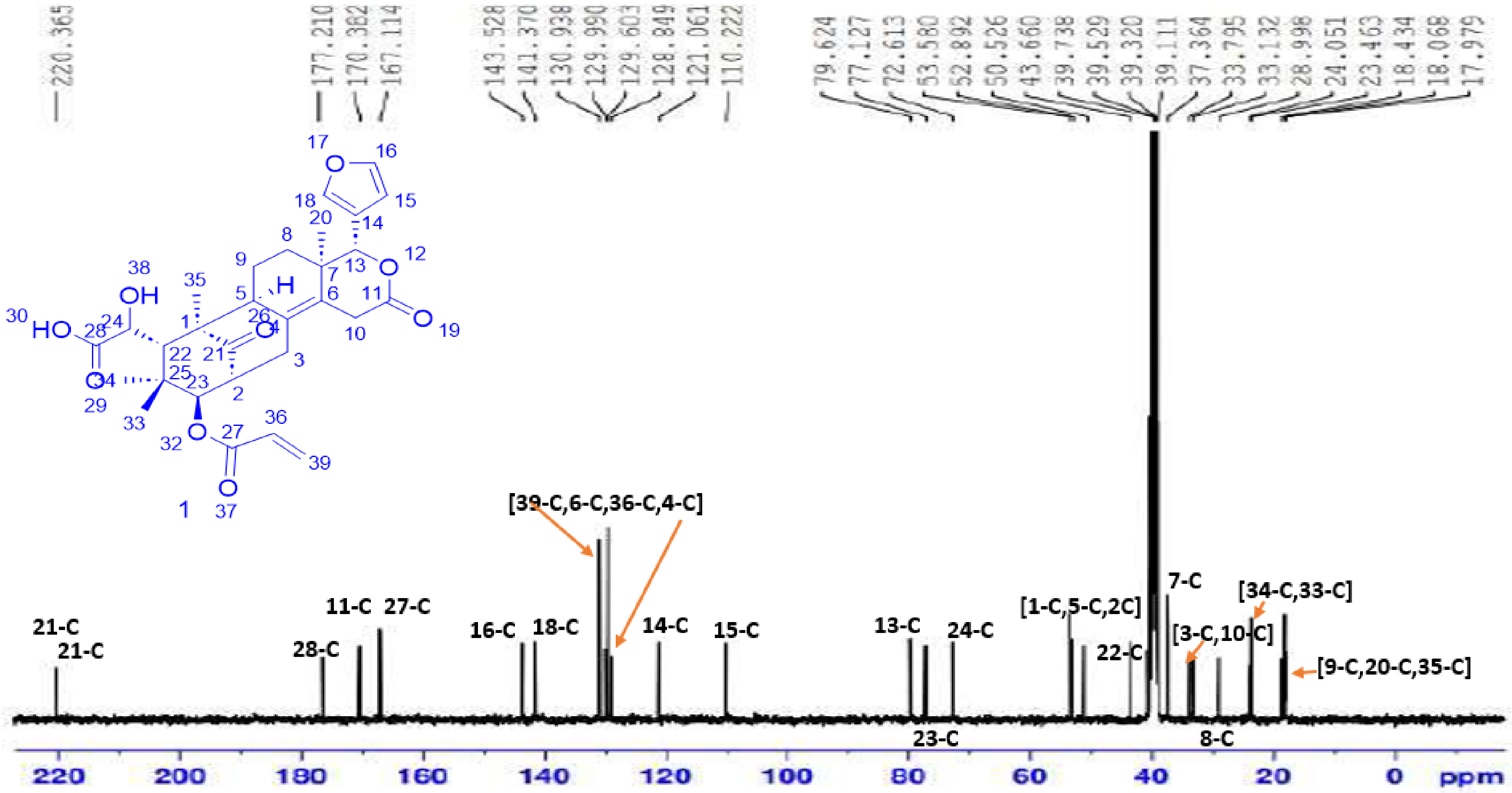

Figure S5. HSQC Spectrum of compound **1** at (400 MHz) in DMSO- *d6*

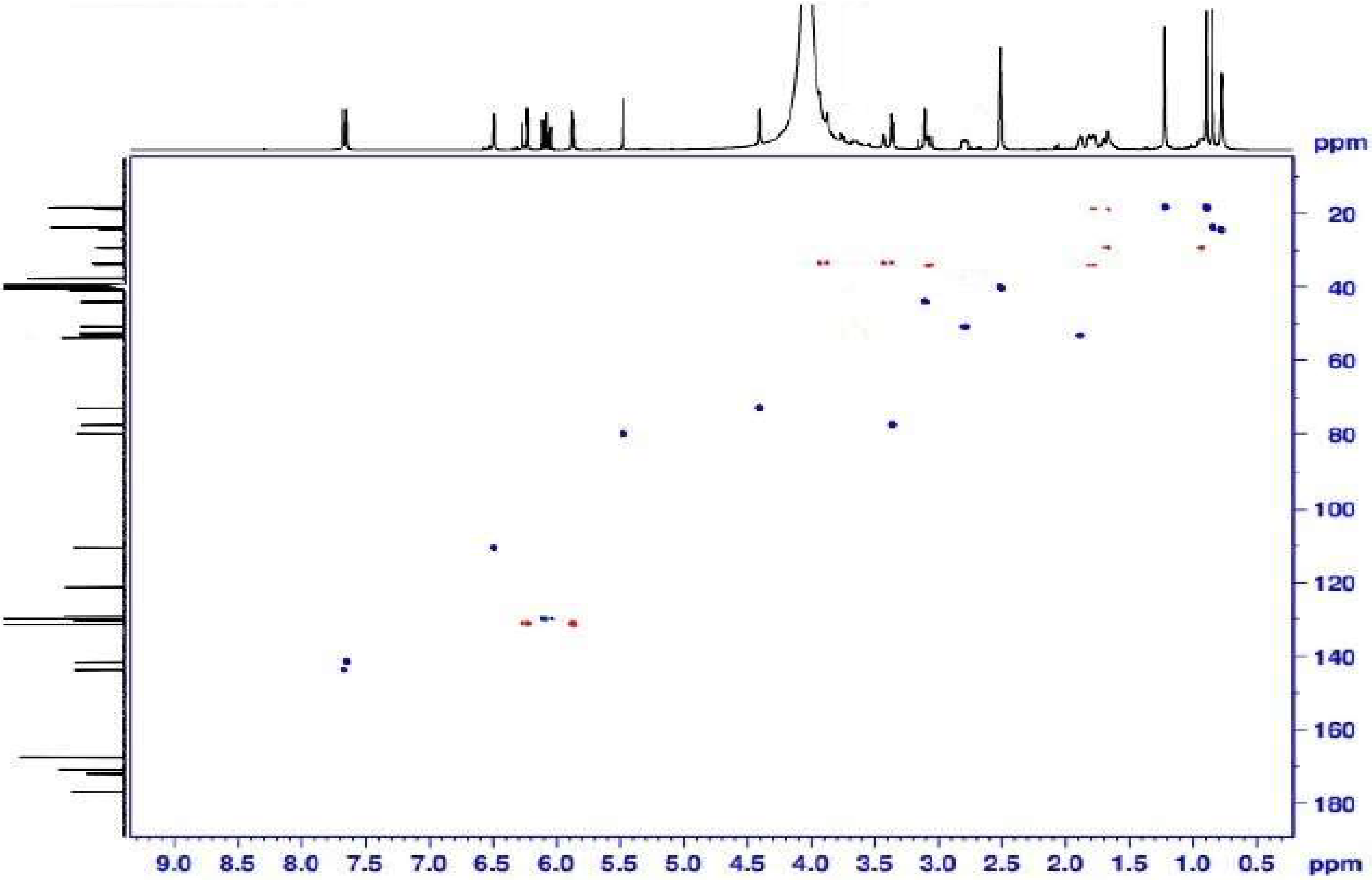

Figure S6. HMBC Spectrum of compound **1** at (400 MHz) in DMSO- *d6*

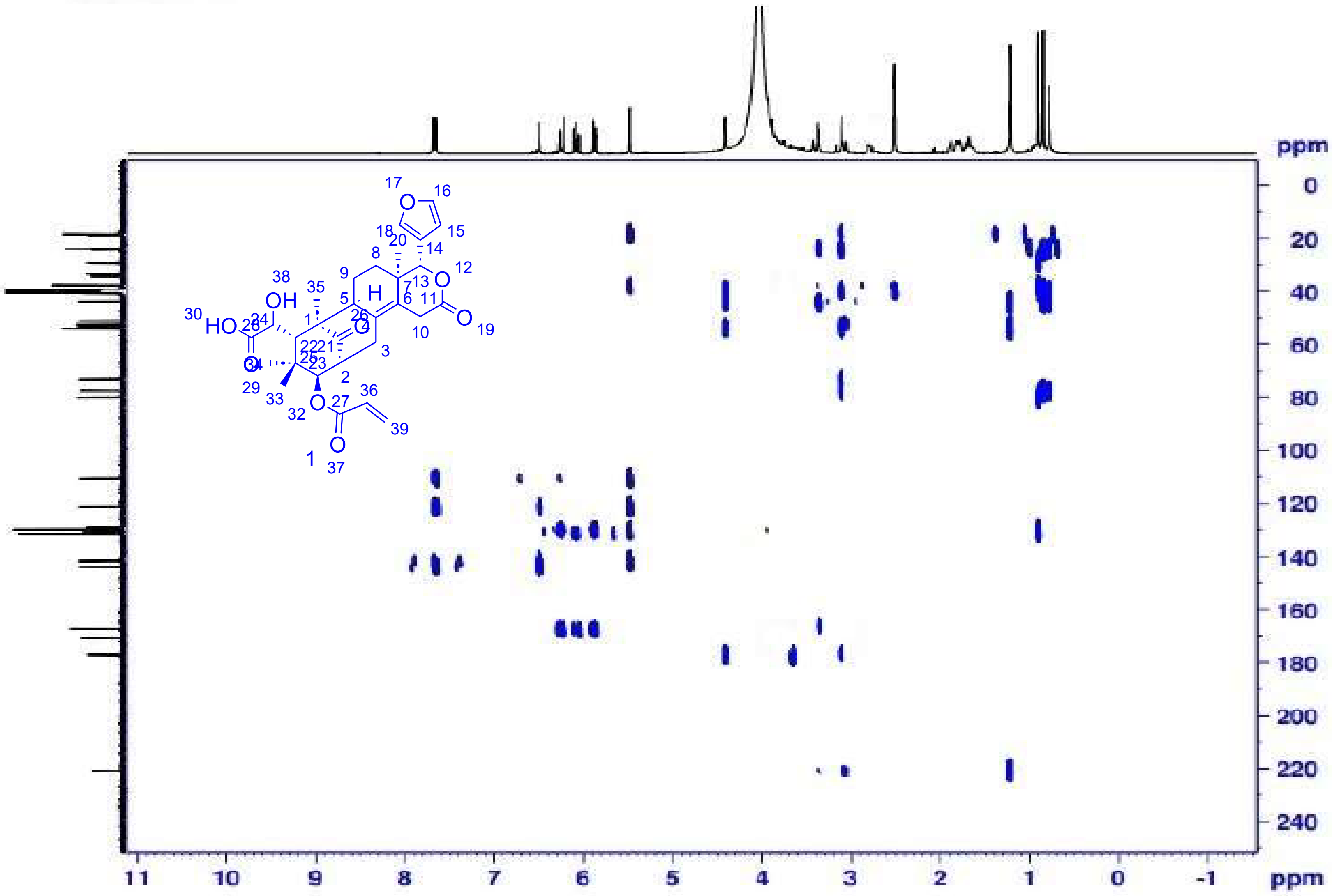

Figure S7. HRESI mass spectrum of compound **1**

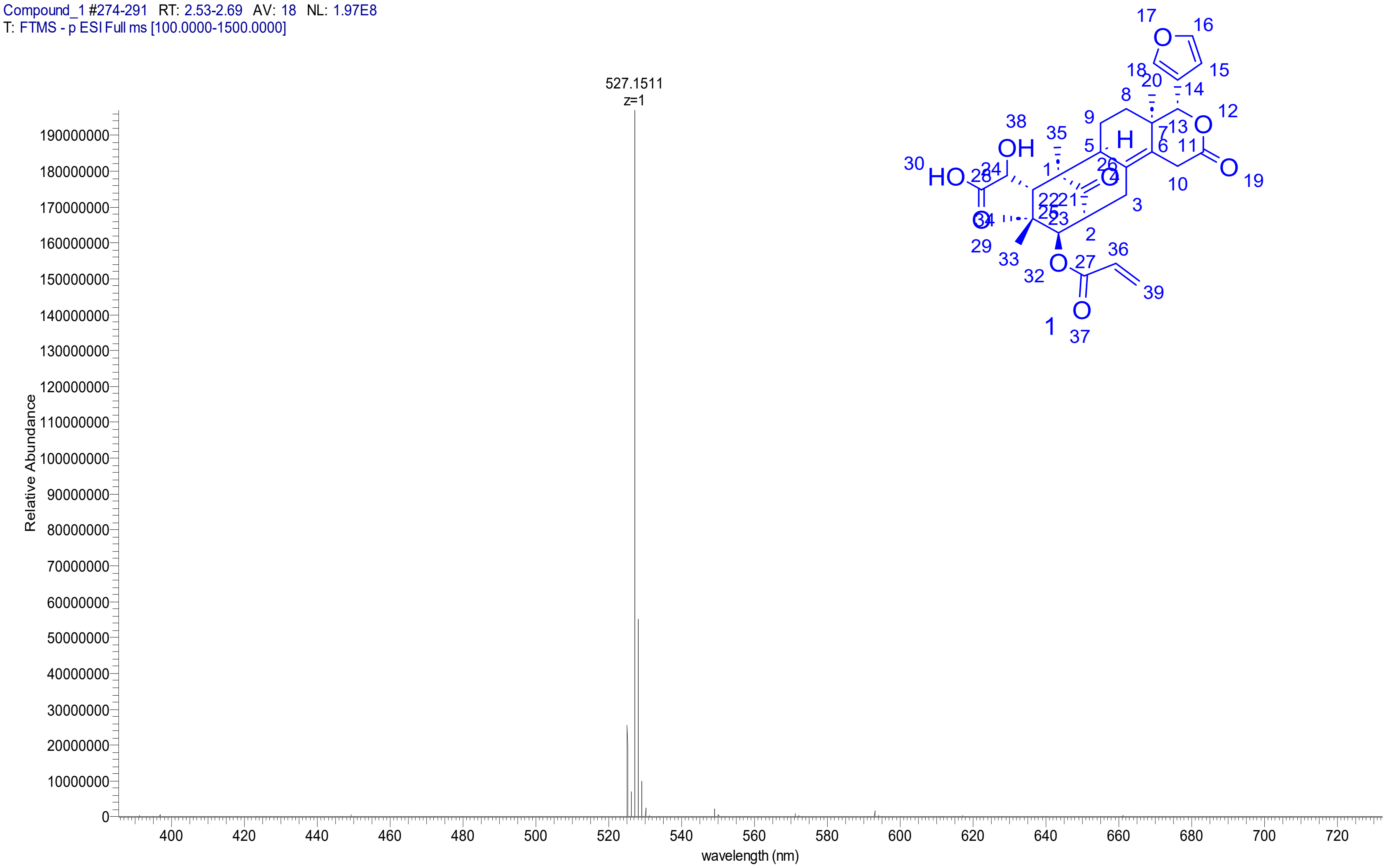

Figure S8. IR spectrum of compound **1**

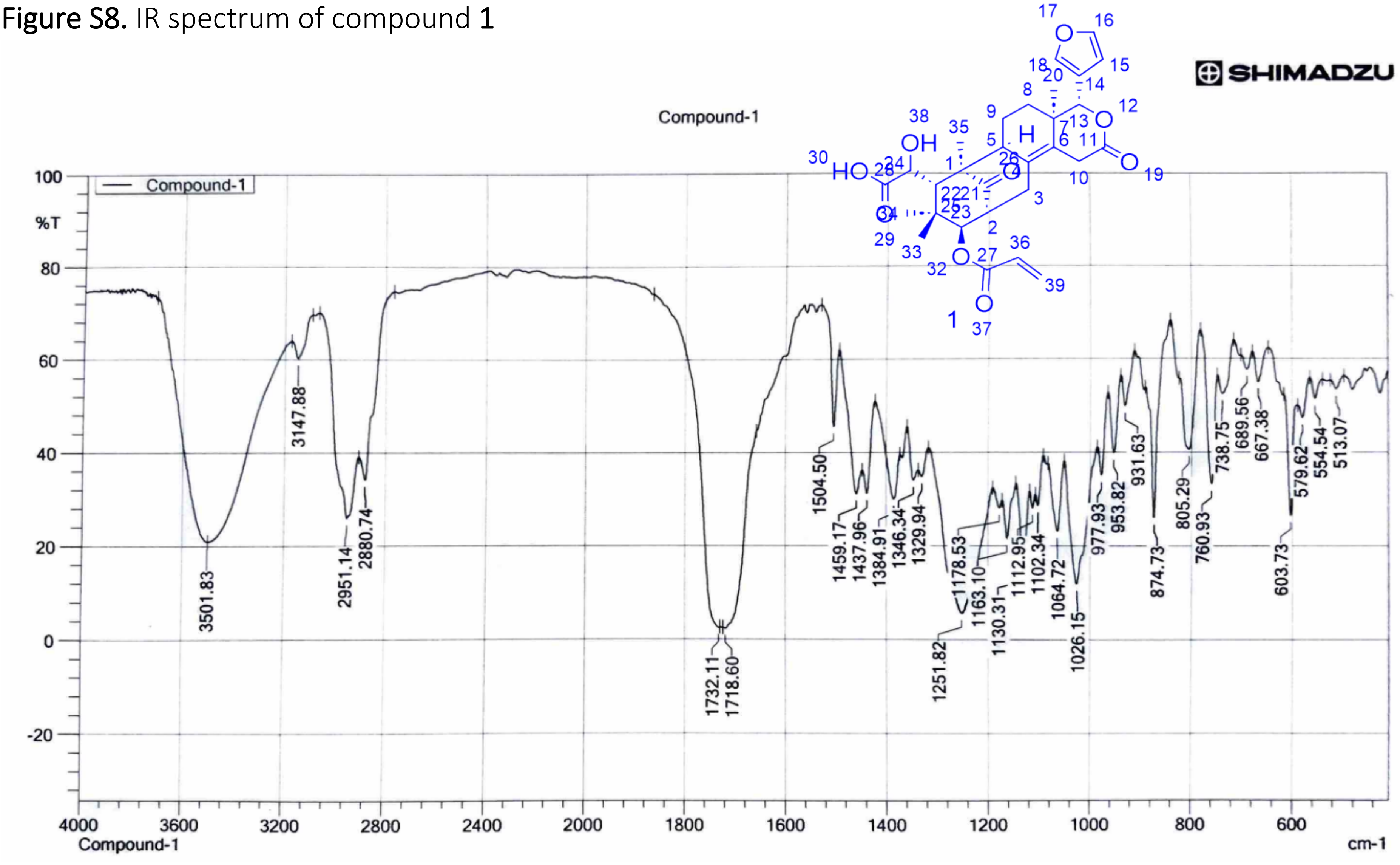

Figure S9. Optical rotation of compound 1

Comment	0.1% in Methanol
Username	
Division	
Organization	Aragen Life Sciences Pvt Ltd

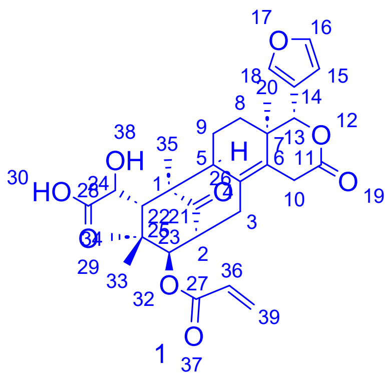

1

[Measurement Information]

Instrument Name	Polarimeter
Model Name	P-2000
Serial No.	B209561232
Polarizer	Dichrom
Faraday Cell	Flint Glass
Accessory	PTC-262
Accessory S/N	C051961481
Temperature	25.00 C
Control Sonsor	Holder
Monitor Sensor	Holder
Start Mode	Keep target temperature +/-0.10 C while 5 seconds
Light Source	WI
Monitor wavelength	589 nm
D.I.T.	5 sec
No. of cycle	5
Cycle interval	5 sec
Temp. Monitor	Holder
Temp. Corr. Factor	0 at 20 C
Aperture(S)	8.0mm
Aperture(L)	Auto
Mode	Specific O.R.
Path Length	10 mm
Concentration	0.1 w/v%
Water content of sample	0 %
Factor	1

		No.	Sample No.	Calc. Data	Meas. Data	Temperature(C)	Blank	Comment
1	*	1	Compound-1-1	-180.2000	-0.0180	24.99	+0.0064	0.1% in Methanol
2	*	2	Compound-1-2	-181.2000	-0.0181	24.99	+0.0064	0.1% in Methanol
3	*	3	Compound-1-3	-181.2000	-0.0181	25.00	+0.0064	0.1% in Methanol
4	*	4	Compound-1-4	-181.2000	-0.0181	25.00	+0.0064	0.1% in Methanol
5	*	5	Compound-1-5	-180.2000	-0.0180	25.01	+0.0064	0.1% in Methanol
6	*	6	Avg.	-180.8000				
7		7	S.D	0.5477				
8		8	C.V	0.3029				

Figure S10. UV Spectra of compound **1**

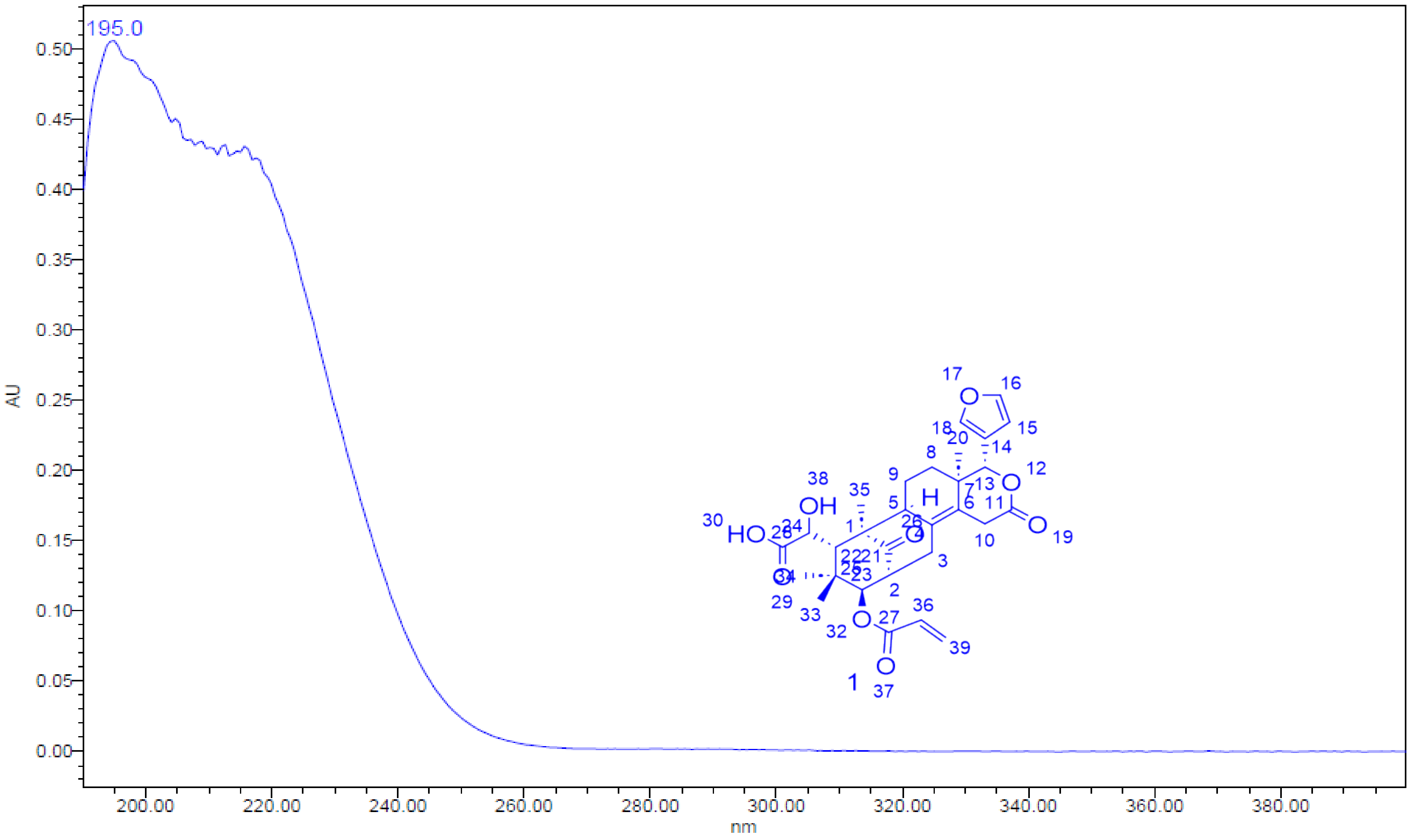

Figure S11. ^{1}H NMR Spectrum of compound **2** at (400 MHz) in DMSO- *d6*

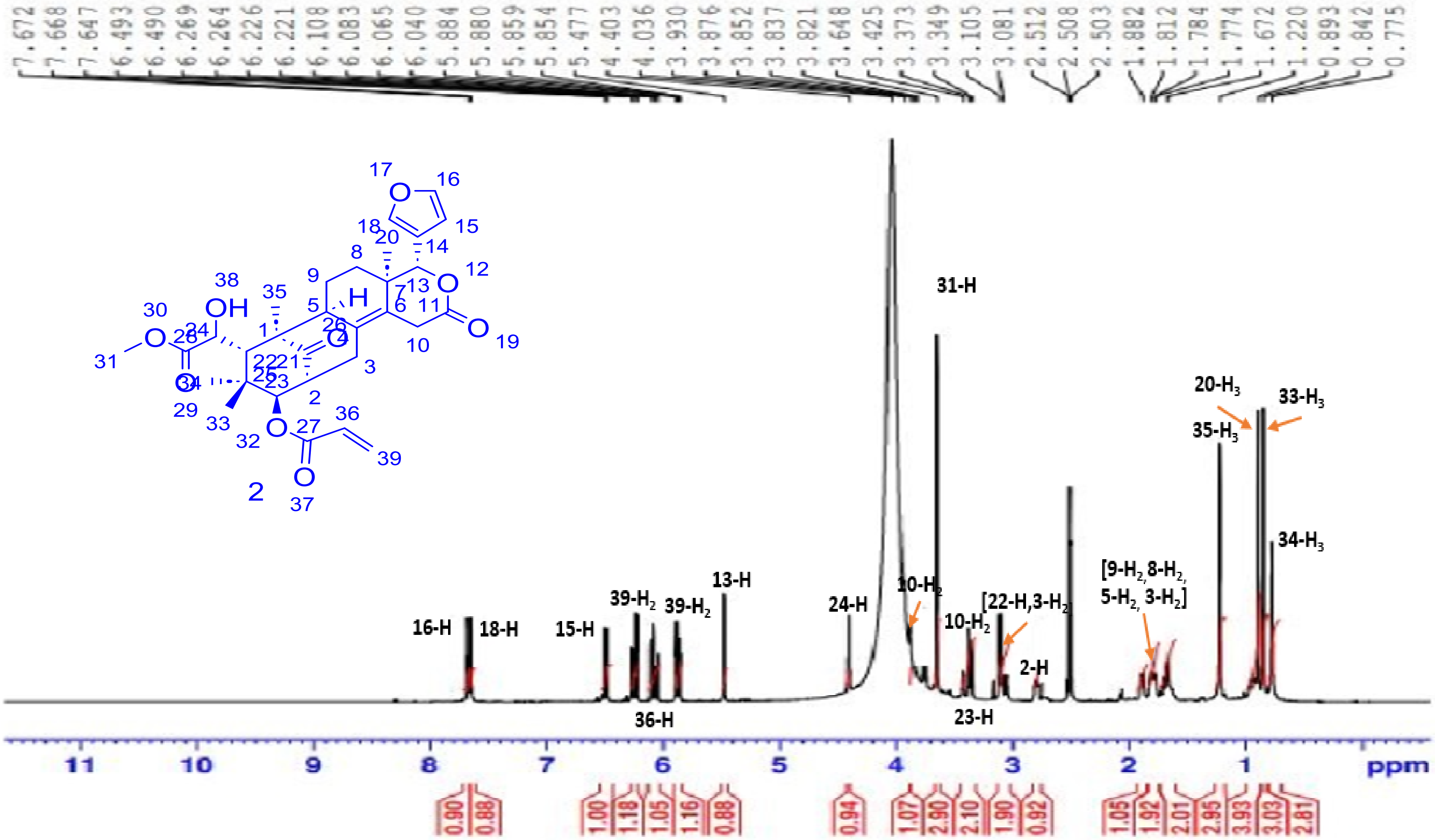

Figure S12. ^{13}C NMR Spectrum of compound **2** at (400 MHz) in DMSO- *d6*

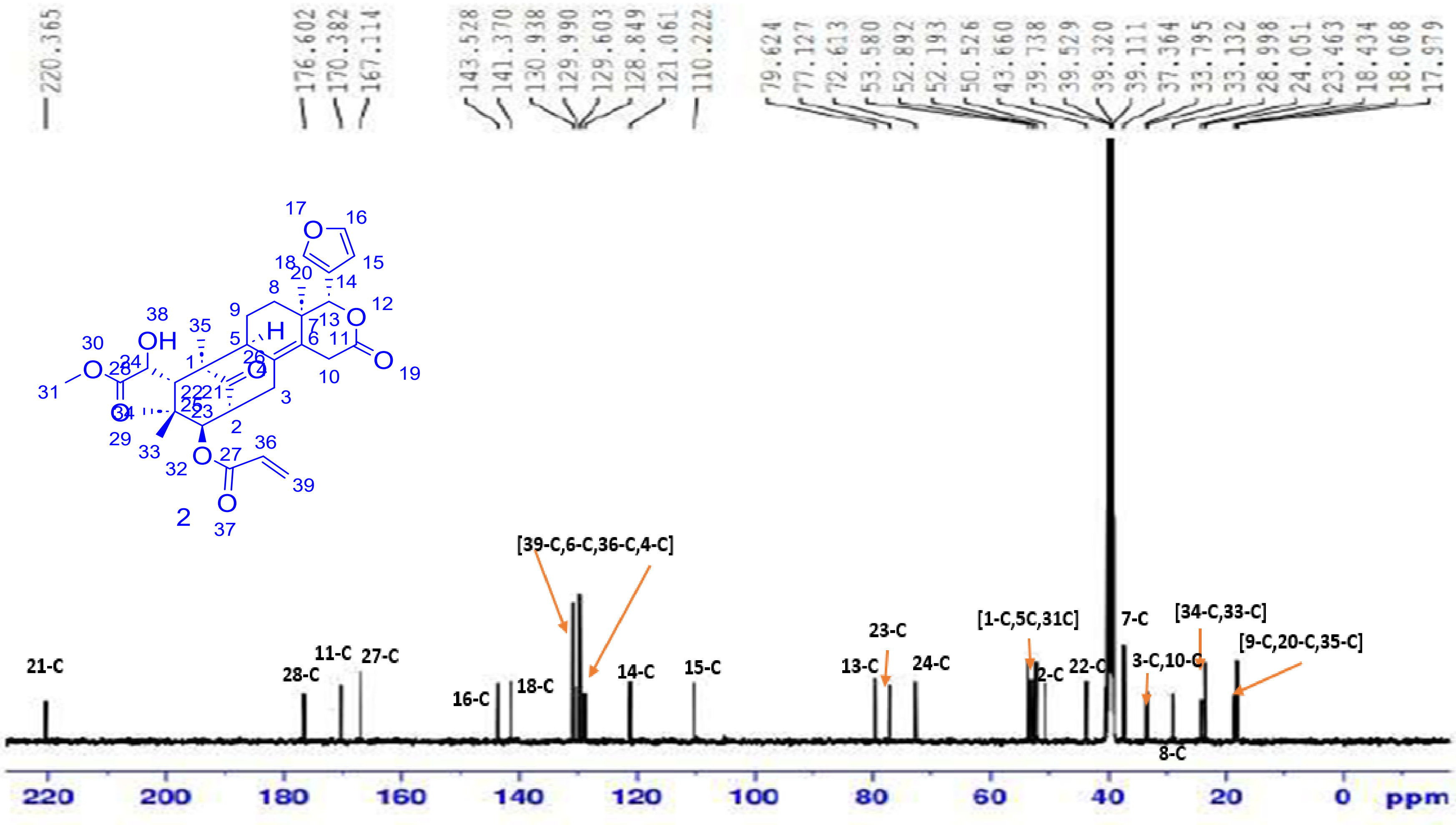

Figure S13. HSQC Spectrum of compound **2** in DMSO- *d6*

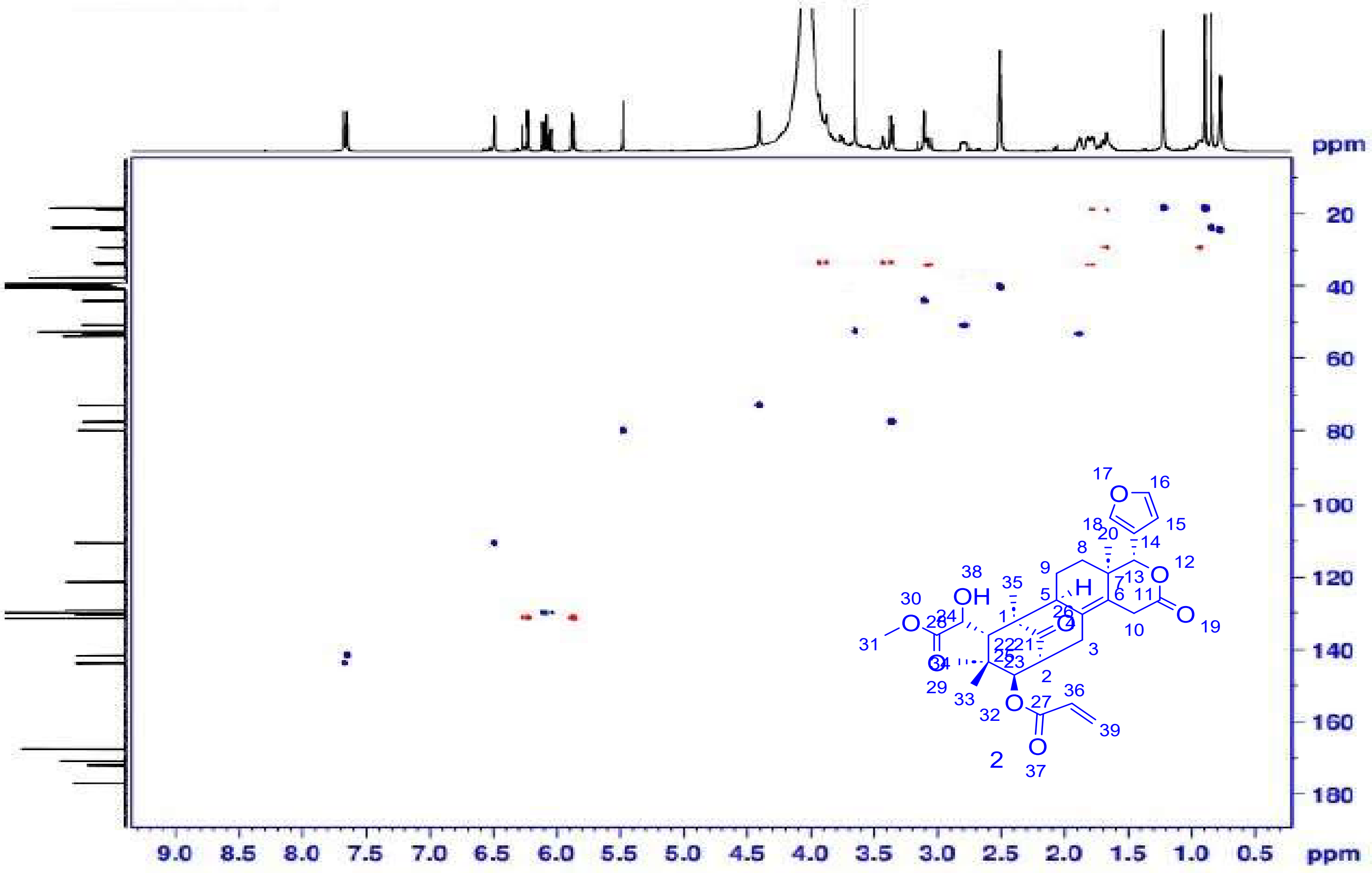

Figure S14. HMBC Spectrum of compound **2** in DMSO- *d6*

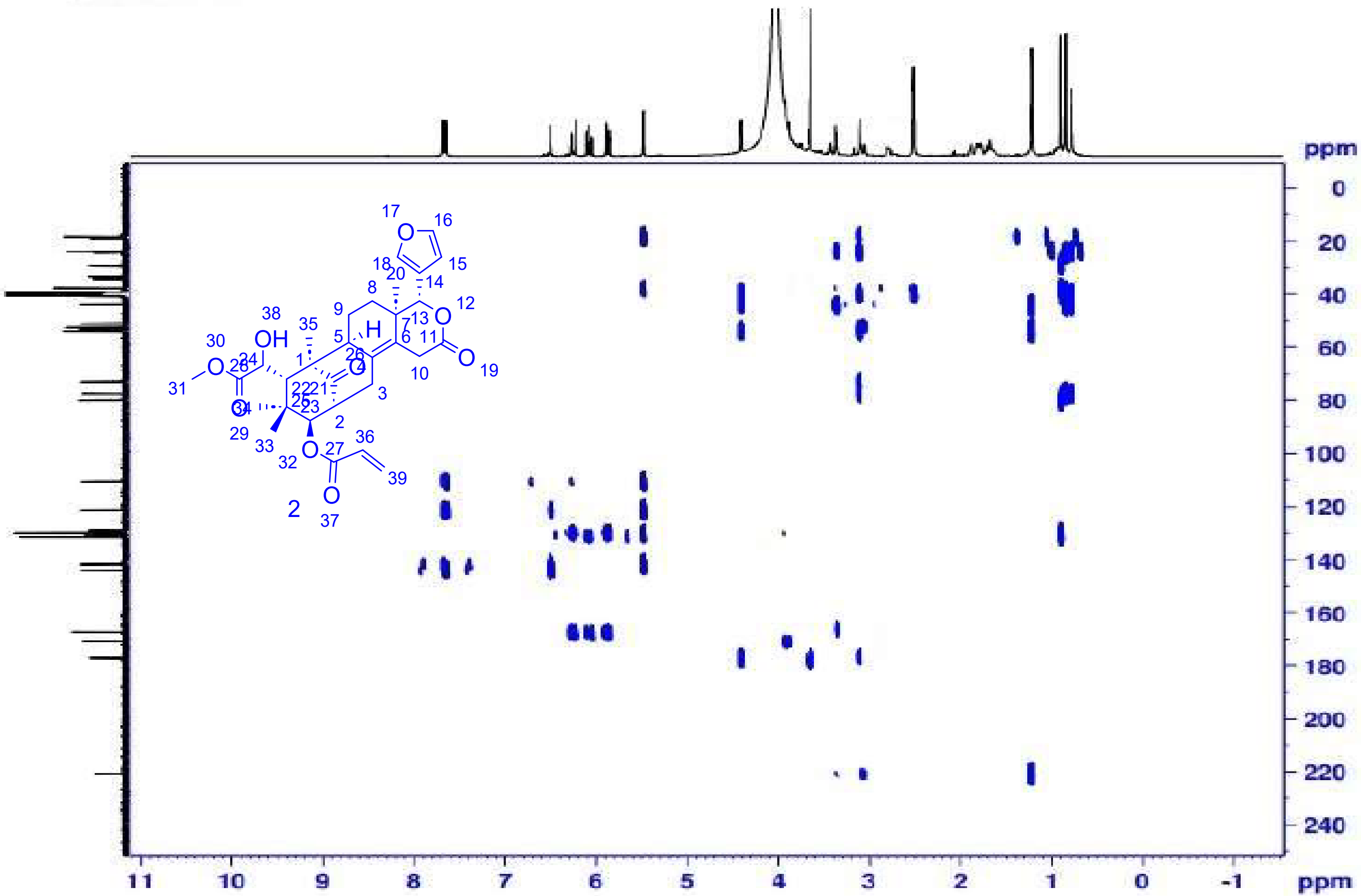

Figure S15. HRESI Mass spectrum of compound **2**

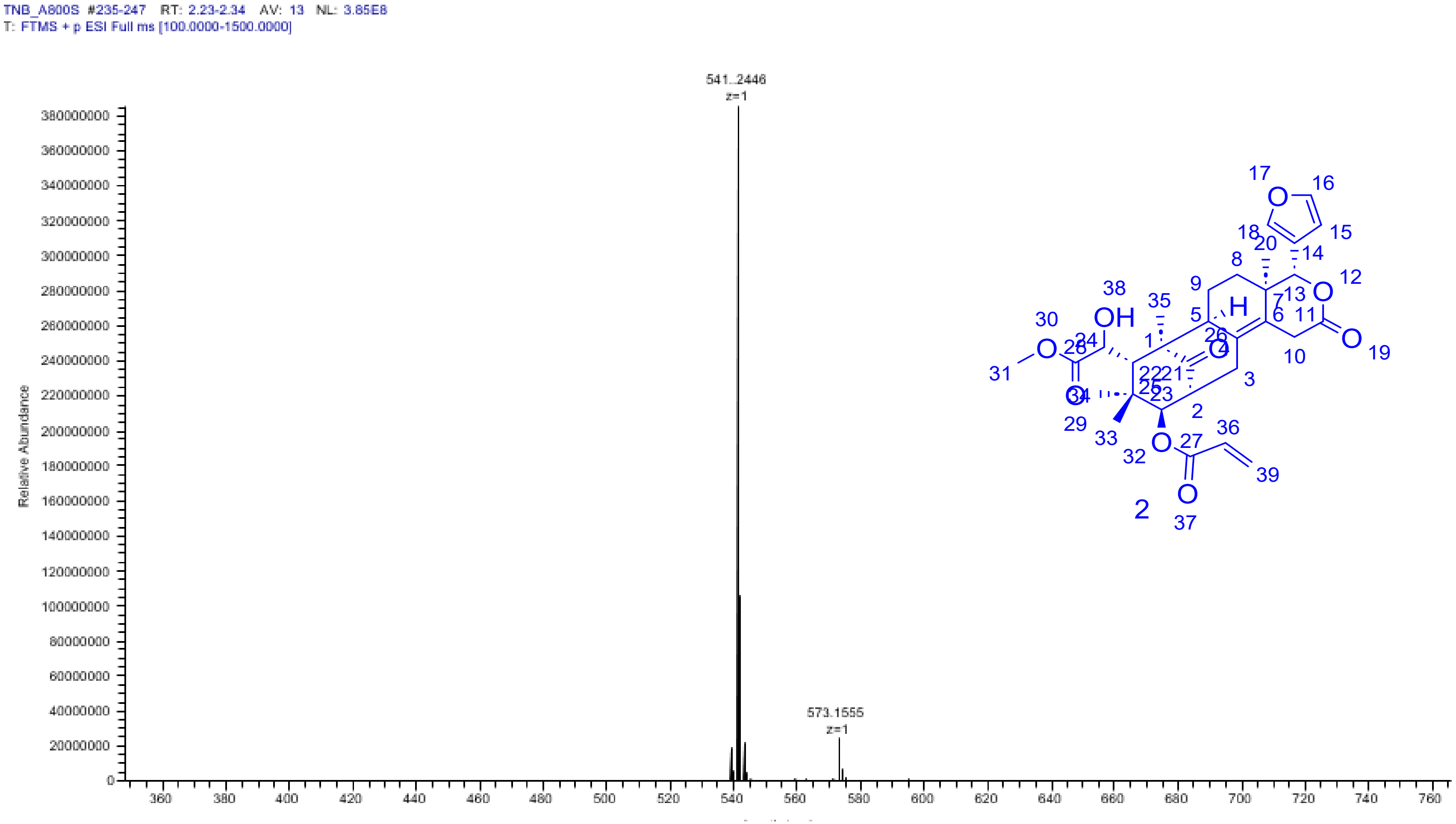

2

Figure S16. IR spectrum of compound **2**

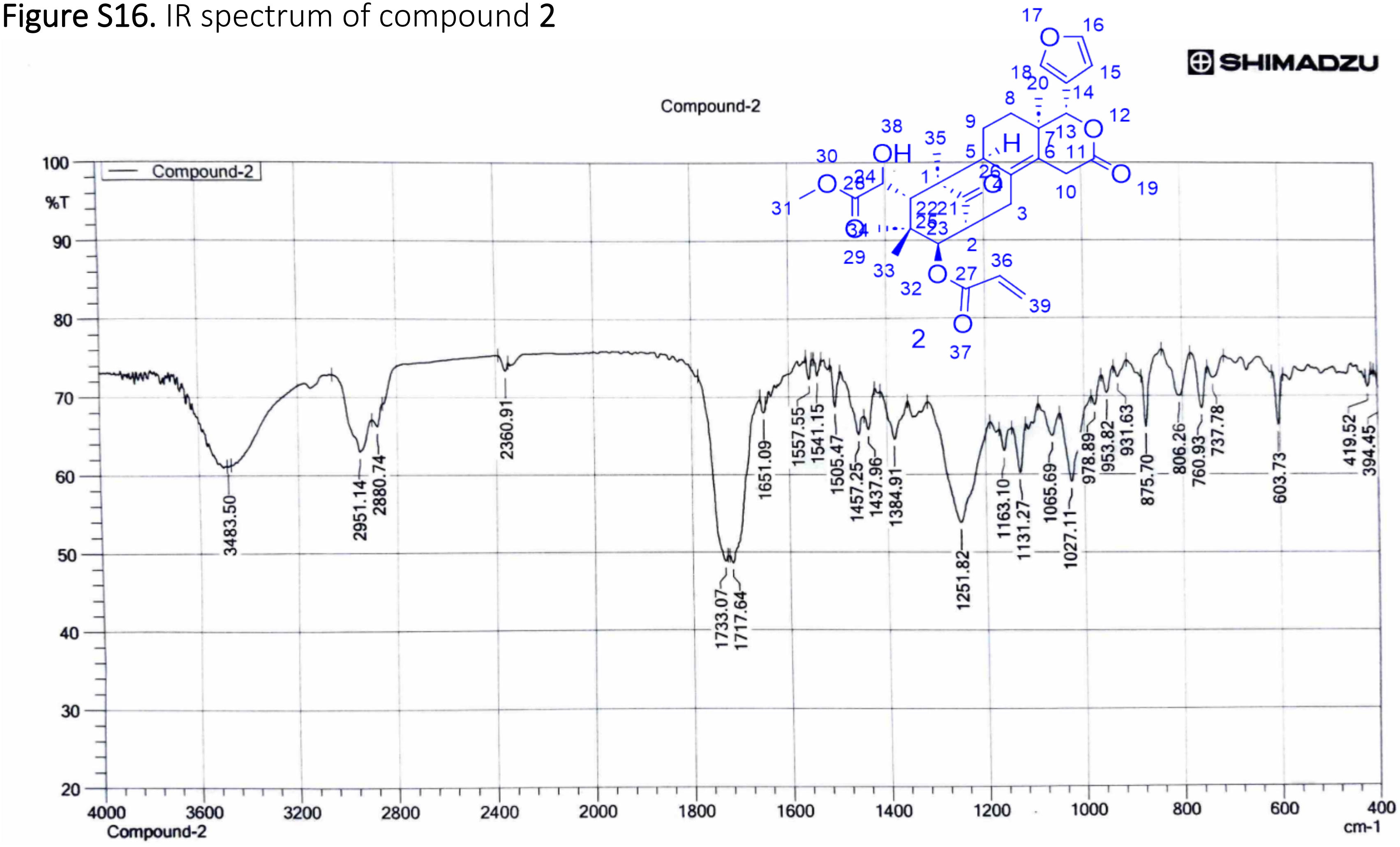

Figure S17. Optical rotation of compound **2**

[Measurement Information]

Instrument Name	Polarimeter
Model Name	P-2000
Serial No.	B209561232
Polarizer	Dichrom
Faraday Cell	Flint Glass
Accessory	PTC-262
Accessory S/N	C051961481
Temperature	25.00 C
Control Sonsor	Holder
Monitor Sensor	Holder
Start Mode	Keep target temperature +/-0.10 C while 5 seconds
Light Source	WI
Monitor wavelength	589 nm
D.I.T.	5 sec
No. of cycle	5
Cycle interval	5 sec
Temp. Monitor	Holder
Temp. Corr. Factor	0 at 20 C
Aperture(S)	8.0mm
Aperture(L)	Auto
Mode	Specific O.R.
Path Length	10 mm
Concentration	0.1 w/v%
Water content of sample	0 %
Factor	1

Comment	0.1% in Methanol
Username	
Division	
Organization	Aragen Life Sciences Pvt Ltd

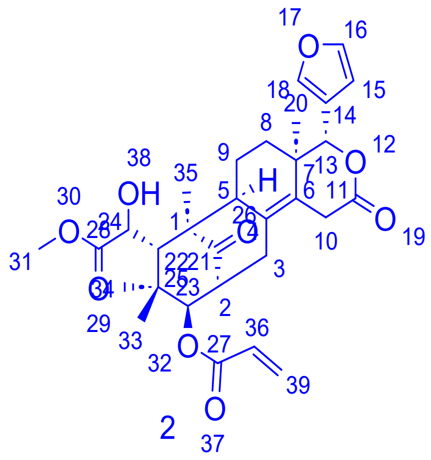

2

		No.	Sample No.	Calc. Data	Meas. Data	Temperature(C)	Blank	Comment
1	*	1	Compound-2-1	-122.2000	-0.0122	25.01	+0.0064	0.1% in Methanol
2	*	2	Compound-2-2	-117.2000	-0.0117	25.01	+0.0064	0.1% in Methanol
3	*	3	Compound-2-3	-121.2000	-0.0121	25.00	+0.0064	0.1% in Methanol
4	*	4	Compound-2-4	-116.2000	-0.0116	25.00	+0.0064	0.1% in Methanol
5	*	5	Compound-2-5	-120.2000	-0.0120	24.99	+0.0064	0.1% in Methanol
6	*	6	Avg.	-119.4000				
7		7	S.D	2.5884				
8		8	C.V	2.1679				

Figure S18. UV spectra of compound **2**

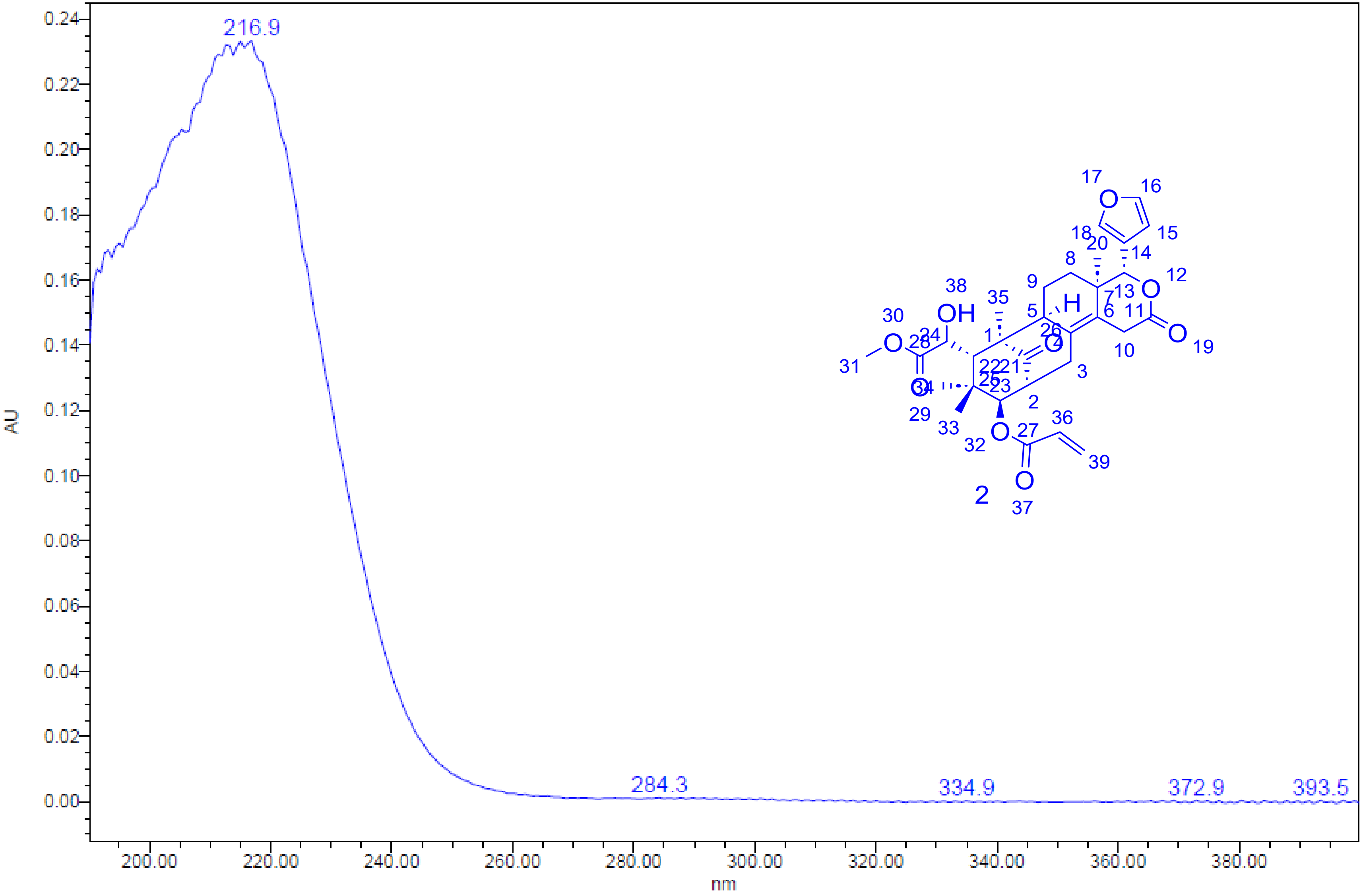

Figure S19. ^{1}H NMR Spectrum of compound 3 at (400 MHz) in DMSO- *d6*

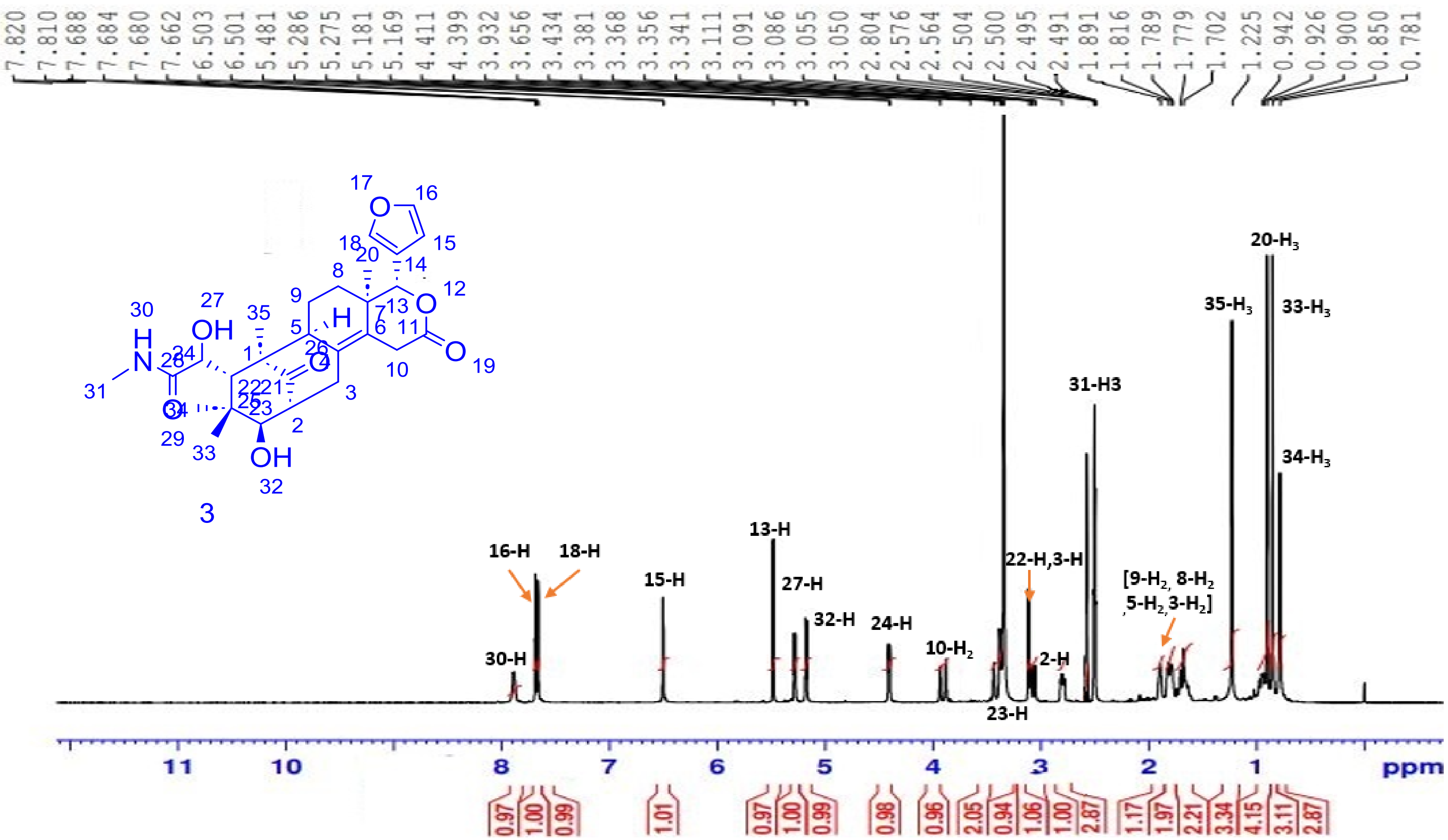

Figure S20. ^{13}C NMR Spectrum of compound **3** at (400 MHz) in DMSO- *d6*

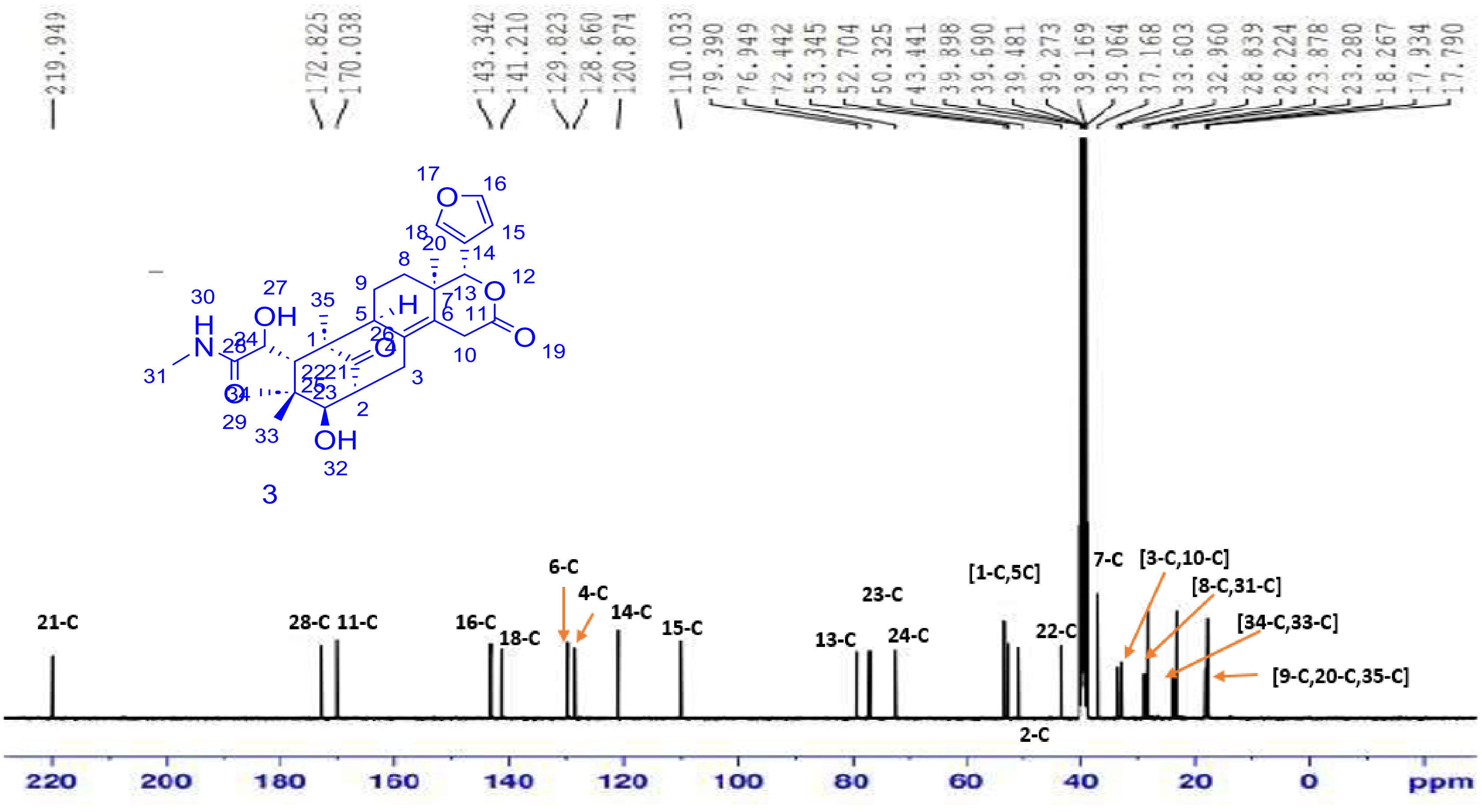

Figure S21. HSQC Spectrum of compound **3** in DMSO- *d6*

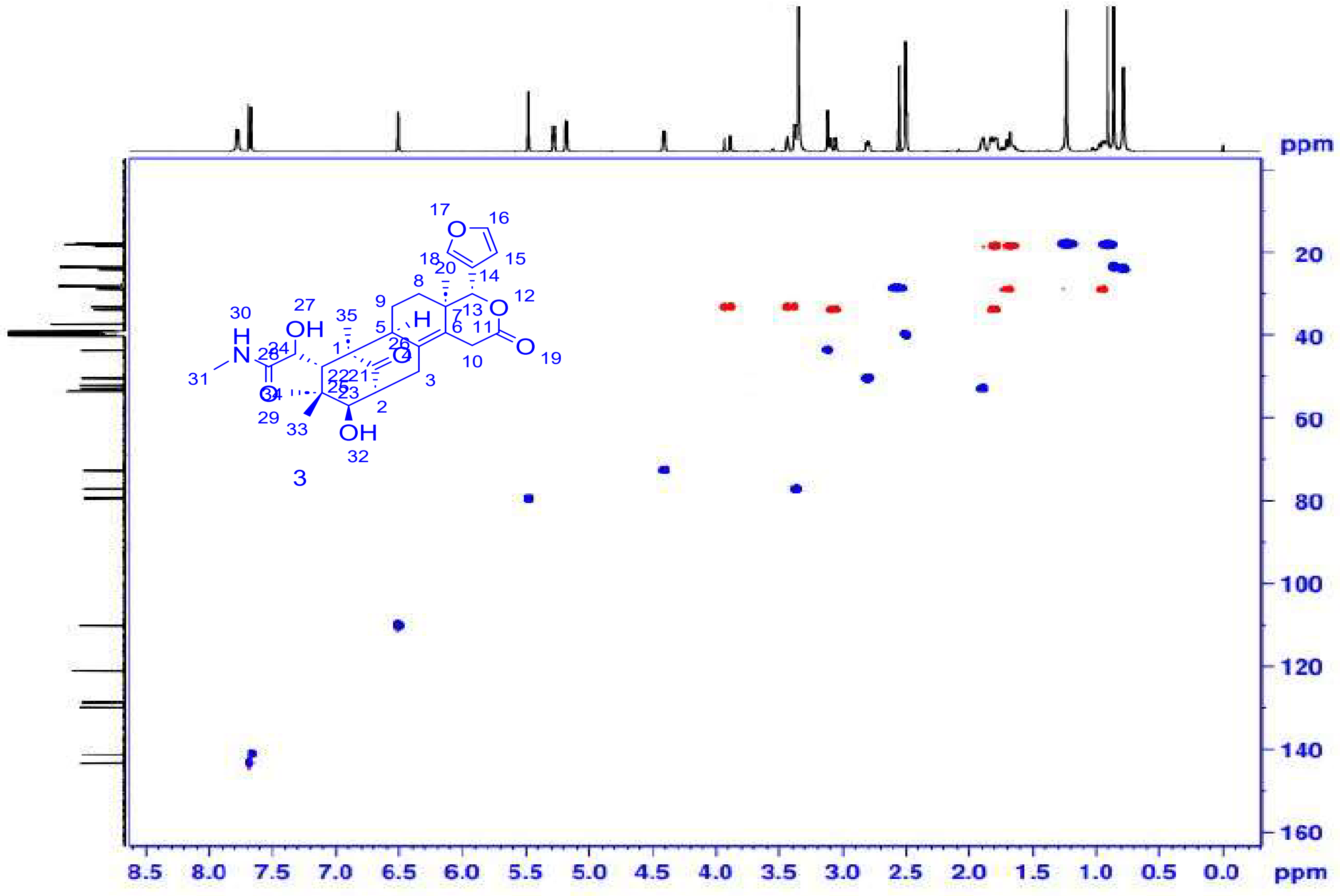

Figure S22. HMBC Spectrum of compound **3** in DMSO- d6

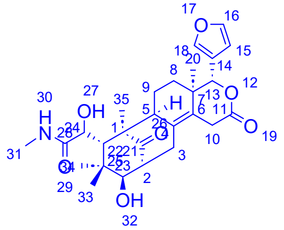

3

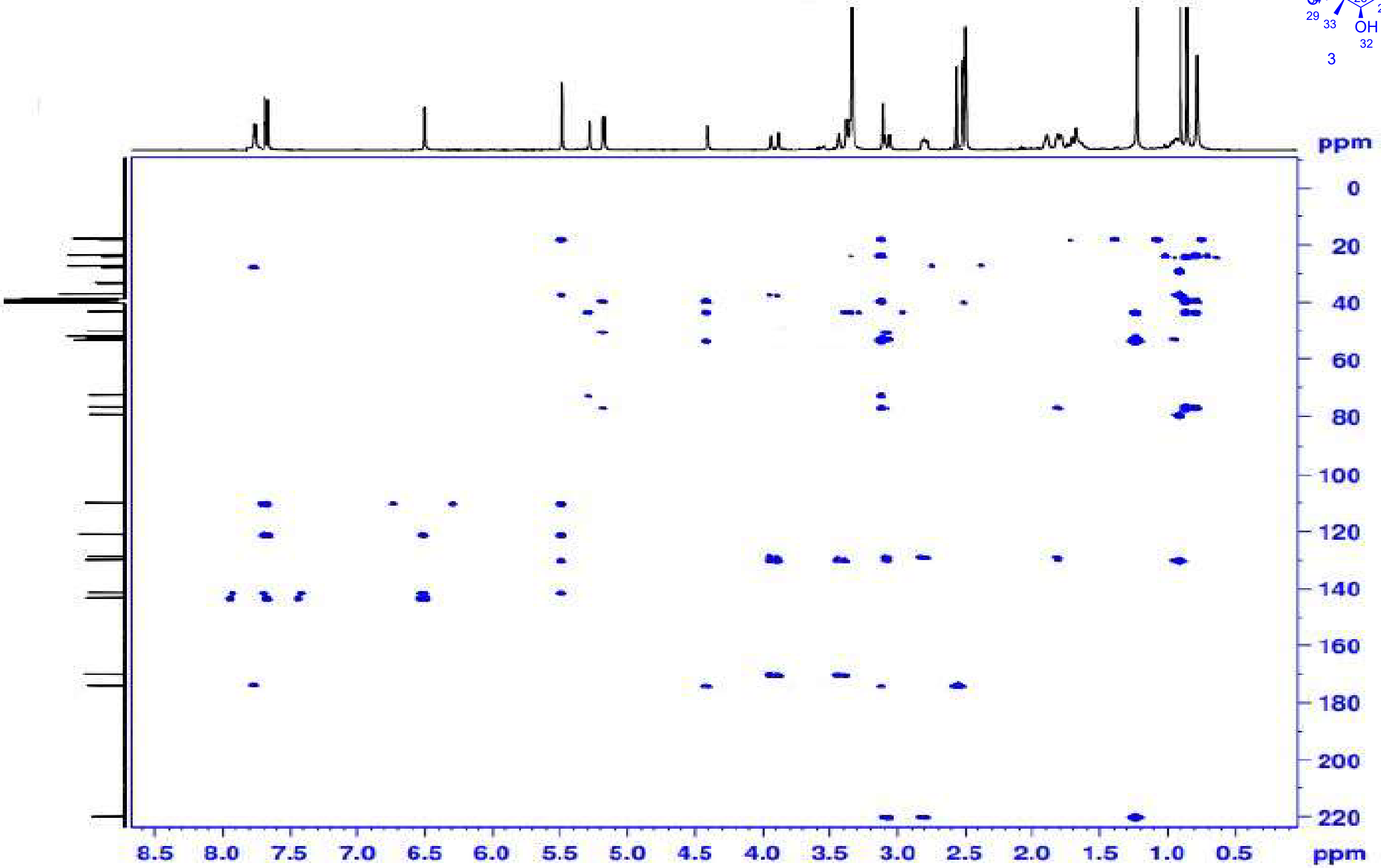

Figure S23. N15 HSQC Spectrum of compound **3** in DMSO- *d6*

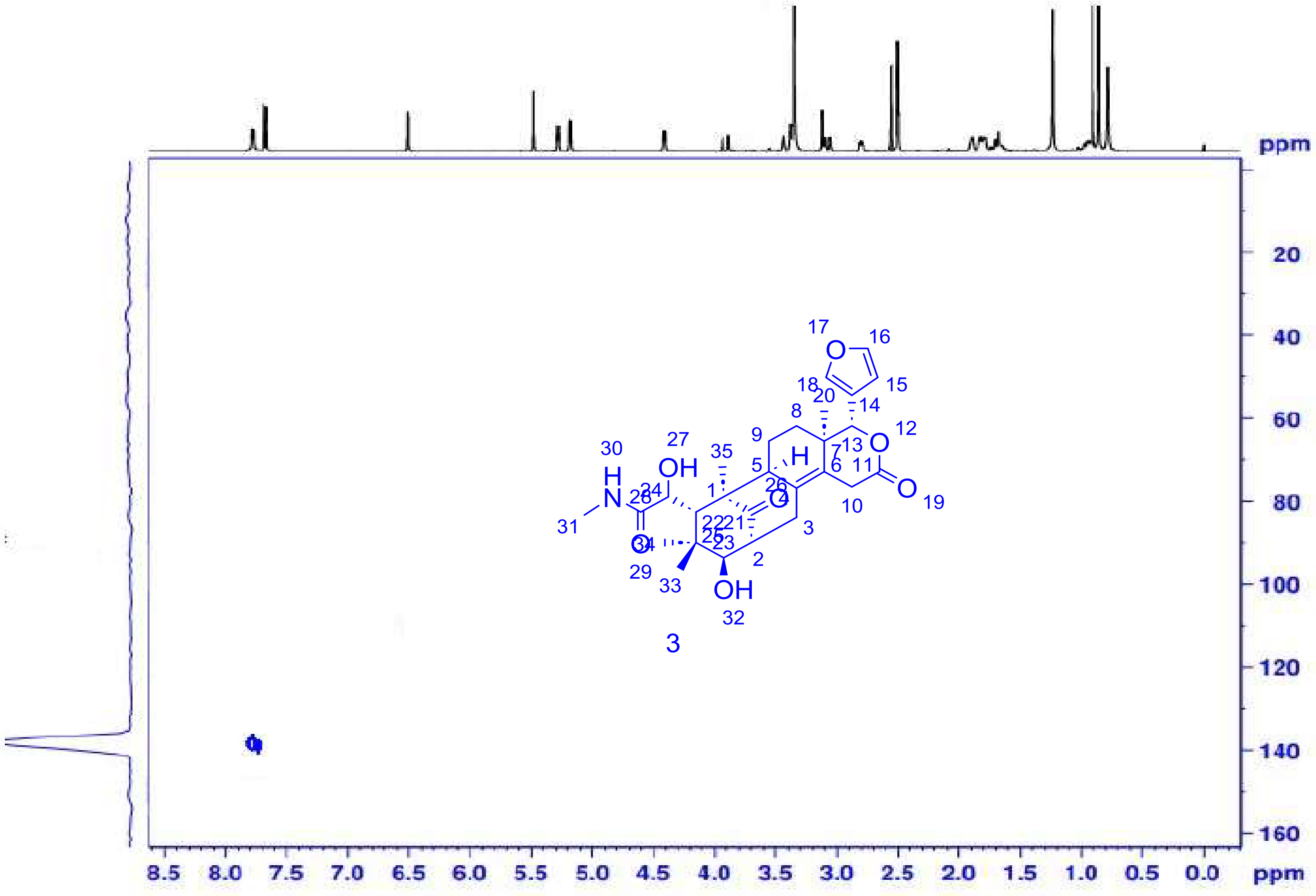

Figure S24. HRESI Mass spectrum of compound **3**

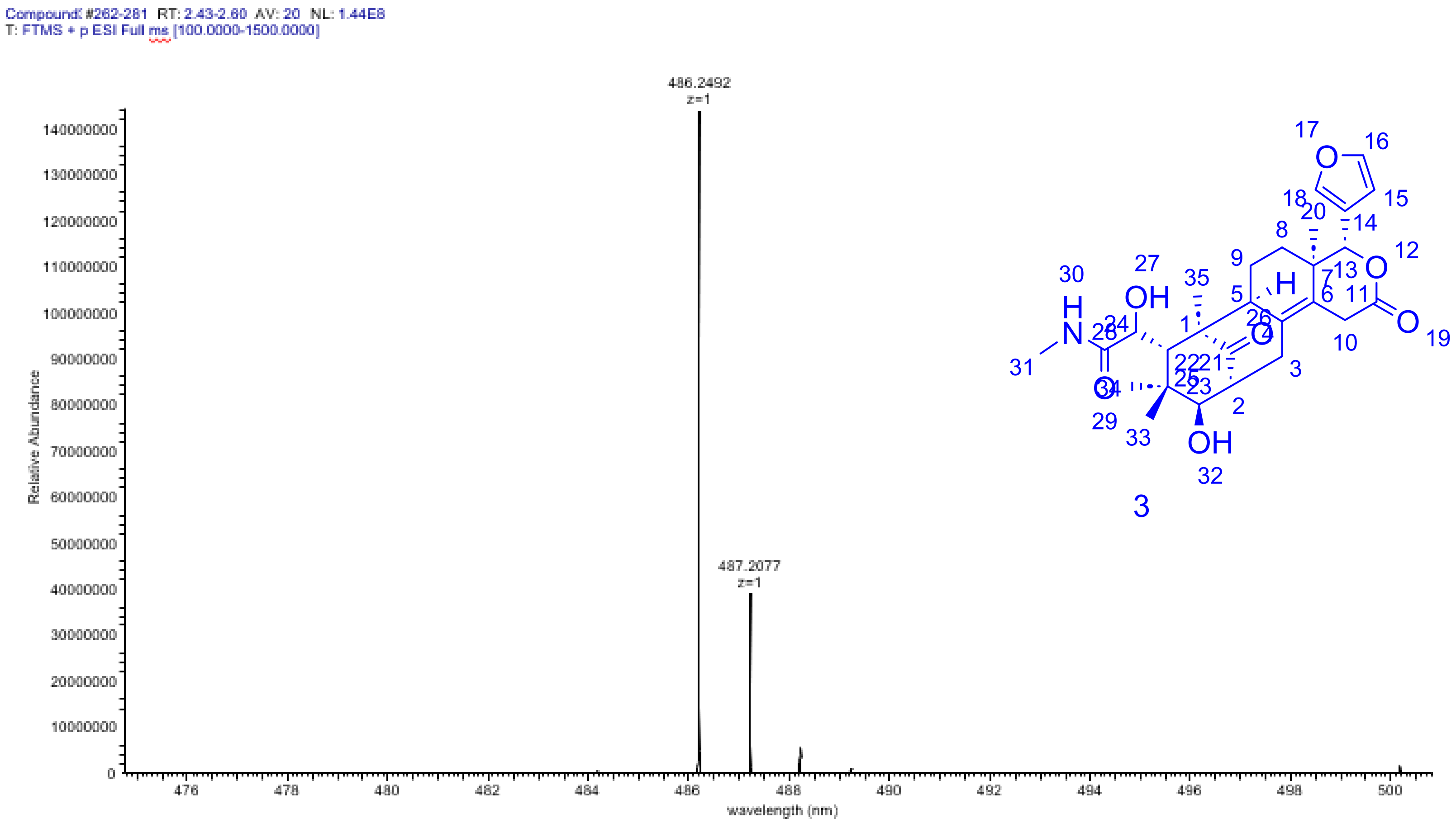

3

Figure S25. IR spectrum of compound **3**

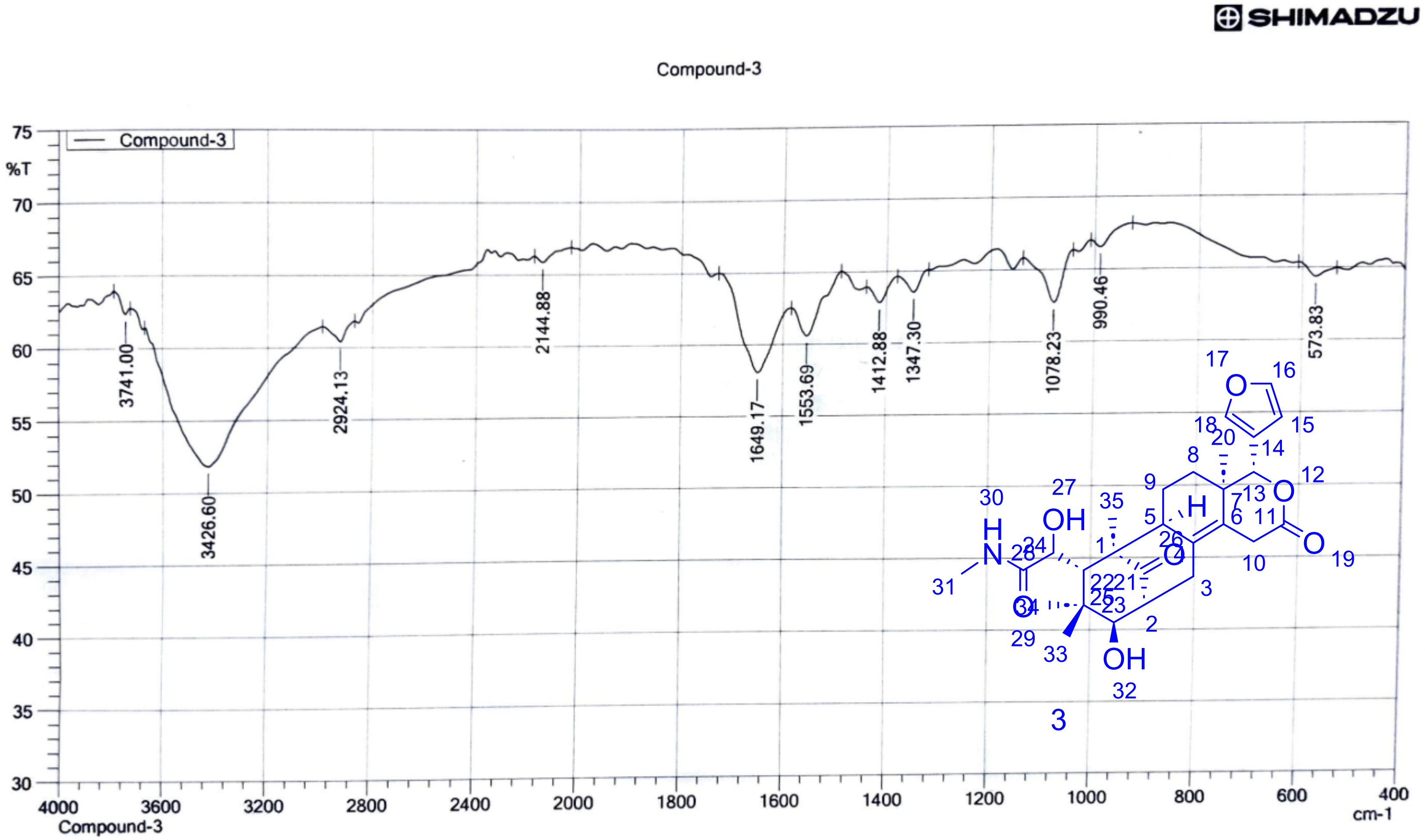

Figure S26. Optical rotation of compound 3

[Measurement Information]
Instrument Name Polarimeter
Model Name P-2000
Serial No. B209561232
Polarizer Dichrom
Faraday Cell Flint Glass

Accessory PTC-262
Accessory S/N C051961481
Temperature 25.00 C
Control Sonsor Holder
Monitor Sensor Holder
Start Mode Keep target temperature +/-0.10 C while 5 seconds
Light Source WI
Monitor wavelength 589 nm
D.I.T. 5 sec
No. of cycle 5
Cycle interval 5 sec
Temp. Monitor Holder
Temp. Corr. Factor 0 at 20 C
Aperture(S) 8.0mm
Aperture(L) Auto
Mode Specific O.R.
Path Length 10 mm
Concentration 0.1 w/v%
Water content of sample 0 %
Factor 1

Comment 0.1% in Methanol
Username
Division
Organization Aragen Life Sciences Pvt Ltd

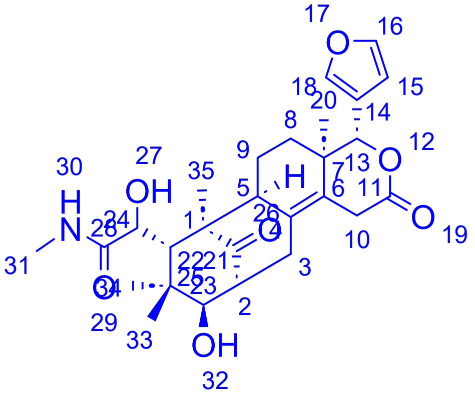

3

		No.	Sample No.	Calc. Data	Meas. Data	Temperature(C)	Blank	Comment
1	*	1	Compound-3-1	-125.2000	-0.0125	25.00	+0.0064	0.1% in Methanol
2	*	2	Compound-3-2	-123.2000	-0.0123	25.00	+0.0064	0.1% in Methanol
3	*	3	Compound-3-3	-123.2000	-0.0123	25.00	+0.0064	0.1% in Methanol
4	*	4	Compound-3-4	-128.2000	-0.0128	24.99	+0.0064	0.1% in Methanol
5	*	5	Compound-3-5	-129.2000	-0.0129	24.99	+0.0064	0.1% in Methanol
6	*	6	Avg.	-125.8000				
7		7	S.D	2.7928				
8		8	C.V	2.2201				

Figure S27. UV spectrum of compound **3**

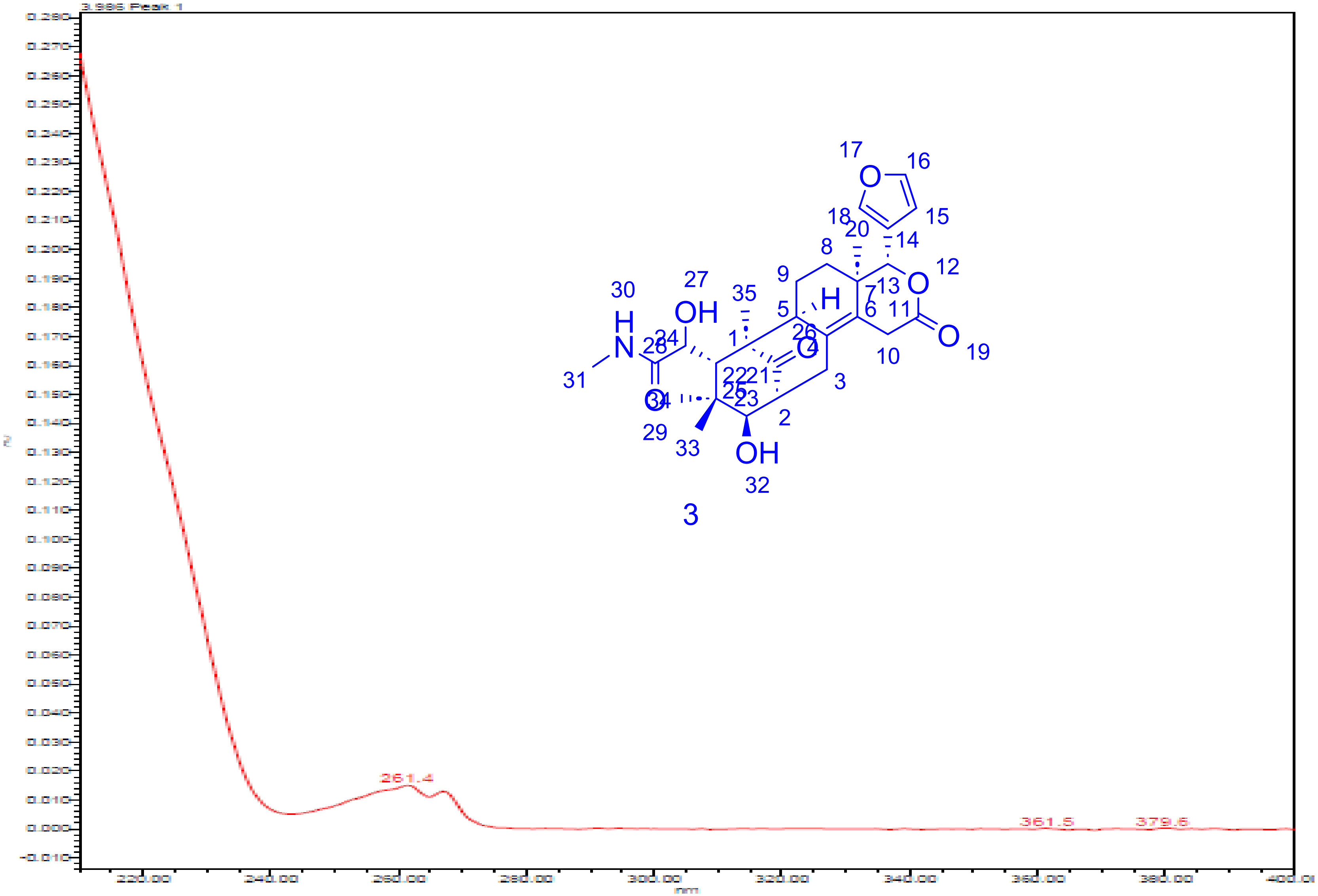

Figure S28. ^{1}H NMR Spectrum of compound **4** at (400 MHz) in DMSO- *d6*

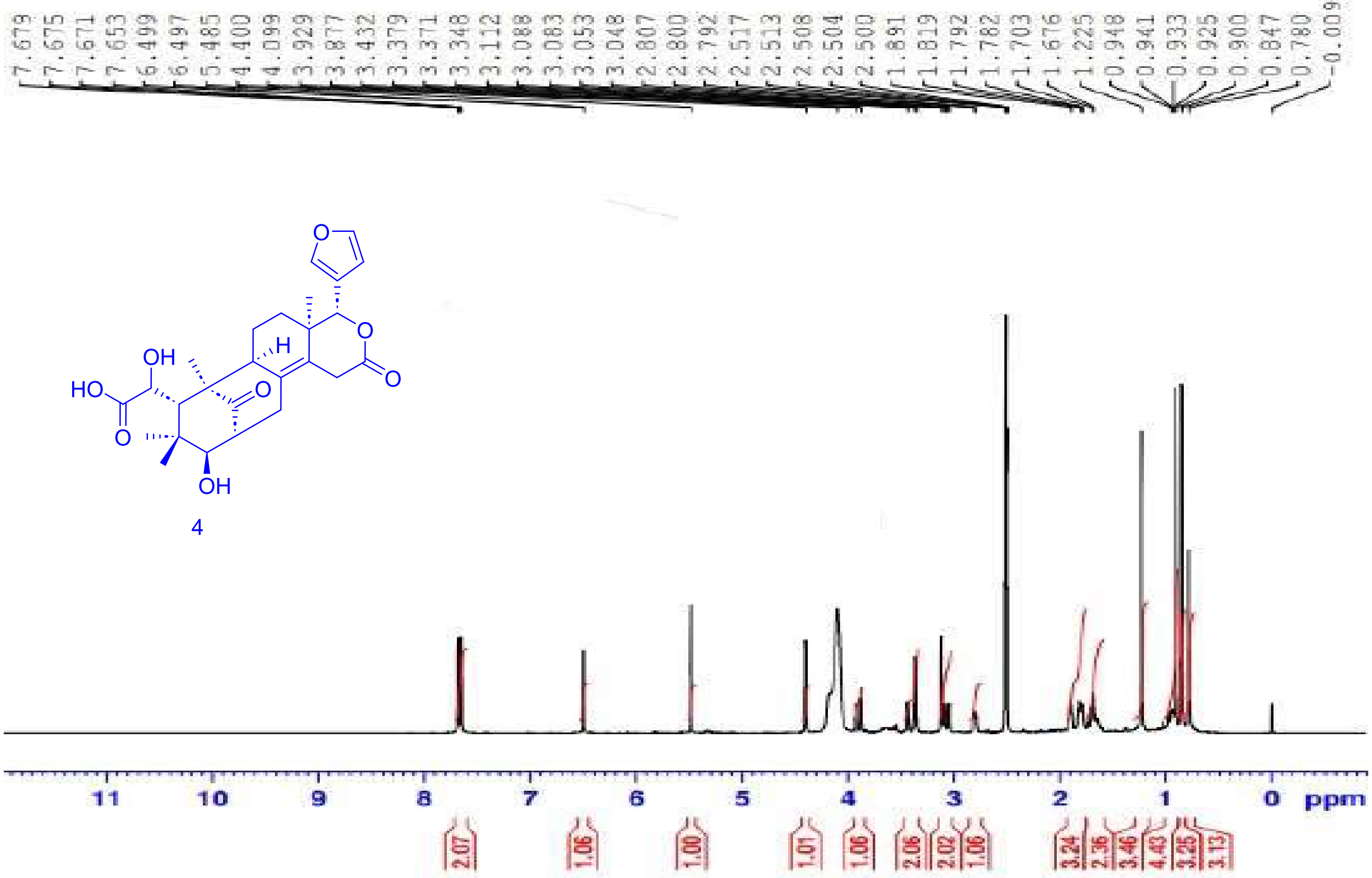

Figure S29. ^{13}C NMR Spectrum of compound **4** at (400 MHz) in DMSO- d6

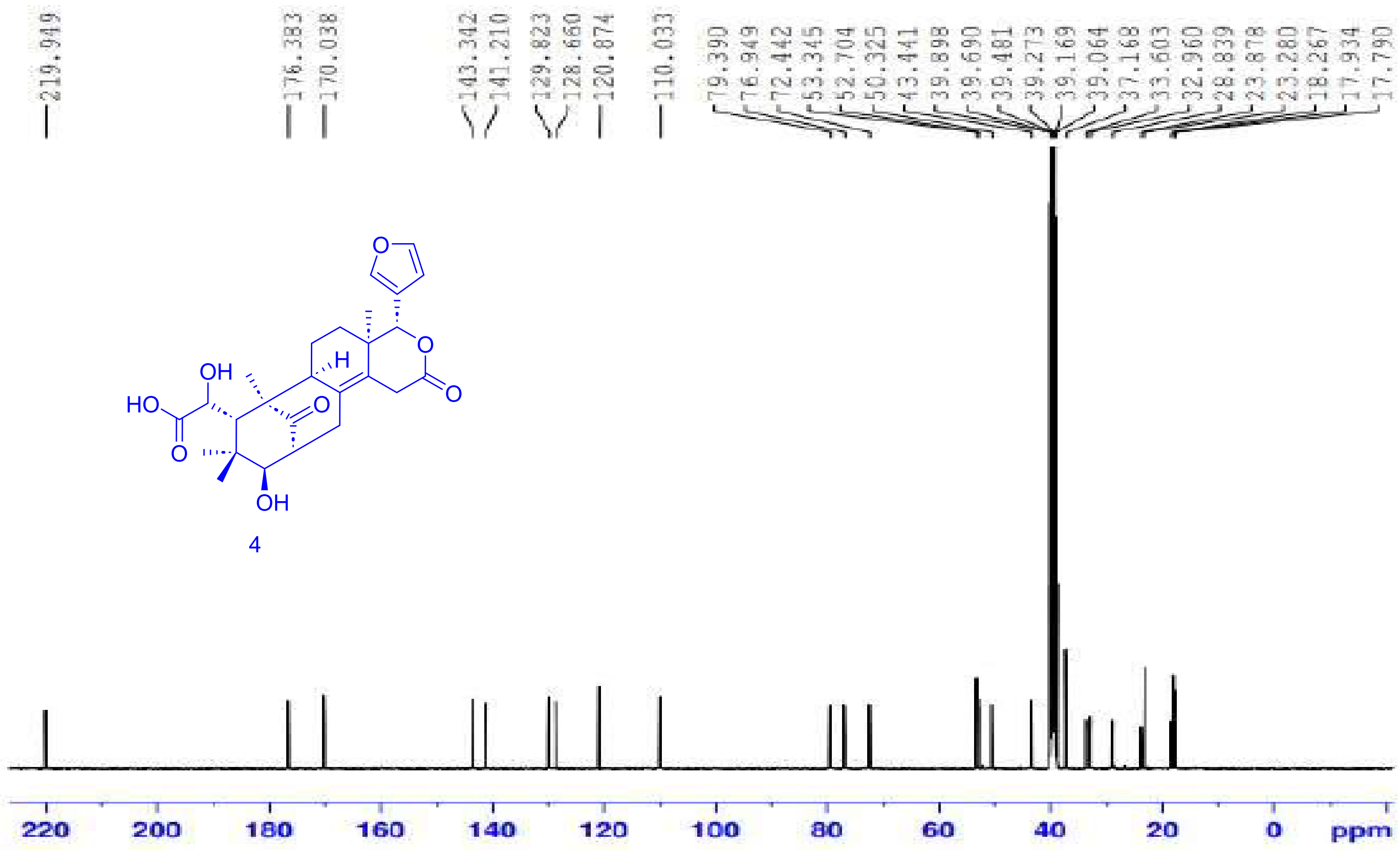

Figure S30. HSQC Spectrum of compound **4** in DMSO- *d6*

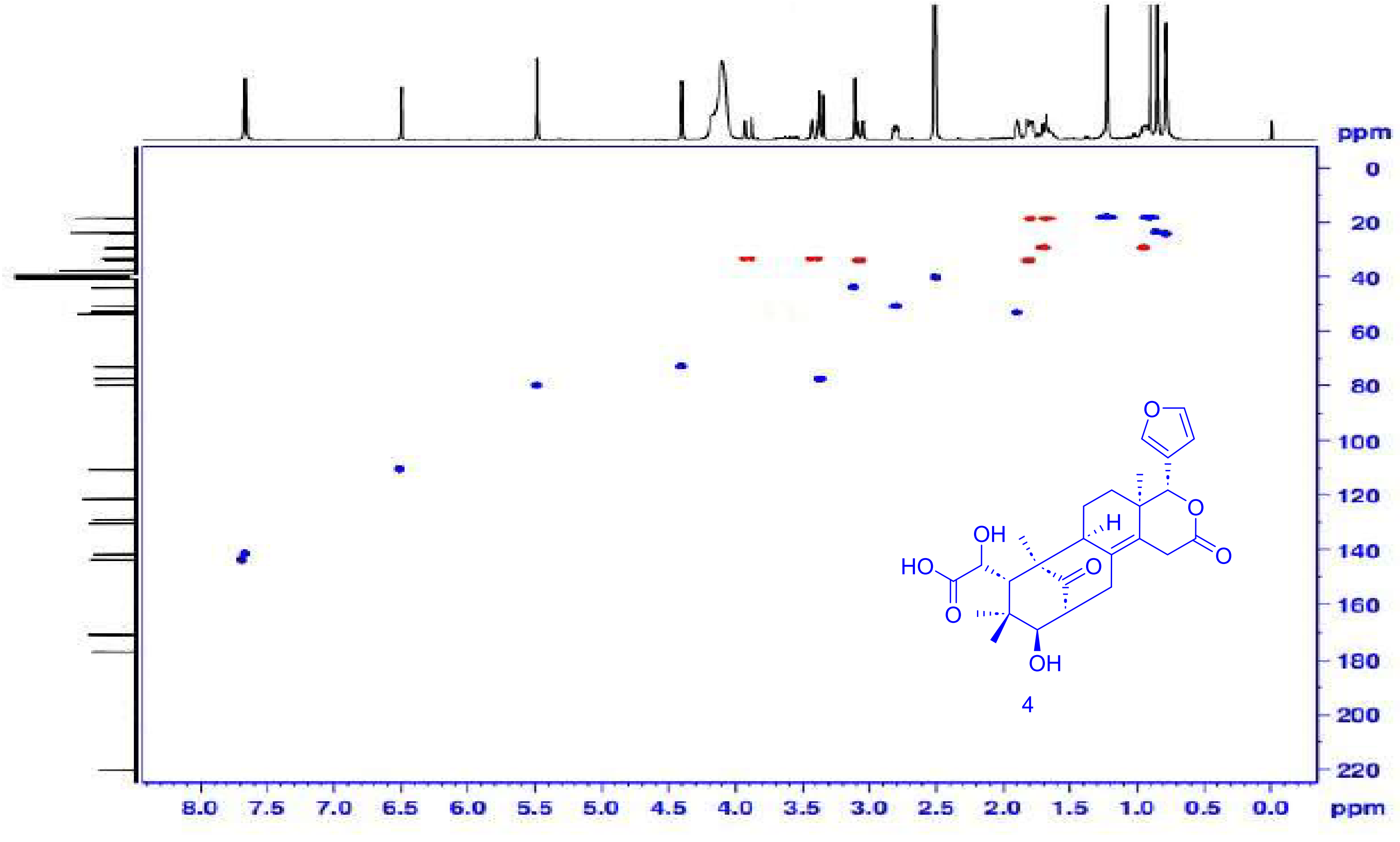

Figure S31. HMBC Spectrum of compound **4** in DMSO- d6

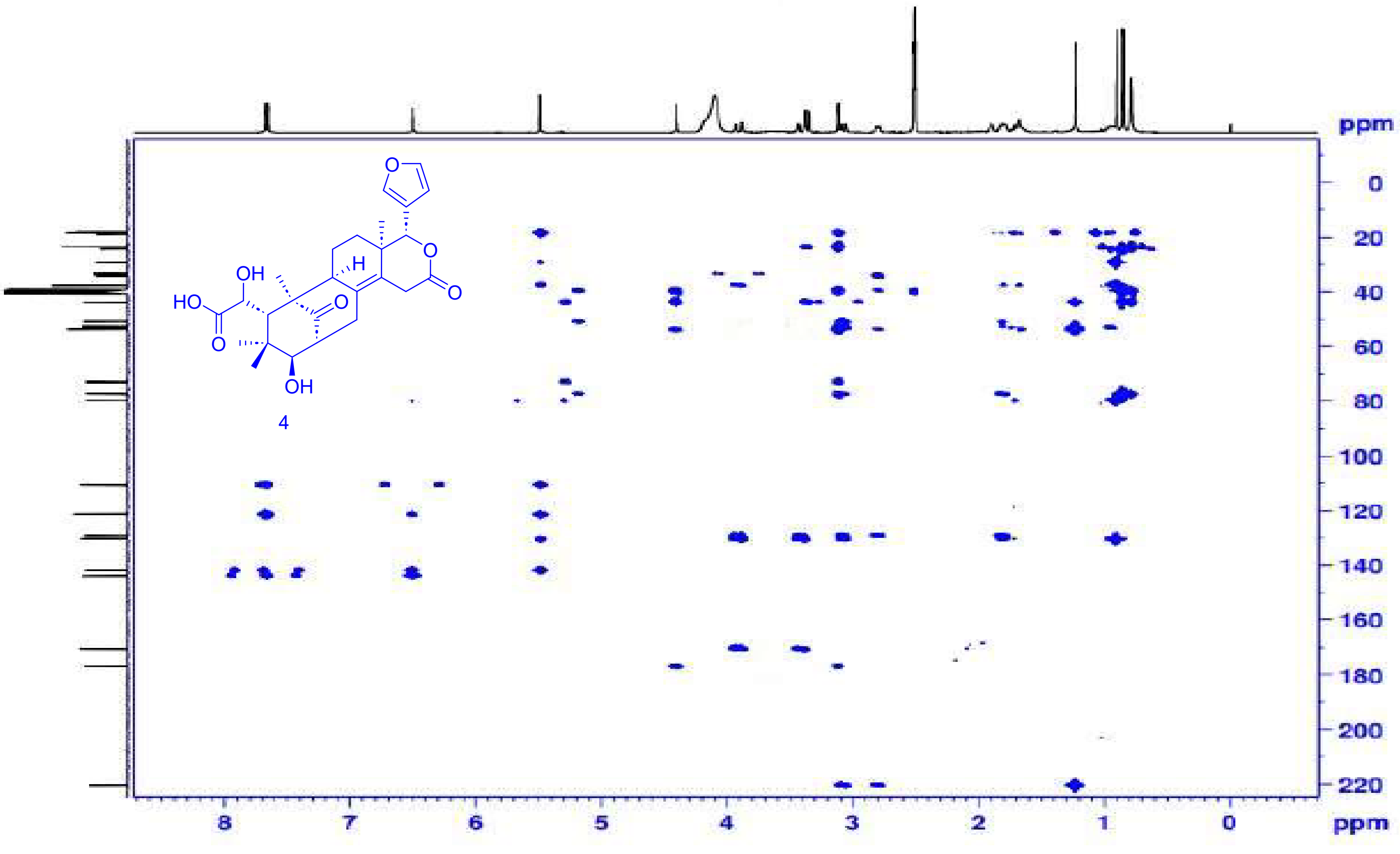

Figure S32. HRESI Spectrum of compound **4**

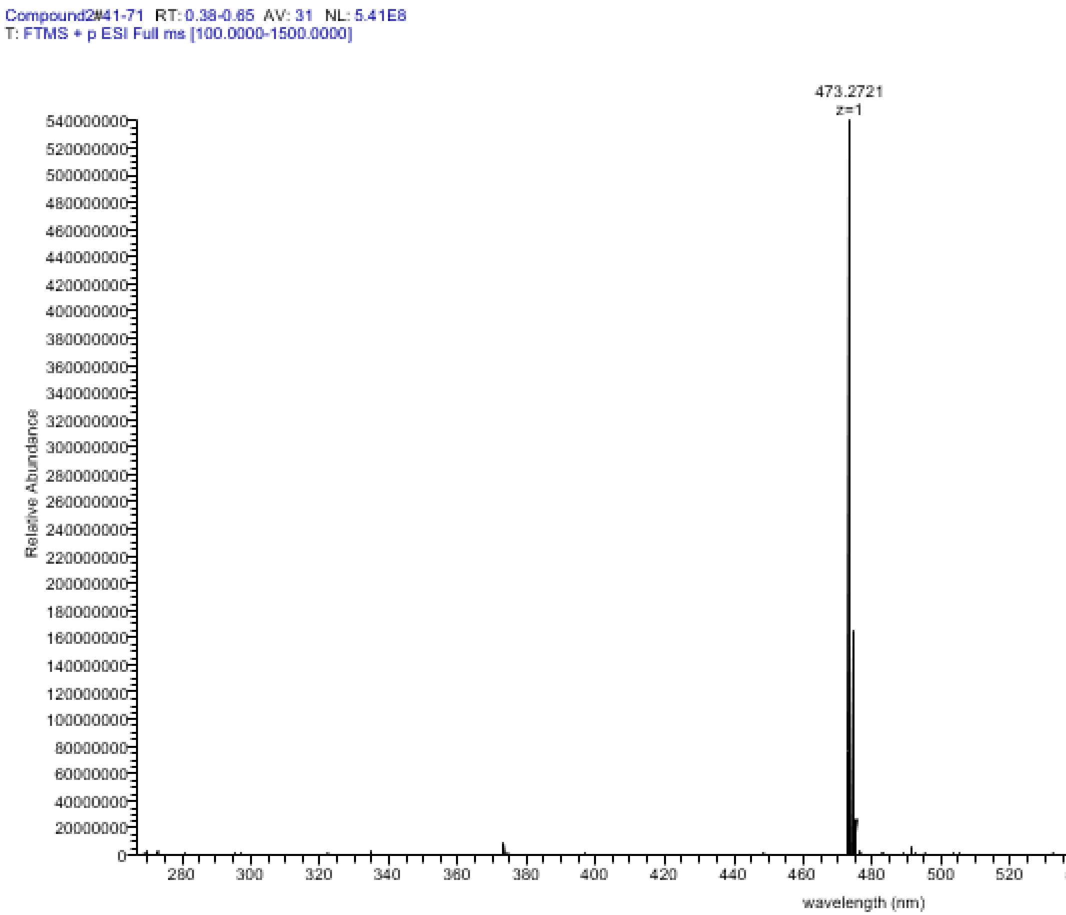

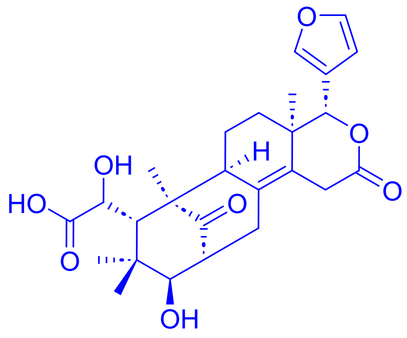

Figure S33. ^{1}H NMR Spectrum of compound **5** at (400 MHz) in DMSO- *d6*

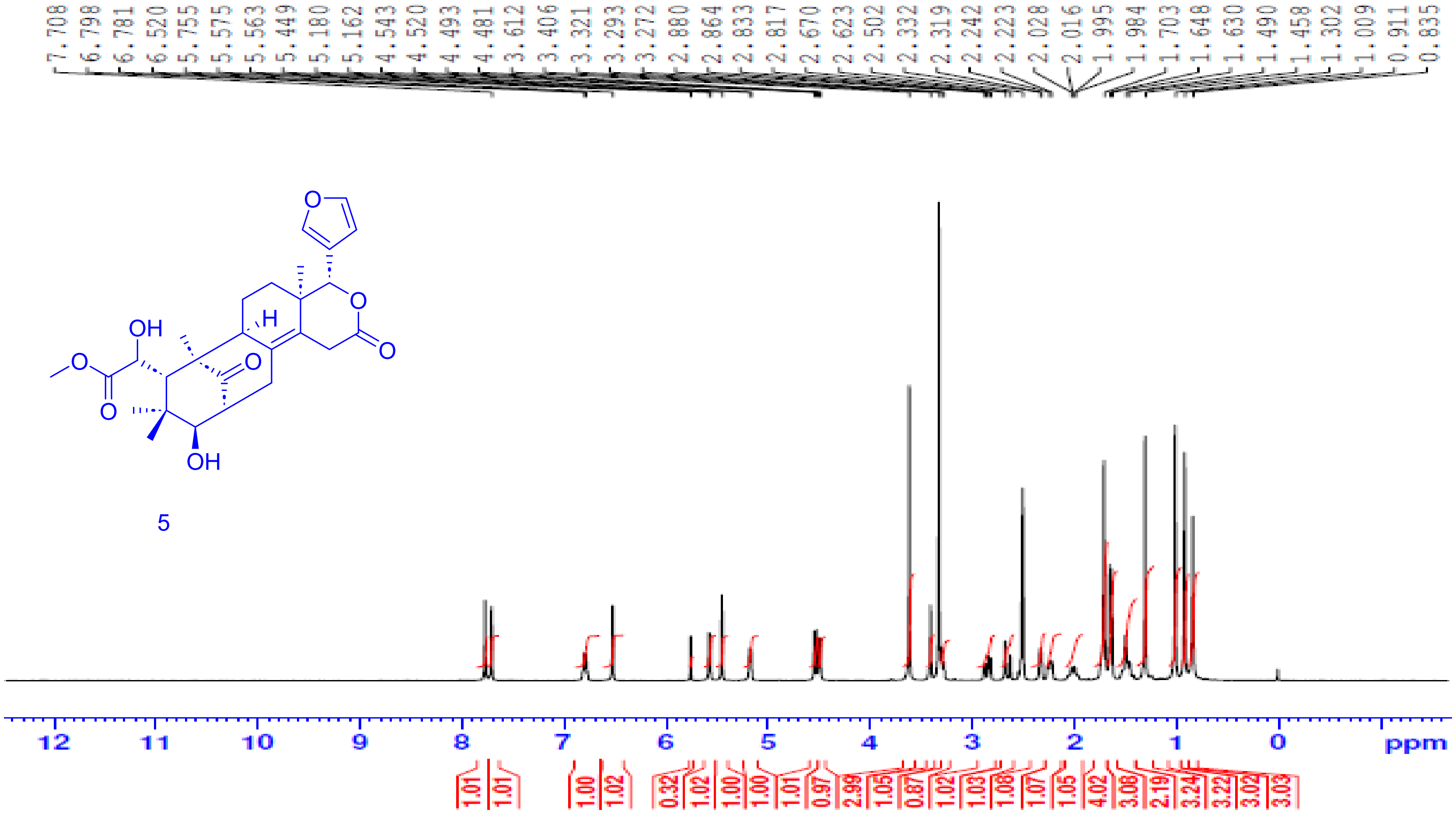

Figure S34. ^{13} CNMR Spectrum of compound **5** at (400 MHz) in DMSO- *d6*

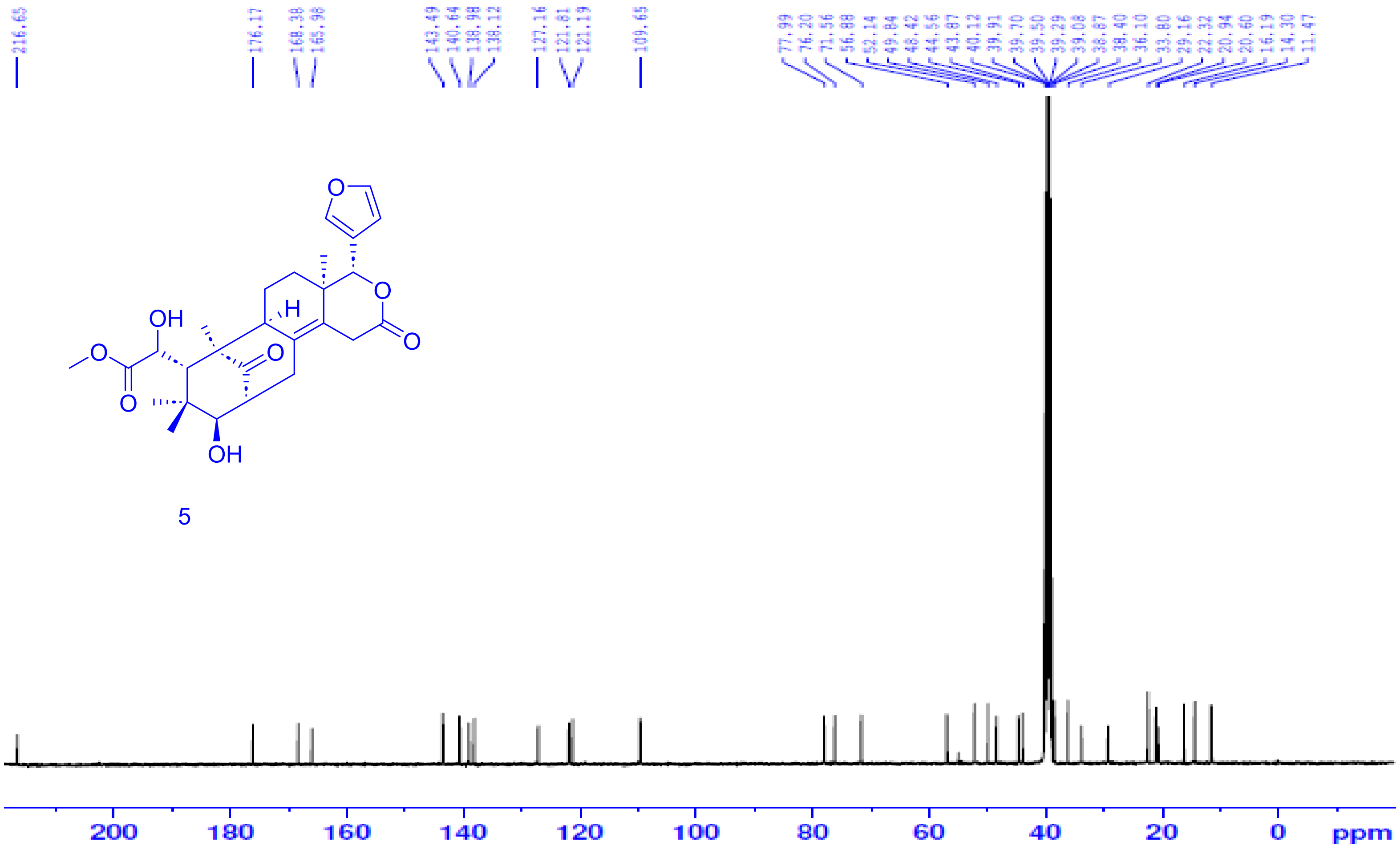

Figure S35. HSQC Spectrum of compound **5** in DMSO- *d6*

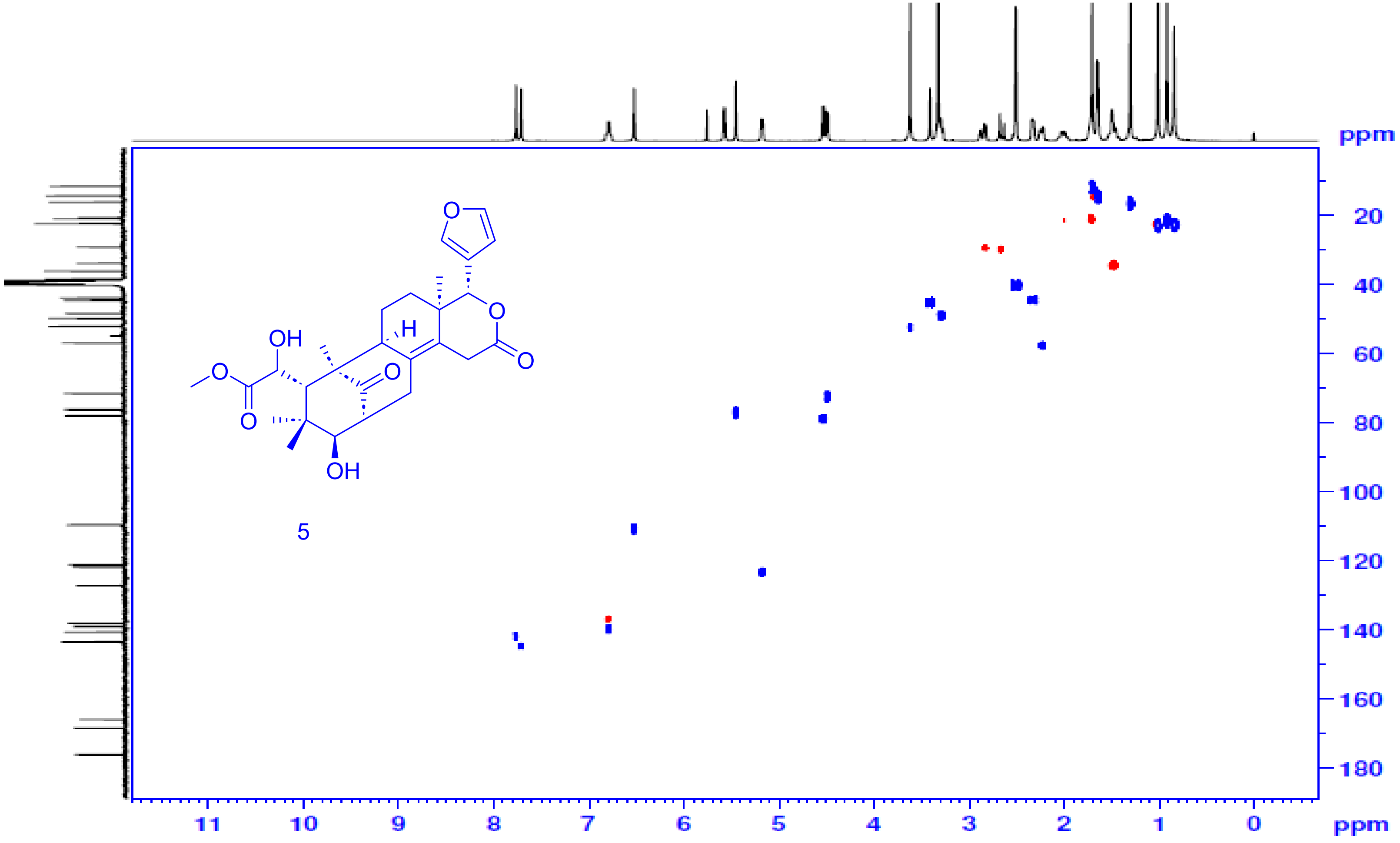

Figure S36. HMBC Spectrum of compound **5** in DMSO- *d6*

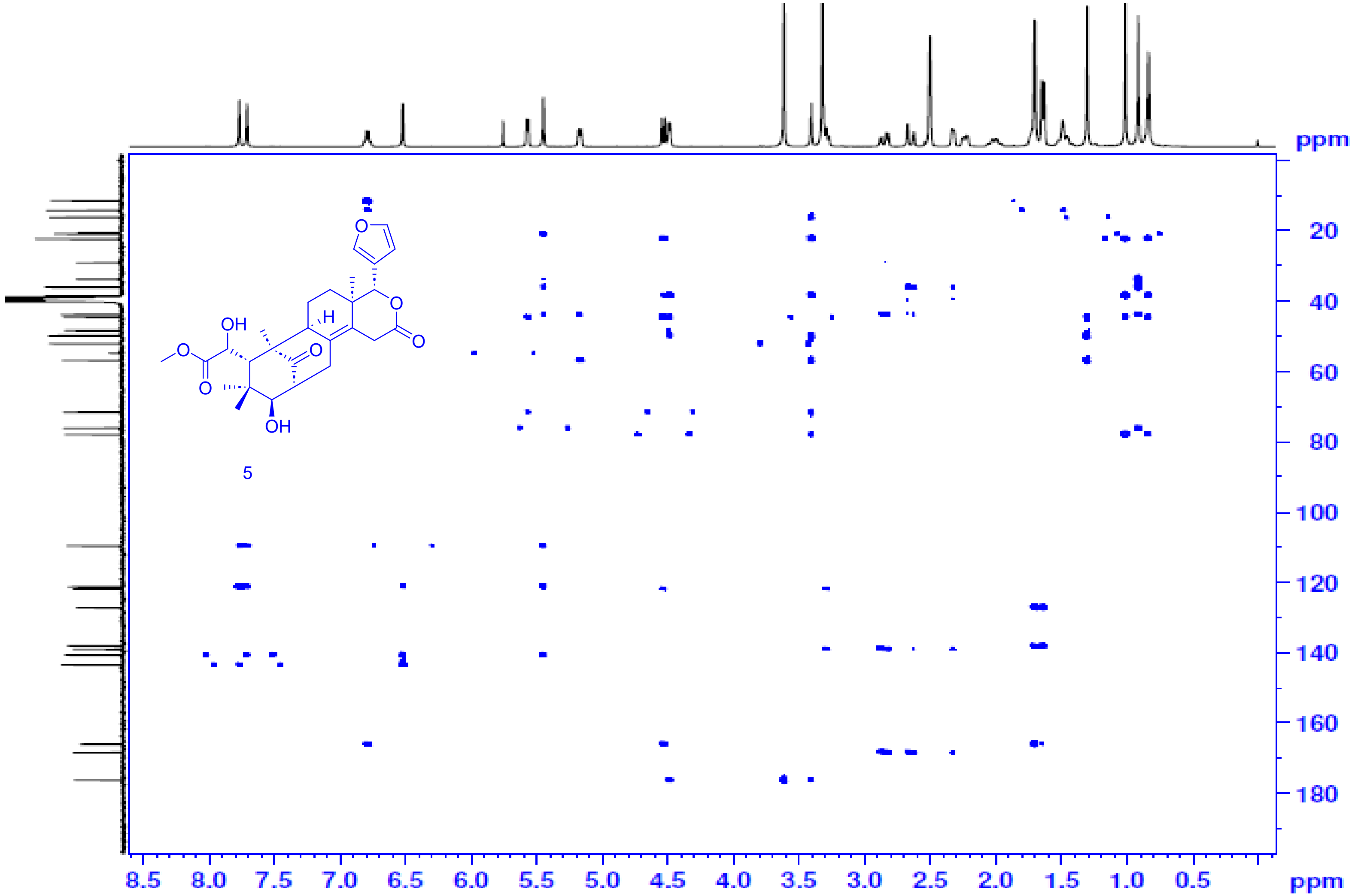

Figure S37. HRESI Mass Spectrum of compound **5**

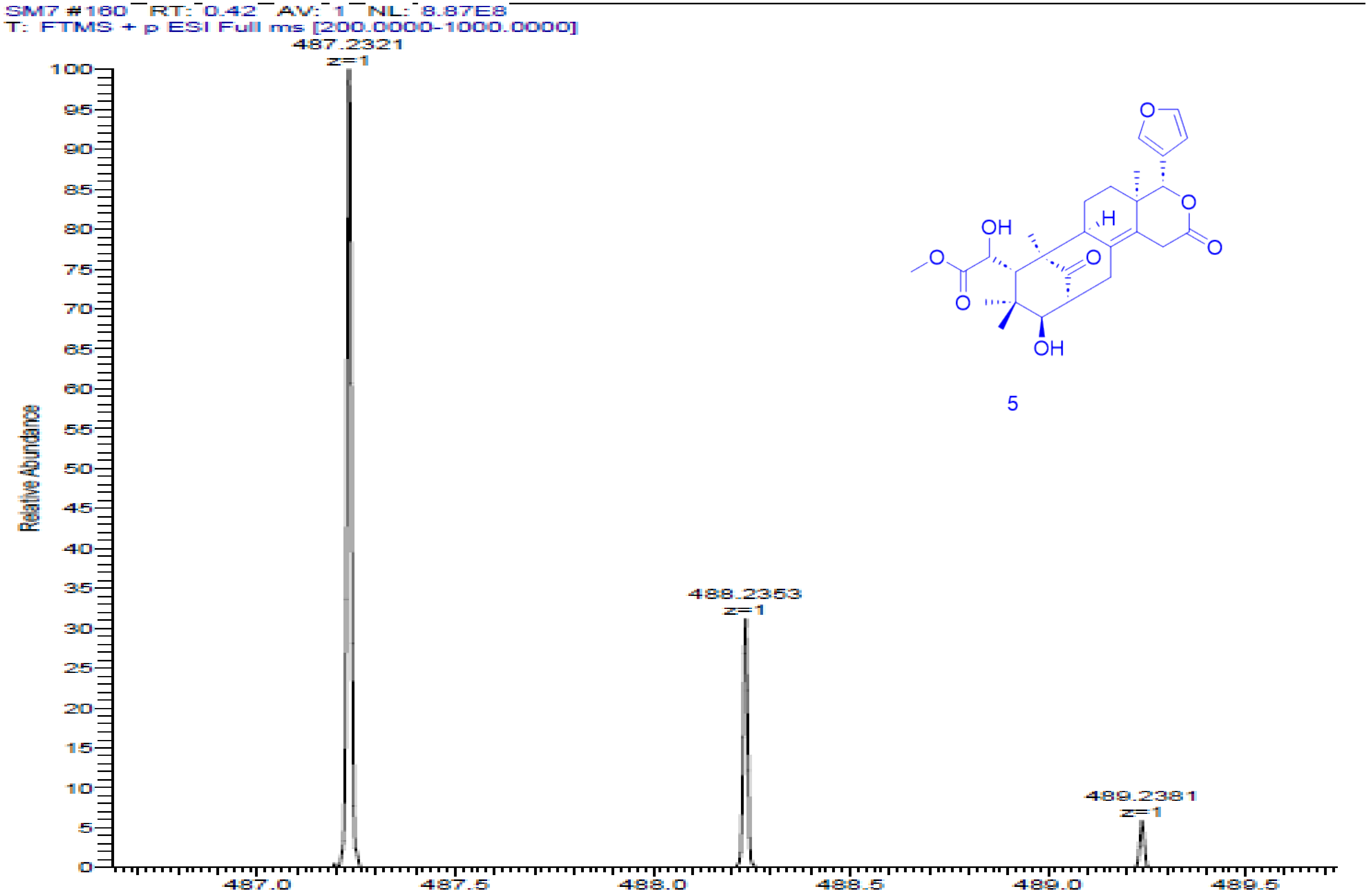

Figure S38. [1] H NMR Spectrum of compound **6** at (400 MHz) in DMSO- *d6*

4-(furan-3-yl)-8-(1-hydroxy-2-methoxy-2-oxoethyl)-4a,7,9,9-tetramethyl-2,13-dioxo-1,4,4a,5,6,6a,7,8,9,10,11,12b-dodecahydro-2H-7,11-methanocycloocta[f]isochromen-10-yl (E)-2-methylbut-2-enoate

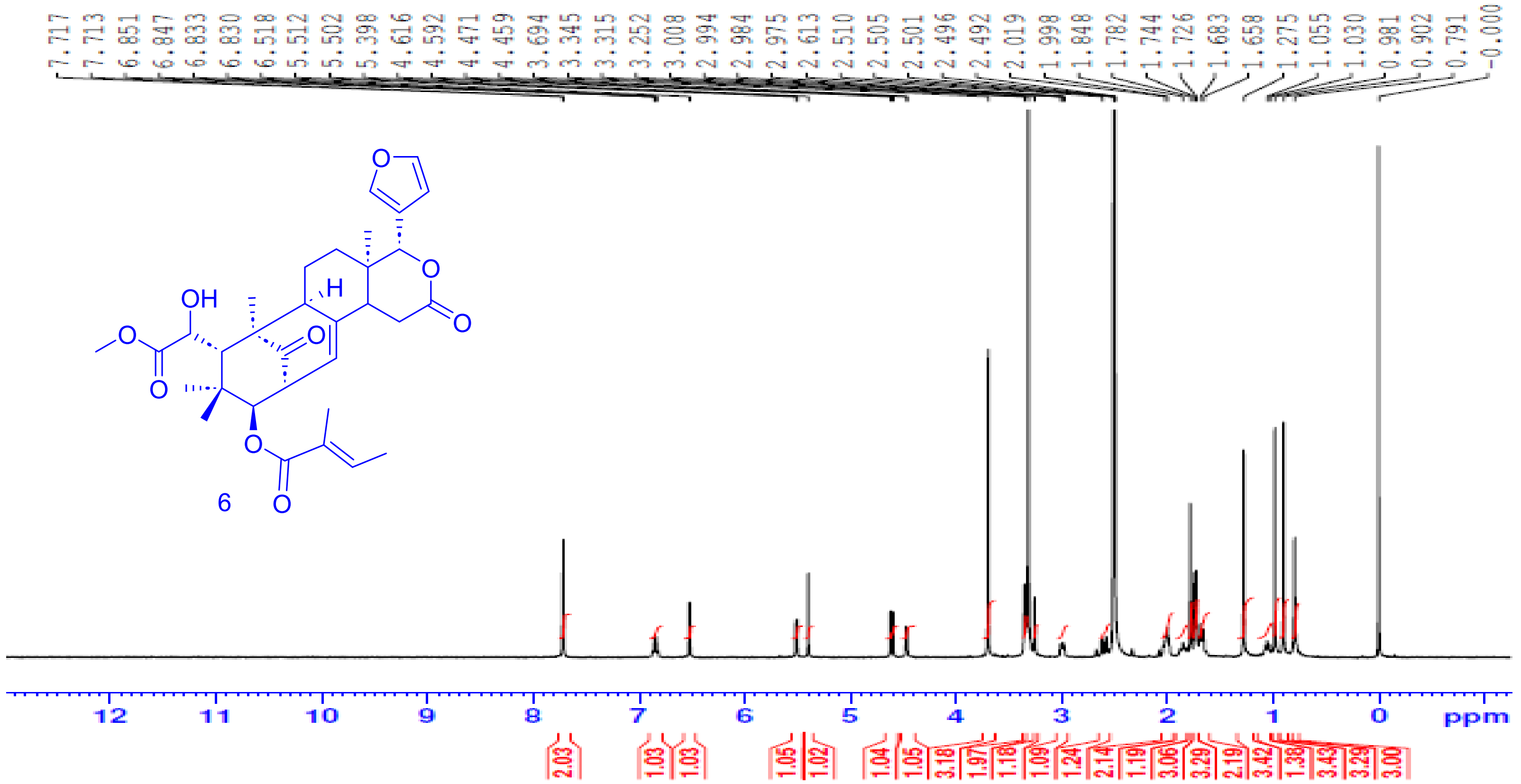

Figure S39. ^{13}C NMR Spectrum of compound **6** at (400 MHz) in DMSO- *d6*

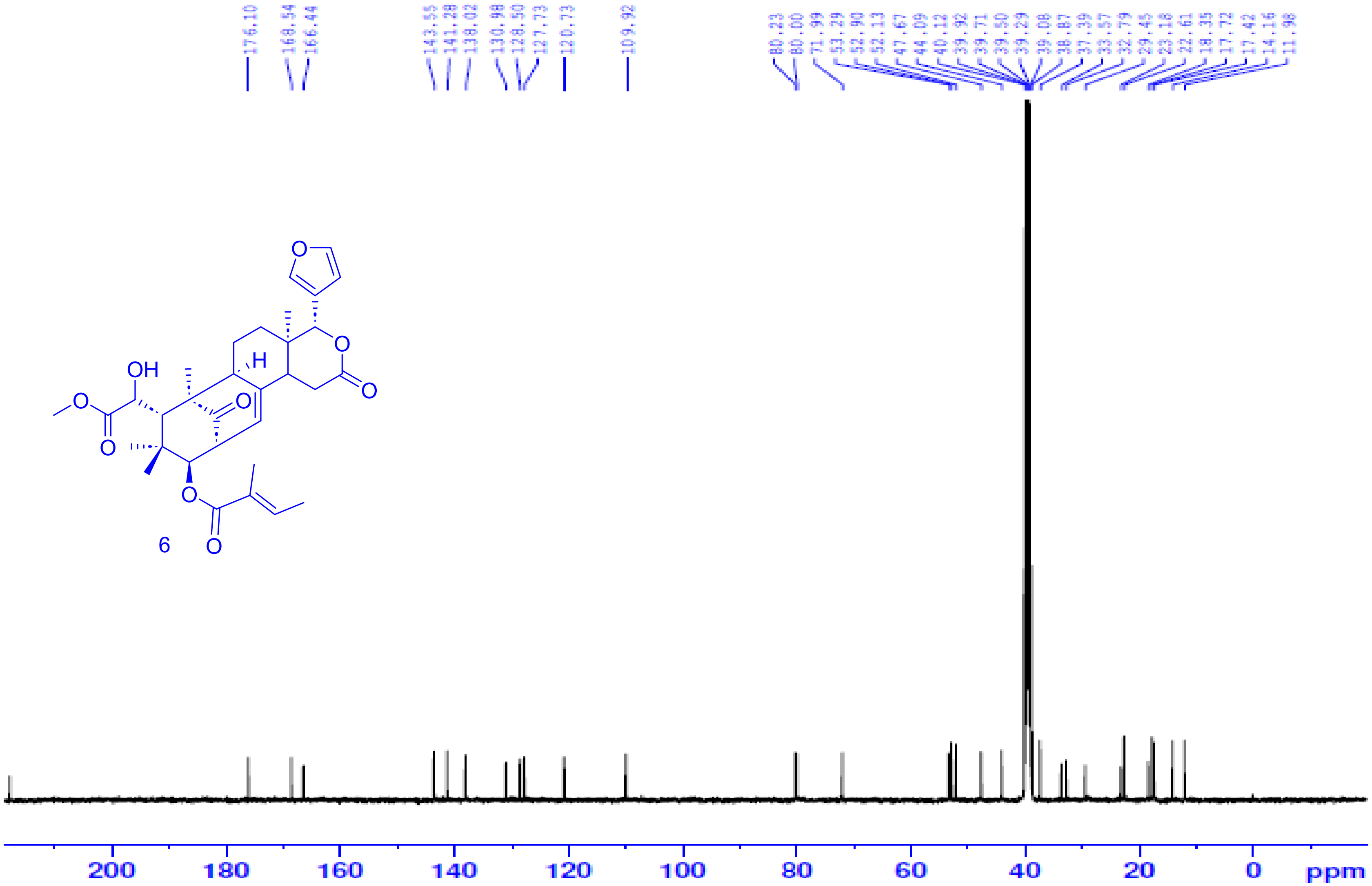

Figure S40. HSQC Spectrum of compound **6** in DMSO- *d6*

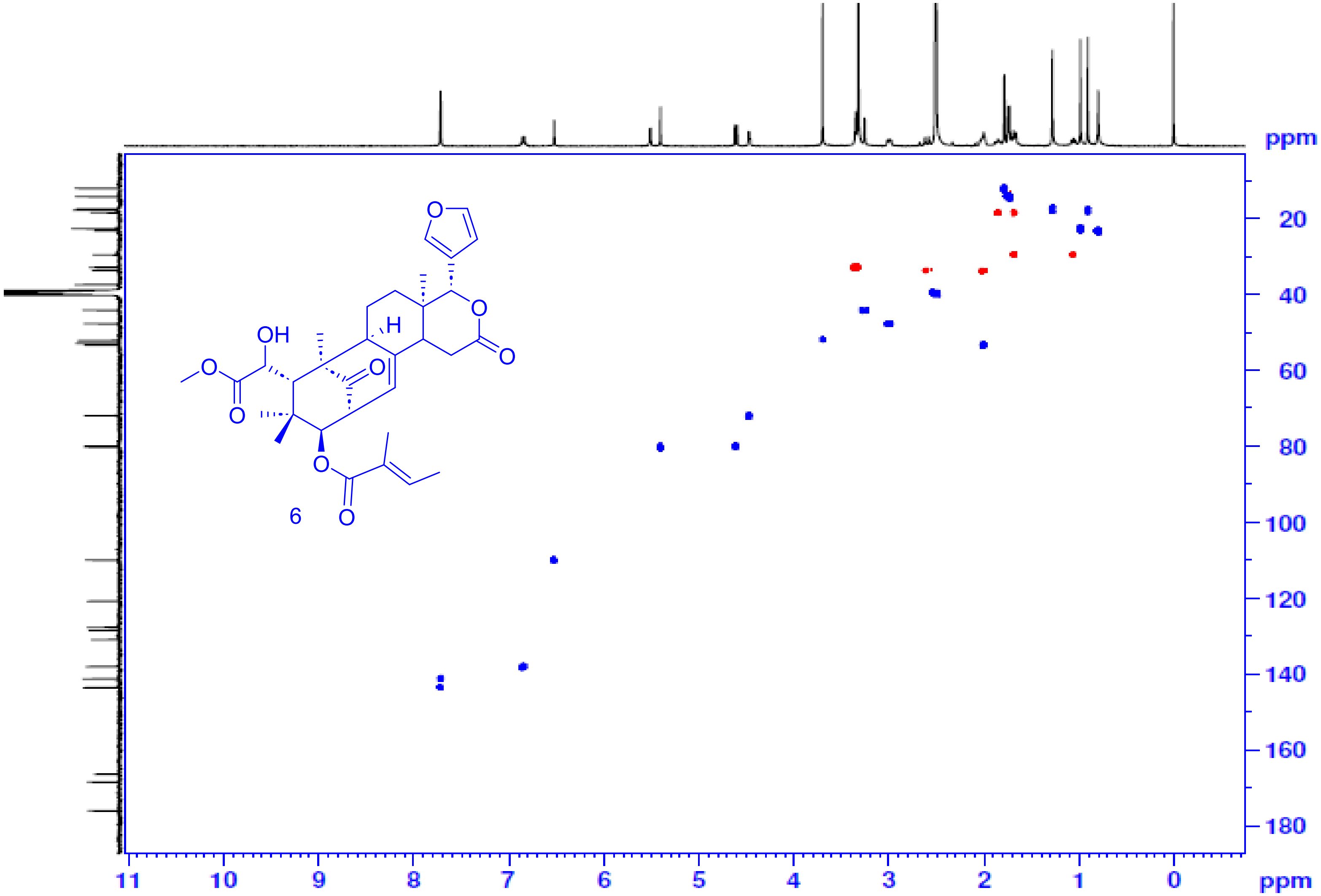

Figure S41. HMBC Spectrum of compound **6** in DMSO- *d6*

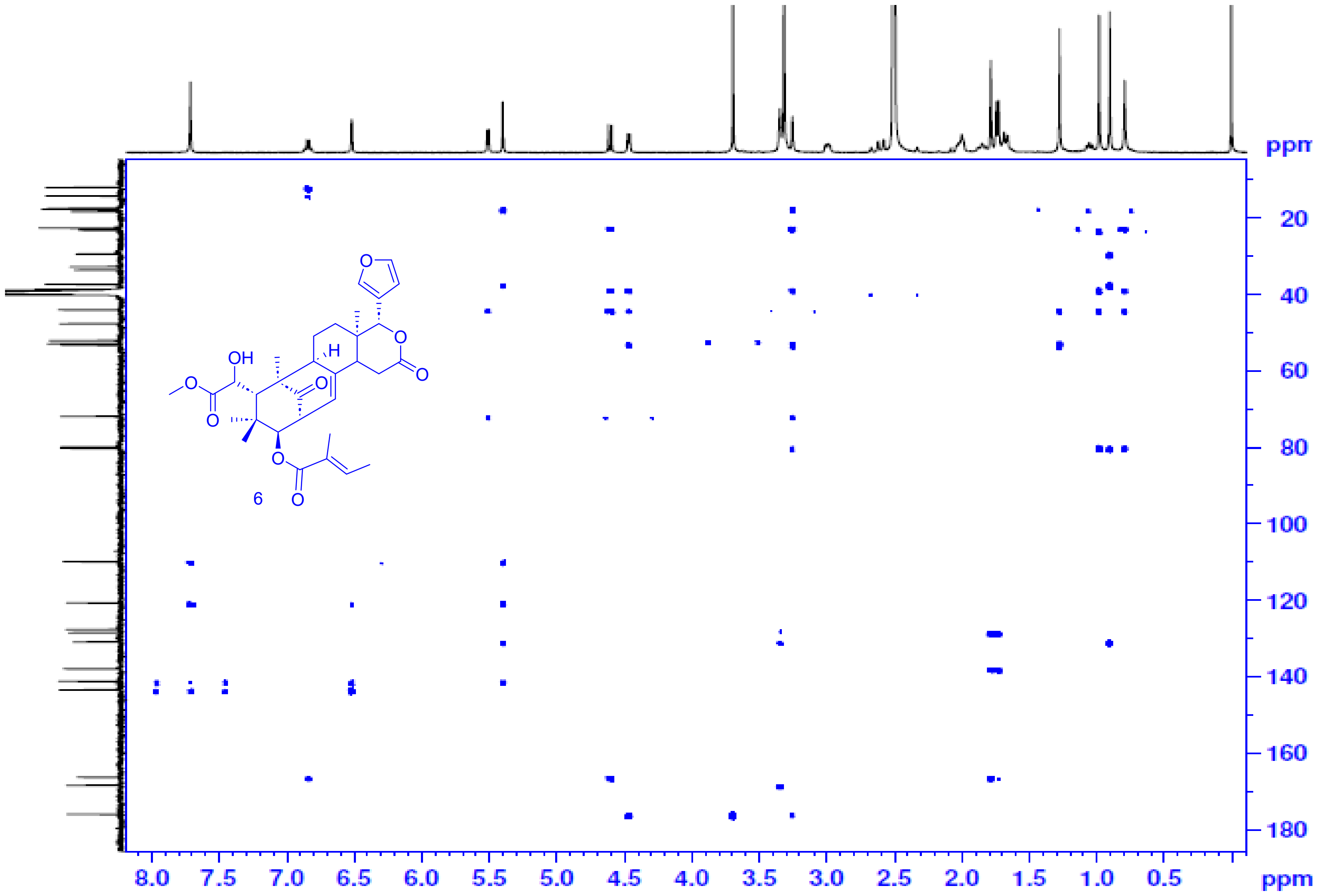

Figure S42. LCESI Mass Spectrum of compound **6**

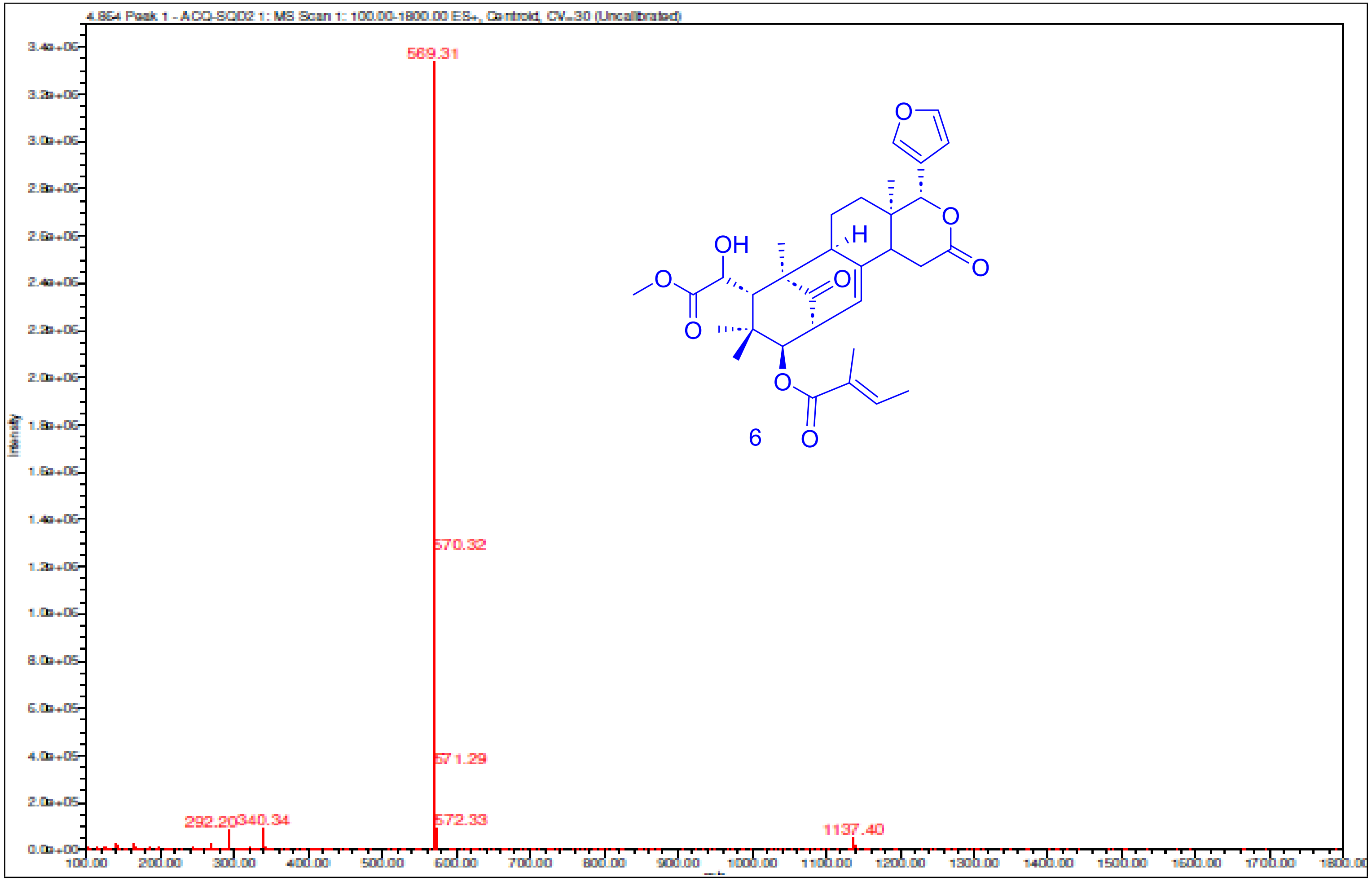

Figure S43. [1] H NMR Spectrum of compound **7** at (400 MHz) in DMSO- *d6*

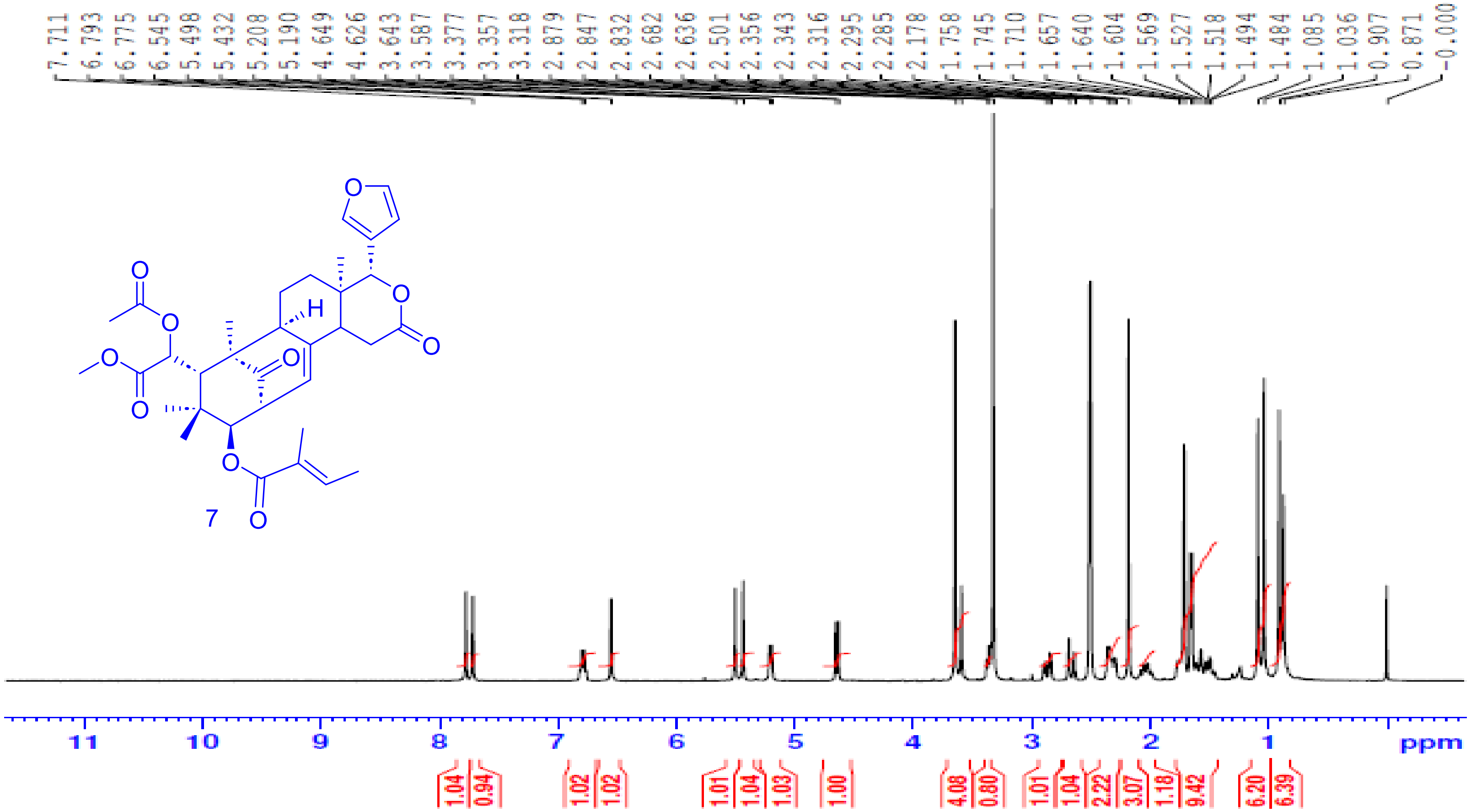

Figure S44. ^{13}C NMR Spectrum of compound **7** at (400 MHz) in DMSO- *d6*

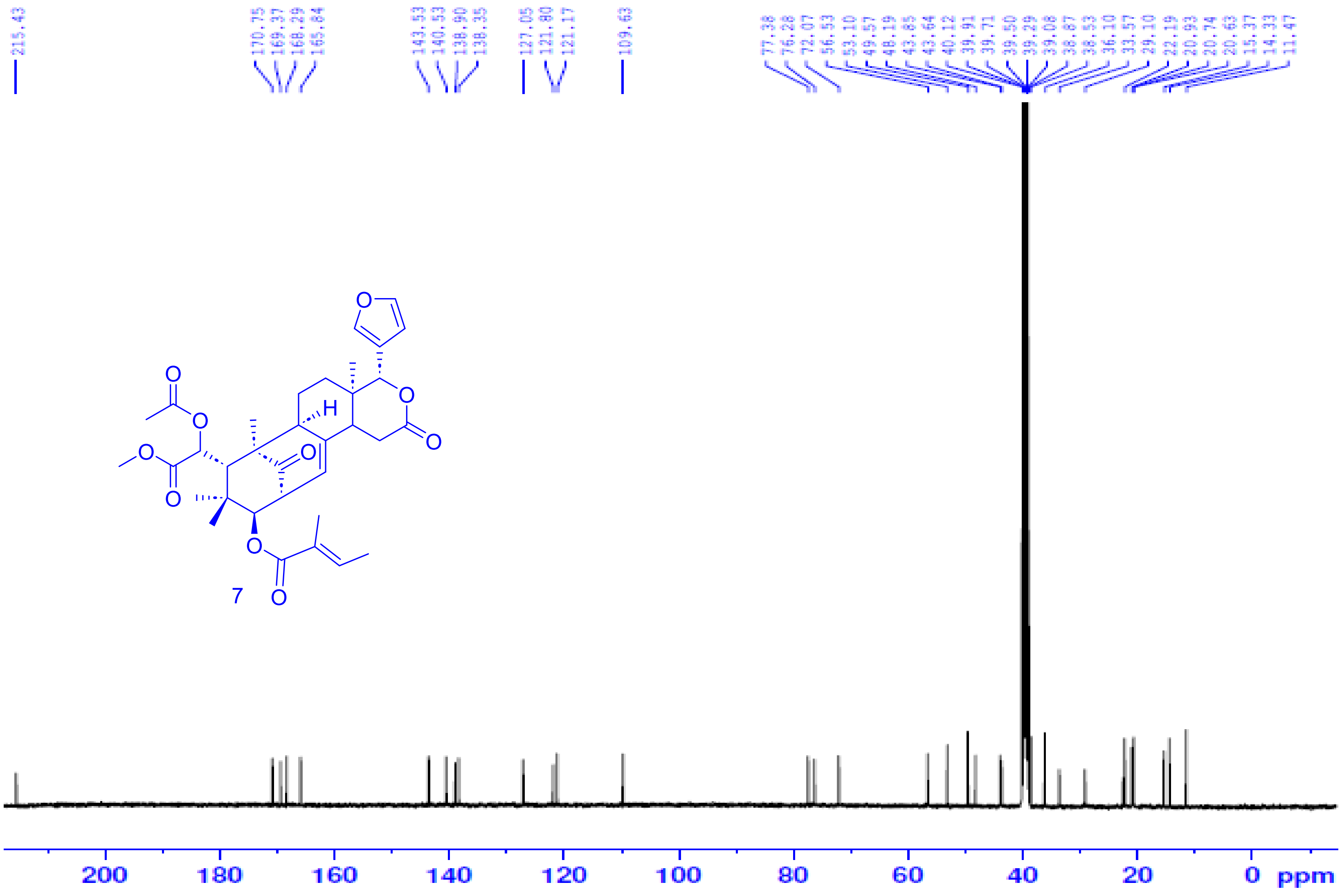

Figure S45. HSQC Spectrum of compound **7** in DMSO- *d6*

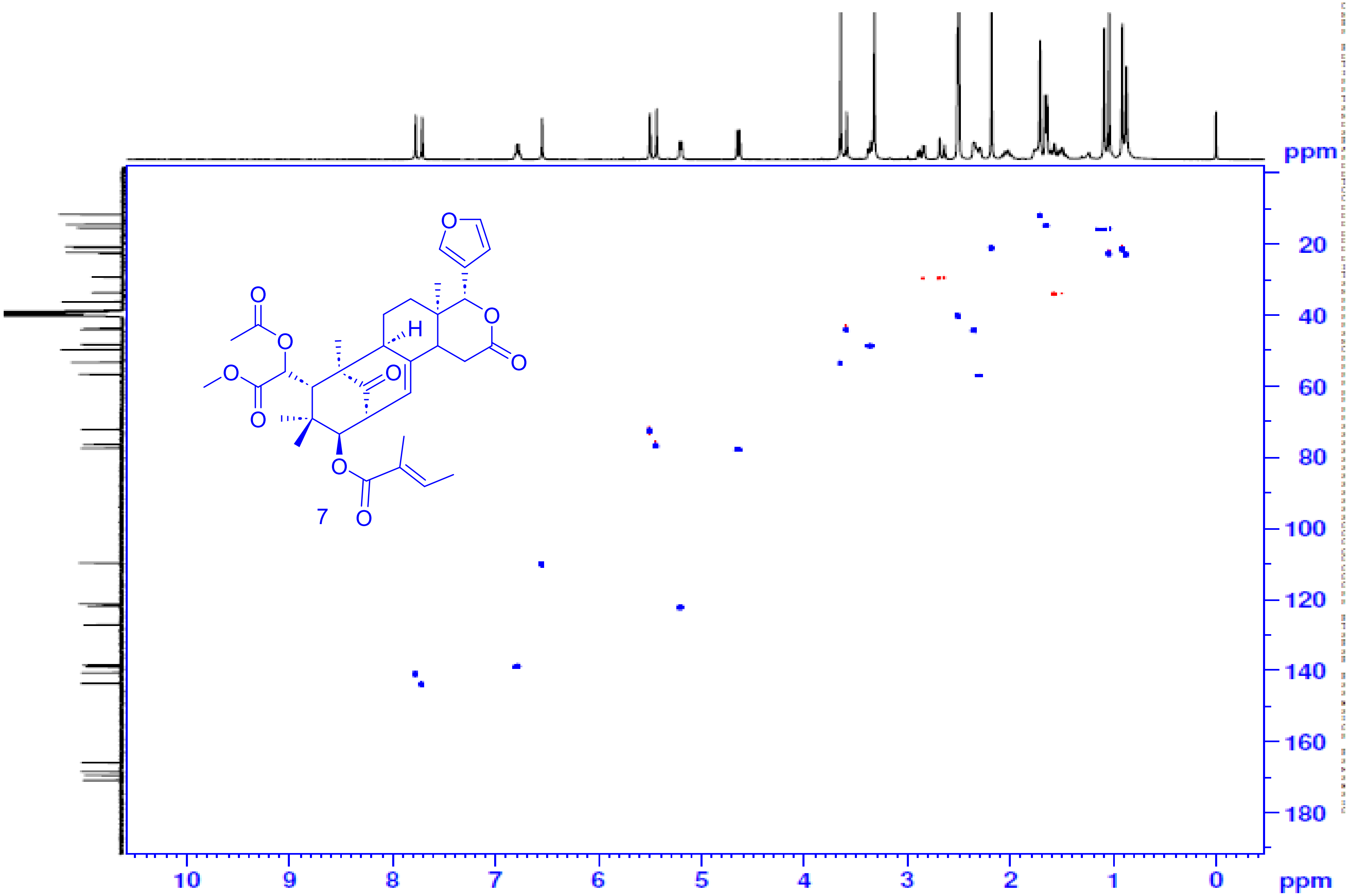

Figure S46. HMBC Spectrum of compound **7** in DMSO- *d6*

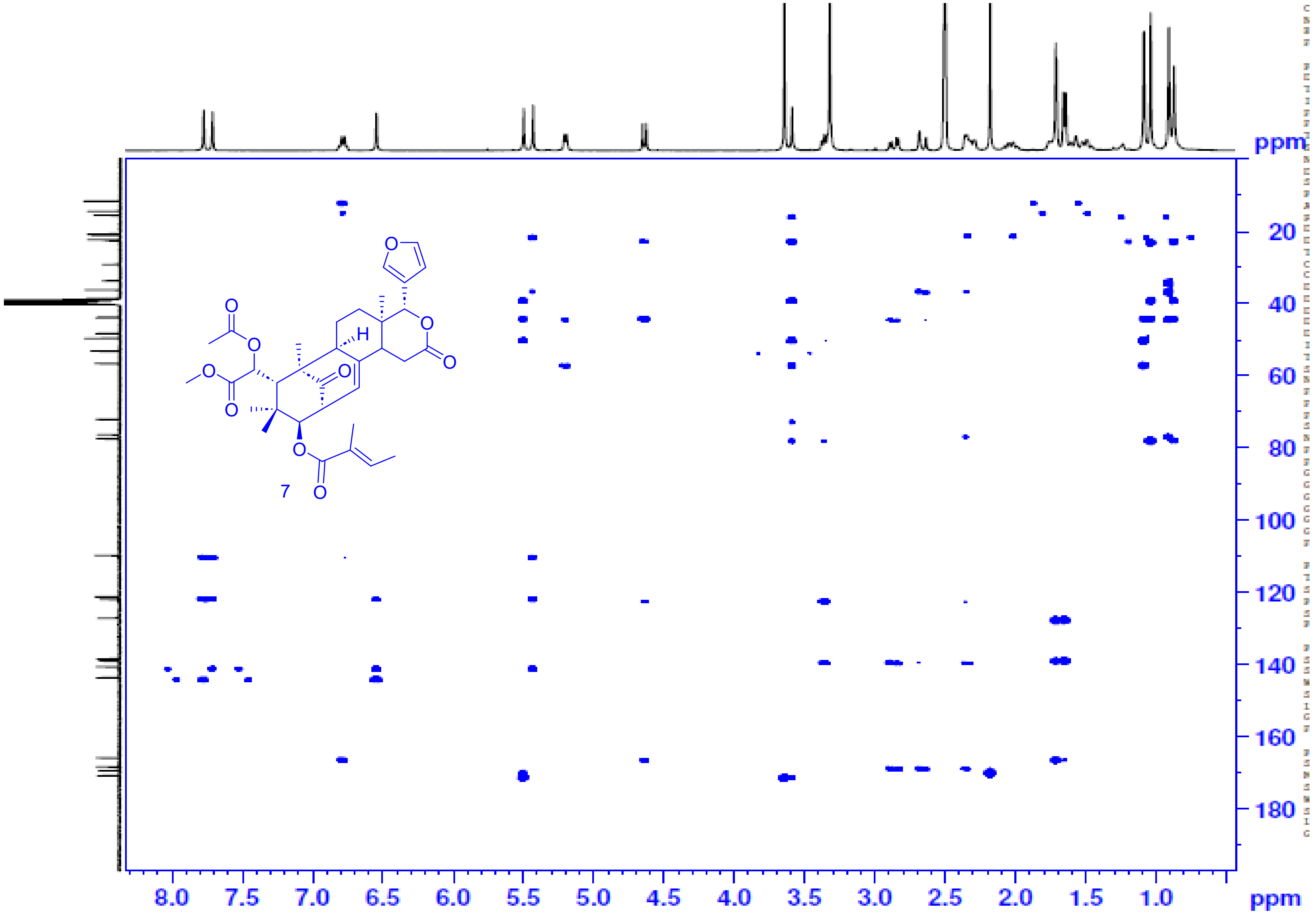

Figure S47. HRMS-ESI Mass Spectrum of compound 7

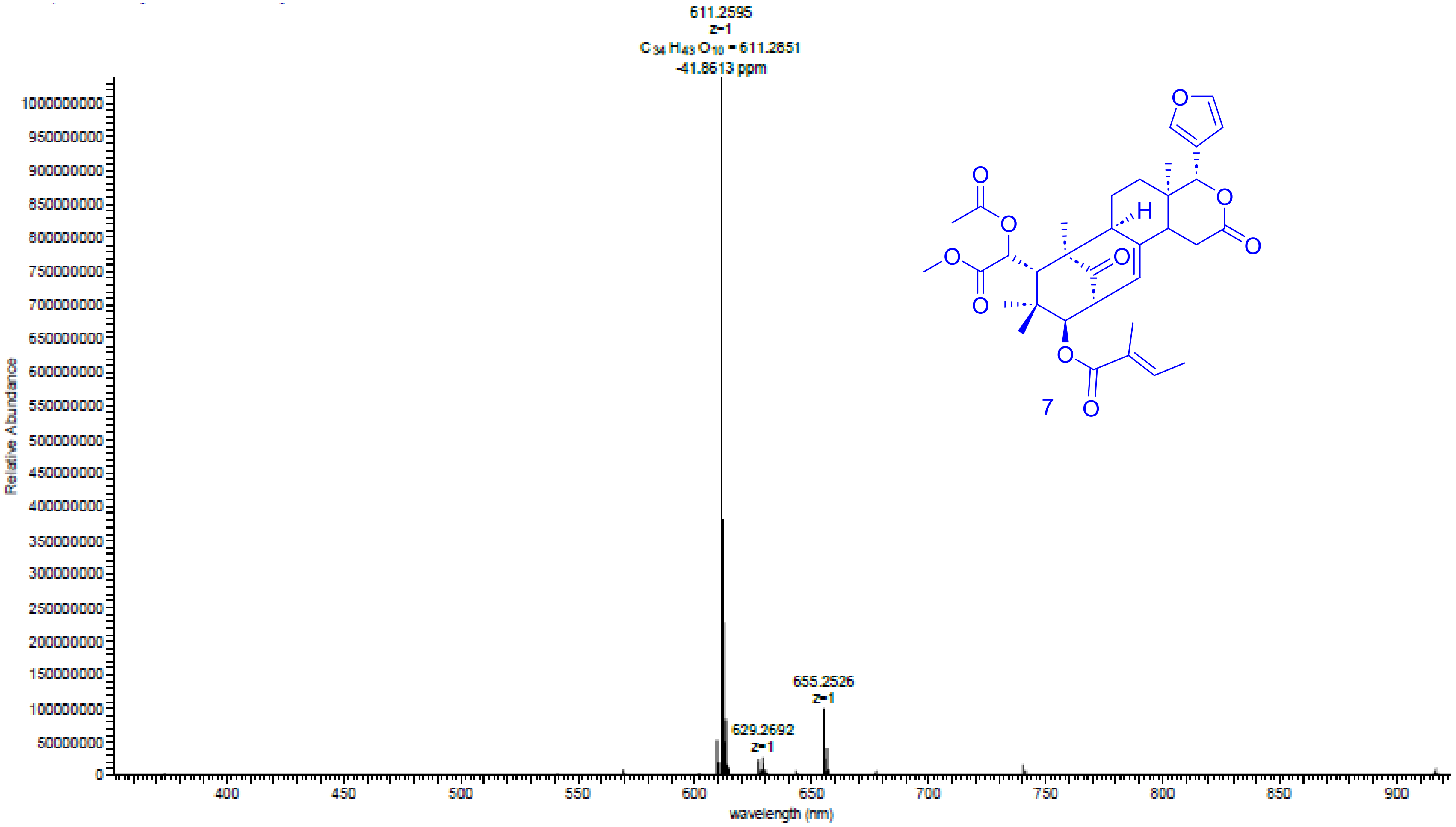

Figure S48. [1] H NMR Spectrum of compound **8** at (400 MHz) in DMSO- *d6*

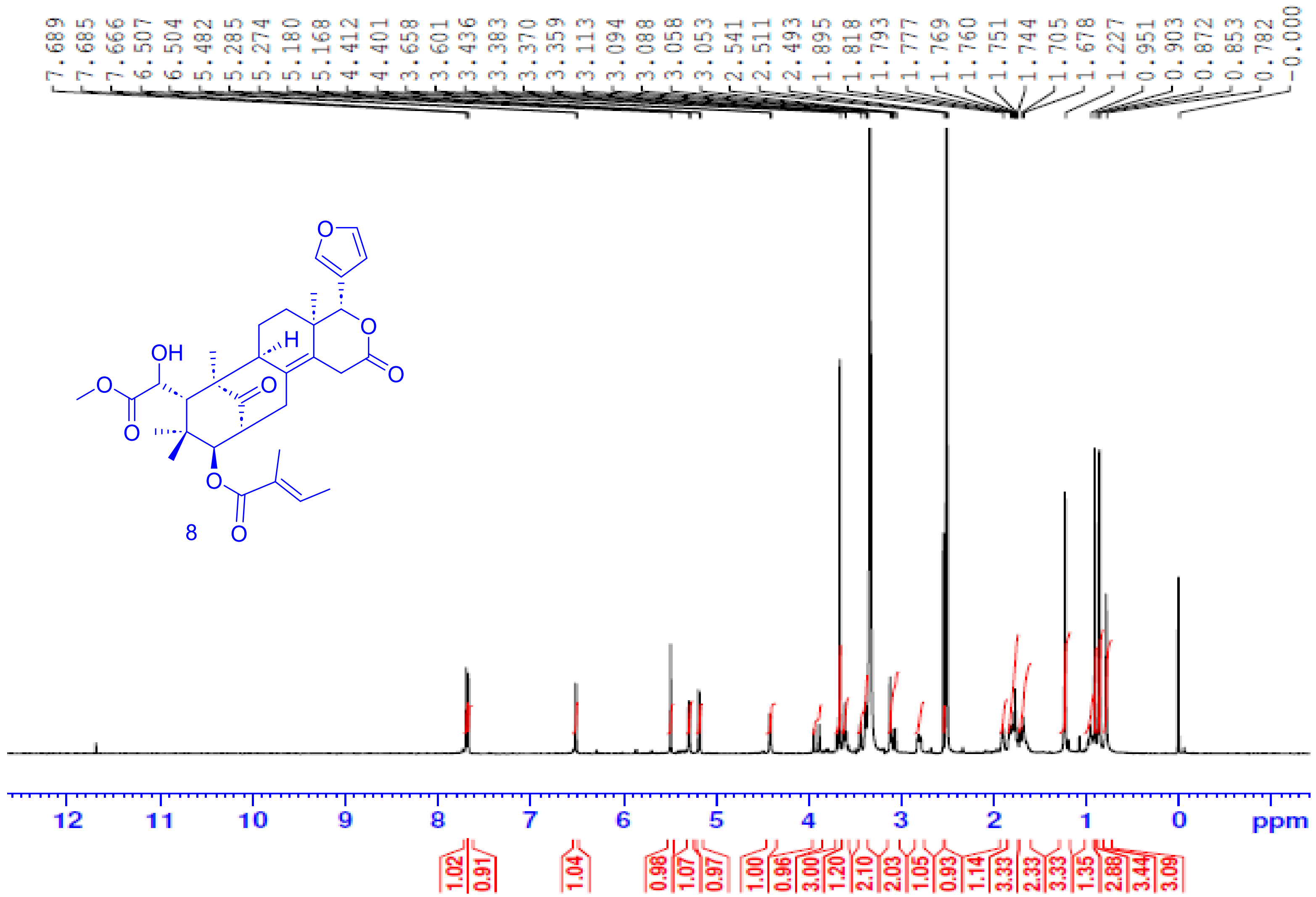

Figure S49. ESI LCMS spectrum of compound **8**

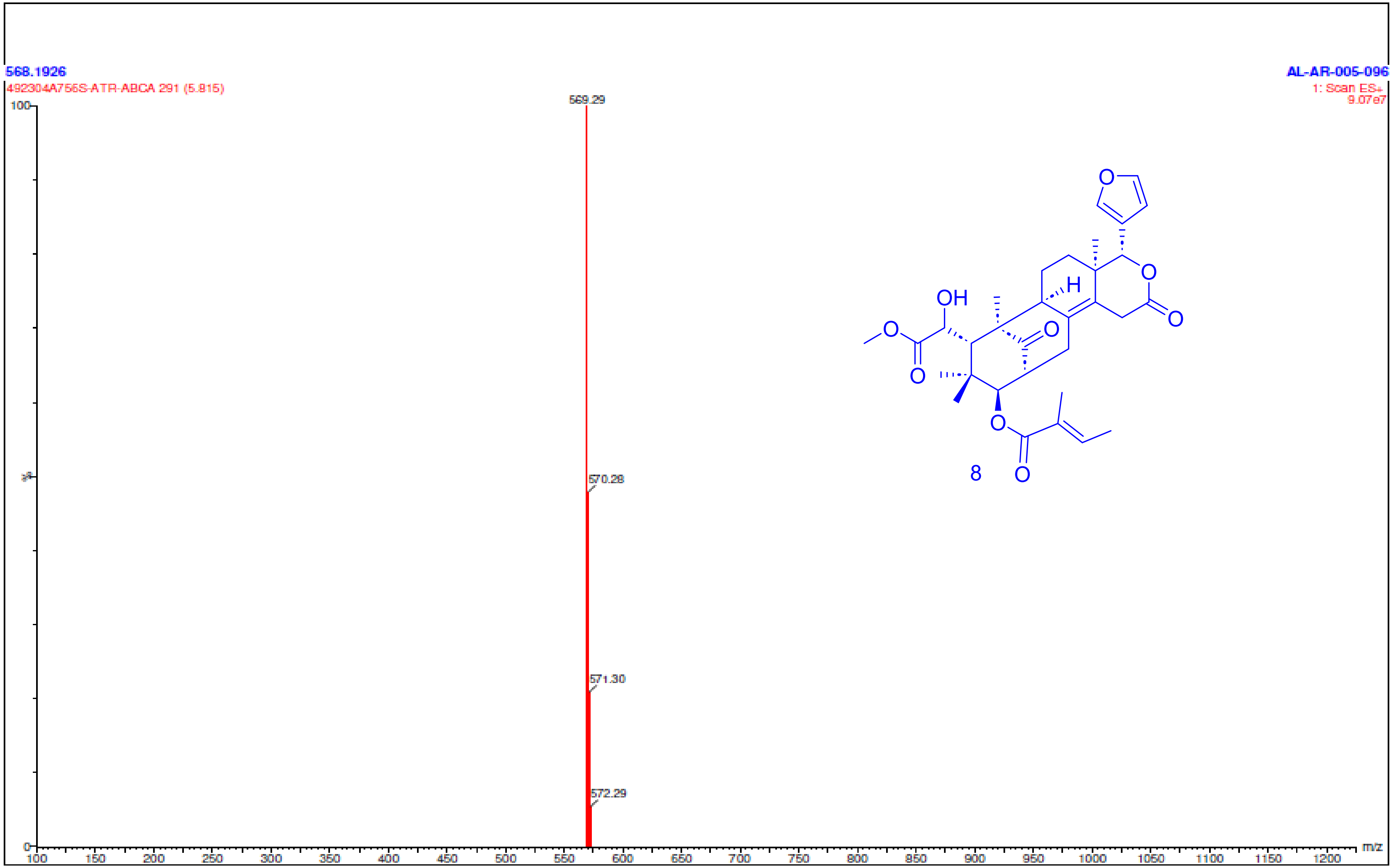

Figure S50. ^{1}H NMR Spectrum of compound **9** at (400 MHz) in DMSO- *d6*

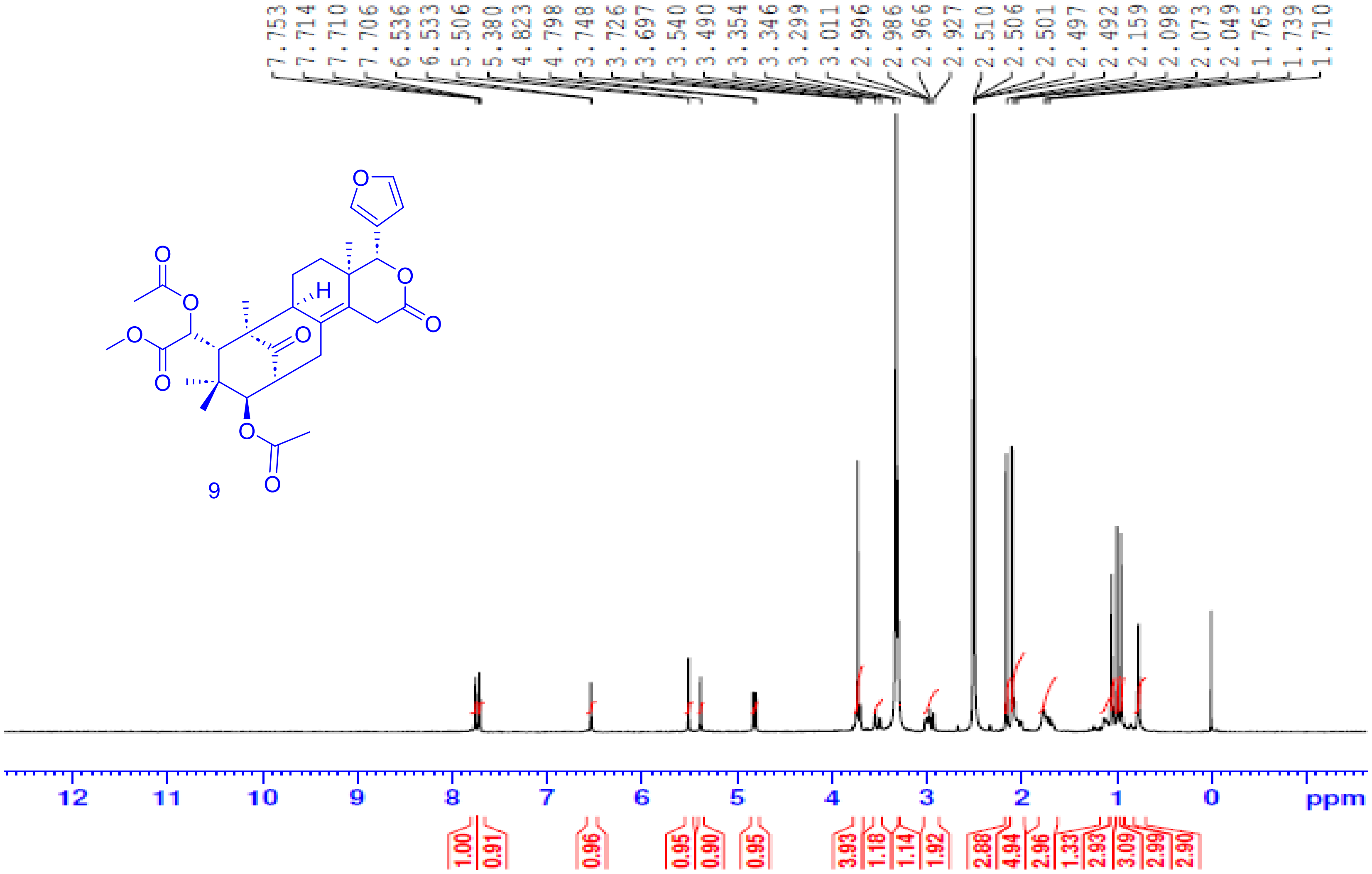

Figure S51. ^{13}C NMR Spectrum of compound **9** at (400 MHz) in DMSO- *d6*

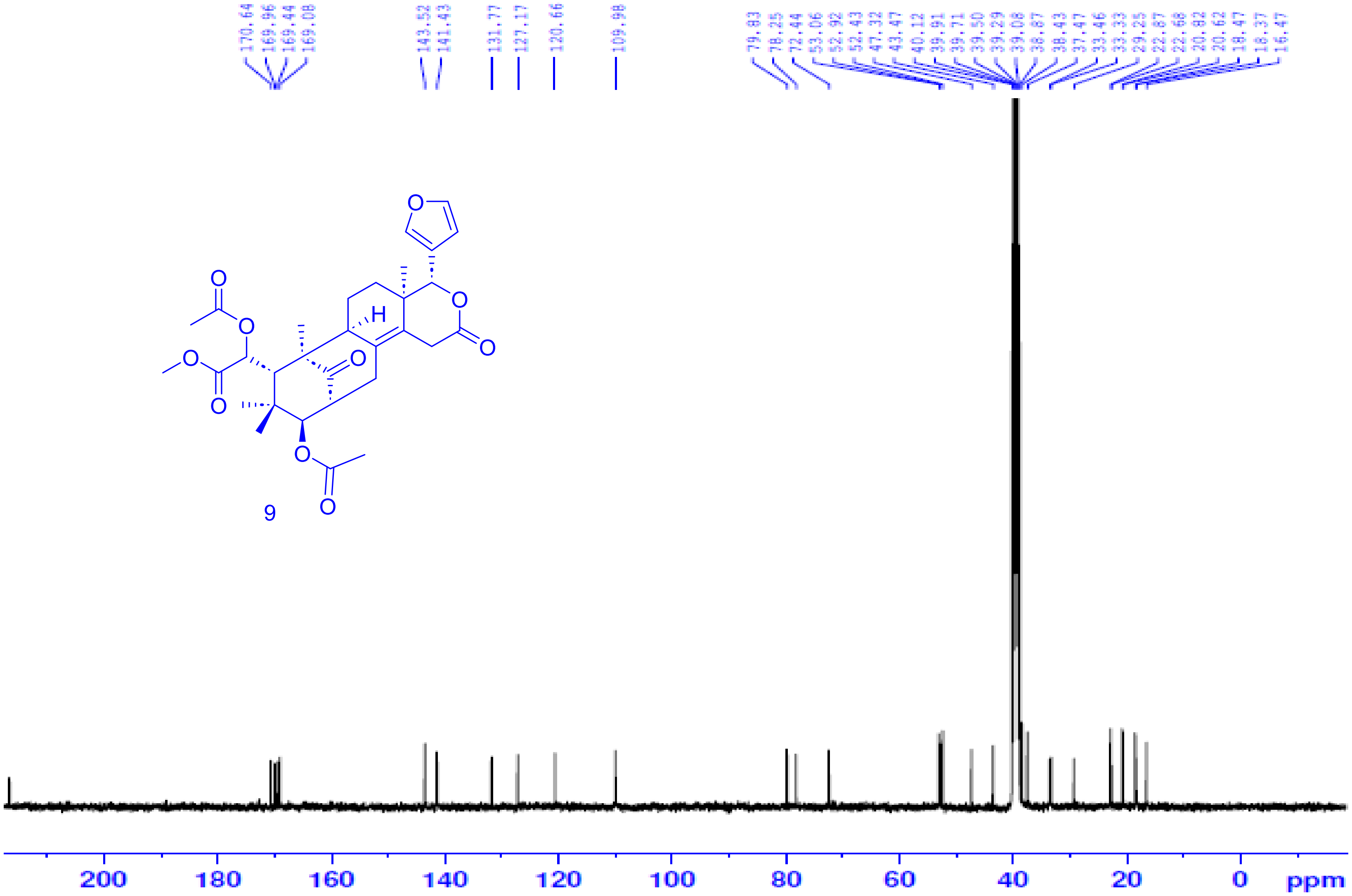

Figure S52. HSQC Spectrum of compound **9** in DMSO- *d6*

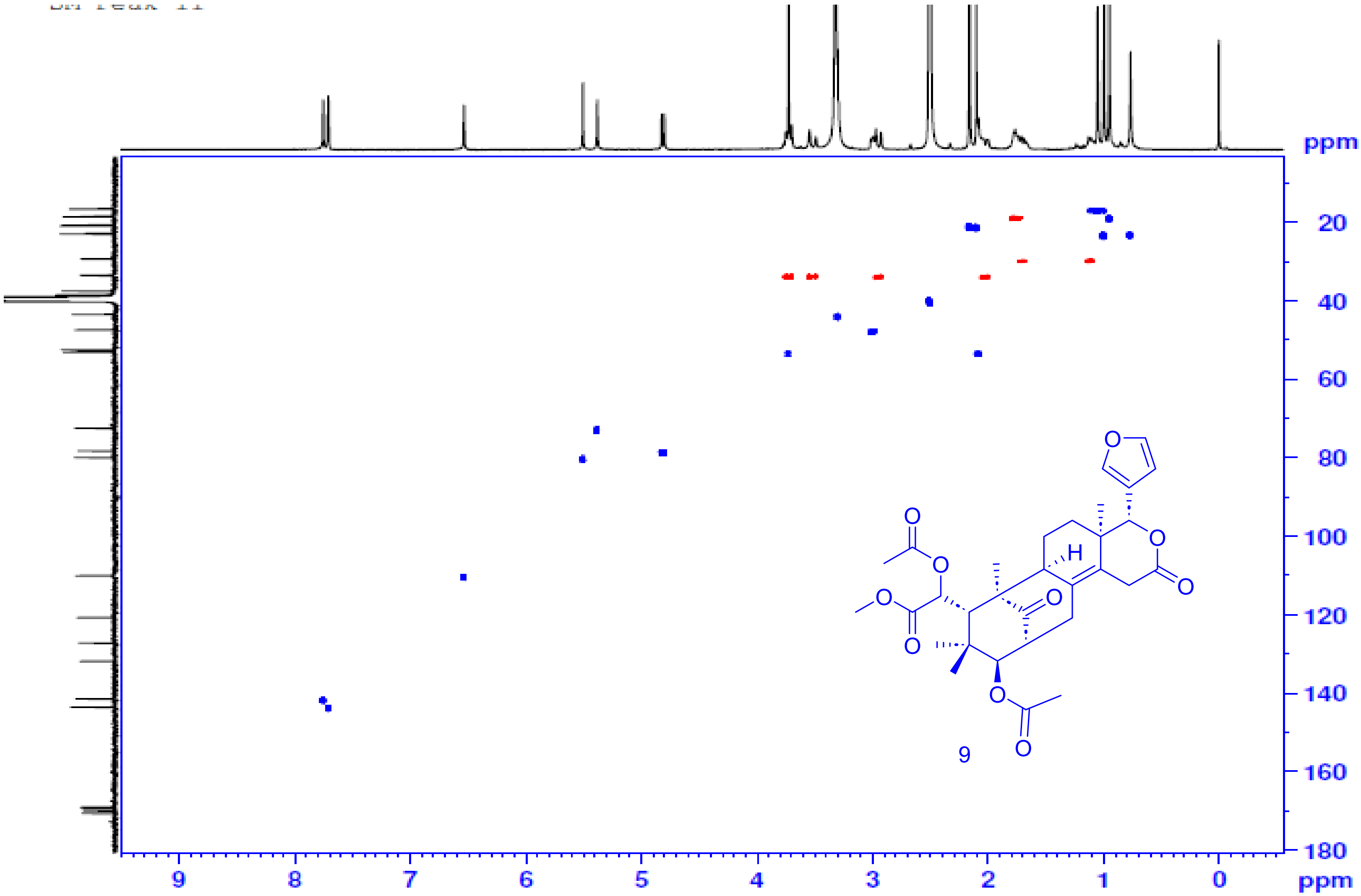

Figure S53. HMBC Spectrum of compound **9** in DMSO- d6

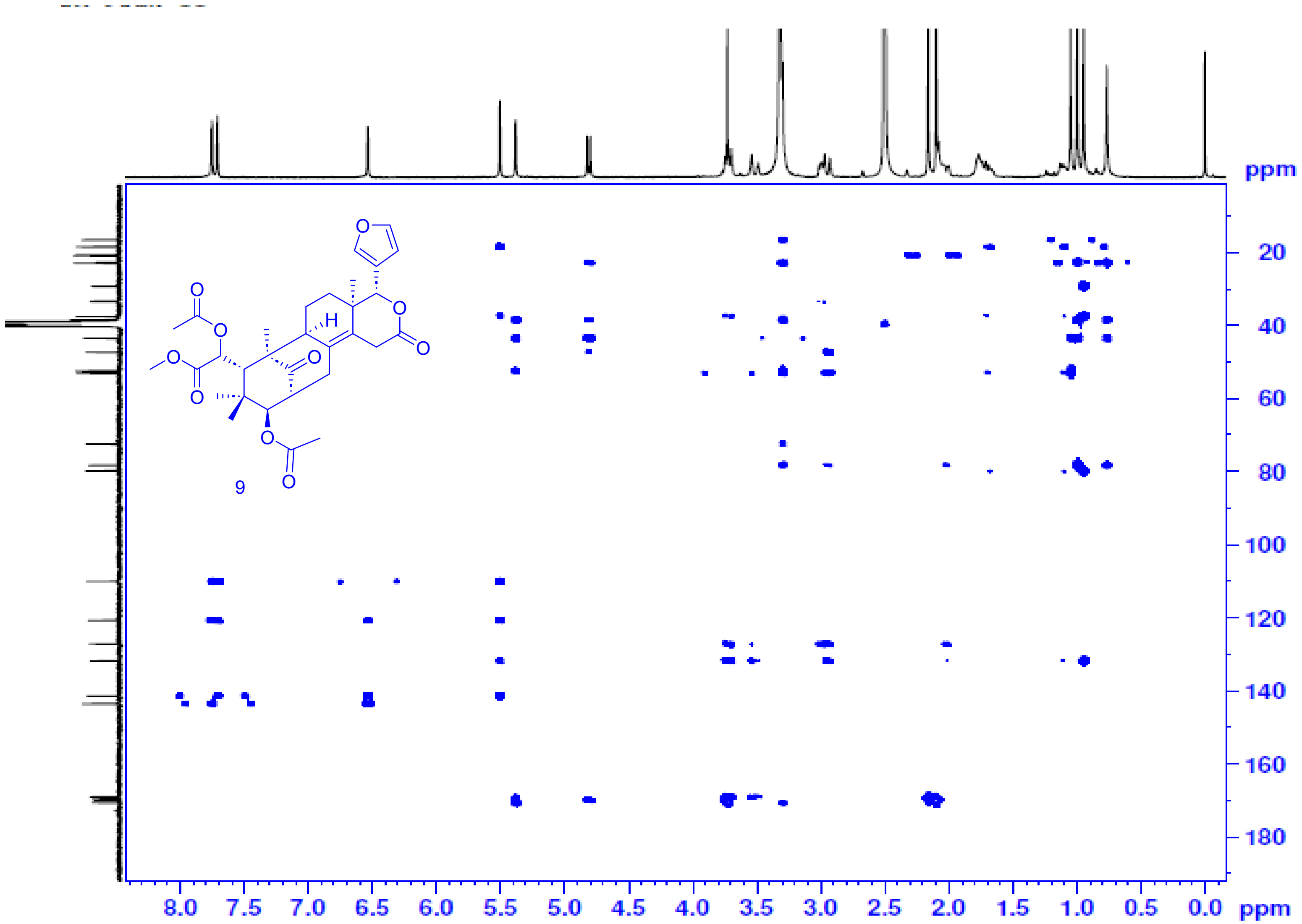

Figure S54. LC-ESI Mass Spectrum of compound 9

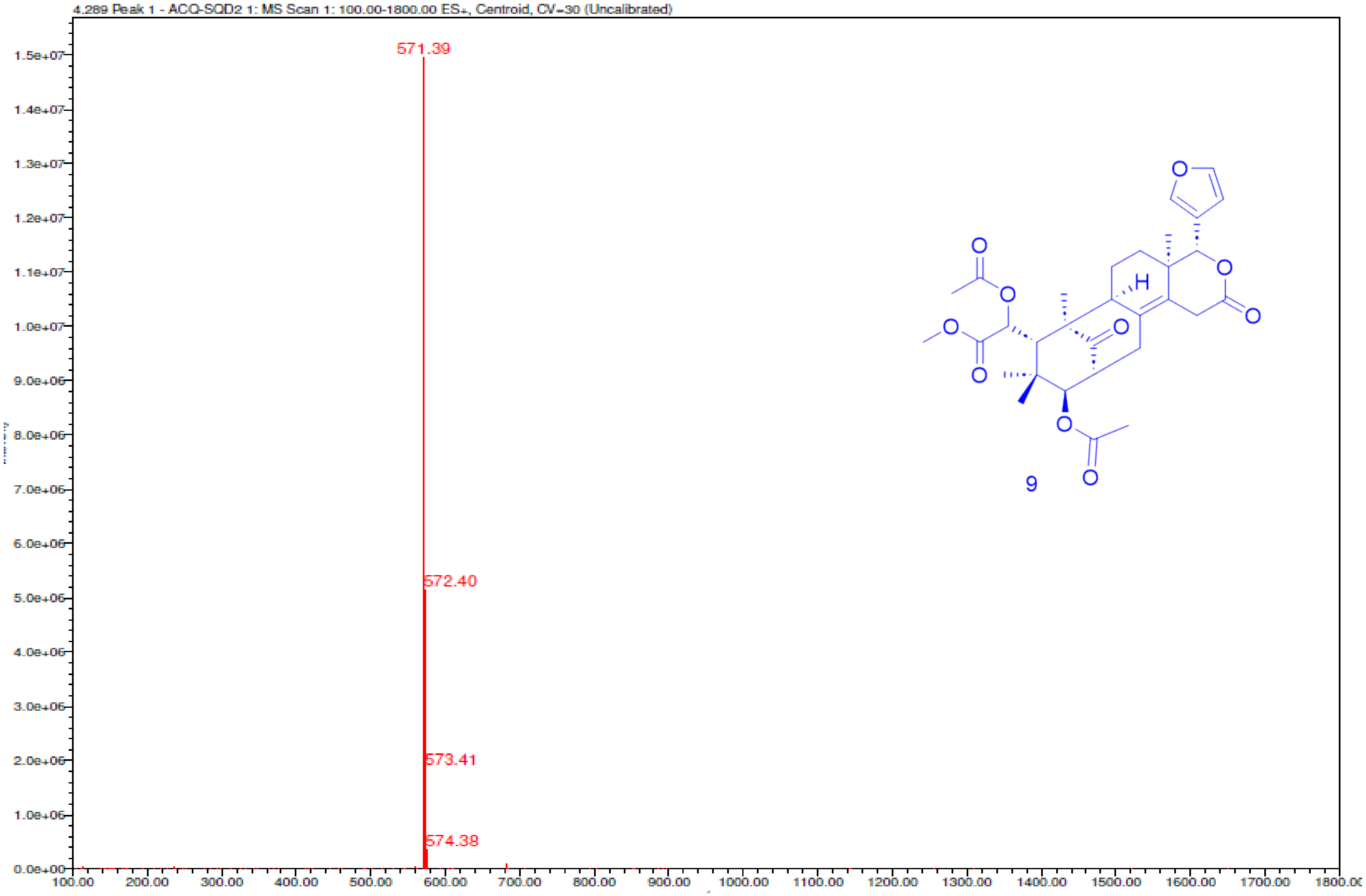

Figure S55. ^{1}H NMR Spectrum of compound **10** at (400 MHz) in DMSO- *d6*

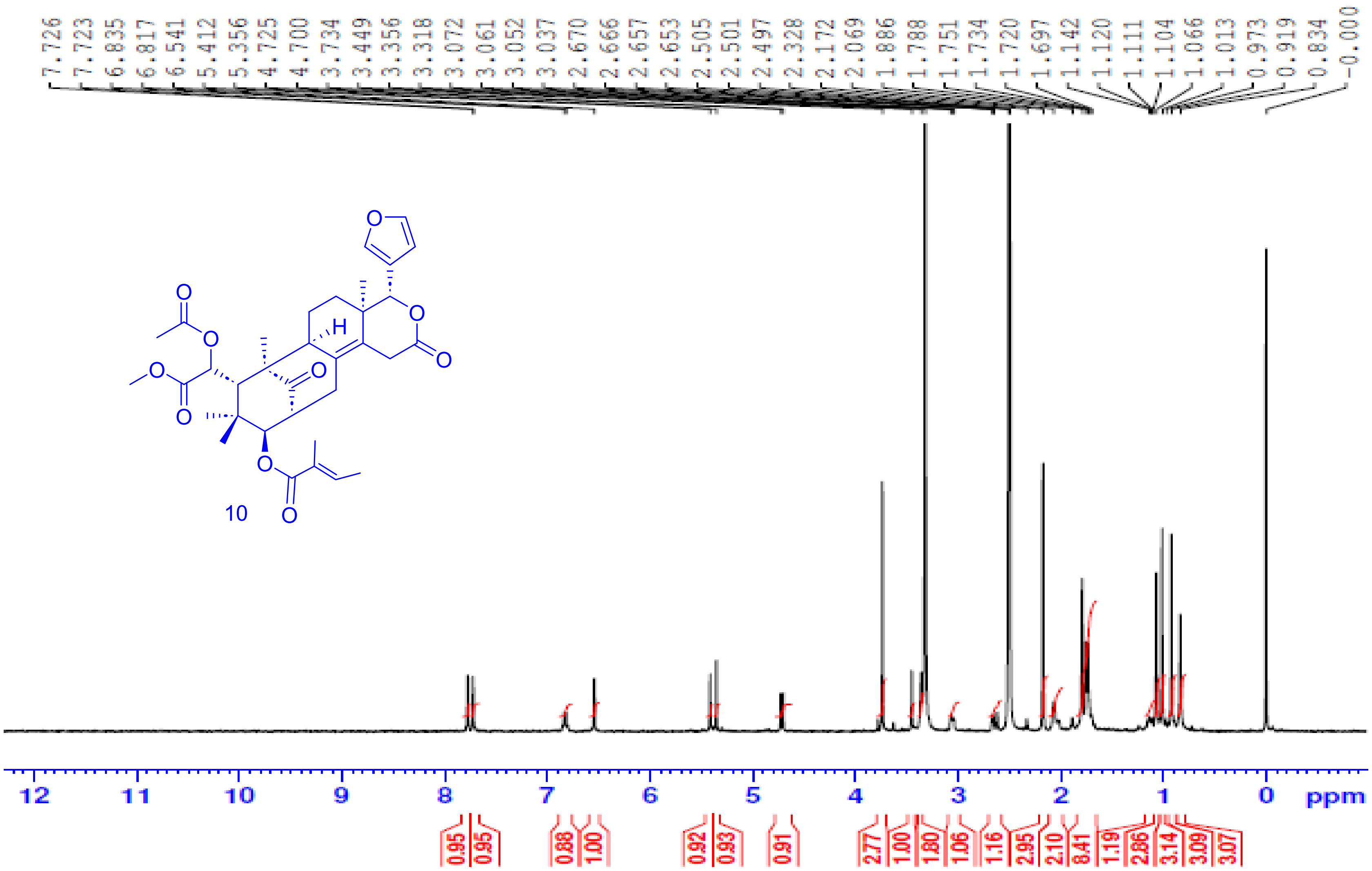

Figure S56. ^{13}C NMR Spectrum of compound **10** at (400 MHz) in DMSO- *d6*

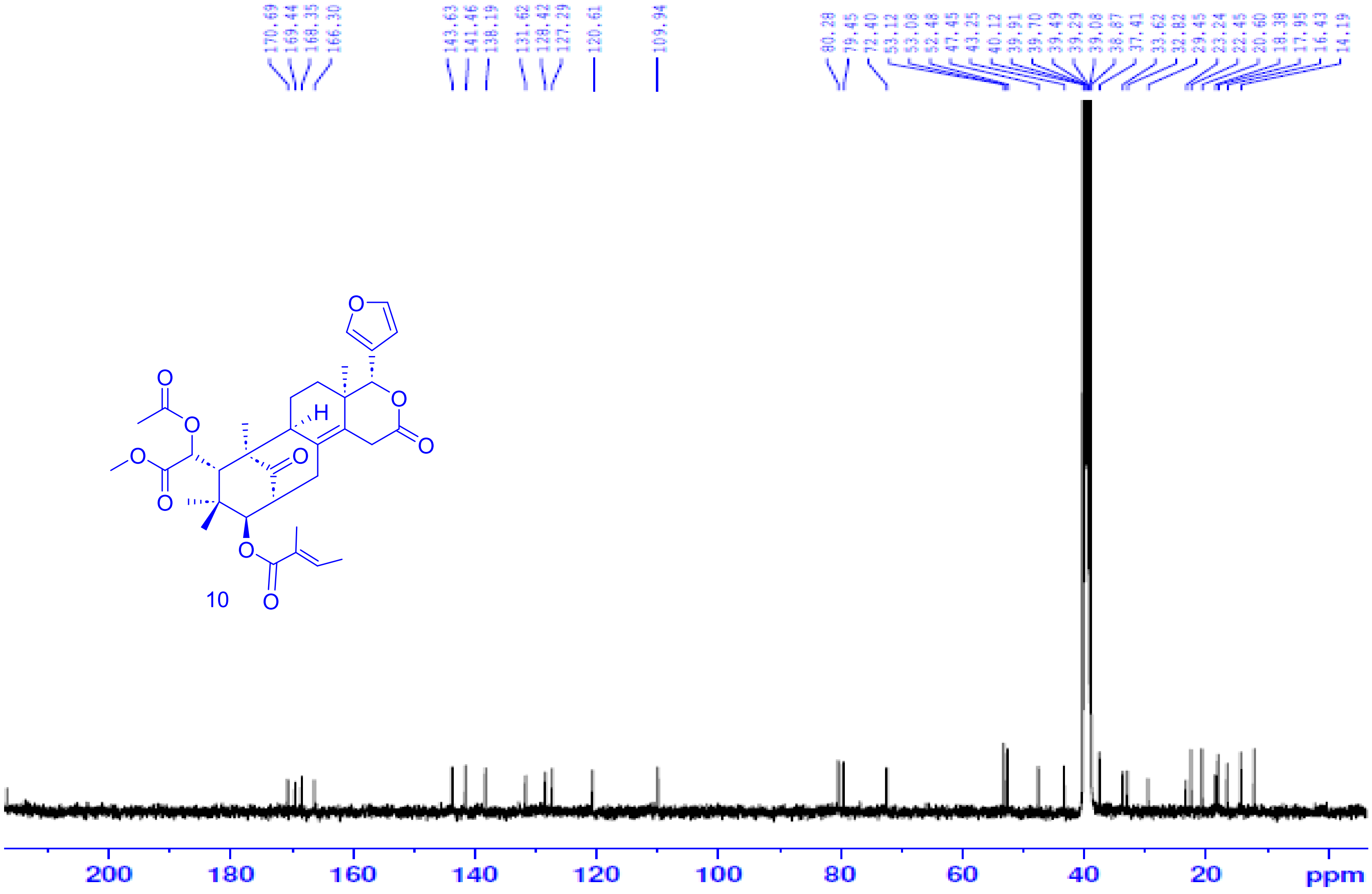

Figure S57. HSQC Spectrum of compound **10** in DMSO- d6

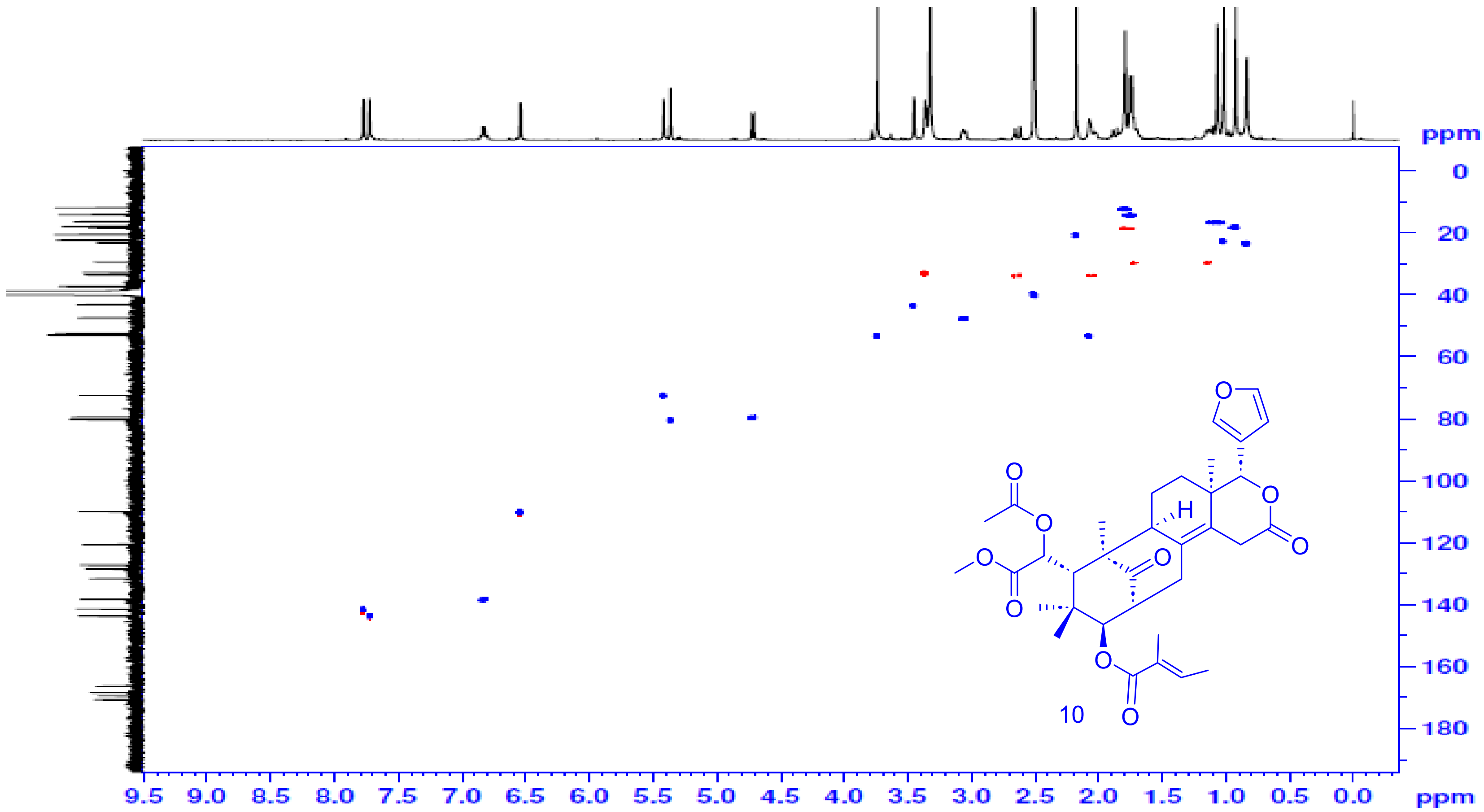

Figure S58. HMBC Spectrum of compound **10** in DMSO- d6

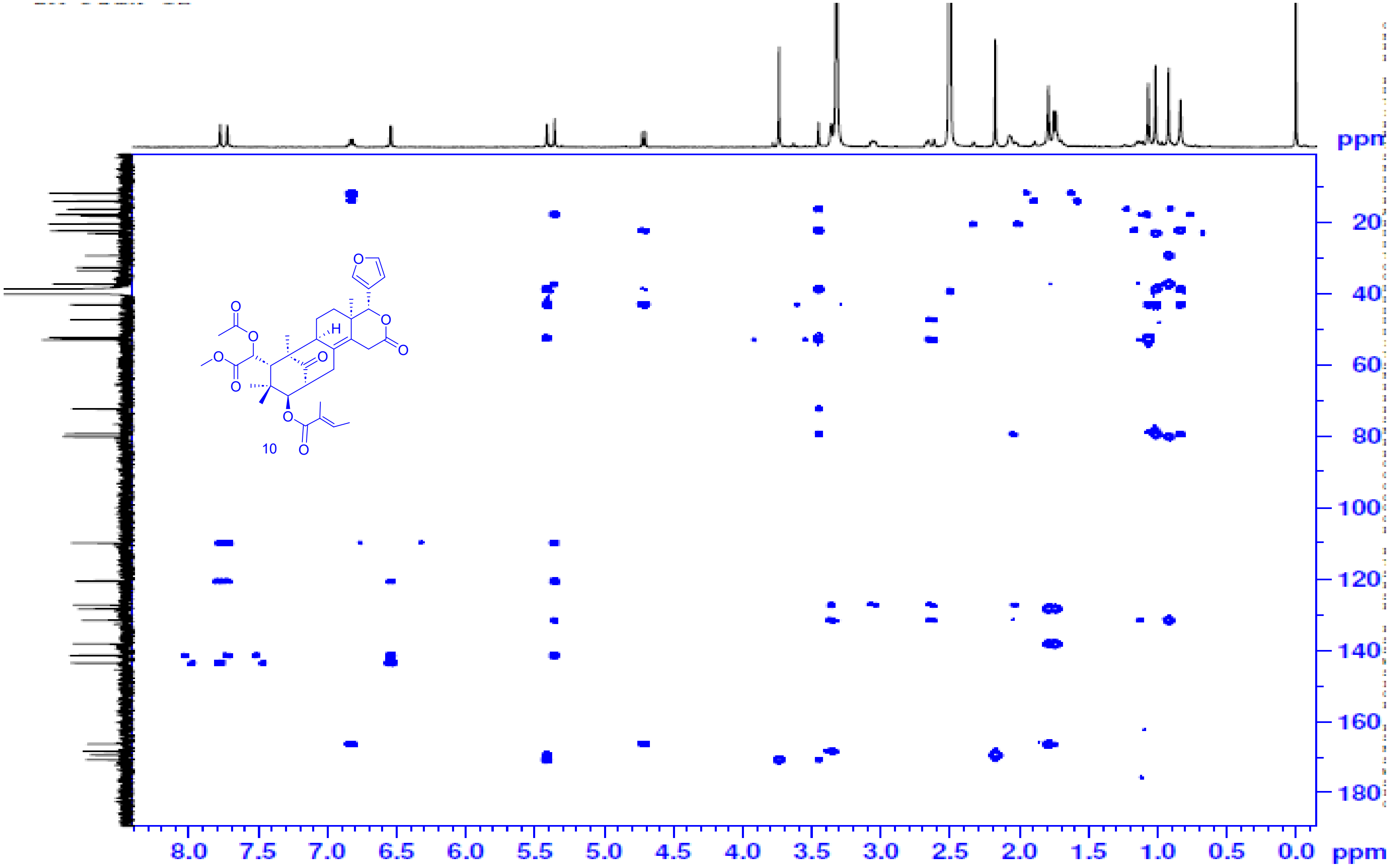

Figure S59. ESI LCMS Spectrum of compound **10**

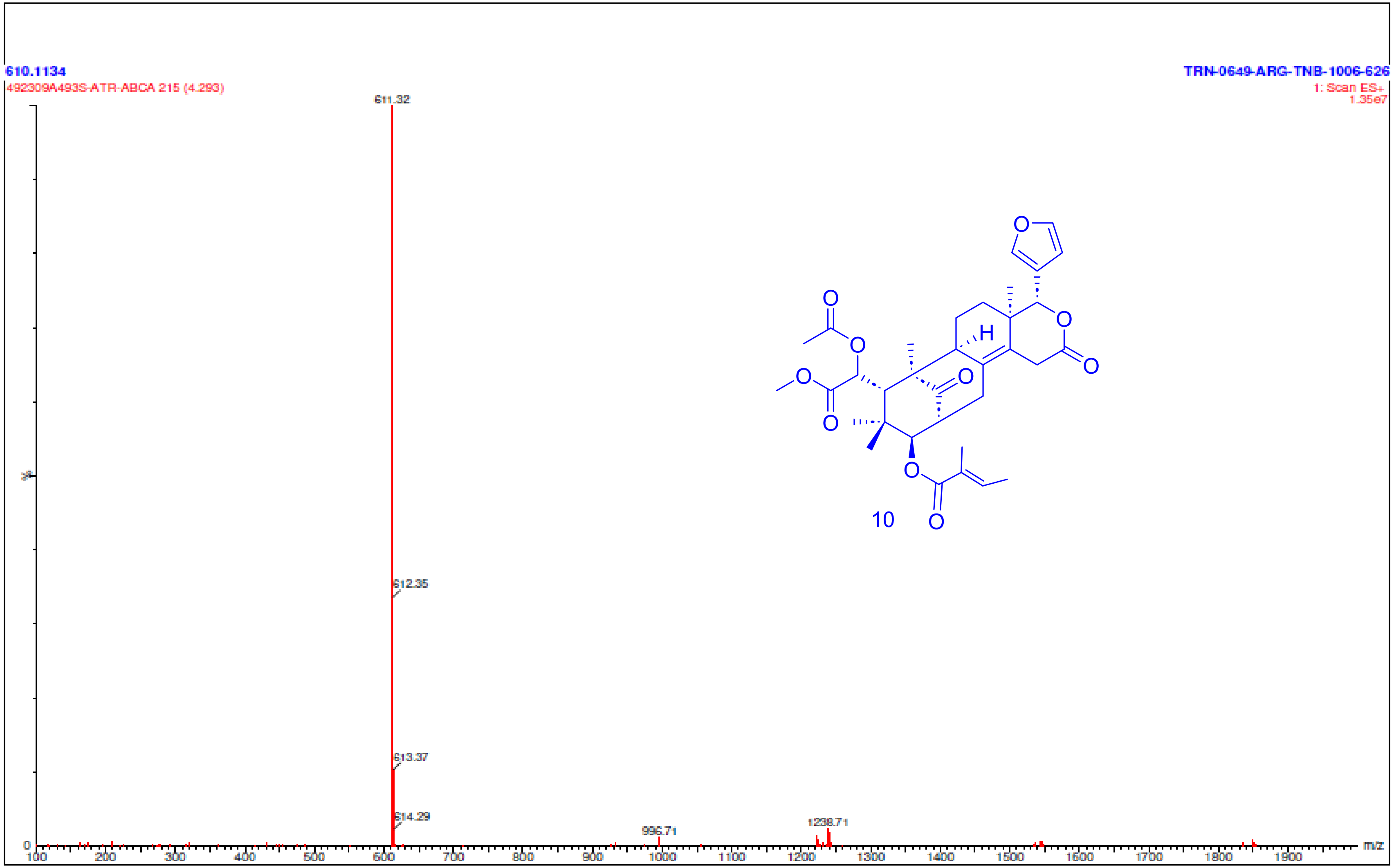

Figure S60. ^{1}H NMR Spectrum of compound **11** at (400 MHz) in DMSO- d6

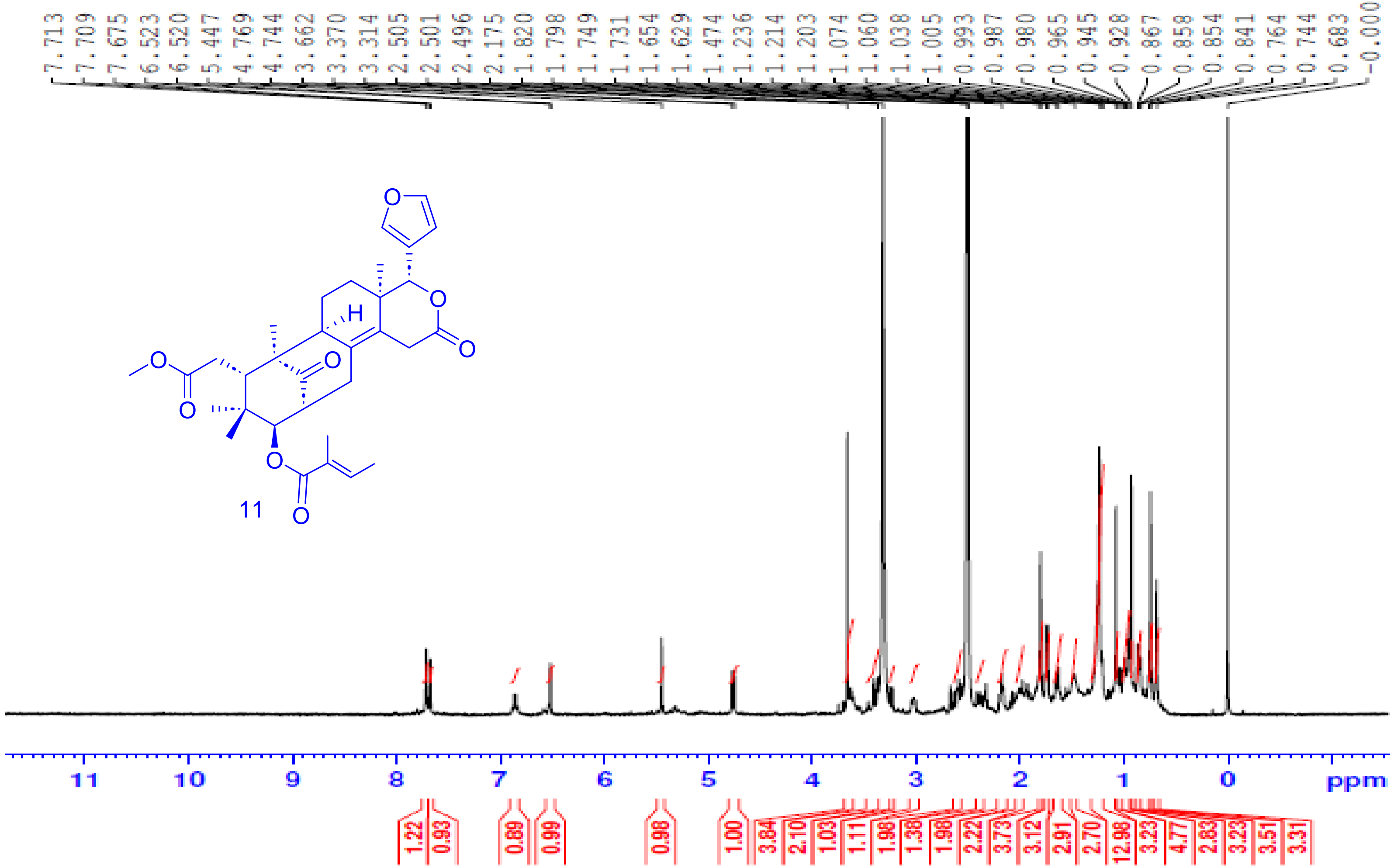

Figure S61. ^{13}C NMR Spectrum of compound **11** at (400 MHz) in DMSO- d6

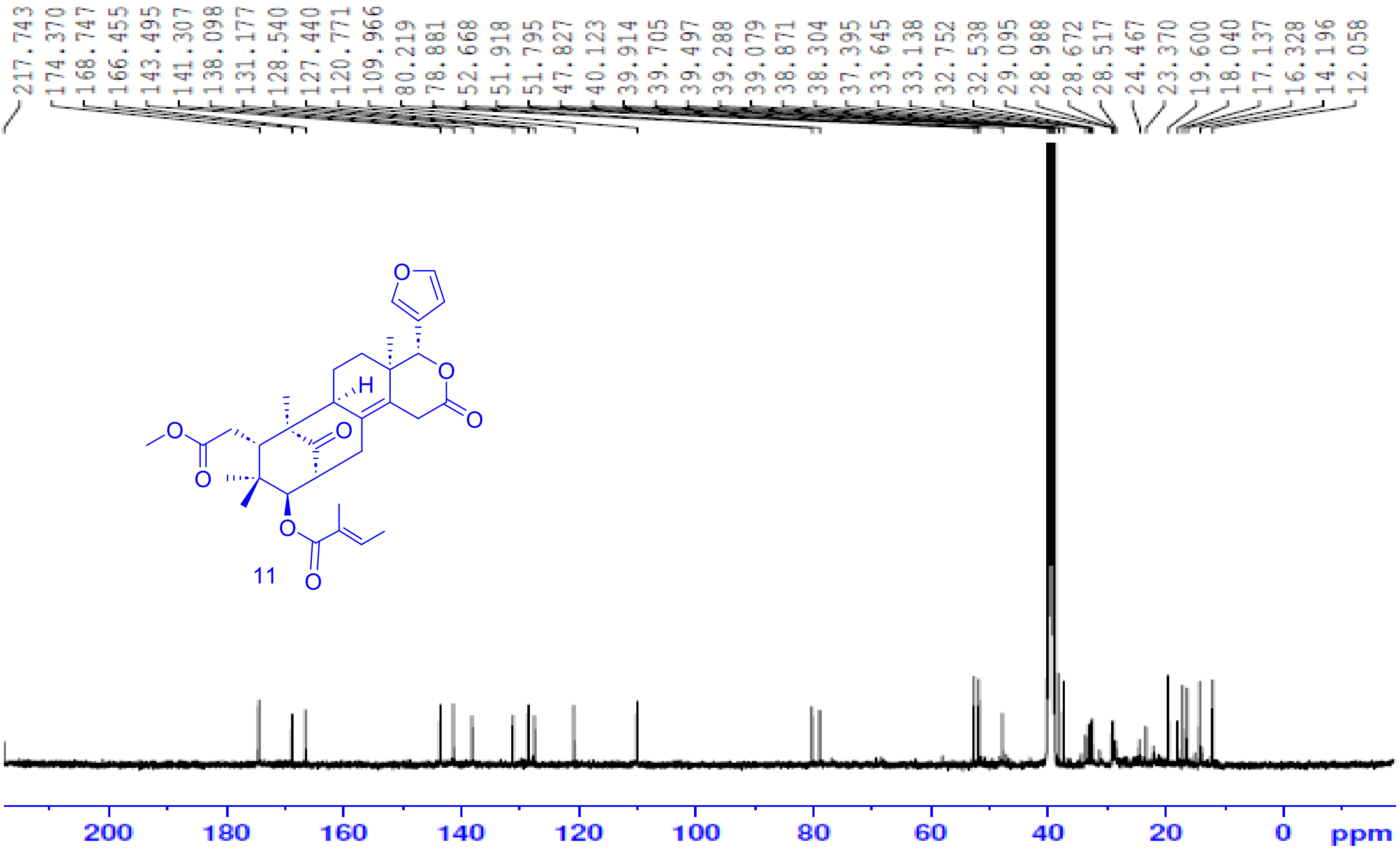

Figure S62. HSQC Spectrum of compound **11** in DMSO- d6

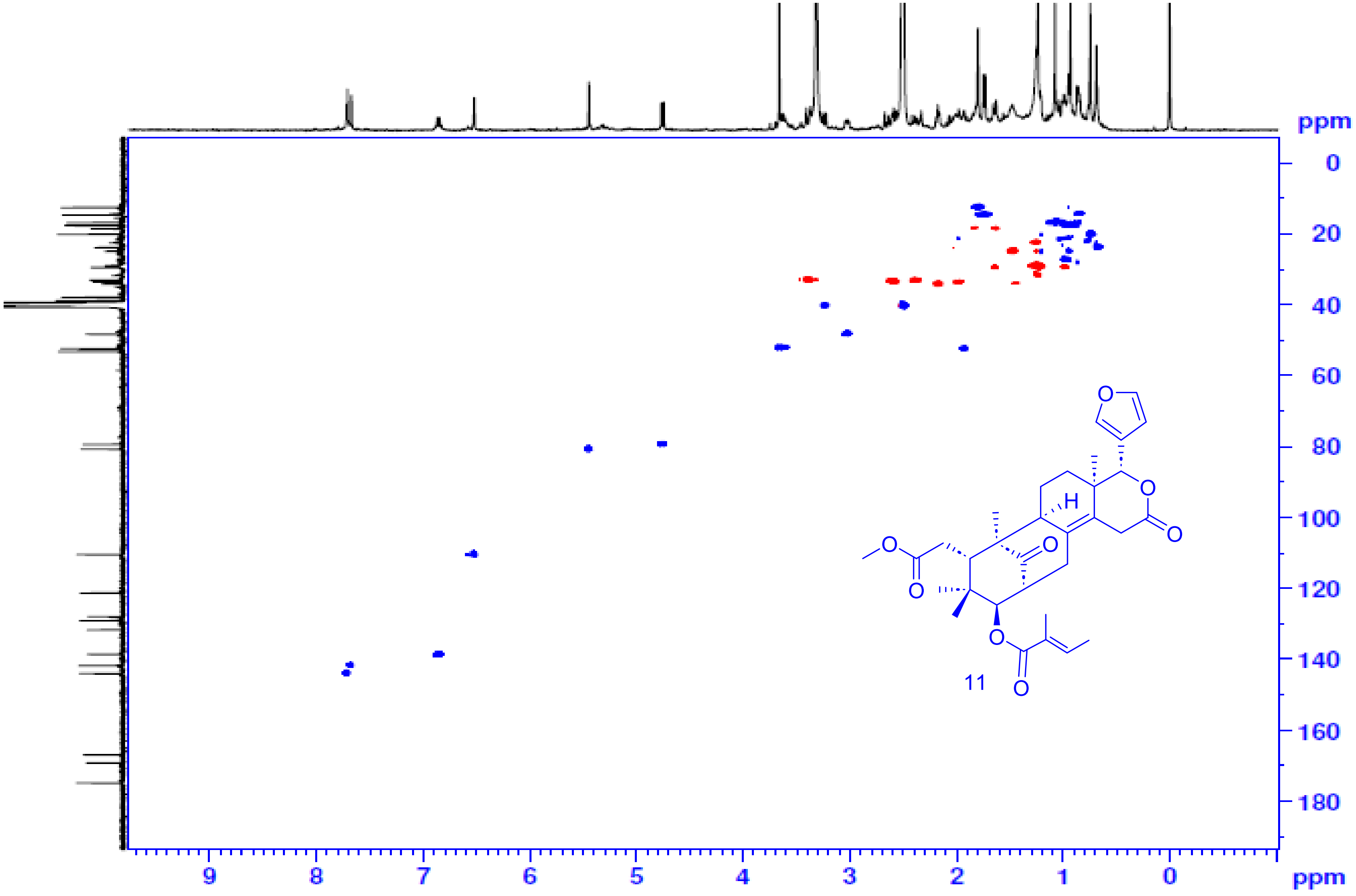

Figure S63. HMBC Spectrum of compound **11** in DMSO- d6

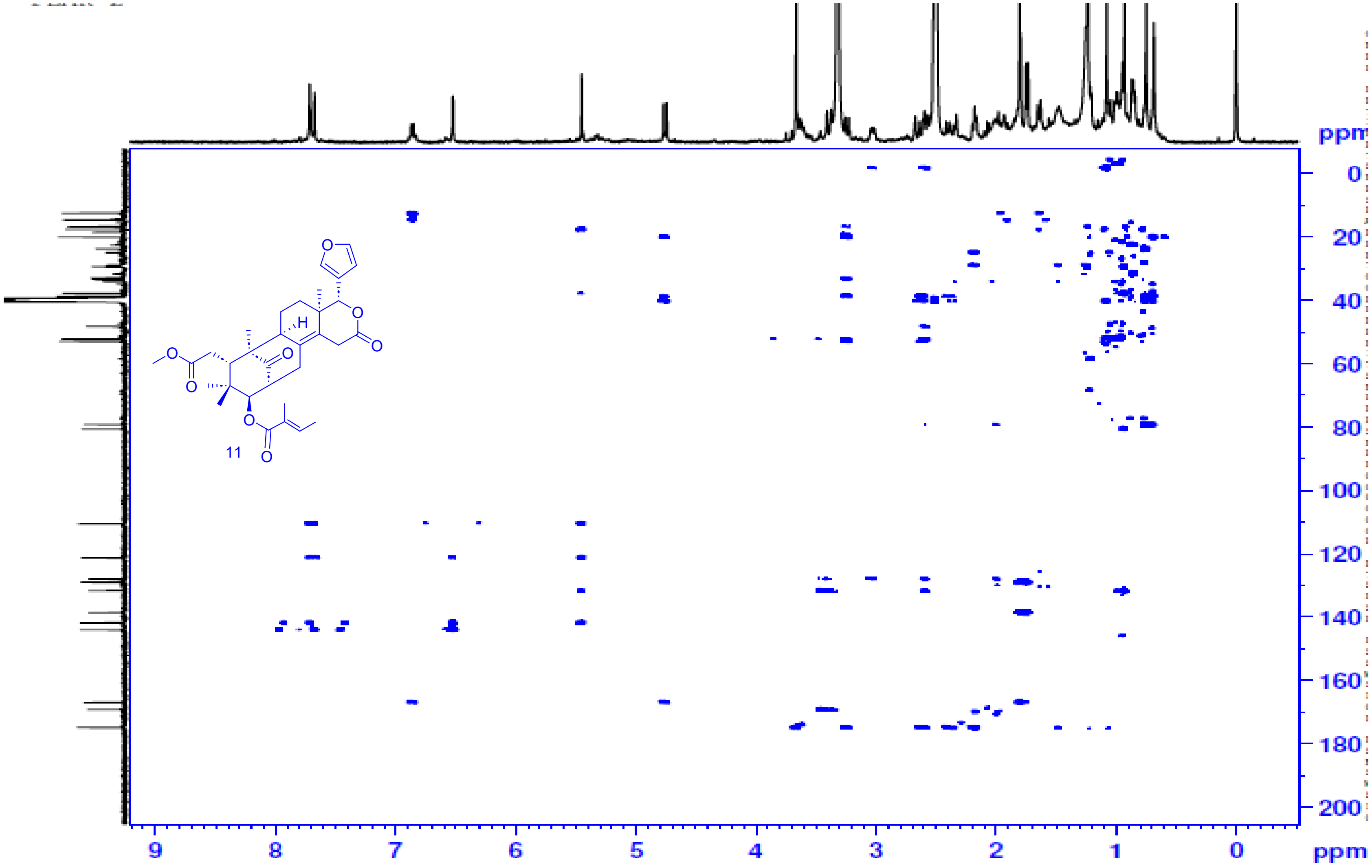

Figure S64. HRESI Spectrum of compound **11**

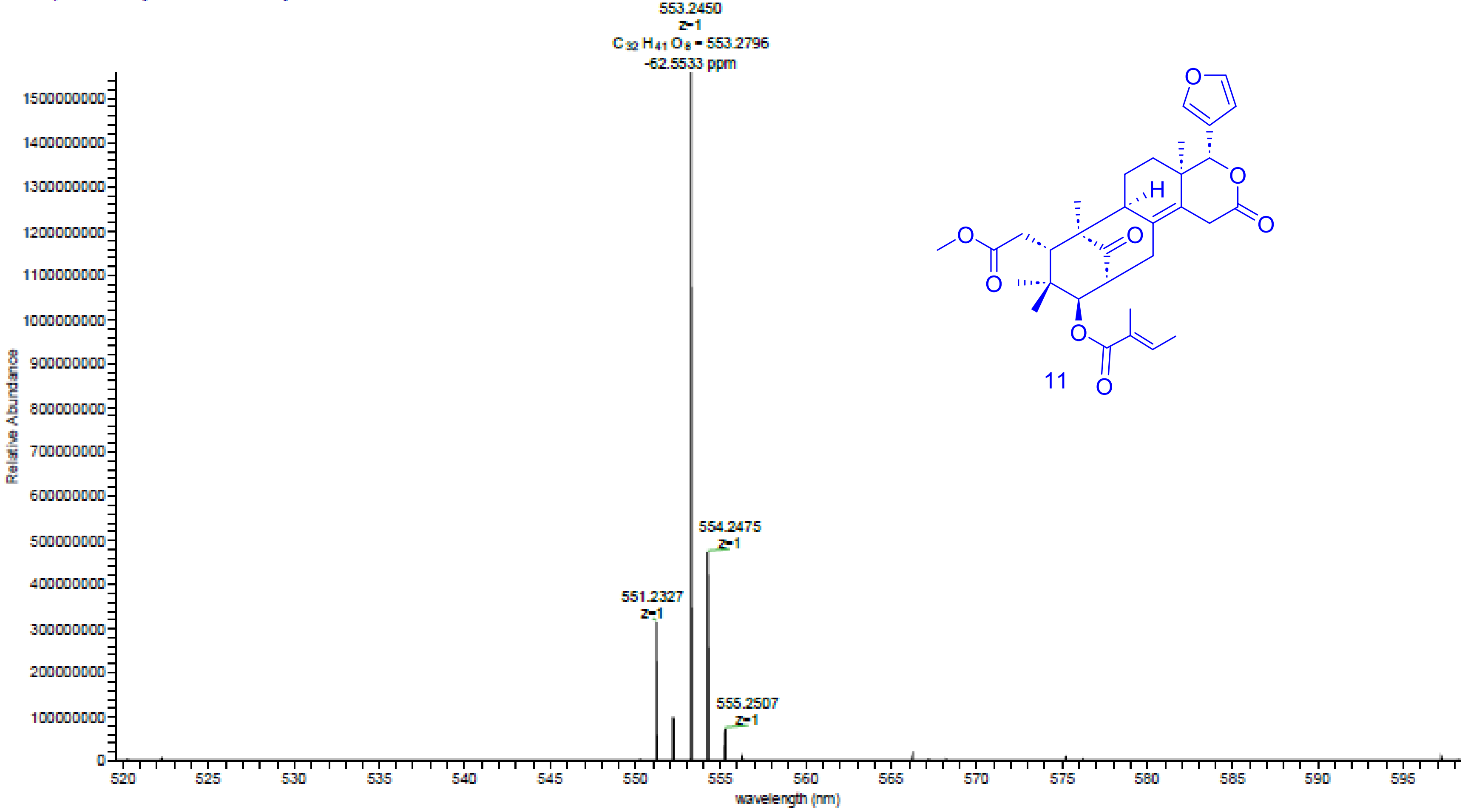

11

Figure S65. ^{1}H NMR Spectrum of compound **12** at (400 MHz) in DMSO- d6

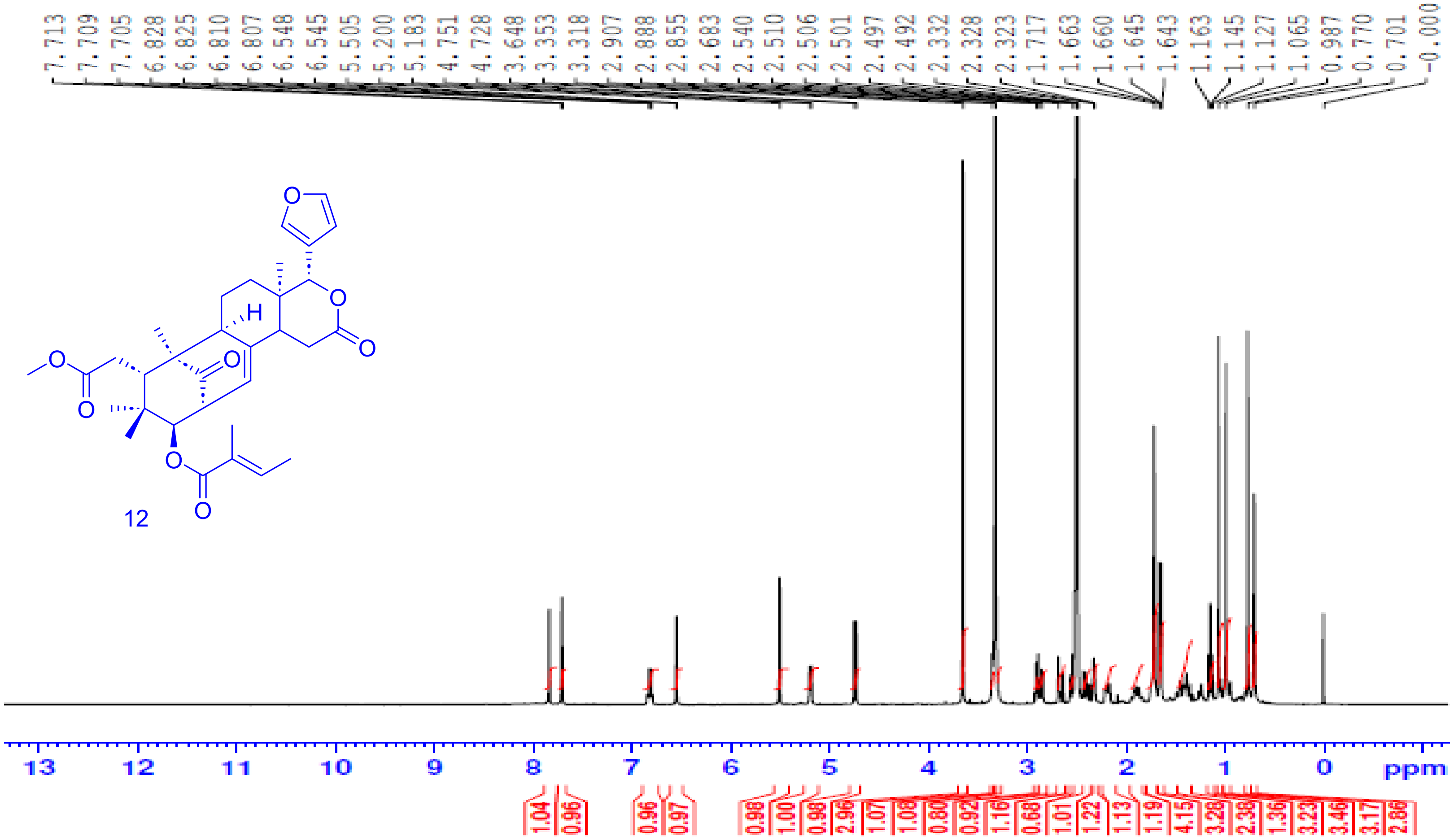

Figure S66. HSQC Spectrum of compound **12** in DMSO- d6

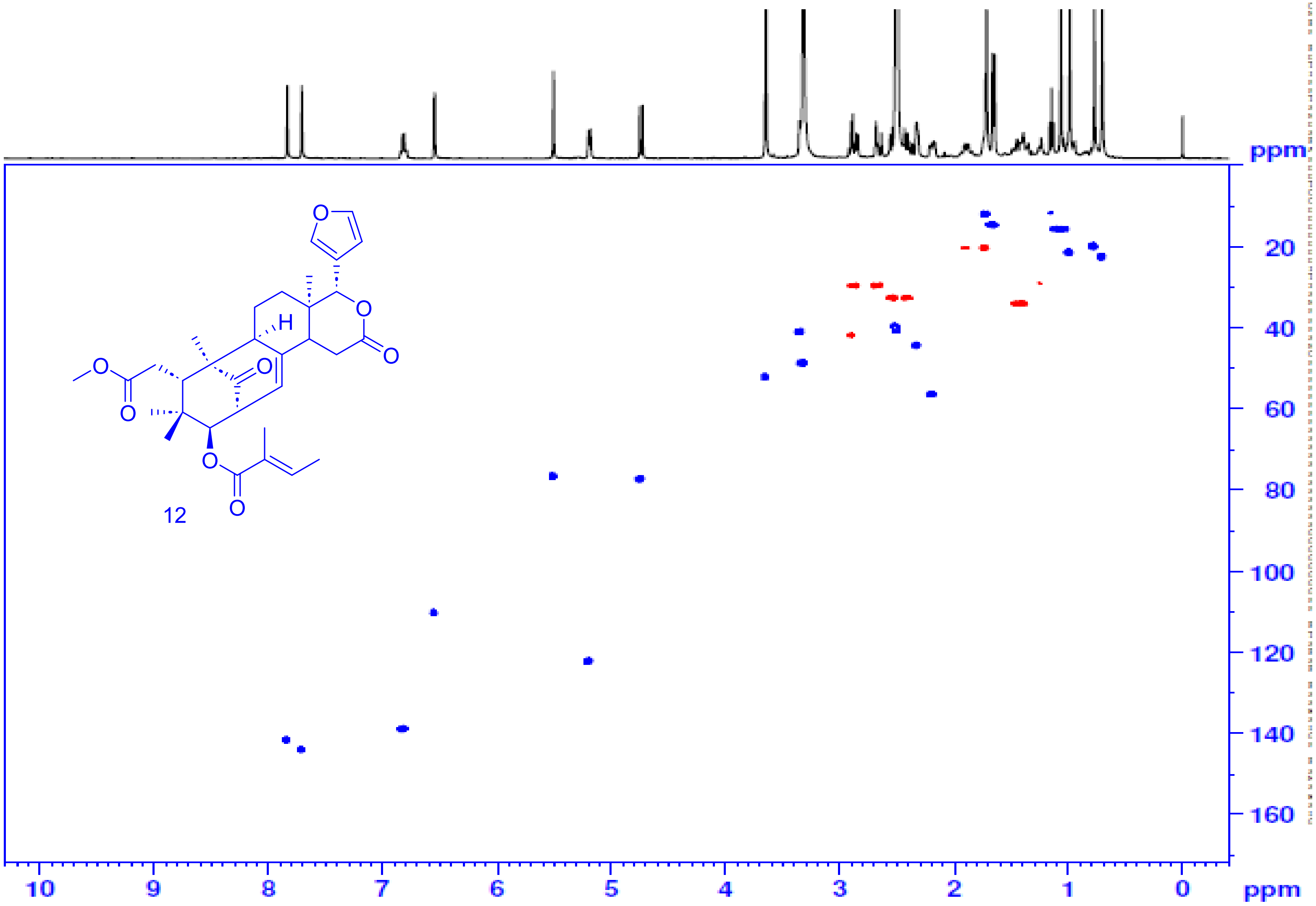

Figure S67. HSRMS Spectrum of compound **12**

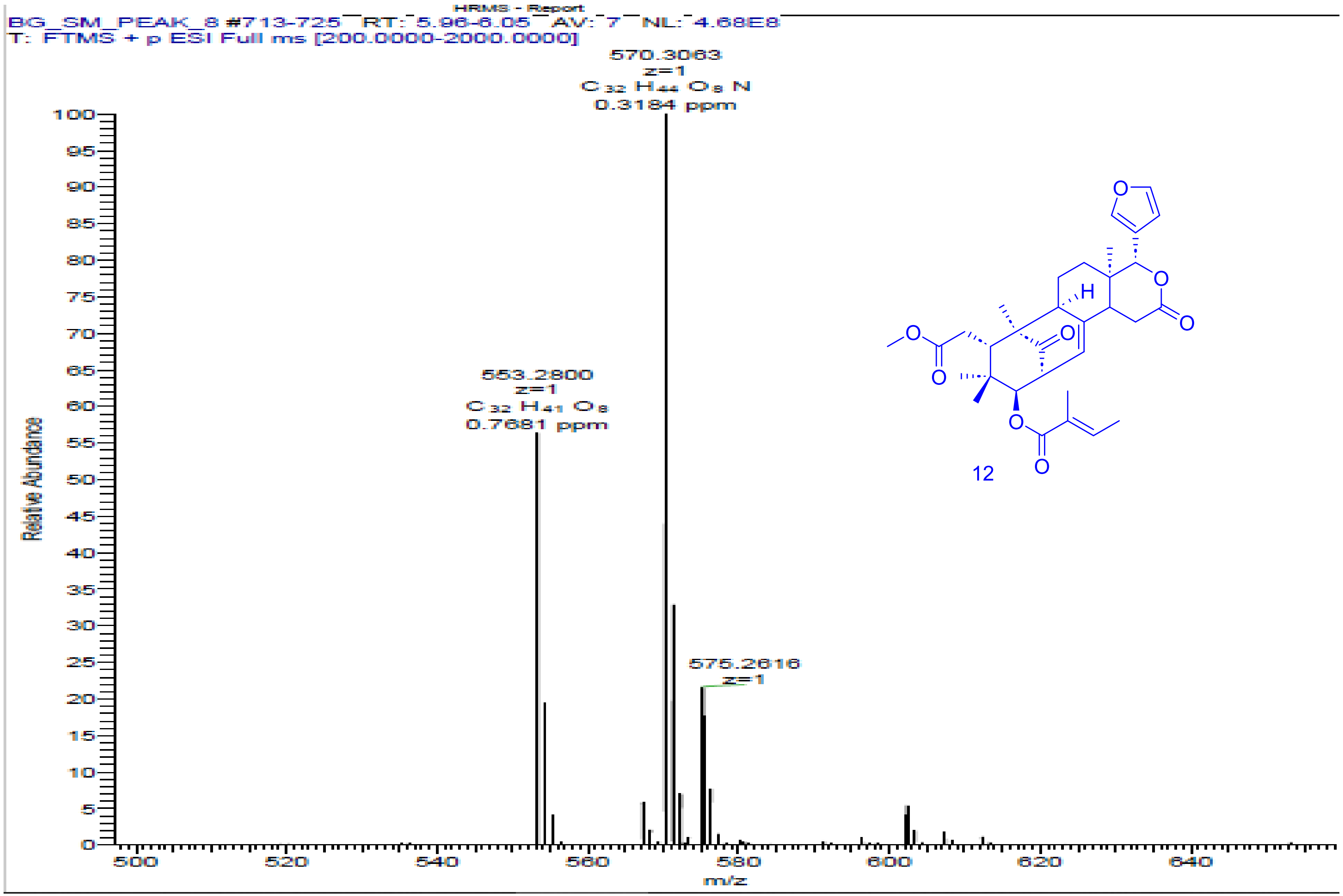

Pharmacological activities

1. Anti-inflammatory Assay

Protein denaturation has been well correlated with the occurrence of the inflammatory response and leads to various inflammatory diseases including arthritis. Tissue injury during life might be referable to denaturation of the protein constituents of cells or of intercellular substance. Hence, the ability of a substance to inhibit the denaturation of protein signifies apparent potential for anti-inflammatory activity.

METHODOLOGY:

Effect of compounds 1, 2, and 3 on Protein Denaturation

The reaction mixture, consisted of 0.2mL of 1% bovine albumin, 4.78ml of phosphate buffer saline (pH 6.4). 25µl (0.05 mg) and 50µl (0.1 mg) concentration of samples and the mixture was mixed, and was incubated in water bath (370C) for 15 min, and then reaction mixture was heated at 700C for 5 min. After cooling the turbidity was measured at 660nm. Phosphate buffer used as the negative control and Aspirin is used as a positive control (Osman et al., 2016; Kumari et al 2015).

To make 1 L of PBS, add 100 mL of 10X PBS to 900 mL of water. This PBS recipe contains 137 mM NaCl, 2.7 mM KCl, 10 mM Na_2HPO_4, and 1.8 mM KH_2PO_4.

The percentage inhibition of protein denaturation was calculated by using the formula.

$$\% \text{ inhibition of denaturation} = 100 \times [1 - A2/A1].$$

Where A1= absorption of control sample, A2= absorption of test sample.

MEMBRANE LYSIS ASSAY

Preparation of Erythroyte Suspension

For preparation of Erythrocyte suspension, Human blood was collected from a healthy human subject. The blood was centrifuged at 3000 rpm for 5 minutes in heparinized centrifuge tubes, and washed with saline (0.9% NaCl). After centrifugation the blood volume was measured and reconstituted as a 10% (v/v) suspension with isotonic buffer solution. Composition of the buffer solution (g/L) used was NaH_2PO_4, Na_2HPO_4 and NaCl (Ranasinghe et al., 2012).

Haemolytic activity

25µl (0.05 mg) and 50µl (0.1 mg) of sample and add 3ml of freshly prepared stock make it up to 5ml by using distilled water. Incubated at 37oC for 60min. After the incubation, Tubes were centrifuged at 2000rpm for 2min. The absorbance of the supernatant

was measured at 540nm against blank (Karin et al., 2020). Aspirin used as a positive control. Activity was calculated using the formula (Sæbø et al., 2023; Rafique et al.,2023):

(ODtest-ODnegative control/ODpositive -ODnegative)×100

Heat-induced Hemolytic Activity

0.05 mLof blood cell suspension and 25µl (0.05 mg) jand 50µl (0.1 mg) of sample concentration. 2.95ml of phosphate buffer (7.4 ph) added to the tubes. Incubated at 54ºC for 20 min in water bath. Aspirin used as a positive control. After the incubation, Vials were centrifuged at 5000rpm for 3min. Supernatant were collected and absorbance measured at 540nm (Ranasinghe et al., 2012; Moualek et al., 2016; Sakat et al., 2010) .

Formula for Heat induced haemolytic assay percentage calculation:

(1-ODS)/ODC *100

Whereas, ODC-optical density of control, and ODS-optical density of sample.

2. α- Amylase inhibition activity

The α-amylase inhibition assay was conducted using a modified technique based on the method developed by Kusano et al. The presence of undigested starch, resulting from the inhibition of enzymes, was identified at a wavelength of 630 nm. This was shown by the blue color of the starch-iodine combination. The substrate was produced by dissolving 200 mg of starch in 25 ml of 0.4 M NaOH solution through heating at 100°C for 5 minutes. Following the cooling process, the pH was modified to 7.0 and the total volume was increased to 100 ml by adding distilled water. Acarbose served as the positive control. Pre-incubation was performed by combining 40 µl of substrate solution with 20 µl of either acarbose or plant extract at different concentrations (10, 20, 40, 80, 160, and 640 µg/ml). The mixture was then incubated at 37°C for 3 minutes. Next, 20 µl of α-amylase (at a concentration of 3 U/ml) was added to the mixture, which was then incubated at 37°C for 15 minutes. The α-amylase was prepared in a 20 mM phosphate buffer with 6.7 mM NaCl, pH 6.9. The reaction was terminated by adding 80 µl of hydrochloric acid (0.1 M). Subsequently, a volume of 100 µl of iodine reagent with a concentration of 2.5 mM was introduced, and the absorbance was determined at a wavelength of 630 nm (Ritupama et al 2014; Balan et al.,2017; Ogunyemi et al., 2022).

% inhibition= (Absorbance-control− Absorbance-test) Absorbance-control ×100.

3. Antimicrobial activity of the samples using well diffusion method

The anti-bacterial properties of tested against a variety of bacteria, including Staphylococcus epidermis (MTCC 2044), Bacillus cereus (MTCC2128) (gram-positive) and Escherichia coli (MTCC2412), Klebsiella pneumonia (MTCC 2451), (gram-negative) by using the agar-well diffusion method. Ciprofloxacin (1 mg/ml) is used as a positive reference. The bacterial strains culture was spread on sterilized Mueller Hinton Agar (MH) plates and incubated for 24 hrs at 37 ºC overnight, following which the diameter of inhibition zone (DIZ) was measured in mm around each well to evaluate the anti-bacterial activity (Ishnava, et al.2014; Magaldiet al., 2004; Valgas et al., 2007)). Experiments were performed in triplicates. Similar procedure was followed for the antifungal activity using Aspergillus niger in Potato Dextrose Agar (PDA). Itraconazole (10mg/ml) was used as the positive control.

Figure S62. Antibacterial activity

Antibacterial activity of selected test samples by disc diffusion method

Bacillus

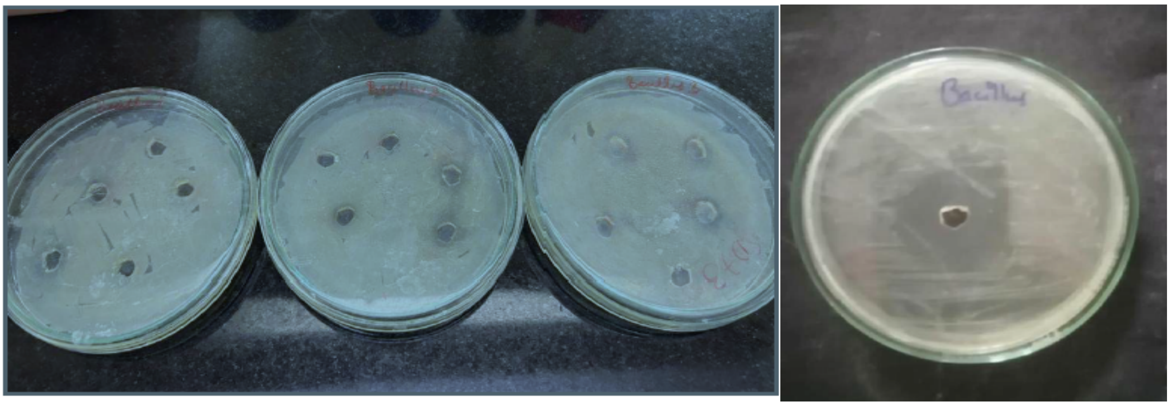

Antibacterial activity of Compound 1 with Bacillus organism at different concentrations

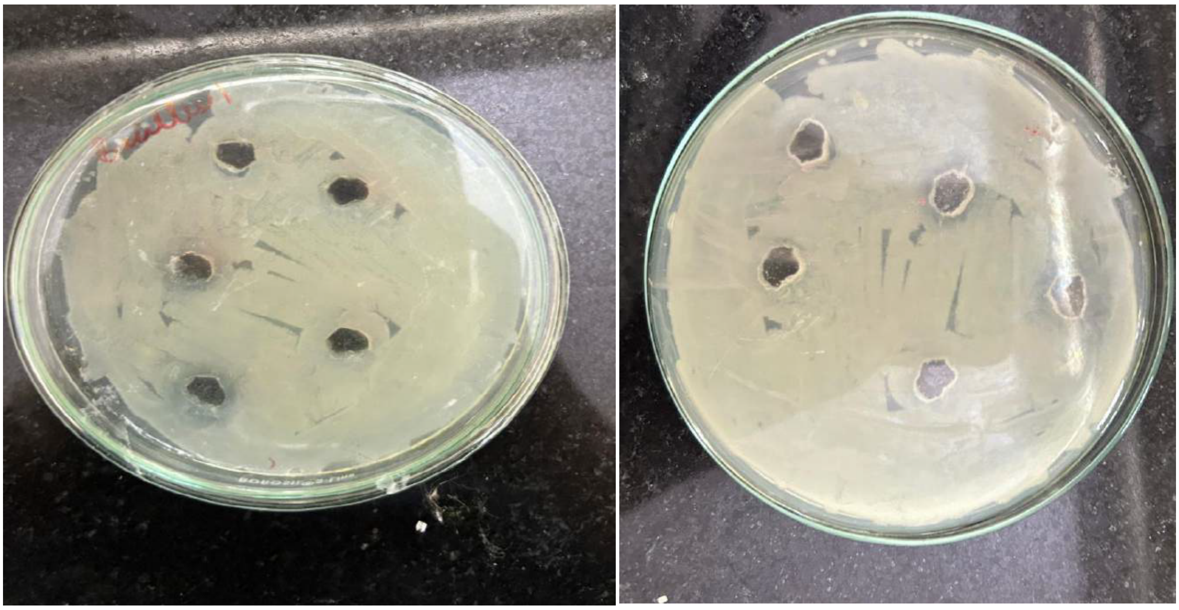

Antibacterial activity of Compound 2 with Bacillus organism at different concentrations

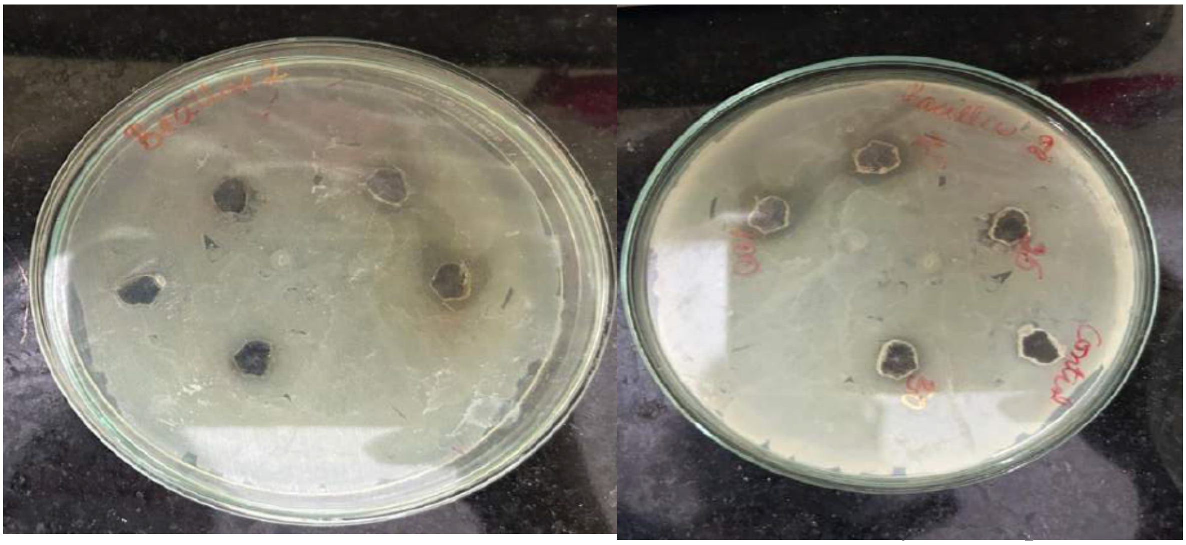

against Bacillus organism at different concentrations

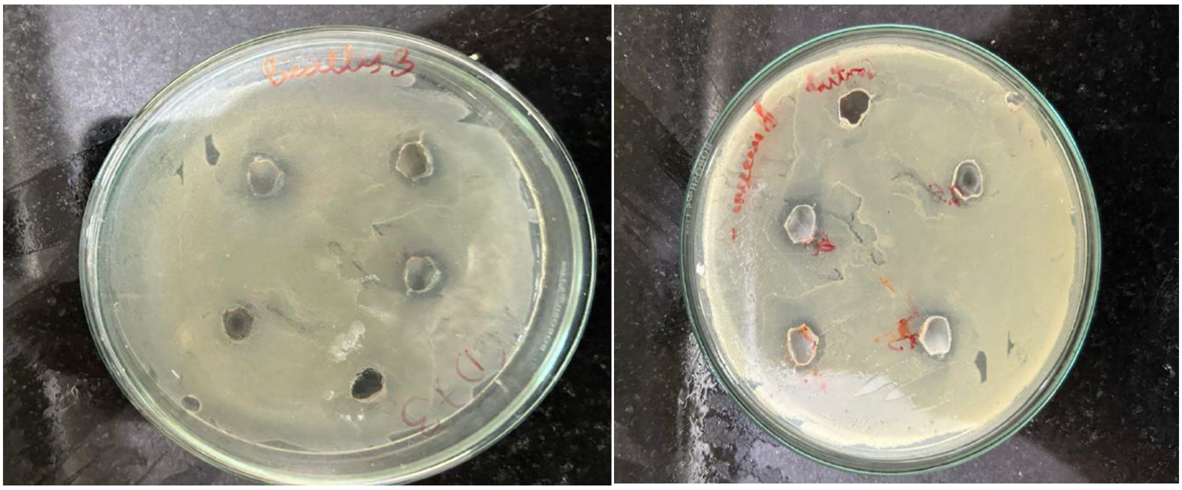

E. coli

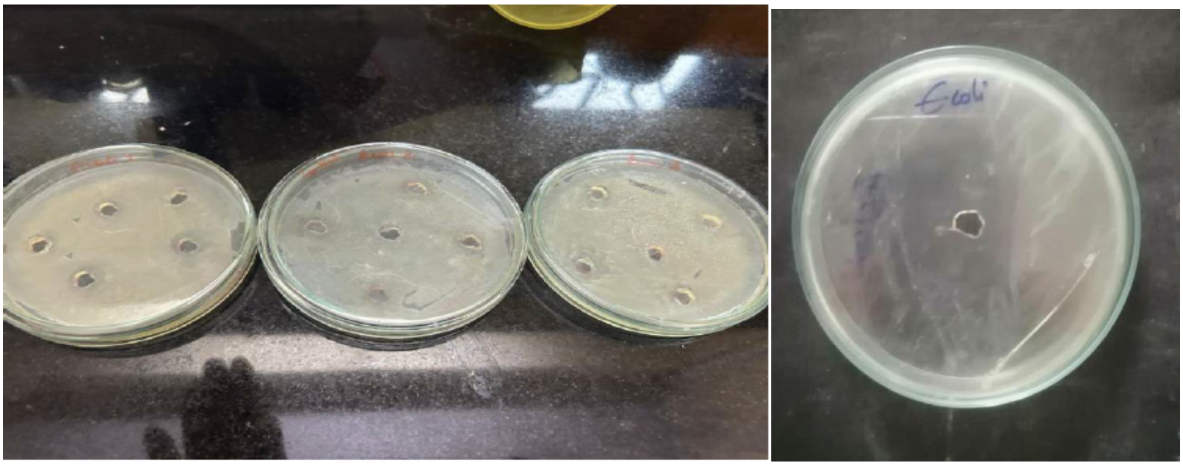

Antibacterial activity of Compound 1 against *E coli* organism at different concentrations

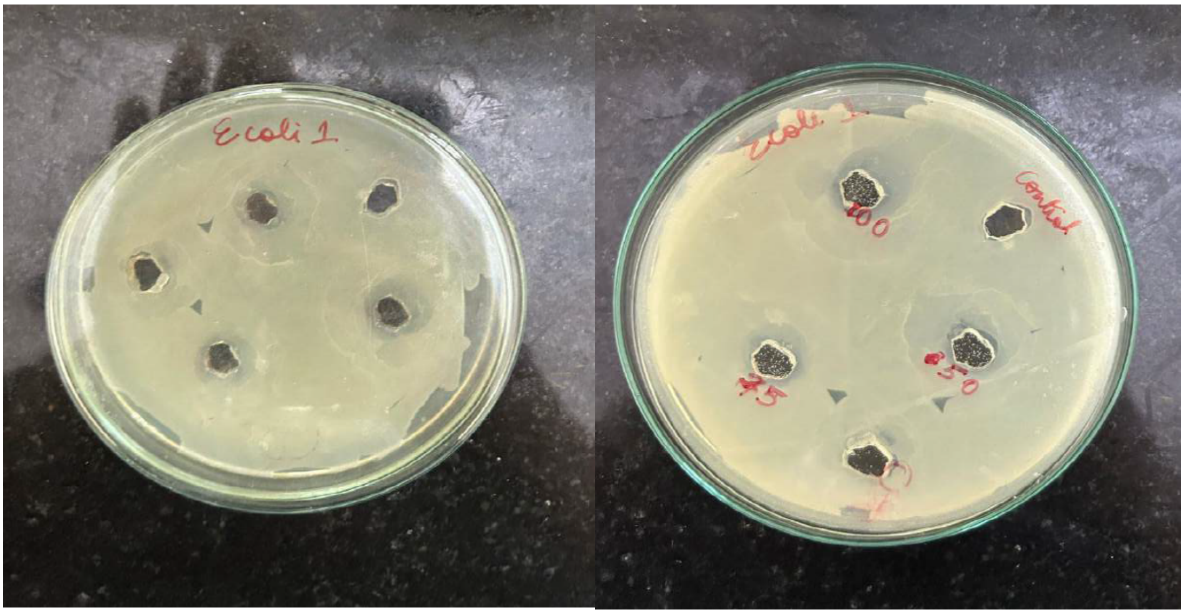

Antibacterial activity of Compound 2 with E coli organism at different concentrations

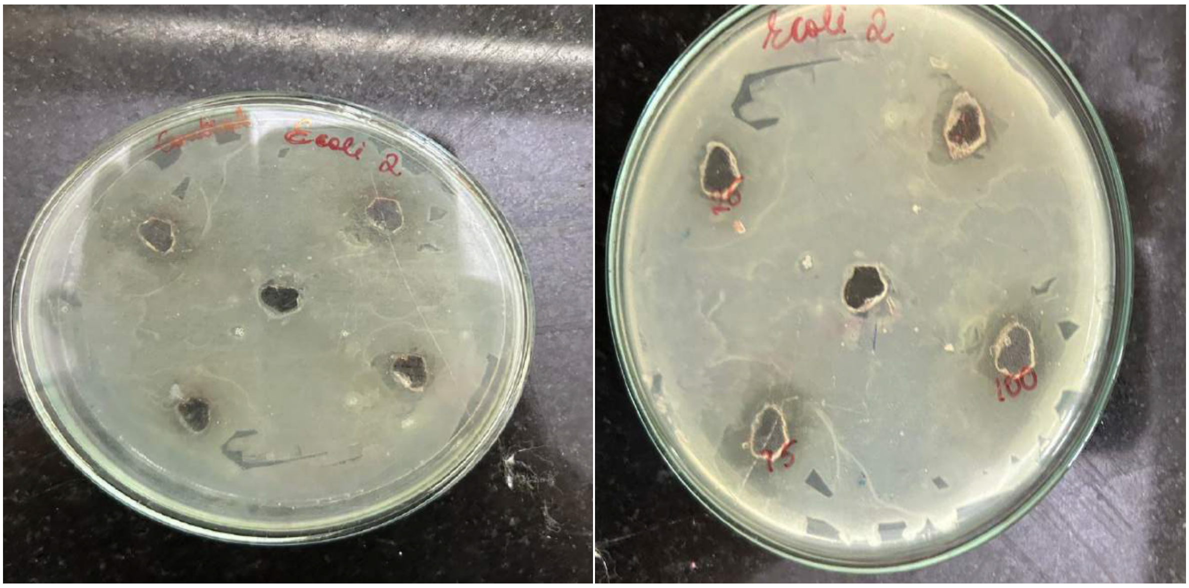

Antibacterial activity of Compound 3 with E coli organism at different concentrations

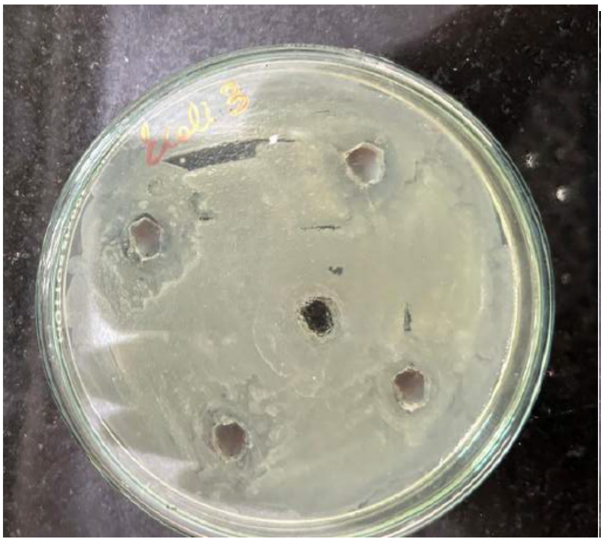

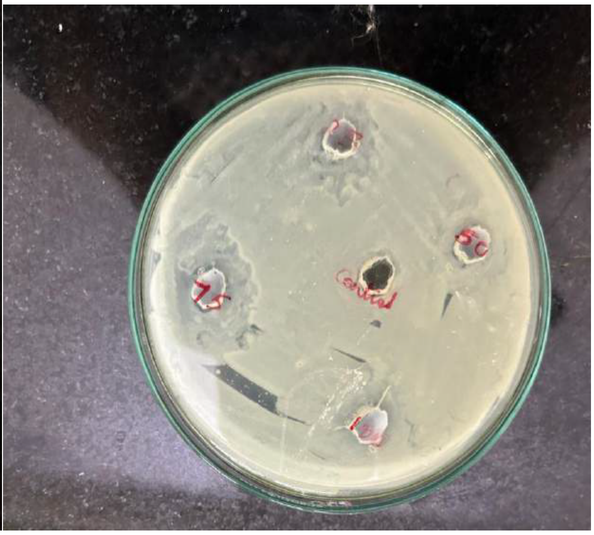

Klebsiella pneumoniae

Anti Bacterial Activvity

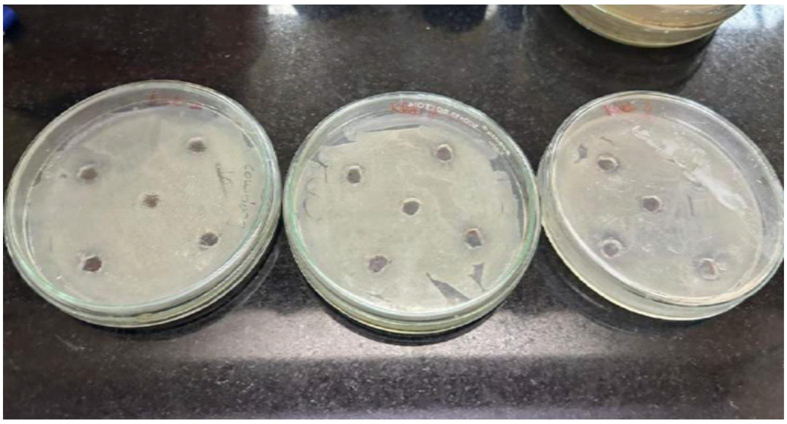

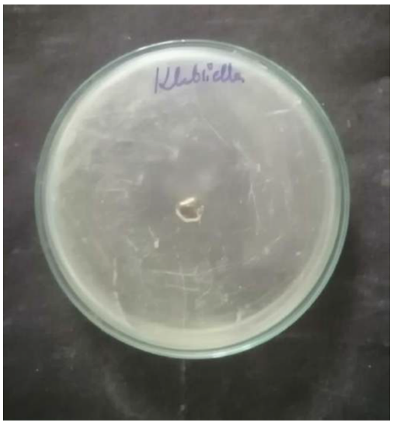

Antibacterial activity of Compound 1 against Klebsiella pneumoniae **at different concentrations**

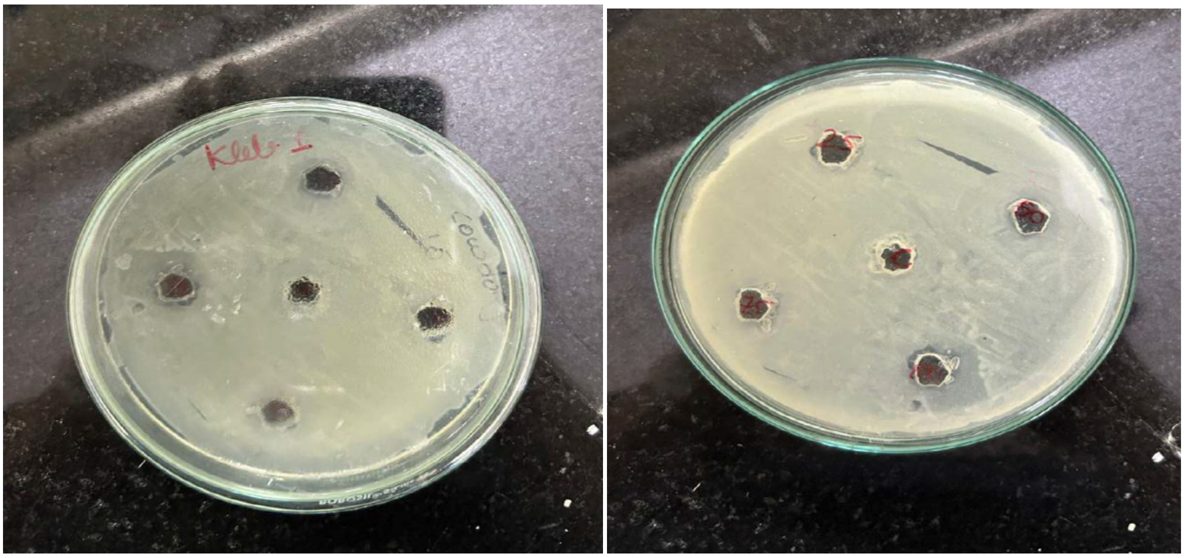

Antibacterial activity of Compound 2 against Klebsiella organism at different concentrations

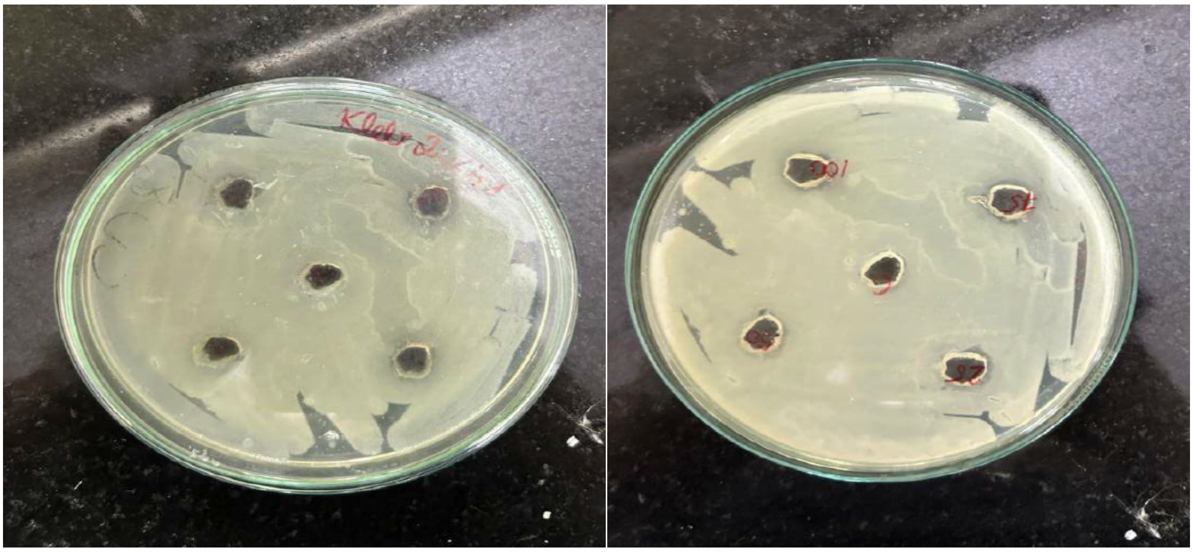

Antibacterial activity of Compound 3 against with Klebsiella organism at different concentrations

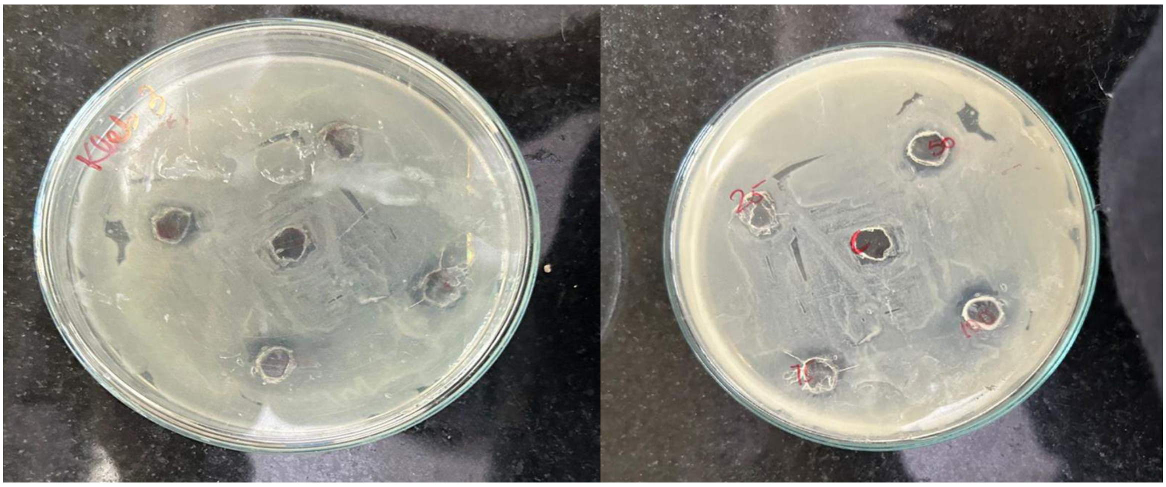

Antibacterial activity of compound 1, 2, 3 against Staphylococcus aureus

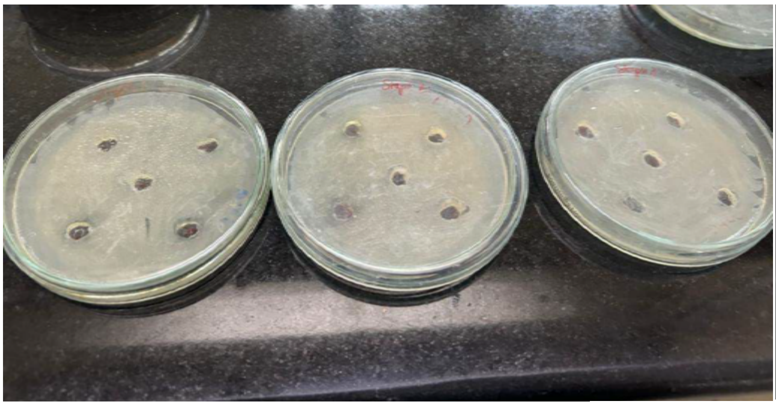

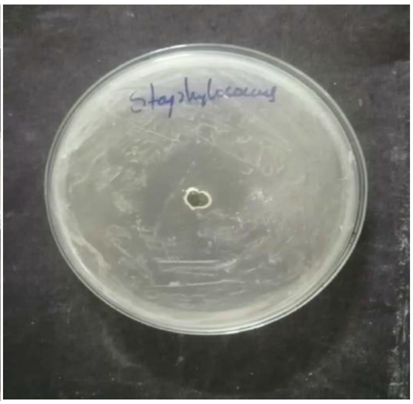

Antibacterial activity of Compound 1 with Staphylococcus aureus at different concentrations

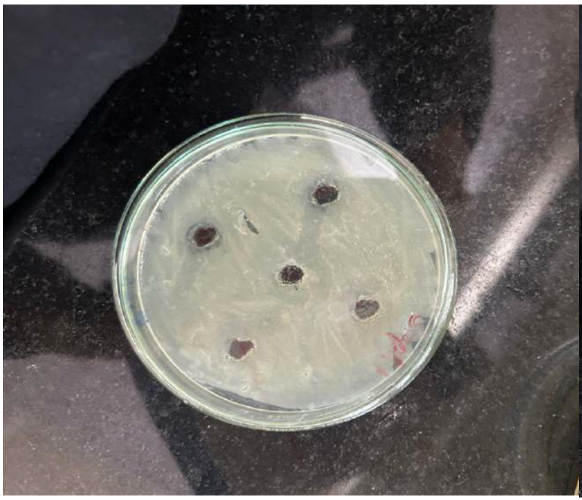

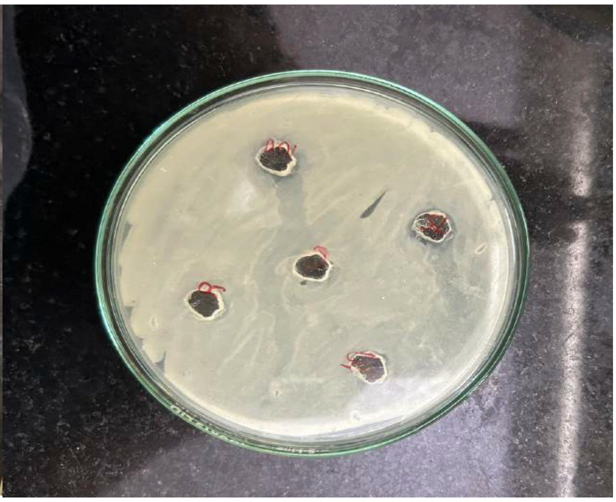

Antibacterial activity of Compound 2 with Staphylococcus aureus at different concentrations

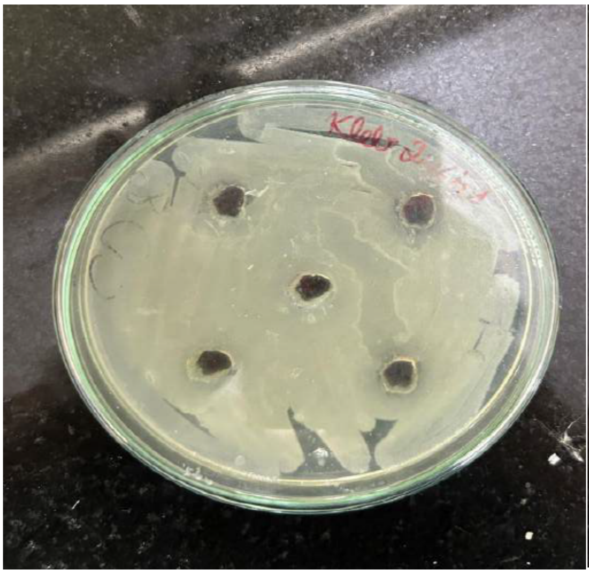

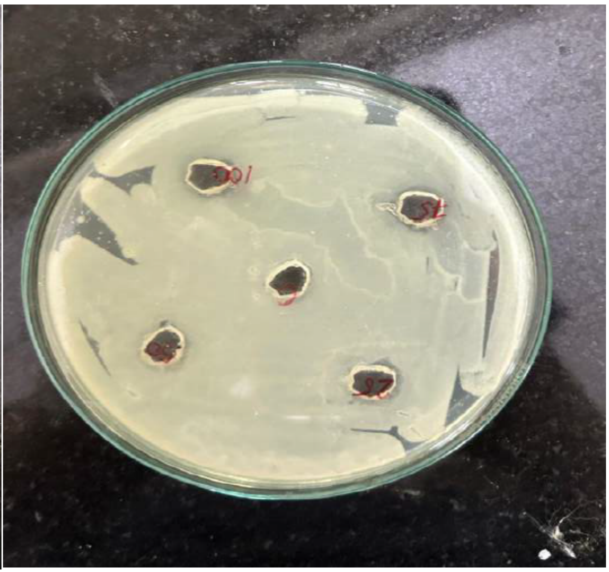

Antibacterial activity of Compound 3 with Staphylococcus aureus at different concentrations

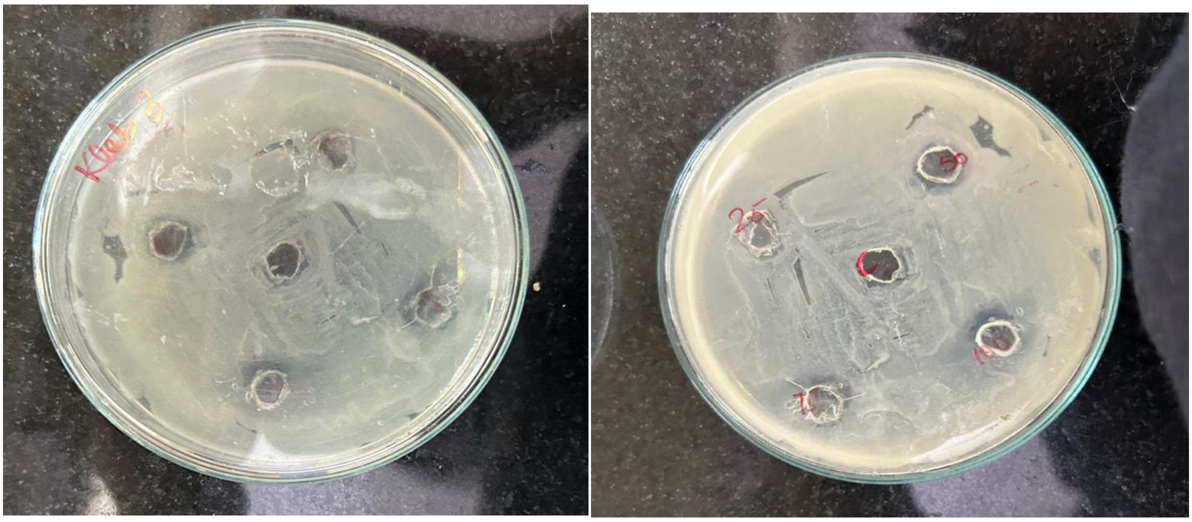

The antibacterial Anti-bacterial activity of activity of the compounds 1, 2 and 3 increases with the increase in concentration of the compounds.

Figure 68. Antifungal activity

None of the test compounds showed any inhibition at 25µl (0.05 mg), 50µl (0.1 mg), 75µl (0.15 mg) and 100 µl (0.2 mg), against Aspergillus niger.

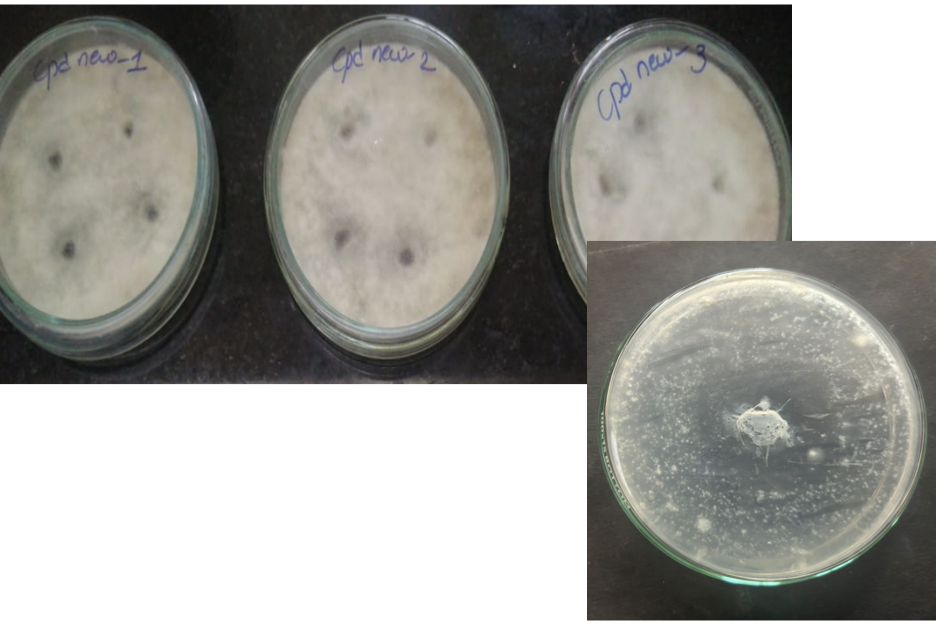

Figure 2. **Antifungal Activity of Compoumds 1, 2, and 3 against *Aspergillus niger***

Figure 69. Anti-mutagenic activity

Comet assay

The comet assay, or single-cell gel electrophoresis (SCGE), is a method used to quantify DNA damage in individual cells. This is a conventional method used to assess DNA damage and repair, monitor biological samples, and conduct tests to determine the potential for genetic damage.

PROTOCOL

The fresh blood was taken and the lymphocytes are extracted. The extracted lymphocytes are used as samples for testing. The collected blood was diluted with phosphate-buffered saline (PBS) and mixed with Ficoll-Hypaque. Afterwards, the blood mixed with Ficoll-Hypaque solution was subjected to centrifugation at a low speed for a duration of 30 minutes in order to separate the cells based on their density. The lymphocytes were retrieved from the intermediate layer following centrifugation (Chiu et al., 2019).

Preparation of slide

1. First Layer: Dust free, plain slides were covered a layer of 140 µl of 0.67% NMA and allowed to dry for 10mins in hot air oven. This layer serves as an anchor for additional layers to prevent slippage.
2. Second Layer: About 110 µl of NMA was layered as second layer and was immediately covered with cover slip and was kept at 4°c for 10mins.
3. Third Layer: 20 µl of blood sample (Approximately 1000-5000 cells) was mixed with 110 µl of warm LMA and mixture was layered as third additional layer and gelled at 4c° for 10mins.
4. Fourth Layer: A fourth additional layer of 110 µl of LMA was added on top and gelled again in the similar way as mentioned above, to sandwich the middle layer and to prevent loss of sample.
5. Lysing: After fourth layer of gel was set, the slides were treated overnight in freshly prepared chilled lysing buffer solution at 4°c with this treatment the cell membrane and nuclear membrane were lysed and the majorities of proteins were removed to expose the nucleosides.
6. Alkali treatment: The slides were then removed from the lysing solution, drained and placed in a horizontal electrophoresis tank side by side avoiding spaces and with agarose end facing the anode. The tank was filled carefully with fresh

electrophoresis buffer to a level approximately 0.25cms above the slides. The slides were left in the high PH (PH>13) buffer for 20mins to allow unwinding DNA and expression of alkali labile site before electrophoresis.

7. Electrophoresis: Electrophoresis was carried out at room temperature for 40mins at 3000mA, 20 V.

8. Neutralizing: After electrophoresis, the slides were flooded 3 times gently with chilled neutralizing solution (Tris PH 7.5) for 5mins so as to remove any traces of detergent and alkali which would otherwise interfere with staining the slides were washed thrice with distilled water, air dried.

9. Silver staining: The slides were silver stained by the method of Ahiya and Saran 1999. Briefly air-dried slides were immersed in the fixing solution for 10mins and washed gently with distilled water several times. The washed slides were allowed to air dry for about 1hour before staining. 68ml of ss (B) was mixed with 32ml of ss (A) and poured over the dried slides so as to cover the slide uniformly. This step was repeated with a fresh mix of ss until a grayish/brakish silver color developed on the slides. No need of stopping solution.

10. Analysis: The assay has the ability to detect differenced between cells in their susceptibility to DNA damage and their subsequent repair response which may vary with their proliferative or differentiative status. Hundred consecutive cells (50 cells from each end of slide) were manually selected and quantified with, which also determined the olive tail moment parameter.

[(Tail mean-head mean) (% tail DNA /100)] used to quantify DNA damage.

The head of comet in the nucleus of the cell and the tail of the comet is damaged DNA that has been liberated from the nucleus by electrophoresis. The tail mean is the tail DNA intensity subtracted from background intensity, the head mean is the head DNA intensity subtracted from background intensity and percentage of tail DNA is the fraction of DNA that has migrated from the head. The difference between the tail mean and head mean represents the difference in the distance between center of gravity of DNA distribution in comet head and center of gravity of DNA distribution in comet tail (S. Nandan et al., 2012).

References

1) F. Robert, B. Mootoo, R. Ramsewak, A. Khan, A. Ramsubhag, W. Reynolds and M. Nair, *Pest Management Science* ., 2010, **66**, 1298-1303, DOI:10.1002/ps.2013.

2) B. S. Mootoo, A. Ali, R. Motilal, R. Pingal, A. Ramlal, A. Khan, W. F. Reynolds and S. McLean, J. *Nat. Prod .,* 1999, **62** (11), 1514–1517, DOI:10.1021/np990199x.

3) A. K. M. S. Rahman, A. K. A. Chowdhury, H. –A. Ali, S. Z. Raihan, M.S. Ali, L. Nahar and S. D. Sarker, *Journal of Natural Medicines.,* 2009, **63**, 41-45, DOI:10.1007/s11418-008-0287-3.

4) L. –C. Chen, H. –R. Liao, P.-Y. Chen, W. -L. Kuo, T. –H. Chang, P. –J. Sung, Z. –H. Wen and J. –J. Chen, *Molecules.,* 2015, **20**, 18551-18564, DOI: 10.3390/molecules201018551.

5) Chen, Jih-Jung; Huang, Sheng-Shiou; Liao, Chang-Hui; Wei, Dau-Chang; Sung, Ping-Jyun; Wang, Tai-Chi; Cheng, Ming-Jen. *Food Chemistry.,* 2010, **120**, 379-384, DOI: 10.1016/j.foodchem.2009.09.093.

6) A. H. Nour, Mohammed, B. Sulieman, M. Yousf, Abdurahman, Adam, M. Mazza, *Australian Journal of Basic and Applied Sciences.,* 2016, **10**(17Spec.Iss.), 55-62.

7) Y.-B. Cheng, Y.-T. Chien, J.-C. Lee, C.-K. Tseng, H.-C. Wang, I.-W. Lo, Y. –H. Wu, S.-Y. Wang, Y.-C. Wu and F.-R. Chang, *J.Nat.Prod.,* 2014, **77**, 2367-2374, DOI: 10.1021/np5002829.

8) Y.-Q. Ma, K. Jiang, Y. Deng, L. Guo, Y.-Q. Wan, C.-H. Tan, *Journal of Asian Natural Products Research.,* 2018, **20**, 299-305, DOI:10.1080/10286020.2017.1335715.

9) B. H. Goh, H. A. Kadir, S. N. A. Malek and S. W. Ng, *Acta Crystallographica.,* 2010, **66**(6), o1396, DOI:10.1107/S1600536810017733.

10) C. K. Mahendra, H. L. Ser, S. Z. Abidin, S. U. Khan, P. Pusparajah, T. T. Htar, L. H. Chuah, S. Y. Tang, Siah Ying; Ming, Long Chiau; Goh, Khang Wen, *Biomedicine & Pharmacotherapy.,* 2023, **162**, 114659, DOI: 10.1016/j.biopha.2023.114659.

11) J. Zhang, S.-X. Yang, X.-B. Yang, M.-Y. Li, G. Feng, J.-Y. Pan and T. Satyanandamurty, *Chemical & Pharmaceutical Bulletin.,* 2010, **58**(4), 552-555, DOI:10.1248/cpb.58.552.

12) G. Robert, Mootoo, S. Baldwin, Ramsewak, S. Russel. Khan and Ayub, *Pharmaceutical Biology.,* 2012, **50**(2), 264-267, DOI: 10.3109/13880209.2011.581670.
